# Adapted problem adaptation therapy for depression in mild to moderate Alzheimer's disease dementia: A randomized controlled trial

**DOI:** 10.1002/alz.13766

**Published:** 2024-03-13

**Authors:** Robert Howard, Elizabeth Cort, Charlotte Rawlinson, Martin Wiegand, Anne Downey, Vanessa Lawrence, Sube Banerjee, Peter Bentham, Chris Fox, Rowan Harwood, Rachel Hunter, Gill Livingston, Esme Moniz‐Cook, Monica Panca, Malgorzata Raczek, Chineze Ivenso, Gregor Russell, Alan Thomas, Philip Wilkinson, Nicholas Freemantle, Rebecca Gould

**Affiliations:** ^1^ Division of Psychiatry University College London London UK; ^2^ Priment Clinical Trials Unit University College London London UK; ^3^ David Goldberg Centre (H1.01) King's College London London UK; ^4^ University of Plymouth Plymouth Devon UK; ^5^ Birmingham and Solihull NHS Trust Birmingham UK; ^6^ University of East Anglia Norwich Norfolk UK; ^7^ University of Nottingham Queen's Medical Centre Nottingham UK; ^8^ University of Hull Hull UK; ^9^ Brighton and Sussex School of Medicine Brighton East Sussex UK; ^10^ Aneurin Bevan NHS Trust St Cadoc's Hospital Newport South Wales UK; ^11^ Bradford District Care Foundation Trust Shipley UK; ^12^ University of Newcastle Campus for Ageing and Vitality Newcastle upon Tyne UK; ^13^ Department of Psychiatry University of Oxford Warneford Hospital Oxford UK; ^14^ Comprehensive Clinical Trials Unit University College London London UK

**Keywords:** Alzheimer's disease, clinical trial, depression, mood, problem adaptation therapy, psychological, psychotherapy

## Abstract

**INTRODUCTION:**

Trials of effectiveness of treatment options for depression in dementia are an important priority.

**METHODS:**

Randomized controlled trial to assess adapted Problem Adaptation Therapy (PATH) for depression in mild/moderate dementia caused by Alzheimer's disease.

**RESULTS:**

Three hundred thirty‐six participants with mild or moderate dementia, >7 on Cornell Scale for Depression in Dementia (CSDD), randomized to adapted PATH or treatment as usual. Mean age 77.0 years, 39.0% males, mean Mini‐Mental State Examination 21.6, mean CSDD 12.9. For primary outcome (CSDD at 6 months), no statistically significant benefit with adapted PATH on the CSDD (6 months: −0.58; 95% CI −1.71 to 0.54). The CSDD at 3 months showed a small benefit with adapted PATH (−1.38; 95% CI −2.54 to −0.21) as did the EQ‐5D (−4.97; 95% CI −9.46 to −0.48).

**DISCUSSION:**

An eight‐session course of adapted PATH plus two booster sessions administered within NHS dementia services was not effective treatment for depression in people with mild and moderate dementia. Future studies should examine the effect of more intensive and longer‐term therapy.

## 1 BACKGROUND

Depression is common in dementia, with meta‐analysis suggesting that 14.8% people with Alzheimer's disease (AD) have a major depressive disorder.^1^ Depression in dementia reduces quality of life and functional abilities^2^, ^3^ and increases caregiver burden.^4^ There is little evidence for therapeutic efficacy of antidepressant drugs in dementia,^5^ with one typical trial showing that improvements in depression scores at 13 and 39 weeks did not differ between participants with major depressive disorder allocated to receive antidepressant or placebo.^6^ A systematic review within which no studies were considered at low risk of bias, concluded that cognitive behavioral therapies are probably slightly better than treatment as usual or active control for reducing depressive symptoms in dementia (SMD −0.23, 95% confidence interval [CI] −0.37 to −0.10; 13 trials, 893 participants; moderate‐certainty evidence).^7^ A network meta‐analysis found non‐drug interventions (cognitive stimulation with or without a cholinesterase inhibitor, massage and touch, multidisciplinary care, occupational therapy, exercise combined with social interaction and cognitive stimulation, and reminiscence therapy) were more efficacious than drug interventions for reducing symptoms of depression in people with dementia who did not have a major depressive disorder.^8^

Problem Adaptation Therapy (PATH) aims to improve emotion regulation through situation selection and modification, attentional deployment, cognitive change, and response modulation, using a problem‐solving approach and caregiver participation. PATH has been reported to improve depression symptoms with a moderate effect size (Cohen's d 0.60; 95% CI 0.13 to 1.06) during a 12‐week randomized controlled trial (RCT) in 74 patients with major depression and cognitive impairment (Mini‐Mental State Examination [MMSE]^9^ score of 17 or greater), only some of whom were affected by mild‐to‐moderate dementia.^10^ PATH has been further adapted for the current study, using a person‐centered qualitative approach by members of the original PATH team and PATHFINDER investigators, specifically for use with people with the full range of cognitive impairment in moderate dementia and major depression.^11^ We have conducted an RCT in patients with mild to moderate dementia (MMSE score 10 or greater) caused by AD, who also had symptoms consistent with major depression, to investigate the clinical and cost‐effectiveness of eight sessions in 12 weeks of treatment with adapted PATH plus two booster sessions over 12 months of follow‐up.

## 2 METHODS

### 2.1 Study design

PATHFINDER (Problem Adaptation Therapy for Individuals with Mild to Moderate Dementia and Depression) was a multicenter, single‐blind, parallel, two‐arm, RCT to assess the clinical and cost‐effectiveness of an adapted PATH intervention for depression in mild to moderate dementia caused by AD. It was conducted in 24 centers in England and Wales. Ethical approval was given by Wales Research Ethics Committee 4 Wrexham on June 14, 2018, IRAS ID 238724. The trial was preregistered with the ISRCTN Registry on May 31, 2018 (ISRCTN11185706). The trial protocol is included in Supplemental materials.

### 2.2 Participants

Patients were recruited from National Health Service (NHS) memory services, community mental health services for older people, primary care, and third sector services for people with dementia. Inclusion criteria were: (1) diagnosis of probable AD or mixed AD and vascular dementia using National Institute on Aging and Alzheimer's Association (NIA‐AA) criteria^12^; (2) mild to moderate dementia severity, defined by Standardized Mini‐Mental State Examination (sMMSE)^13^ score of at least 10; (3) clinically significant depression, defined by score of 8 or more on Cornell Scale for Depression in Dementia (CSDD)^14^; (4) aged > 50 years; (5) sufficiently fluent in English to engage with the intervention; (6) identified caregiver who spends > 1 h per day on at least 3 days/week with participant and agrees to act as co‐therapist for intervention; (7) living in their own household (i.e., not in residential care); (8) participant had been informed of their dementia diagnosis. Exclusion criteria were: (1) diagnosis of other dementias, including dementia with Lewy bodies, Parkinson's disease dementia, and frontotemporal dementia; (2) initiation of prescription or change in dose of antidepressant or other psychotropic medication in previous 4 weeks or plan to change treatment in the next 12 weeks; (3) current or planned formal psychological therapy; (4) requiring treatment for a severe psychiatric disorder such as schizophrenia or bipolar disorder; (5) severe depression and expressing suicidal ideation with active plans or suicidal intent and behaviors. Participating patients and their caregivers gave written informed consent for inclusion.

### 2.3 Randomization and masking

Participants were randomized 1:1 between the adapted PATH intervention and treatment as usual (TAU) using a Web‐based secure randomization service (https://www.sealedenvelope.com/). Randomization was stratified by baseline use of antidepressant medication. Outcomes assessors and central trial staff were blinded to treatment allocation.

### 2.4 Intervention

The adapted PATH intervention^11^ consisted of up to eight manualized (manual included in Supplemental materials) 1‐hour sessions involving a trained and supervised therapist, the patient participant and the caregiver, over 12 weeks, with therapy comprising two assessment sessions, five sessions focused on problem solving using PATH tools, and one review session. This was supplemented by 1‐hour booster sessions at 6 and 9 months, which reviewed key problem‐solving and emotional regulation strategies used in PATH. Caregivers were involved as co‐therapists to help to identify problems that were maintaining depression, meaningful pleasurable activities that the person with dementia had previously enjoyed, solutions to the problems, and helping the person with dementia use PATH tools to overcome these problems and engage in pleasurable activities. As part of the PATH adaptation, themes were identified that typified the experience of depression in people with dementia, including sense of isolation and role loss, feelings of being a burden and misunderstood, interpersonal tensions, diverging views among carers and patients about capabilities and changeability in abilities and mood.^11^ Although it had been intended that sessions would be delivered face‐to‐face, the coronavirus disease 2019 (COVID‐19) pandemic considerations meant that they were conducted for some participants over the telephone or by videoconferencing using MS Teams or Attend Anywhere with the patient participant and caregiver, depending on national guidance from March 2020 onward. Adapted PATH was designed to be scaled up easily within the NHS using existing staff. Therapists were nurses, assistant psychologists, occupational therapists, psychiatrists, and clinical psychologists based in memory services and community mental health teams. Therapists attended a 1‐day training workshop, developed and delivered by the research team and supported by a Manual (included in supplemental materials). Fortnightly supervision was provided locally throughout the intervention delivery period by clinical psychologists who had also attended the 1‐day training workshop. In addition, therapists were invited to attend fortnightly group supervision via video call, while supervisors were offered monthly group consultation via video call, both of which were provided by members of the research team. All therapy sessions were recorded and 10% of recordings were rated for adherence to the therapy manual. Treatment fidelity was assessed using a Treatment Adherence Rating Scale (see Supplemental materials), a 13‐item adherence measure developed specifically for this trial. Nine items examined adherence to key PATH components, two items adherence to general therapeutic components, and two global ratings assessed overall adherence and competence. Items were rated on a 5‐point Likert scale from 1 = very poor to 5 = very good. Sessions were rated by four independent therapists who had received adapted PATH training.

Participants allocated to receive treatment as usual received usual follow‐up and support from memory services, community mental health teams, primary care, social care and the third sector.

**RESEARCH IN CONTEXT**

1. **Systematic review**: The authors reviewed the literature on treatment of depression in Alzheimer's disease (AD) dementia. Published systematic reviews indicate little advantage for antidepressants or psychotherapies over placebo.
2. **Interpretation**: An adapted Problem Adaptation Therapy (PATH) intervention, delivered by dementia services' usual Staff, supported by a manual, brief training and supervision, can modestly improve depression symptoms in AD over 12 weeks of the therapy, but effects do not persist after therapy discontinuation.
3. **Future directions**: Symptoms of depression in dementia represent an important treatment target with a realistic prospect of therapeutic success. Future trials should be conducted to investigate the effectiveness of longer durations of adapted PATH, with and without combination treatment with antidepressants.

### 2.5 Outcomes

The primary outcome was the Cornell Scale for Depression in Dementia (CSDD)^14^ score at 6 months post‐randomization. This is a 19‐item scale on which each item is rated by a combination of observed and informant‐based scores as 0 (absent), 1 (mild or intermittent), or 2 (severe) and covers mood‐related signs, behavioral disturbance, physical signs, cyclic functions, and ideational disturbance. Secondary outcomes included: CSDD score at 3 and 12 months post‐randomization; dementia‐specific quality of life measured using DEMQOL and DEMQOL‐Proxy^15^ interviewer‐administered self‐ and informant‐ratings of the quality of life of the person with dementia in the previous week; generic health‐related quality of life with EQ‐5D,^16^ a 5‐item self‐report measure of health‐related quality of life used to calculate utility scores and where the main item used for analysis is Question 6, where participants are asked to assess their overall health on a scale of 0–100; functional abilities with the Bristol Activities of Daily Living Scale (BADLS),^17^ a 20‐item scale completed with the caregiver and covering everyday daily living activities; cognitive functioning with the sMMSE,^13^ anxiety with the Rating Anxiety in Dementia scale (RAID),^18^ a 20‐item scale assessing individual anxiety symptoms; caregiver burden and wellbeing with the Zarit Burden Inventory,^19^ a 22‐item self‐report measure for caregivers and General Health Questionnaire (GHQ‐12),^20^ sensitive to the detection of minor psychiatric disorder through assessment of change in respondents’ state; resource use was collected with the Client Service Receipt Inventory (CSRI) for Health and Social Care Resource Use,^21^ a measure of service utilization used to calculate patient and caregiver costs.

### 2.6 Statistical analysis

Pre‐study power calculations estimated that a sample size of 334 participants was required to allow for detection of a 0.4 SD effect size (corresponding to a 2.0‐point difference in CSDD score, considered to be a minimum clinically important difference),^8^ with a two‐sided alpha of 5% and 90% power, allowing an additional 20% for loss to follow up or other methodological challenges.

The prespecified primary outcome analysis used a mixed effects model, to compare the mean effect of treatment with adapted PATH compared to treatment as usual. The main explanatory variables were assigned study arm, baseline use of antidepressant medication (stratification factor) and baseline CSDD score. Potential clustering within study site was accounted for by random intercepts for sites. The primary analysis was by intention to treat and accounted for baseline. Patients with missing values or values collected outside the prespecified −2/+4‐week time window around scheduled follow‐up at either time point were not included in the primary analysis. Data extraction was carried out with STATA version 17, data processing and analysis were done in R version 3.4.3. Additional analysis and verification of the primary analysis were done with SAS 9.4 (SAS Institute, Cary NC). The Statistical Analysis Plan was version 0.6 dated January 20, 2023 (included inSupplemental materials).

### 2.7 Role of the funding source

The study was funded by the National Institute for Health Research Health Technology Assessment Programme (Grant Reference 16/155/01) in response to a commissioned call to develop an intervention based on PATH to treat depression in people with mild to moderate dementia within the UK NHS and to test clinical and cost‐effectiveness in a multicenter RCT.

## 3 RESULTS

**FIGURE 1 alz13766-fig-0001:**
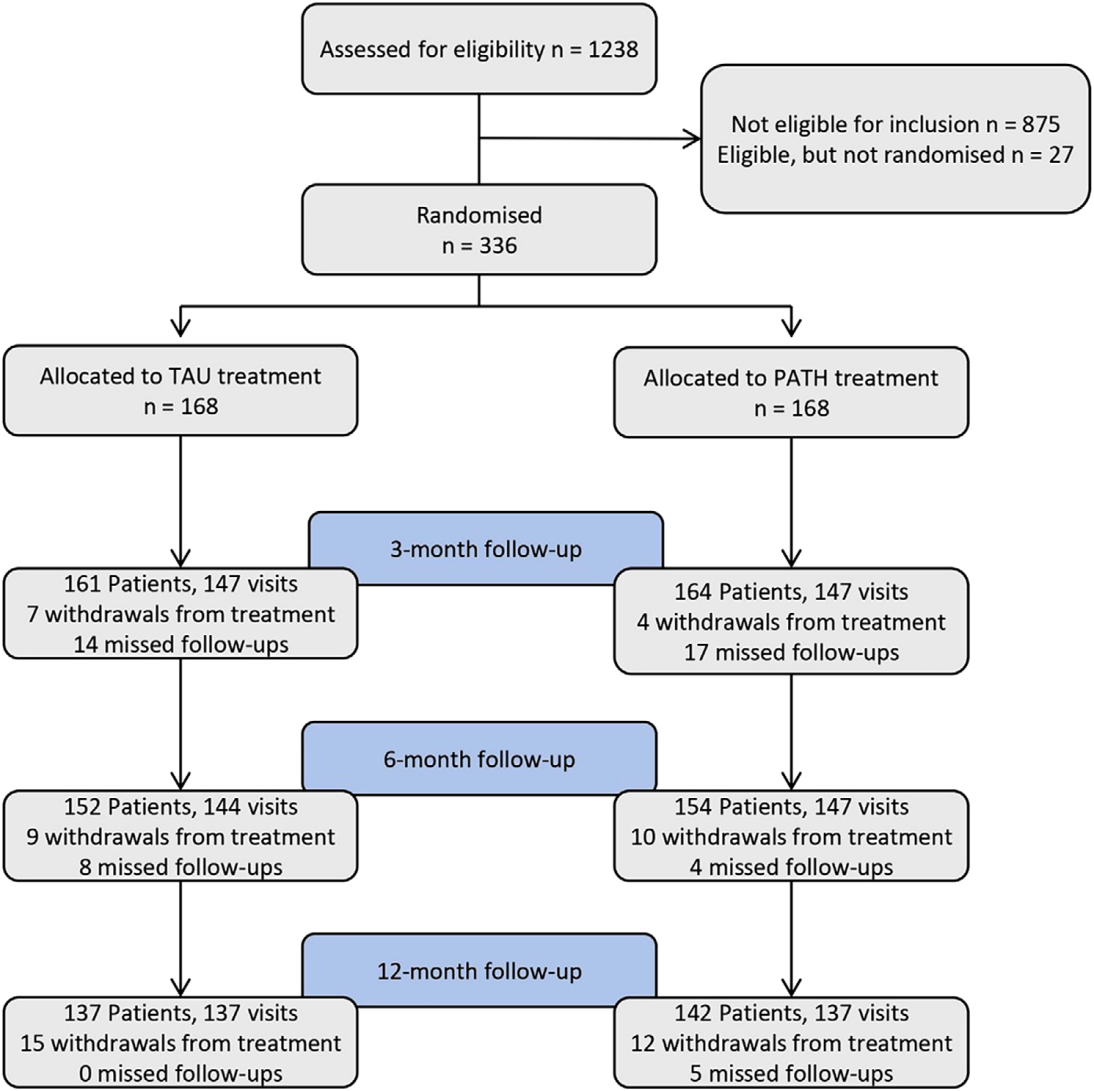
Participant flow through the study

A total of 1238 patients were screened for eligibility, of whom 363 met inclusion criteria and 336 participants were randomized 1:1 to adapted PATH and TAU between September 24, 2019 and January 22, 2022. Table 1 presents baseline characteristics of participants randomized to adapted PATH and TAU. Participant flow and numbers through the study are captured by a CONSORT diagram in Figure 1.

**TABLE 1:** Baseline characteristics of participants randomized to adapted PATH and treatment as usual

Parameter	PATH Allocation	TAU Allocation
**Demographics**		
N	168	168
Age, Median [IQR] (Range)	78 [7383] (5497)	76 [7183.25] (5294)
Gender (male), *n* (%)	66 (39.3%)	65 (38.7%)
**Ethnicity**		
White British	148 (88.1%)	148 (88.1%)
White Irish	2 (1.2%)	5 (3.0%)
White Other	6 (3.6%)	7 (4.2%)
White & Black Caribbean	1 (0.6%)	1 (0.6%)
Other mixed background	1 (0.6%)	1 (0.6%)
Indian	2 (1.2%)	0 (0%)
Caribbean	1 (0.6%)	2 (1.2%)
African	1 (0.6%)	0 (0%)
Black Other	1 (0.6%)	0 (0%)
White and Asian	0 (0%)	1 (0.6%)
Other Asian background	0 (0%)	2 (1.2%)
Other	5 (3.0%)	1 (0.6%)
**Education**		
Higher degree	8 (4.8%)	12 (7.1%)
Degree	20 (11.9%)	24 (14.3%)
A level (or equivalent)	16 (9.5%)	10 (6.0%)
HNC/HND (or equivalent)	18 (10.7%)	15 (8.9%)
NVQ (or equivalent)	18 (10.7%)	12 (7.1%)
GCSE (or equivalent)	29 (17.3%)	28 (16.7%)
School Leaving Certificate	18 (10.7%)	26 (15.5%)
No formal qualifications	41 (24.4%)	41 (24.4%)
**Marital status**		
Married	105 (62.5%)	110 (65.5%)
Divorced	14 (8.3%)	7 (4.2%)
Single	3 (1.8%)	3 (1.8%)
Cohabiting	6 (3.6%)	5 (3.0%)
Widowed	40 (23.8%)	39 (23.2%)
Other	0 (0%)	4 (2.4%)
**Baseline antidepressant use**		
Antidepressant prescription	94 (56.0%)	93 (55.4%)
**Score at baseline**		
CSDD at baseline	13 (3.8)	12.8 (3.8)
Adjusted sMMSE at baseline	21.9 (4.52)	21.2 (4.68)

Abbreviation: CSDD, Cornell Score for Depression in Dementia; HNC, Higher National Certificate; HND, Higher National Diploma; GCSE, General Certificate of Secondary Education; NVQ, National Vocational Qualification; sSSME, Standardized Mini‐Mental State Examination.

**FIGURE 2 alz13766-fig-0002:**
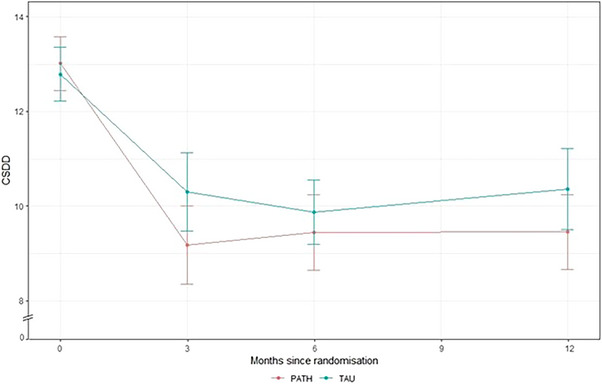
Cornell Scale for Depression in Dementia (CSDD) scores at each time point, stratified by treatment allocation. Error bars are 95% confidence intervals

The adapted PATH group had a mean baseline CSDD score of 13 (SD 3.75) and the TAU group 12.8 (SD 3.75). Six months after randomization, the mean CSDD score was 9.45 (SD 5.26) for the adapted PATH group and 10.3 (SD 5.48) for the TAU group. Mean CSDD scores over the 12 months of study follow‐up are shown in Figure 2. The effect of being allocated to adapted PATH was −0.58 (95% CI −1.71 to 0.54).

**FIGURE 3 alz13766-fig-0003:**
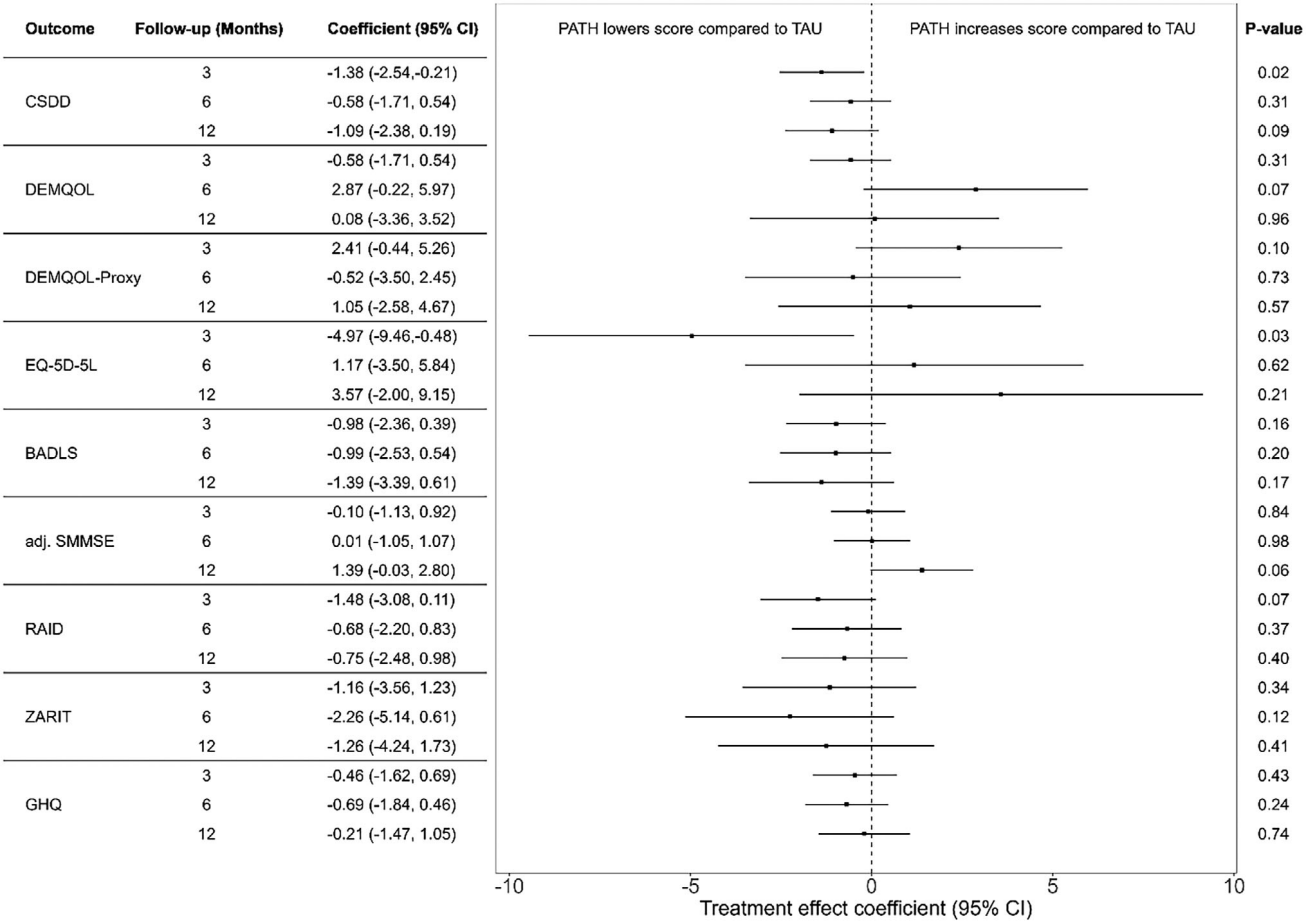
Table and forest plot of the treatment effects of all outcomes

The analyses of all outcomes at the three time points are compiled in Figure 3. The table on the left shows the treatment effect coefficients of adapted PATH for the respective outcomes and time points, with the corresponding 95% CI. The forest plot in the center visualizes the treatment coefficient as a point and the CI as a line. The dotted line marks 0, which would be a treatment effect that is equal to TAU. Negative values imply a stronger treatment effect compared to TAU, and positive values the opposite direction of effect.

A total of 56 serious adverse events (SAEs) were reported for 30 patients in the adapted PATH group, and 53 for 35 patients in the TAU group. None of the SAEs was deemed related to the treatment.

Due to the COVID‐19 pandemic, the trial was affected by social distancing rules, which necessitated delivery of adapted PATH remotely or as a mixture with face‐to‐face sessions. To investigate whether the mode of delivery affected the treatment effect, we stratified the treatment into three categories of face‐to‐face sessions, hybrid sessions, and remote only sessions, with TAU being the alternative. We observed coefficients of −0.78 (95% CI −2.45 to 0.91), −1.00 (95% CI −2.63 to 0.62), and −0.59 (95% CI −2.09 to 0.91) for remote sessions, hybrid sessions, and face‐to‐face sessions, respectively. Face‐to‐face sessions appeared no more effective than remotely delivered therapy sessions.

We also investigated if patients who did not receive the intended eight therapy sessions might have a diminished treatment effect. In total 41 (31%) out of 133 patients assigned adapted PATH did not receive the intended eight sessions. We introduced an interaction term of number of completed adapted PATH sessions and treatment allocation into the primary model. The interaction coefficient was −0.67 (95% CI −1.86 to 0.52), which did not suggest that the number of completed sessions impacted the treatment effect of adapted PATH compared to TAU.

Patient motivation can play an important role in the success of interactive treatments such as a talking therapy that requires patient engagement. Participants were asked that if given the opportunity to choose between adapted PATH or TAU, on a scale from 0 to 3, how willing they would be to participate in adapted PATH. The coefficient of the interaction term provides no support for the hypothesis that motivation may have affected treatment effect.

**FIGURE 4:**
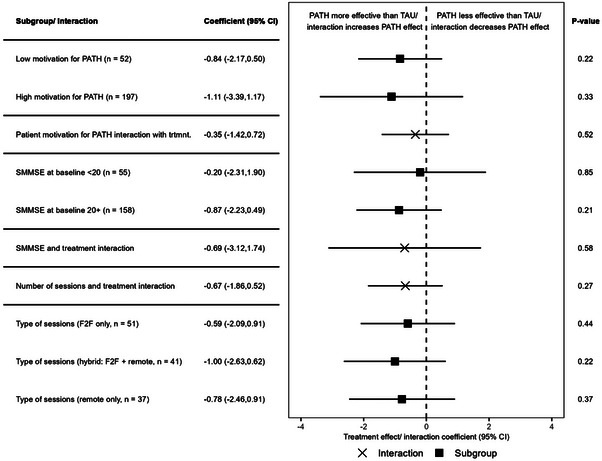
Table and forest plot of the treatment and interaction coefficients of the exploratory analyses

We[Fig alz13766-fig-0001], [Fig alz13766-fig-0002], [Fig alz13766-fig-0003] investigated whether the treatment effect was affected by participants’ degree of cognitive impairment. We used baseline sMMSE to stratify patients into more severely cognitively impaired patients (sMMSE score < 20) and less impaired patients (sMMSE score > 20). These two categories were introduced as an additional covariate in the primary outcome model, complemented by an interaction term with the treatment indicator. We also stratified the primary outcome analysis by binary sMMSE group and compared the coefficients of the two separate models. While the coefficients for the treatment group appear to differ in magnitude, neither was significant at the 5% threshold. The CIs surrounding the coefficients include 0, and we cannot conclude that adapted PATH was more effective than TAU in either group. Similarly, the interaction term of sMMSE group and treatment allocation was not significantly different from 0 and, thus, does not support the hypothesis that sMMSE level affected treatment effect. Figure 4 displays a table and forest plots of the treatment and interaction coefficients of the exploratory analyses.

Finally, we performed a post hoc sensitivity analysis to determine whether the effect of PATH was modified by baseline CSDD score. High or low baseline scores were defined as being above or below the median of 12. Repeating the primary analysis accounting for baseline CSDD, treatment allocation, medication status and an interaction term of binary CSDD level did not indicate a treatment effect modification (interaction coefficient −0.51 [−2.74 to 1.73] *p* = 0.65). 11.2% (*N* = 145/1295) of sessions were rated for treatment adherence using the Treatment Adherence Rating Scale. Treatment adherence was high: the mean overall adherence score was 3.9 out of 5 (SD 1.0), and the mean overall competence score was 4.0 (0.8). Most (91.7%, *N* = 133/145) sessions received a rating of ≥ 3 (corresponding to at least a satisfactory score) for overall adherence and overall competence (96.6%, *N* = 140/145).

## 4 DISCUSSION

We adapted PATH as a treatment for depression for delivery on a large scale within standard NHS services for people with mild and moderate dementia, and further adapted this for remote delivery in response to the COVID‐19 pandemic. The neutral finding on the primary outcome contrasts with Kiosses et al.,^10^ who found PATH statistically significantly reduced depression severity. However, their's was a single‐center trial, therapy was administered by research staff therapists, outcomes were at 3 months or before and not collected beyond the course of therapy, participants had less severe cognitive impairment (MMSE > 17), not all had a dementia diagnosis and the trial was smaller, with 78 participants. By contrast, all PATHFINDER participants had dementia, and their overall degree of cognitive impairment was more severe. Because our trial participants had all progressed to the point where they had been diagnosed with AD or mixed Alzheimer's and vascular dementia, it can be assumed that significant neurodegeneration had occurred in pathways subserving abilities to understand, recall, and apply mood regulation strategies and to engage in self‐directed, sustained application of these to drive behavior and mood change. Therapy was delivered by a range of multidisciplinary healthcare professionals within routine clinical settings, rather than research team therapists, as PATHFINDER was a pragmatic trial to investigate how adapted PATH would perform if it were delivered at scale within the NHS by existing staff already working in services for people with dementia. Additionally, the primary outcome was at 6 months (i.e., 3 months post‐intervention), rather than immediately post‐intervention. Although a small numerical benefit was seen in our study immediately after therapy completion, this was not maintained at 6 months. It is plausible that participants were unable to retain and later apply strategies taught in therapy sessions, despite the support of their caregivers, so that depression symptoms were only ameliorated during the period of the therapy course. Our finding, that the severity of cognitive impairment did not have an effect on the outcome of adapted PATH treatment, may indicate that even participants who were only mildly affected by dementia (sMMSE > 20) were unable to retain and later apply PATH strategies so that the small improvements seen at 3 months were not maintained at later assessment points.

Other large‐scale, pragmatic studies of pharmacological and psychosocial interventions delivered to people with dementia with their caregivers have also failed to show long‐term benefit in reducing depression severity and improving well‐being^6^, ^22^, ^23^ and this raises the possibility of specific differences in the neurobiological basis of depression in the context of a progressive neurodegenerative disease that impact adversely on response to drug and non‐drug treatments.^24^ However, adapted PATH may not have been entirely without benefit and, although these were secondary outcomes in a neutral trial and thus chance is a plausible explanation,^25^, ^26^ small improvements with treatment were seen at 3 months on both the CSDD and EQ‐5D‐5L. This suggests that psychological therapy for people with dementia and depression may have to be delivered for longer periods, or continuously, until low mood has improved, and this understanding should inform the design of future trials. Although trials of antidepressant medication for depression and dementia have generally indicated no advantage over placebo^5^ (although it is important to recognize that participants in all allocated treatment arms showed overall improvement in symptoms), no trials have yet examined the efficacy of antidepressant combined with psychological interventions. Such combination therapy is generally accepted to be more effective than antidepressant or psychological intervention alone in people without dementia who are depressed,^27^, ^28^ and the severity and apparent treatment resistance of depression associated with dementia would justify exploration of this in future studies.

The PATHFINDER trial and participating patients, families, and the medical and social care services that supported them were all affected by COVID‐19 and measures that were introduced to protect vulnerable people with dementia from infection. In response, trial procedures were modified so that the adapted PATH intervention and collection of outcomes data could be conducted by telephone or videoconferencing. Although this was generally well received by participants and caregivers and took place at a time when many routine clinical services within the NHS were being delivered by such remote means, it could be argued that the consequent lack of face‐to‐face contact with therapists represented a dilution of therapeutic content and effect. However, in (admittedly, underpowered) sensitivity analyses, we did not find that the mode of delivery had a significant impact on the outcome of the adapted PATH intervention. The isolation and loneliness experienced by people with dementia during COVID‐19 might have been expected to enhance the potential beneficial effects of contact with therapists, even virtually, at a time when both family and non‐urgent health or social care visits were suspended.

Strengths of the study include the sample size, representativeness of participants from a large number of NHS community services for people with dementia, and the good levels of demonstrated adherence to and competence in delivery of the manualized intervention achieved by therapists recruited from participants’ usual clinical teams. Had adapted PATH shown clinical effectiveness, it could have been quickly adopted within current NHS services with only small additional training and supervision needed for clinicians.

Study limitations include our choice of available multidisciplinary staff (nurses, clinical psychologists, assistant psychologists, occupational therapists), rather than dedicated trained therapists, for adapted PATH delivery. Failure to recruit a participant population that was representative of the UK's ethnic diversity was disappointing, despite our inclusion of study sites that included areas that had diverse communities. This is a particular problem for trials involving people with dementia, and future studies should take specific steps to recruit more representative participants.^29^ The restrictions imposed by the COVID‐19 pandemic and its potential effect on recovery from depression in people with dementia are further limitations. The results of the trial indicate that eight therapy sessions over 12 weeks, supported by two later booster sessions, did not produce a persistent improvement in rating of low mood. It may be that the adapted PATH intervention was insufficiently intensive and prolonged to have demonstrable efficacy. Although we adapted PATH for people with moderate and severe dementia in collaboration with the research team who originally developed PATH^11^ and the training manual and workshops for PATHFINDER therapists were co‐developed with members of the Cornell group, supervision of adapted PATH delivery and quality assurance of the intervention were managed within the PATHFINDER trial team. Further, although Dr Kiosses personally delivered adapted PATH training to the first cohort of study therapists and supervisors, he did not train therapists and supervisors who subsequently joined the trial. Therefore, it could be argued that this was different from the single‐center, single supervisor approach taken in the original PATH study, which may have influenced the results. Finally, trials of psychological interventions are inevitably more vulnerable to bias than those of medications where blinding of treatment allocation is straightforward. While we took all possible steps to maintain blinding of outcomes assessors, participating patients and their caregivers were not blinded.

Depression in people with dementia is common, frequently severe and associated with reduced quality of life and functioning and increased institutionalization. We chose eight sessions for the trial for reasons of cost and potential scalability and because England's National Institute for Health and Social Care Excellence guideline for treatment and management of depression in adults^30^ recommends eight‐session psychological interventions for depression, though for more severe depression and depression comorbid with other problems, at least 16 sessions are recommended. Although adapted PATH did not show efficacy at the trial's 6‐month primary outcome point, small numerical benefits in mood and quality of life close to the end of the eight sessions of therapy suggest that future trials should examine longer treatment periods as well as potential benefits of combining adapted PATH with antidepressants, along with other components of comprehensive dementia care management.

## CONFLICT OF INTEREST STATEMENT

None of the authors have conflicts of interest that relate to the study reported in this paper. Author disclosures are available in the Supporting Information.

## CONSENT STATEMENT

All participants provided informed consent unless they did not have capacity to do so in which case their relevant representative provided informed consent on their behalf for participation.

## Supporting information

PATH Training Manual

PATH Adherence Rating Scale

Trial protocol

Statistical analysis plan

Supporting Information

Supporting Information

Supporting Information
