# Supplementary material for: Adapted problem adaptation therapy for depression in mild to moderate Alzheimer's disease dementia: A randomized controlled trial
Source: Alzheimers Dement. 2024 Mar 13;20(4):2990–9. doi: 10.1002/alz.13766 (PMC11032547; doi:10.1002/alz.13766)
Supplement: Supplementary file 1 — PATH Training Manual [file ALZ-20-2990-s005.pdf]

THERAPIST MANUAL

Full title: PROBLEM ADAPTATION THERAPY (PATH) FOR PEOPLE WITH MILD TO MODERATE DEMENTIA AND DEPRESSION

Short Title: PATH FOR THE NHS

Adapted from: PROBLEM ADAPTATION THERAPY (PATH): A GUIDE TO A HOME-DELIVERED INTERVENTION FOR ELDERS WITH MAJOR DEPRESSION AND COGNITIVE IMPAIRMENT

Dimitris N. Kiosses, Rebecca L. Gould, Lisa Ravdin, Liz Cort, George S. Alexopoulos, Philip Wilkinson & Robert Howard

July 2019

Table of Contents

Press 'ctrl' at the same time as clicking on a heading to follow the link to that section.

Acknowledgements 4

A guide to how to use this manual 5

Chapter 1: Evidence of treatment for depression in people with mild to moderate dementia 6

1.1 Evidence for treatment of depression in people with mild to moderate dementia 6

1.2 Needs and preferences of people with mild to moderate dementia and depression with respect to psychological interventions 6

Chapter 2: Problem Adaptation Therapy 8

2.1 Summary of Problem Adaptation Therapy 8

2.1.1 What is Problem Adaptation Therapy? 8

2.1.2 Why was a simplified problem solving approach chosen? 8

2.1.3 How does PATH help people with cognitive impairment to manage their emotions? 9

2.1.4 How does PATH help facilitate improved emotion regulation? 9

2.2 Problem Solving Approach 12

2.3. Tools to support memory, attention and planning in PATH 12

2.4 Involvement of caregivers in PATH 13

2.4.1 A note on caregiver involvement in PATH 13

2.4.2 Assessment 13

2.4.3 Involvement in PATH 14

2.4.4 Address tension between the person with dementia and their caregiver 14

2.5 Therapist stance in PATH 15

2.6 Case examples 16

Chapter 3: The modified PATH intervention 17

3.1 Brief overview of the PATHFINDER study 17

3.2 Structure of the modified PATH intervention 17

3.3 Therapist supervision 18

3.4 Key points to bear in mind when delivering PATH 18

3.4.1 Stay focused on emotions 19

3.4.2 Adapt how you engage the person with dementia 19

3.4.3 Stay focused on the person with dementia 19

3.4.4 Set an appropriate pace for the sessions 19

3.4.5 Use behavioural activation techniques to help people with dementia engage in pleasurable activities 20

3.4.6 Ensure adequate discussion of home practice 21

3.5 Engaging people with dementia in psychological therapy 21

Chapter 4: Troubleshooting 22

4.1 Common obstacles 22

4.1.1 Difficulties with organising the list of problems and/or narrowing complaints to a specific problem 22

4.1.2 Difficulties implementing emotion regulation strategies 22

4.2 Common pitfalls 22

Chapter 5: Session outlines, worksheets and handouts 26

5.1 Session outlines, worksheets and handouts 26

5.1.1 Session 1 - Assessment 28

5.1.2 Session 2 - Things that trigger negative and positive emotions 42

5.1.3 Sessions 3-7 - Problem solving 55

5.1.4 Session 8 - Review 74

5.1.5 Top-up sessions 1 & 2 - Recap and review 84

Chapter 6: References 100

Chapter 7: Appendices 102

Appendix 1: Themes from qualitative interviews and focus groups in the PATHFINDER study. 103

Appendix 2: Tools to support memory, attention and planning 110

Appendix 3: Safeguarding guidelines 113

Appendix 4: Case examples 114

Appendix 5: Issues to discuss in therapist supervision 121

Appendix 6: PATH cheat sheet - a reminder of the key principles of PATH 122

Appendix 7: Examples of problems faced by people with mild to moderate depression and dementia 125

Appendix 8: Suicidal ideation guidelines 127

Appendix 9: PATH conceptualisation 129

Appendix 10: An example of a personalised treatment summary for a person with milder dementia (version A). 130

Appendix 11: An example of a personalised treatment summary for a person with more moderate dementia (version B) 132

Acknowledgements

To be completed after the study has ended. It will include an acknowledgement to the PATH developers, manual development group (including names), PATHFINDER co-applicants and collaborators, NIHR HTA, PPI representatives, participants in interviews/focus groups, and participants, therapists and supervisors in the RCT. It will also include the NIHR HTA disclaimer.

[Return to Table of Contents]

A guide to how to use this manual

This manual has been adapted from Problem Adaption Therapy, as developed and used by Dimitris Kiosses and George Alexopoulos at the Weill-Cornell Advanced Center for Interventions and Services Research. It has been adapted to take into consideration the needs and preferences of people with mild to moderate dementia and depression who are receiving care in the NHS, in collaboration with Dimitris Kiosses and his colleagues.

Adaptation of this manual has been informed by qualitative data generated from interviews with people with dementia and depression and their caregivers, focus groups with healthcare professionals and discussions with experts in the field. This manual has been developed by a multi-professional team, including Patient and Public Involvement representatives. **It has been designed for use by practitioners within Memory Services and Mental Health Services for Older People within the NHS who have completed a 1-day training course in the approach**. It is assumed that practitioners (hereafter called therapists) will have some knowledge of dementia and depression.

There are seven sections in this manual, as outlined below (press 'ctrl' at the same time as clicking on a heading to follow the link to that section):

Chapter 1: Evidence for treatment of depression in people with mild to moderate dementia
The evidence for treatment of depression in people with mild to moderate dementia is briefly reviewed, along with the rationale for offering Problem Adaption Therapy (PATH). It also highlights the needs and preferences of people with mild to moderate dementia and depression with respect to psychological interventions, as reported by service users, caregivers and healthcare professionals in qualitative interviews.

Chapter 2: Problem Adaptation Therapy
This section provides a summary of PATH, and describes the stages involved, as well as the tools that can be used to support memory, attention and planning and how caregivers can be involved in the intervention.

Chapter 3: The modified PATH intervention
A brief overview of the PATHFINDER study is given. This section also describes the structure of the modified PATH intervention and key points to bear in mind when delivering the intervention.

Chapter 4: Troubleshooting
Common problems and pitfalls in delivering the modified PATH intervention are outlined.

Chapter 5: Session outlines, worksheets and handouts
This section describes each of the sessions in the modified PATH intervention, and provides worksheets and home practice sheets that accompany each of the sessions.

Chapter 6: References
This section lists the references.

Chapter 7: Appendices
Additional information relevant to the modified PATH intervention is provided.

[Return to Table of Contents]

Chapter 1: Evidence of treatment for depression in people with mild to moderate dementia

1.1 Evidence for treatment of depression in people with mild to moderate dementia

Depression and depressive symptoms are common in people with dementia. Around 50% of people with Alzheimer's disease have clinically significant depressive symptoms (Lyketsos et al., 1997; Starkstein et al., 2005) and 20% meet criteria for major depressive disorder (Enache et al., 2011). Depression in dementia is important because it reduces quality of life (Lyketsos et al., 1997), exacerbates cognitive and functional impairments (Greenwald et al., 1989), and increases mortality (Burns et al., 1990), risk of transition to a residential care or nursing home (Stern et al., 1997) and caregiver burden (Gonzalez-Salvador et al., 1999).

Antidepressant medication is often prescribed to people with dementia (Kessing et al., 2007). However, previous randomised controlled trials have reported no superiority of antidepressants over placebo (Banerjee et al., 2011; Rosenberg et al., 2010). Two meta-analyses have also found **no significant benefits of antidepressants over placebo in people with dementia and depression** (Thompson et al., 2007; Orgeta et al., 2017). The poor response of depression in dementia to antidepressants seen in the trials does not seem to be attributable to the severity of depression in participants, the type of people recruited or to reduced adherence with trial medication, and it has been suggested that the neurobiology of depression in dementia might be different from that of depression in people without dementia.

Conventional psychological therapies have also been generally disappointing in this situation. A Cochrane review of psychological therapies, including cognitive behavioural therapy, for depression in dementia identified six randomised controlled trials (Orgeta et al., 2015). This reported a **small, significant effect of psychological therapies, suggesting only very modest benefit at best**. Variation between the individual Cochrane reviewed studies, in terms of both the modality of psychological intervention and treatment duration, makes it very difficult to draw conclusions regarding which therapies should be recommended for depression in dementia.

Problem solving therapy has been widely used with older people with depression, and a systematic review and meta-analysis of six studies reported some improvement in depression scores (Kirkham et al., 2016). Problem Adaption Therapy is based on problem-solving therapy and was largely developed for use with depressed and mildly cognitively impaired older people (Kiosses et al., 2010, 2011, 2015; Arean et al., 2010; Alexopoulos et al., 2011). Preliminary results demonstrated that Problem Adaption Therapy reduces depression and disability in older people with major depression and cognitive impairment, ranging from mild cognitive impairment to mild dementia, compared to home-delivered Supportive Psychotherapy (Kiosses et al., 2015). It is therefore possible that, if Problem Adaption Therapy is sufficiently adapted to the needs and preferences of people with mild to moderate dementia and depression, then this group could similarly benefit from this intervention.

1.2 Needs and preferences of people with mild to moderate dementia and depression with respect to psychological interventions

We conducted a series of interviews and focus groups with people with mild to moderate dementia, caregivers and healthcare professionals in order to determine the needs and preferences of people with mild to moderate dementia and depression with respect to psychological interventions. A brief summary of our findings is shown in Figure 1, with a full summary being provided in Appendix 1.

[Return to Table of Contents]

Figure 1: The main themes and subthemes derived from qualitative interviews with people with dementia and their caregivers, and focus groups with healthcare professionals. The thick arrows indicate subthemes within a theme, and the thin arrows illustrate major links between subthemes.

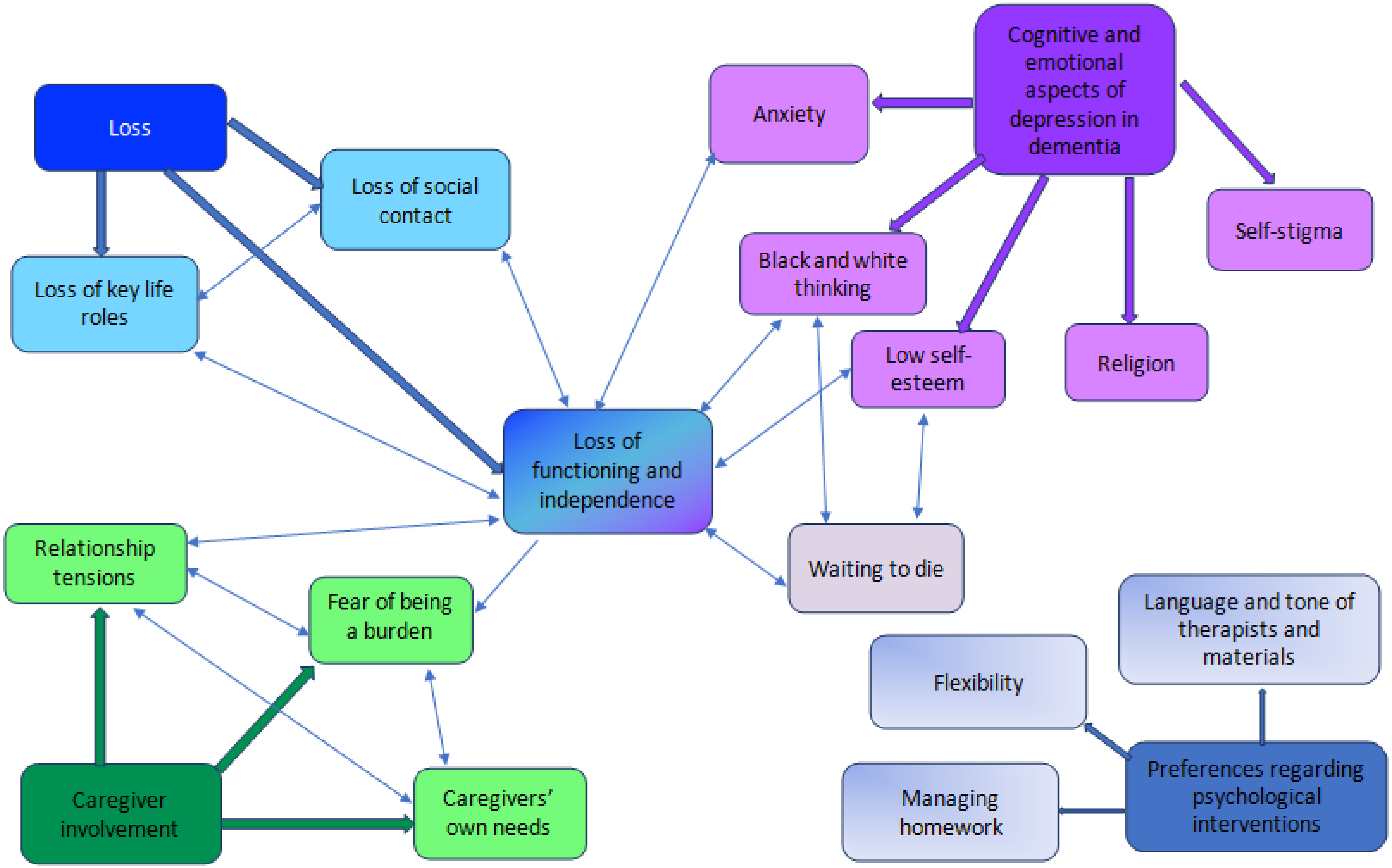

Chapter 2: Problem Adaptation Therapy

2.1 Summary of Problem Adaptation Therapy

2.1.1 What is Problem Adaptation Therapy?

Problem Adaptation Therapy or PATH is a home-delivered non-pharmacological intervention for older people with major depression and cognitive impairment. It focuses on the person with dementia's "ecosystem" or the situation within which they are living, which includes the person with dementia, the caregiver, and the home-environment, in order to facilitate problem-solving and adapting to life with cognitive impairment. **The goals of PATH are to reduce depression and disability in people with dementia through improved emotion regulation or management of emotions** (Gross, 1998, 2014).

PATH aims to reduce negative emotions associated with depression, including depressed mood, hopelessness, helplessness, guilt, anxiety and anhedonia, as well as promoting positive emotions. The tools to improve emotion regulation are: a) a hands-on problem solving approach, simplified from Problem Solving Therapy (D'Zurilla et al., 1999); b) compensatory strategies and environmental adaptations or changes to the environment to bypass cognitive, functional and behavioural limitations; c) careful integration of caregiver involvement; and d) increasing engagement in pleasurable activities (Teri et al., 1997). Using these tools, PATH can create an environment that decreases everyday stressors, empowers people with dementia, instills hope and promotes improved emotion regulation (Fig. 2).

Figure 2: An outline of PATH.

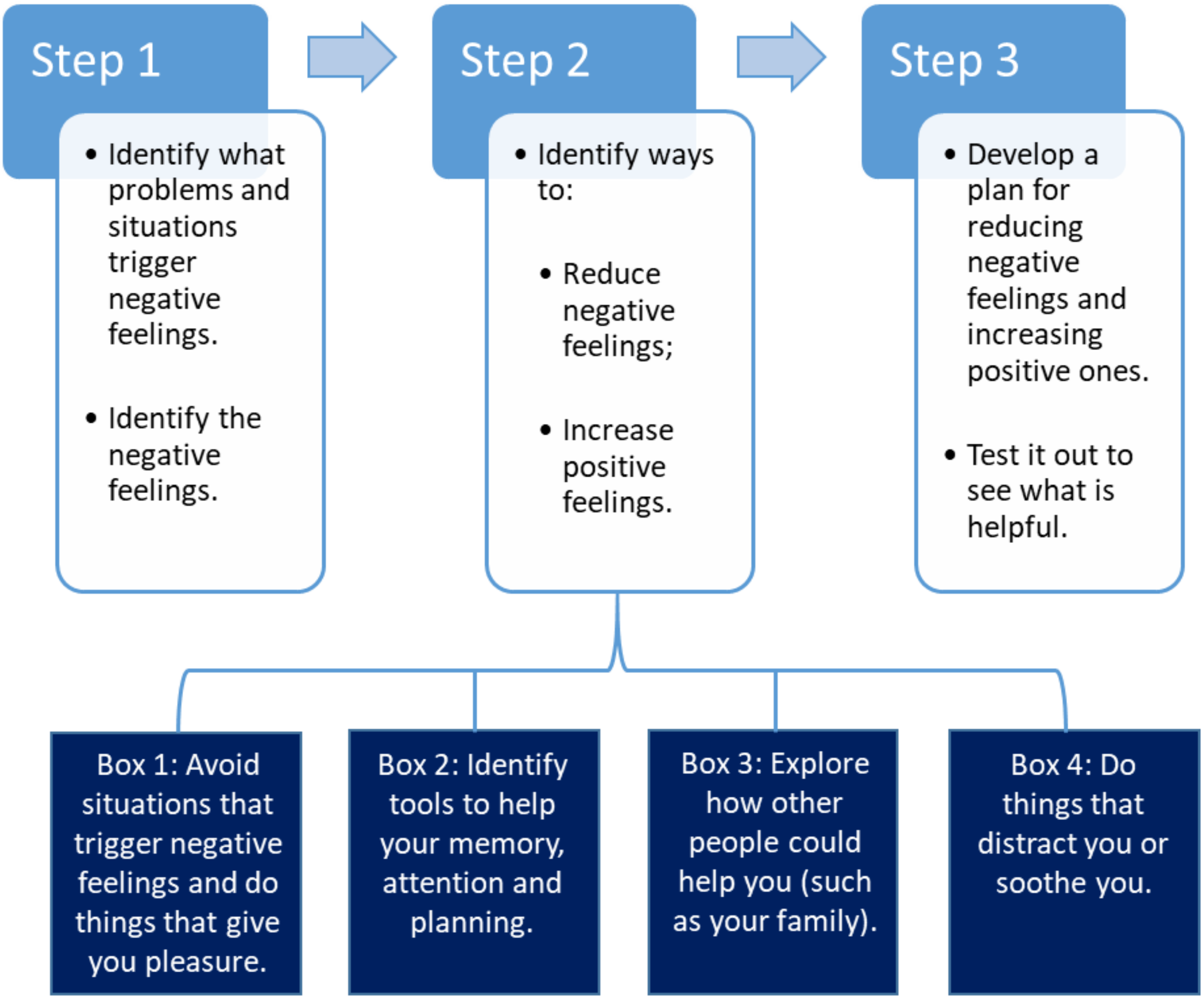

2.1.2 Why was a simplified problem solving approach chosen?

A simplified Problem Solving Therapy approach has been chosen because: a) Problem Solving Therapy has been found to be effective in cognitively intact depressed older people and those with mild executive dysfunction

(Alexopoulos et al, 2003); b) Problem Solving Therapy creates a forum within which patients and therapists can collaboratively set treatment goals, and it provides the structure and the process for patient problem solving pertinent to decreasing depressive symptoms and functional limitations; c) problem-solving strategies can be appropriately modified in order to help remedy behavioural limitations resulting from cognitive impairment (Cicerone et al., 2000); and d) behavioural interventions focusing on caregiver problem-solving can decrease depression in people with dementia (Teri et al., 1997). **The problem-solving approach used in PATH does not follow the formally structured approach of Problem Solving Therapy, but uses each stage of Problem Solving Therapy when appropriate**. Because finding solutions to problems may not necessarily reduce depression, **the focus of PATH's problem solving approach is to reduce negative emotions and promote positive emotions that are associated with the specific problem**. To achieve improved emotion regulation (Gross, 1998; Gross, 2014), the PATH therapist helps the person with dementia to find the best possible solution, but also helps them to adapt to the problem (i.e. to best live their life with the problem) if the problem cannot be solved.

2.1.3 How does PATH help people with cognitive impairment to manage their emotions?

Even though a problem solving approach may be effective in cognitively intact people or people with mild executive dysfunction, it would have limitations with people with more advanced cognitive impairment. Specifically, cognitive impairment would be anticipated to interfere with the learning, administration, and management of problem-solving skills, as well as the effective selection and implementation of the solution. Further, cognitively impaired people may need additional help to bypass the behavioural limitations caused by their cognitive deficits. Therefore, in addition to the problem solving approach, **PATH utilises three strategies to improve emotion regulation**:

1. *Tools to support memory, attention and planning:* PATH encourages the use of tools to support memory, attention and planning in the person's environment, which aim to overcome cognitive, functional and behavioural limitations and create a more supportive environment. This environment may facilitate improved emotion regulation by reducing environmental stress (Gross, 1998; Gross, 2014). The use of similar environmental tools has been shown to reduce psychopathology and disability (Velligan et al., 2000a, 2000b) in non-depressed patient populations with pronounced cognitive impairment (e.g. people with chronic schizophrenia, traumatic brain injury and intellectual disability). The tools that have been incorporated into PATH have been modified appropriately for depressed older people with cognitive impairment. Tools to support memory, attention and planning *do not intend to repair or recover* brain dysfunction, but rather to **compensate for cognitive functions that are lost** and to help the person with dementia **use their remaining cognitive functions** to overcome behavioural limitations due to depression, cognitive impairment and disability.

2. *Delivery of the first two sessions at home:* Older people who are depressed and cognitively impaired may spend much of their time at home. It is, therefore, important that the **first two PATH sessions are delivered in the home environment**. PATH therapists will then have the ability to evaluate situations occurring in the person's own environment and to use what is available in that environment to help them. Helping people with dementia in their own homes has further practical value because they typically experience most of their difficulties within their homes.

3. *Caregivers as facilitators:* Available and willing family or other caregivers (i.e. significant other family and friends or professionals) are invited to participate to help reduce negative emotions and promote positive emotions experienced by the person with dementia. The caregiver may facilitate the problem-solving process, the use of tools to support memory, attention and planning, and the person with dementia's engagement in pleasurable activities, when the person's cognitive deficits prevent them from performing these tasks alone. The participation of caregivers has been influenced by the Behavioural Treatment for Depression in Dementia programme (Terri et al., 1997), which has been successfully used with more severely cognitively impaired depressed older people. Appropriately introduced, PATH is well accepted by caregivers. Most perceive the PATH work as reassuring and experience their role as empowering them to solve the patient's problems. However, there is a **potential for tension to arise** if a caregiver acts as a facilitator for a person with dementia who is more cognitively able. Therefore, it is important to consider the person with dementia's preferences with respect to the involvement of their caregiver. If there are differences in opinion between them then this could be identified as an issue to work on in PATH.

2.1.4 How does PATH help facilitate improved emotion regulation?

PATH follows the process model of emotion regulation (Gross, 1998; Gross, 2014). According to this model, **emotions such as those associated with depression can be regulated or managed in five broad steps**: 1) selecting the situations a person is exposed to; 2) changing the situations that may elicit distressing emotions; 3) shifting attention within a situation; 4) changing how one thinks about a situation; and 5) making direct efforts to alter

emotional responses (or to manage emotions). PATH utilises the strategies outlined earlier (i.e. problem solving approach, tools to support memory, attention and planning, and caregiver involvement) to achieve improved emotion regulation through the five broad steps of the process model of emotion regulation (Table 1).

Table 1: Examples of ways to regulate emotions.

**Ways to regulate emotions**	**Examples**
Select the best situations to expose the person to	1. Identify situations and activities that trigger negative emotions associated with depression and avoid these situations.
	2. Identify and promote situations and activities that trigger positive emotions.
Change the situation the person is in	1. Identify tools to support memory, attention and planning to overcome functional limitations that trigger a strong negative emotional response in the person with dementia.
	2. The caregiver modifies these emotionally charged situations accordingly.
Shift or redirect attention	1. Use tools to support memory, attention and planning to overcome functional limitations and redirect a person's attention to positive aspects of life (e.g. through distraction).
Change perspectives	1. Help the person with dementia and caregiver (if necessary) to develop a realistically hopeful approach to functional and cognitive limitations (e.g. cognitive impairment doesn't necessarily prevent them from enjoying life; focus on what they can do rather than what they can't do; tools to support memory and attention that may reduce functional limitations).
Manage emotions	1. Help the person with dementia and caregiver (if necessary) to use skills for managing emotions during emotionally charged situations (e.g. using techniques to reduce escalation of tension between the person with dementia and caregiver).

Techniques that may be particularly helpful for regulating specific emotions in people with dementia and depression are shown in Table 2.

Table 2: Specific techniques for regulating specific emotions in people with dementia and depression.

**Emotion**	**Technique(s)**	**Rationale / Suggested approaches**
Sad or depressed	• Select the best situations by establishing a structured daily routine and scheduling pleasant activities within it (see "Activities that I enjoy doing" and "Calendar of Activities" worksheets)	• "When we feel sad or down we often feel like not getting out of bed, staying at home or isolating ourselves from others. However, this is not helpful for two reasons. One, because it means that we have more time to think about the things that are upsetting us. And two, because we have fewer opportunities to experience pleasure. Therefore, what we need to do is help you to start doing more things that give you pleasure in order to help you feel better."<br>• "Sometimes we think that if we engage in a particular activity, we will not enjoy it. As a result, our motivation to engage in activities is reduced. To overcome this hurdle, we need to engage in activities even if we think we won't enjoy them. Don't wait to feel motivated to engage in activities; engage in activities to increase your motivation."
Bored or lonely	• Select the best situations by establishing a structured daily routine and scheduling pleasant activities within it (see "Activities that I enjoy doing" and "Calendar of Activities" worksheets)	• "Not doing much each day can make you feel bored or lonely and even lower in mood. What we need to do is create more structure in your daily life in order to keep you busy and distract you from feeling lonely or bored."
Lacking motivation or not feeling like doing anything	• Select the best situations by establishing a structured daily routine and scheduling pleasant activities within it (see "Activities that I enjoy doing" and "Calendar of Activities" worksheets)	• "Feeling down affects the way we think. We tend to think in a negative way when we're feeling down. For example, we may think that we need to feel like doing something before we can do it. In fact, the opposite is true. We need to do start doing something before we can feel like doing it."

**Emotion**	**Technique(s)**	**Rationale / Suggested approaches**
Feeling hopeless or useless (e.g. due to thinking "I can't do anything anymore")	• Change perspectives by exploring what the person with dementia can still do and shift or redirect attention to this (e.g. "I can still do...")<br>• Change the situation by exploring new ways of doing activities using tools to support cognition and help from others<br>• Shift or redirect attention to something else (see "List of strategies for distracting or soothing myself" worksheet)	• "It's easy to focus on the negatives such as all the things we can no longer do when we're feeling down. However, this can make us feel even lower in mood. Instead, it is more helpful to focus on the positives and what we still can do. It is also helpful to look at what tools we can use or what support we can get from others to help us do things."<br>• "When we feel useless or bad about ourselves, we may ask ourselves "How is this helping me?", "What do I gain by being hard on myself?" We might reflect that it will probably make us feel worse, it will not help us experience pleasure in our day-to-day life, and it will affect our relationships. Saying to ourselves "Let me try to distract myself from these negative feelings by focusing on something positive, like engaging in a pleasurable or rewarding activity" can be helpful."
Anxious, frustrated or angry	• Shift or redirect attention to something else (see "List of strategies for distracting or soothing myself" worksheet)<br>• Manage emotions by soothing oneself (see "List of strategies for distracting or soothing myself" worksheet)	• "When we're feeling anxious/frustrated/ angry, we tend to focus on the things that are making us feel this way, which can make us feel even worse. What we need to do in these situations is do something that either distracts us or that calms us down."
Rumination	• Shift or redirect attention to positive aspects of the situation (e.g. shift attention to positive memories of one's partner, rather than ruminating about negative memories)<br>• Shift or redirect attention to something else (see "List of strategies for distracting or soothing myself" worksheet)	• "When we keep ruminating or dwelling on things that have happened in the past, this can make us feel upset and even lower in mood. Instead, it is more helpful to shift our attention to more positive aspects of the situation."<br>• "It is important to try to reduce rumination or dwelling on things as soon as it starts; so, whenever we notice that we have started ruminating, it's a great idea to shift our attention away from it. It's helpful to have created a list of distraction strategies so we will be ready to distract ourselves when we need to."
Tension with caregiver	• Shift or redirect attention to positive aspects of the patient/caregiver relationship<br>• Change perspectives by encouraging them to look at the situation from the other person's perspective (if possible)	• "When we're feeling upset with another person, it's easy to focus on the negatives in that relationship. However, this can make us feel even lower in mood. Instead, it is more helpful to focus on the positives in that relationship. It is also helpful for us to try to look at the situation from the other person's viewpoint."<br>• "It's always helpful to set aside our emotions – whenever we can – and think about the usefulness of having tension with our spouse/children/friend. How is this helping us? And if it's not helping us, how can we reduce it?"
All	• Explore the pros and cons of the emotion in order to see whether it is helping the person to feel better<br>• If it is not, then use self-talk to shift or redirect attention to something else	• "It's always helpful to examine the usefulness of our feelings and the effect that they have on us. If we realise that our feelings are not helpful to us, we should try to find ways to modify them. For example, when we're feeling upset, it is important to think about whether what we're feeling is helping us to feel better in our mood. If it is not, then reminding ourselves of this can be useful. For example, it can be useful to tell ourselves "This feeling is not helping me feel better...let me do X instead".

2.2 Problem Solving Approach

As noted above, PATH uses a simplified problem-solving approach to regulate emotions. The **PATH therapist uses each stage of problem solving when appropriate and necessary** (Table 3). For example, a therapist and the person with dementia (and the caregiver, if necessary) may find the best solution to a problem without applying all the sub-stages of the second stage ('Investigate possible solutions').

Table 3: Problem Solving Stages

**Stage**	**Sub-stage**	**Description**
1. Explore the problem	Identify the problem	List and select problems
	Define the problem	Examine the details of the problem (where, when, with whom and under what circumstances the problem occurs) and the negative emotions associated with it
2. Investigate possible solutions	Brainstorm solutions	Generate possible solutions to the problem
	Evaluate solutions	Look at the pros and cons of each solution
	Choose which solution(s) to test out	Select the best solution(s) to test out
	Make a plan for testing out the solution(s)	Specify exactly how the solution can be tested out
3. Test out the solution	Prepare to put the plan into practice	Make all the necessary preparations for the plan (e.g. use tools to support memory, attention and planning, etc)
	Put the plan into practice	Put the plan into practice and review whether it helped

2.3. Tools to support memory, attention and planning in PATH

Tools for supporting memory, attention and planning are intended to help people with dementia bypass their behavioural limitations, reduce their negative emotions and promote positive emotions, with or without the assistance of family or other caregivers. Any tool that changes the person's environment does not improve the person's cognitive impairment *per se*, but reduces the negative consequences of the cognitive impairment on day-to-day functioning. In turn, this may reduce or change the person's emotional response. These tools resemble giving a walking stick to a person who has difficulty in walking. It does not fix the underlying orthopaedic problem, but allows the person to walk. Table 4 presents tools for supporting memory, attention and planning that can be utilised in PATH. Further information about these tools is provided in Appendix 2.

Table 4: Types of tools or strategies for supporting memory, attention and planning in PATH.

**Domain**	**Tool / strategy**	**Examples**
Memory	Memory aid	*Visual aids:* Calendars, clocks, coloured tags, daily checklists, diaries, magnetic notepads, markers, medication kit, notebook, pictures, signs (e.g. black and white signs with regular font, coloured signs, black and white signs with large font), sticky notepapers and reminders, weekly planner, whiteboard<br>*Auditory aids:* Alarms, alarm clocks, beeping watches, computerised phone calls, customised audiotapes, key-chain recorder, timers, timed pre-recorded messages
Attention	Keep the person's attention on the task at hand	Providing positive social reinforcement (e.g. encouragement, praise, rewards) to keep a person's attention on the task at hand - usually done in conjunction with minimising distractions.
	Minimise distractions	Reducing clutter in the environment, turning off the TV or radio when completing a task, completing a task when one is least likely to be disturbed
Planning	Break a task down into smaller steps	Cooking a meal can be broken down into smaller steps → getting all the ingredients, putting all the ingredients into the pan in the right order and right amount, and setting the timer on the stove
	Specify the preparatory steps for a task	Preparatory steps for cooking a meal → putting all the necessary ingredients in a specific place, having a cookbook specifying the right amount of ingredients and the right order, making the measurements easily accessible to the person with dementia, and having instructions for using the stove timer
	Tools for filing	Folders in which instructions and checklists for completing certain tasks are kept

The **selection** of appropriate tools to support memory, attention and planning **depends on** the person with dementia's **behavioural and functional limitations, cognitive functioning, depression severity, availability of caregivers** to assist with the use of the tools, and the **specifics of the problem**. The same tools can be used in multiple ways to deal with different behavioural limitations. For example, reminders can be utilised to help the person follow steps of a plan aimed at improving organisation (often impaired by executive functioning) or to help the person remember to call friends and thus maintain social relationships (often a result of attention or memory impairment).

In certain cases, a combination of tools may be used simultaneously or sequentially for a specific problem. For example, a checklist, a sign, and a timer may help a person to prepare lunch. The checklist can provide the list of the ingredients. For each step, a sign can ask the person to set the timer for the next step. The sound of the timer will indicate the end of one step and the beginning of the next step. The caregiver may be involved, if necessary.

Tools vary in how 'high tech' they are. Voice alarms, customised audio tapes and timed pre-recorded messages are fairly 'high tech', whereas strategically placed notepads, signs/pictures, calendars/diaries/weekly planners, and whiteboards are less so. It can be tempting to try and introduce the use of 'high tech' tools. However, the cost, learning required and the investment of time means that this may not be practical within the PATHFINDER intervention. **'Low tech' tools should be favoured unless the person with dementia is familiar with 'high tech' ones.**

To determine the extent of the use of tools to support memory, attention and planning in PATH, the therapist needs to make a clinical judgment in the following areas:

1) Does the person with dementia have difficulty in learning new procedures or techniques?
2) Does the person with dementia have the ability to form a plan and follow sequential steps until its completion?
3) Does the person with dementia have the ability to complete the home practice assignments?

The therapist also needs to consider:

i) How the person with dementia's cognitive strengths can be capitalised on when developing plans;
ii) Which tools to support memory, attention and planning are already familiar to the person with dementia;
iii) How these tools can be best used to support the person with dementia to carry out any identified plans;
iv) How caregiver involvement can maximise the usefulness of these tools to the person with dementia.

2.4 Involvement of caregivers in PATH

2.4.1 A note on caregiver involvement in PATH

Involving caregivers in PATH is important for aiding successful delivery of the intervention to those with milder dementia, and is crucial for those with more moderate dementia. However, it can raise challenges for therapists, including that the caregiver may dominate discussions of the modified PATH intervention or use the session to discuss their own issues. It could also potentially exacerbate existing tension within the relationship between the person with dementia and the caregiver, which is discussed in more detail in Section 2.4.4.

It is important that therapists ensure that the focus of the intervention remains on the person with dementia and not the caregiver. It is easy for caregivers to potentially dominate sessions, particularly if a person has a more moderate degree of cognitive impairment. Therefore, having a short discussion (5 mins) with the caregiver after the session has ended can help them to feel listened to, without the focus being taken away from the person with dementia. If a caregiver needs additional support, then signposting or referring the caregiver for their own psychological support should be considered.

2.4.2 Assessment

A careful evaluation of family or other caregiver should be completed before the decision is made to engage them in PATH. An assessment of the caregiver's ability, availability and willingness to help will include finding out about:

1. The caregiver's physical and mental ability to help (i.e. whether they have any physical limitations, cognitive limitations, emotional limitations).
2. The caregiver's availability to help (i.e. whether they are working, have other caring responsibilities).
3. The caregiver's motivation to help. Lack of motivation may interfere with the ability to help the person with dementia deal with everyday problems. A caregiver who is not motivated to help may become easily overwhelmed or

frustrated and create tension that may be counter therapeutic.

If the caregiver is able, available and interested in helping, the therapist should explain the goals of PATH, the treatment methods, the anticipated outcomes and potential inconveniences of treatment. The therapist should explore the caregiver's concerns and answer questions. Establishing a therapeutic alliance with both the person with dementia and the caregiver is likely to be experienced as empowering by both of them.

2.4.3 Involvement in PATH

Caregivers can be involved in all aspects of PATH. They may participate in problem-solving, initiation of pleasurable activities, selection and use of tools to support memory, attention and planning, and provide valuable insight and feedback on treatment progress. Caregivers can provide important information at every stage of problem solving. **Their feedback is important, as people with dementia may not accurately recall all of their problem areas.** Caregivers can help identify a person's problem, elaborate on the specifics of the problem, brainstorm possible solutions, evaluate the pros and cons of each solution, and finally test out the solution. Further, they can provide insight into the reasons for the success or failure of a solution. Tools to support memory, attention and planning are critical in bypassing the person with dementia's behavioural and functional limitations. **Caregivers may offer valuable input in the selection of appropriate tools and in their use within the home environment.** Moreover, feedback from a caregiver on the effectiveness of these tools can be beneficial when adapting tools used in specific situations.

Although not every person with dementia will require help from a caregiver, participation of caregivers will often be necessary. The caregiver's participation will depend on the person with dementia's ability to solve the problems unassisted, their cognitive impairment and areas of cognitive strengths, and the specifics of the problem. The same questions used to determine the extent of the use of tools to support memory, attention and planning in PATH (see above) can be used to determine the extent of the involvement of the caregiver in PATH.

2.4.4 Address tension between the person with dementia and their caregiver

As noted above, involvement of caregivers in the modified PATH intervention can exacerbate existing tension within the relationship between the person with dementia and their caregiver. Careful evaluation of the caregiver's participation is crucial throughout each PATH session. A positive relationship between the care recipient and the caregiver will facilitate the appropriate administration of PATH. However, caregiver participation in treatment may also reveal or exacerbate tension between the person with dementia and the caregiver. **The PATH therapist needs to address the tension when it interferes with the problem-solving process and contributes to the person with dementia's depression**. In these circumstances, PATH's goal is short-term and specific: to reduce the tension sufficiently so that problem-solving approaches are effectively utilised.

The following techniques may be helpful in reducing any tension:

<u>i) Identify and define the tension</u>

It is important for the PATH therapist to identify, explore and evaluate the tension. The therapist should explain that varying degrees of tension exist in most interpersonal relationships and that a concerted effort by both the person with dementia and the caregiver is critical to decrease it. It should be emphasised that the goal is to work collaboratively (with the therapist, the person with dementia, and the caregiver working together) to solve the person with dementia's problems and reduce the tension that interferes with this process. The degree to which the person with dementia is able to work collaboratively with the therapist and caregiver on this issue will be affected by the degree of cognitive impairment. Therefore, caregivers and/or therapists may need to take a more active role in developing a plan to reduce the tension in these instances.

> *"It's very common for tension to be present in any relationship, but particularly when life is throwing challenges at us. It's important that we try and work together as a team to reduce this tension. Would you be willing to work together to explore this tension in more detail?".*

The following questions may be helpful in identifying and defining the tension:

> *"What creates tension between you?"*
> *"When did you first notice it?"*

"How intense are the feelings for you both?"
"Under what circumstances does the tension increase?"
"How do you both react to the tension?"
"Does it ever feel like it gets out of control? What happens then?"
"How have you tried to reduce the tension between you?"
"If we can't find a solution to the problem that you are both satisfied with, what could be helpful ways of reducing the tension?"

PATH therapists need to be alert to any potential safeguarding issues (e.g. disclosure or suspicions about any type of abuse experienced by the person with dementia or their caregiver) and should follow guidelines listed in Appendix 3 if any concerns are identified.

ii) Avoid escalation of the tension

PATH's goal is to create an ecosystem or environment that will promote positive emotions and reduce negative emotions in the person with dementia. **As a result, the PATH therapist needs to discourage escalation of the conflict.** The therapist should concentrate on the positive aspects of the caregiver-patient dyad without minimising the interpersonal tension. Encouraging each person to see the other's perspective and focusing on the specifics of the problem might be beneficial in reducing the tension. However, it should be remembered that being able to see things from the caregiver's perspective might be difficult for a person with more moderate dementia. If necessary, meet separately with the caregiver and person with dementia to discuss the other person's perspective.

"I noticed that it has been hard for both of you to find a solution that you both agree on. Would you be willing to try and see if we can identify some common ground here and see whether additional progress can be made before the next session?"

"In terms of things to try out before the next session, would you be willing to try and think about this problem as if you were the other person? So, Jack, you should try and look at this problem as if you are Peggy, and Peggy, you should try and look at this problem as if you are Jack. See if you can try thinking about possible solutions that will be helpful, not to you, but to the other person. What do you think?"

iii) Evaluate the pros and cons of the tension

The therapist may gain further insight by asking the person with dementia and their caregiver to evaluate the advantages and disadvantages of the tension. By recognising the negative consequences of the tension, the person with dementia and the caregiver may become more motivated to work together to reduce the tension.

"How is this tension helping you? How does this tension affect your ability to deal with problems? What are the advantages of the tension? What are the disadvantages of the tension? If the tension wasn't there, how would things be different?"

iv) Explore strategies for reducing the tension

The therapist may specifically identify tension as a problem that requires a solution. Therefore, the problem-solving stages in PATH can be applied to the problem of tension. Specifically, the therapist, the person with dementia, and the caregiver would explore solutions for reducing the tension, and make a plan for testing out the best possible solution and then putting it into practice. **Working together as a team on finding the solution to the tension may promote further collaboration.**

"Is there anything that can be done to reduce this tension? Is there anything you both could do to make you feel better <to person with dementia> and you feel better <to caregiver>?"

2.5 Therapist stance in PATH

There are a number of principles that therapists need to keep in mind when delivering the modified PATH intervention. The therapist should guide the person with dementia and the caregiver to develop their own strategies as much as possible in order to promote independence (e.g. by validating good ideas). At the same time, however, the therapist needs to take an active role in therapy, and strong suggestions may be needed at times if the person with dementia and their caregiver are struggling to identify possible strategies. That is, the therapist might strongly

encourage adoption of a particular strategy if they think this is going to be beneficial in reducing negative emotions or increasing positive emotions (e.g. “This is what I think might work…”). Finally, flexibility is needed in the delivery of the intervention in order to ensure that the unique and individual needs of the person with dementia and their caregiver are adequately met.

2.6 Case examples

Some examples of how PATH can be applied to people with dementia are shown in Appendix 4.

[Return to Table of Contents]

Chapter 3: The modified PATH intervention

3.1 Brief overview of the PATHFINDER study

The PATHFINDER study has been developed in response to a Commissioned Call by the National Institute for Health Research Health Technology Assessment Programme to develop an intervention based on PATH to treat depression in people with mild and moderate dementia within the NHS. The main aims and objectives of the study are to:

(1) Modify PATH so that it is accessible and acceptable to people with mild and moderate dementia and their caregivers, and can be delivered by existing staff in NHS services (memory services and community mental health teams for older people);
(2) Obtain quantitative estimates of the accessibility, acceptability, credibility and feasibility of the modified PATH intervention;
(3) Use qualitative approaches to explore the intervention's acceptability to people with dementia and their caregivers, as well as therapists delivering the intervention;
(4) Establish the clinical and cost-effectiveness of the modified PATH intervention plus usual multidisciplinary care compared to usual multidisciplinary care alone in a multicentre, single-blind, parallel, 2-arm randomised controlled trial.

In Phase 1 of the study, we conducted a series of interviews and focus groups with people with mild to moderate dementia, caregivers and healthcare professionals in order to determine the needs and preferences of people with mild to moderate dementia and depression with respect to psychological interventions. We then developed a modified version of PATH, based on feedback from the interviews and focus groups and discussions with experts in the field.

In Phase 2 of the study, we will assess how helpful the modified PATH intervention is for reducing depression in people with mild to moderate dementia and depression in comparison to usual multidisciplinary care. Recruitment to the trial will start in 2019 and will continue for 24 months.

3.2 Structure of the modified PATH intervention

The modified PATH intervention consists of up to 8 sessions of PATH, adapted for people with mild to moderate dementia and depression, followed by two top-up sessions at 6 and 9 months. Sessions are face-to-face and are delivered over 12 weeks, with each session lasting up to 1 hour.

The intervention will either be delivered at the clinic (with the exception of the first 2 sessions being delivered in the person with dementia's home) or in the person with dementia's home (if they are unable to travel to the clinic). Sessions 1 and 2 are delivered in the home setting as this will enable the therapist to understand the home environment, where the person with dementia spends most of their time and where most problems are typically encountered.

An outline of each of the sessions is presented in Table 5. Home practice is scheduled at the end of each session and reviewed in the next session. In the first session, the home practice focuses on asking the person with dementia and/or caregiver to write down any comments and questions about PATH for the next session. In addition, the person with dementia (and caregiver, if necessary) is asked to complete the 'Activities that I enjoy doing' worksheet, while the caregiver is asked to complete the 'Caregiver Information Form'. Home practice in the second session comprises either asking the person with dementia to engage in at least two pleasurable activities before the next session or completing the 'Activities that I enjoy doing' worksheet (if this has not already been completed for home practice). In the remaining sessions, home practice focuses on putting plans for reducing negative emotions and increasing positive emotions into practice, as well as asking the person with dementia to engage in at least two pleasurable activities before the next session.

Table 5: A summary of the outline of each of the sessions in the modified PATH intervention.

Session	Main focus of session	Goals of the session
1	Assessment	1) Introduce PATH to the person with dementia and the caregiver.<br>2) Evaluate depression, cognitive strengths and difficulties (i.e. what they can and cannot do), and physical and functional limitations in the person with dementia.<br>3) Briefly summarise the session and ask for feedback.<br>4) Set the home practice.
2	Things that trigger negative and positive emotions	1) Assess risk.<br>2) Briefly review previous session and home practice.<br>3) Identify problems/concerns/situations that trigger negative emotions.<br>4) Briefly summarise the session and ask for feedback.<br>5) Set the home practice.
3-7	Techniques to reduce negative emotions and increase positive ones	1) Assess risk.<br>2) Briefly review previous session and home practice.<br>3) Across the 5 sessions, identify a specific problem and the negative emotions associated with it, identify ways to reduce negative emotions and increase positive emotions, and create a plan for this (including which tools to support memory, attention and planning to use and how to involve the caregiver, if necessary).<br>4) Briefly summarise the session and ask for feedback.<br>5) Set home practice.
8	Review	1) Assess risk.<br>2) Briefly review previous session and home practice.<br>3) Review which problems and negative emotions were addressed, and what emotion regulation strategies were used to reduce negative emotions and increase positive ones (including what caregiver assistance was needed and what tools to support memory, attention and planning were utilised).<br>4) Briefly summarise the session and ask for feedback.<br>5) Discuss next booster session.
Top-up at 6 months (top-up session 1) & 9 months (top-up session 2)	Recap and review	1) Assess risk.<br>2) Briefly review previous session.<br>3) Recap on PATH.<br>4) Review and revise (if necessary) the personalised written summary developed in the previous session.<br>5) Briefly summarise the session and ask for feedback.<br>6) Discuss next booster session (if top-up session 1) or ending the sessions and getting help in the future (if top-up session 2).

3.3 Therapist supervision

Therapists are advised to **attend supervision every 2 weeks** in order to support them to deliver the modified PATH intervention to people with mild to moderate dementia and depression, facilitate their learning, and ensure adequate adherence to the treatment manual. This should be viewed as an opportunity for therapists to reflect on their experiences of delivering the intervention and to discuss the following issues: 1) any difficulties or issues that are arising in delivering the intervention; 2) any tension between the person with dementia and the caregiver; 3) any reports of suicidal ideation (with or without plans or intent); 4) any safeguarding concerns; and 5) personalised treatment summaries. A **prompt sheet that serves as a reminder of what therapists should discuss in supervision has been created to facilitate this process** (see "Issues to discuss in supervision" template in Appendix 5).

In addition to therapists attending supervision, supervisors are encouraged to attend their own group supervisory sessions via teleconference. These will be scheduled every 4-6 weeks, and will be helpful for monitoring common issues in delivering the intervention across sites, as well as sharing overall feedback from independent raters.

3.4 Key points to bear in mind when delivering PATH

Some key points to bear in mind when delivering the modified PATH intervention are listed below. These are also summarised in a "cheat sheet" (i.e. a brief reference guide) in Appendix 6, along with a reminder of the stages of PATH and ways to regulate emotions.

3.4.1 Stay focused on emotions

As noted earlier, because solutions to problems may not necessarily reduce depression, the focus of PATH's problem solving approach is to reduce negative emotions and promote positive emotions that are associated with the specific problem. To achieve emotion regulation (Gross, 1998; Gross, 2014), the PATH therapist helps the person with dementia find the best possible solution to their problem, but also helps them to regulate their emotions if the solution is not satisfactory or the problem cannot be solved (i.e. helps them to adapt to their problem so that they can best live their life with their problem). **The aim of PATH is to help regulate the emotions that are being triggered by problems by exploring ways in which negative emotions can be decreased and positive emotions can be increased.**

3.4.2 Adapt how you engage the person with dementia

It is important to ask questions that encourage and guide the person with dementia and their caregiver to develop their own strategies for reducing negative emotions and increasing positive emotions. However, ways of engaging the person with dementia in the sessions will need to be adapted on an individual basis, depending on cognitive impairment. This is so that those with milder dementia do not perceive that things are being dumbed down for them, and those with more moderate dementia do not feel that their cognitive or functional abilities are being tested.

Consequently, therapists are advised to use two levels of prompts when working with people with dementia: **Type A prompts for those with milder dementia and Type B prompts for those with more moderate dementia**. Examples of these are illustrated in Table 6. It is likely that therapists will need to use a mixture of both prompts with a person with dementia as their ability to engage in discussions will differ depending on the type of problem and the person's specific cognitive strengths and weaknesses. If Type B prompts are needed, then it is likely that more caregiver involvement will be required, and therapists will need to be aware that they are not talking over the person with dementia. If it is not possible to engage the person with dementia in discussions (e.g. due to significant verbal comprehension problems), then the therapist should seek consent from the person with dementia to discuss things further with the caregiver.

3.4.3 Stay focused on the person with dementia

As noted in Section 2.4.1, it is important that therapists ensure that the focus of the intervention remains on the person with dementia and not the caregiver. Further guidance about this is listed in Section 2.4.1.

3.4.4 Set an appropriate pace for the sessions

The pace at which people with dementia and their caregivers will be able to engage in the sessions will vary between participants. People with milder dementia may be able to proceed at a faster pace (and hence get through more in the sessions) than people with more moderate dementia. Additionally, those who are more moderately depressed may need to go at a slower pace than those who are more mildly depressed. In cases where the person with dementia is only able to tolerate a short face-to-face interaction, permission should be sought from the person with dementia to speak separately with the caregiver, if appropriate. Be aware that the pace may need to be slower on 'bad days' with respect to memory, confusion or mood.

Table 6: Use of Type A and Type B prompts with people with dementia.

**Type A prompts (for those with milder dementia)**	**Type B prompts (for those with more moderate dementia)**
"Here’s the list of problems that you reported you have been experiencing." <Show list of problems.> Which problem would you like to focus on first?"	"Here’s the list of problems that you reported you have been experiencing." <Show list of problems.> "No longer doing the things that you used to enjoy appears to be quite upsetting for you… shall we work on that? What do you think?"
"What kind of things did you used to enjoy doing?"	"Did you used to enjoy doing any of these activities?" <Show list of activities.>
"What’s getting in the way of you being able to do the things that you used to enjoy?"	"A number of things can get in the way of us doing the things that we used to enjoy. Are any of the following things getting in the way of you doing stuff that you enjoy?" <Show list of common obstacles or barriers.>
"How does no longer being able to do X make you feel?"	"Not doing the things we used to enjoy doing can make us feel sad or frustrated. Does not being able to do X make you feel like this?" <Show list of emotions.>
"You mentioned that problems with your memory and organising things are getting in the way of you doing the things that you used to enjoy." <Show list of tools to support memory, attention and planning.> "Could you use any of these tools to help you with these difficulties?"	"You mentioned that problems with your memory and organising things are getting in the way of you doing the things that you used to enjoy." <Show list of tools to support memory, attention and planning.> “These tools can be used to support your memory and ability to organise things. Can I show you how we can use them to help you?”
"Sometimes it’s helpful to distract ourselves when we’re feeling frustrated. What kind of things help to distract you when you're feeling frustrated?" <Show list.>	"Sometimes it’s helpful to distract ourselves when we’re feeling frustrated. One way is X. Might that be helpful for you?" <Show list.>
"So, you’d like to go to see a film on Saturdays with a friend. If you were to break this task down into smaller steps, what might these be?"	"So, you’d like to go to see a film on Saturdays with a friend. If we were to break this task down into smaller steps, you would need to do X. What do you think?"
“… Is there a phrase that you could say to yourself to help you to do something, even if you don’t feel like doing it?”	“… Sometimes people find it useful to have a phrase that spurs them on to do something, even when they don’t feel like doing it. These are phrases such as “Just do it”, “You can do it” or “You don’t have to feel like it to do it”. Would something like this be useful for you?”
“Sometimes it helps to have another person involved to help us complete things. For example, they might remind us to do something. Would this be helpful for you in this situation? What might be useful for you?”	“Sometimes it helps to have another person involved to help us complete things. For example, they might remind us to do something. Would it be OK if [name of caregiver] helps you to do X?”

3.4.5 Use behavioural activation techniques to help people with dementia engage in pleasurable activities

Depression, cognitive impairment, and disability may interact and contribute to lack of participation in activities. This can isolate the person with dementia further and exacerbate their depressed mood. To break this cycle, it is important to help the person with dementia engage in pleasurable activities. Pleasurable activities include calling a friend, going to the cinema, reading a book, visiting the grandchildren, and exercising. Don’t forget to ask your clinical team for local knowledge and advice (e.g. about local groups, agencies, centres, befriending opportunities), if necessary.

Once pleasurable activities are identified (a worksheet is included for this), **the therapist and the person with dementia, with the help of the caregiver, will make a detailed plan on how to engage in at least two pre-selected activities weekly**. A caregiver's observation and knowledge of the person with dementia may provide crucial insight into the selection, initiation, and continuation of pleasurable activities.

The following behavioural activation techniques may be beneficial when the person with dementia has significant difficulty in engaging in pleasurable activities:

a) Identifying pleasurable activities (see Activities that I enjoy doing worksheet);
a) Keeping a weekly calendar of specific activities, including the time and day of the activity (see Calendar of

activities worksheet);
b) Monitoring the frequency of engagement in each activity per week, as well as the degree of pleasure when involved in each activity (see Calendar of activities worksheet);
c) Creating a detailed plan to help the person with dementia engage in specific activities (e.g. get ready and dress at 9 am; call for a taxi at 9:15 am; go to the Day Centre at 10 am) (see Activity plan - smaller steps worksheet);
d) Making motivating phone calls to help the person with dementia initiate the activity (e.g. the caregiver may call the person on the morning of the planned activity).

Sometimes, people may hold a long-standing belief that may interfere with behavioural change. For example, they may hold the belief "I must be motivated to engage in an activity" or "I must feel like doing something before I can do it", without acknowledging the benefits of engaging in activities without prior motivation. In these cases, the therapist may explore the pros and cons or the advantages and disadvantages of that belief and propose alternative statements. For example, "Even though I don't feel like doing __________, I'll enjoy it when I do it", "Instead of waiting to become motivated before I can engage in an activity, let me try to engage in the activity to improve my motivation", or "Doing something at least distracts me from difficult thoughts or feelings".

> *What are the advantages of believing 'I must feel like doing something before I can do it'? How is that helping you?... And what are the disadvantages of believing 'I must feel like doing something before I can do it'? How is it getting in the way?... If you were to believe a different thought such as 'Just do it even if you don't feel like it', how might that help you?"*

3.4.6 Ensure adequate discussion of home practice

Home practice is a critical aspect of PATH. It helps the person with dementia and the caregiver to think about and work on the situations and the problems and to make the most of therapy. Home practice is a collaborative effort between the therapist and the person with dementia (and the caregiver when necessary). The therapist should:

i) Provide a rationale for the home practice;
ii) Discuss possible obstacles or barriers to completing the home practice with the person with dementia and the caregiver;
iii) Discuss how helpful the home practice might be for them;
iv) Positively reinforce any home practice that is completed (i.e. bring awareness to any positive consequences of engaging in the home practice such as feeling better).

If these steps are carefully followed, the chances of the person with dementia and the caregiver completing the home practice will increase significantly.

3.5 Engaging people with dementia in psychological therapy

Some standard therapeutic skills for helping people with dementia to engage in psychological therapy are listed below:

- **Allow time** for each person in the session to be heard and listened to.
- **Avoid giving too much information** in a short space of time.
- **Allow people time** to take in information, process the information, and respond to any questions being asked.
- Use the person’s **own terminology** (their own words) for terms such as depression, dementia, home practice (“things to try out before the next session” is used here, but they may prefer another term), etc.
- Keep in mind the person with dementia's **capacity to consent to making decisions** in relation to the intervention throughout the sessions. Do not automatically assume that a person does not have capacity to consent to a decision in relation to the intervention. Check ongoing consent with the person with dementia throughout the sessions (e.g. check understanding about any decisions made and check that the person with dementia agrees to any decisions being made).
- **Provide verbal summaries** of what has been discussed throughout the session, and summarise this in writing at the end so that this can serve as a reminder for the person with dementia.

[Return to Table of Contents]

Chapter 4: Troubleshooting

Some common obstacles and pitfalls that therapists may encounter during the delivery of the modified PATH intervention are listed below.

4.1 Common obstacles

4.1.1 Difficulties with organising the list of problems and/or narrowing complaints to a specific problem

Depending on the severity of their depression and the degree of limitations imposed by cognitive impairment, people with dementia may have difficulty in organising their list of problems and/or narrowing their complaints to a specific problem. Alternatively, they may struggle with distinguishing crucial from trivial information. The therapist has to identify the person’s cognitive limitations and help them clarify their list of problems/difficulties and the main aspects of each problem/difficulty. The person's cognitive limitations will guide the selection of tools to support memory, attention and planning and determine the role of the caregiver. Some examples of problems faced by people with mild to moderate depression and dementia, which were identified from our qualitative interviews and focus groups, are provided in Appendix 7.

4.1.2 Difficulties implementing emotion regulation strategies

People with dementia may have difficulty implementing an emotion regulation strategy. A written plan for implementing a strategy and how to use the tools to support memory, attention, and planning should be prepared by the therapist while working with the person with dementia (see Chapter 2). As well as providing suggestions for how to use these tools in order to make a plan for testing out the best strategy, the therapist may need to teach the person with dementia how to use them. For example, the therapist may teach a person how to use a detailed calendar of timed activities, rehearse with the person the steps necessary for putting a chosen solution into practice, and may even practice this using role-playing, when appropriate. **Role-plays are critical in any PATH stage as they provide useful information on the difficulties the person has.** The person with dementia and the caregiver are asked to keep a journal of difficulties encountered when putting a solution into practice. Their thoughts and concerns should be reviewed during subsequent sessions. The therapist may also supervise putting a solution into practice, if necessary.

4.2 Common pitfalls

Tables 7 and 8 illustrate some frequently encountered issues that PATH therapists may face when delivering the modified PATH intervention, as well as possible ways of overcoming them. These are divided into therapist-related issues (Table 7) and issues with respect to the person with dementia and/or their caregiver (Table 8). In all cases, therapists should consider bringing the issue to supervision, if necessary.

Table 7: Common pitfalls with respect to therapists and possible ways of overcoming them.

**Common pitfall**	**Examples of possible ways of overcoming the issue**
Feeling overwhelmed as a therapist (e.g. due to identifying with the hopelessness that the person with dementia and/or caregiver may be experiencing).	Dealing with people with dementia and depression may trigger many emotions in the therapist, depending on the therapist's personality and personal experiences. The therapist may feel overwhelmed, helpless, hopeless, irritable, sad, or angry. Identifying these emotions and exploring them in supervision is a critical aspect of administering the intervention successfully. When you notice emotions coming up for you as a therapist:<br>a) Reflect on how the emotions are impacting on you (e.g. how intense the emotions are, how often you are thinking about this particular situation outside of therapy, whether there are any similarities between your personal life circumstances or history and the person with dementia/caregiver's situation);<br>b) Notice how this is impacting on treatment (e.g. is this getting in the way of finding the best solution or emotion regulation strategy?);<br>c) Try to see the situation from a different perspective (e.g. consider different realistically hopeful perspectives);<br>d) Consider whether feelings of hopelessness are present because the dyad are trying to solve an unsolvable problem. If they are, focus on emotional regulation strategies instead.
Spending too long getting caught up in lengthy discussions.	Give clear, simple instructions and relevant examples. Welcome scepticism and encourage people to try things out.
Talking too much as a therapist.	Reflect on why you feel you might be talking too much (e.g. not engaging the person with dementia, wanting to do well and inadvertently coming across as the 'expert'). Raise it in the session and address any reasons for talking too much. Try to ensure a balance between the person with dementia and caregiver feeling listened to and keeping focus on the manual.
Talking too little as a therapist (e.g. due to allowing people with dementia and/or caregivers to "just talk" or to go off topic).	Reflect on why you feel you might be talking too little (e.g. person going off topic, wanting to allow the person to feel heard, because it's easier than delivering the intervention). Address any reasons for talking too little. Remember that while people may report benefiting from being listened to in the short-term, the effects will only be short-lived. One of the aims of this intervention is to help people with dementia and/or caregivers to continue using the skills introduced beyond these sessions. Explain that you may need to interrupt at times if you notice yourselves being pulled off track in order to ensure that they get the most out of the sessions.
Trying to convince the person with dementia and/or caregiver that a possible solution will not work.	Try to avoid being patronising or prescriptive about how things should be done. Remember that the person with dementia and/or the caregiver are the expert in their situation. Discuss the pros and cons of possible solutions. If the person with dementia and/or caregiver are still convinced that the possible solution will work then see this as an opportunity for them to practice evaluating the choices that are made.
Focusing too much on 'weaknesses'.	It is important to highlight the person's past experience and retained skills (i.e. what they still can do) and build upon their existing strategies and knowledge throughout the sessions.
Focusing too little on increasing pleasurable activities.	Remember that improving mood in people with dementia and depression is not just about removing negative emotions, but also about promoting positive emotions.
Losing the focus on regulating mood (e.g. due to getting caught up in trying to solve a complex problem).	Remember that there are two ways of reducing negative emotions and increasing positive emotions: 1) solving problems; and 2) regulating mood. The latter is particularly important if problems are challenging to solve or cannot be solved. See Appendix 4 for case examples which illustrate how strategies for solving problems and regulating mood can be combined.
Trying to introduce tools to support memory, attention and planning that are too complicated.	Tools vary in how high tech they are (e.g. voice alarms, customised audio tapes and timed pre-recorded messages are fairly high tech). Start with low tech tools (e.g. strategically placed notepads, signs/pictures, calendars/diaries/weekly planners, whiteboards) unless high tech tools are already in place.

Table 8: Common pitfalls with respect to the person with dementia and/or caregiver and possible ways of overcoming them.

**Common pitfall**	**Examples of possible ways of overcoming the issue**
The person with dementia and/or caregiver report that they are not experiencing any problems or say “I just want to feel better”.	Some common problems that people with dementia and their caregivers reported in our qualitative interviews are presented in Appendix 7 and in the List of problems that can trigger negative feelings worksheet. These areas can be used as prompts, if necessary.
The person with dementia and/or caregiver "just wants to talk".	Gently explain the aim of PATH. Explore the pros and cons of "just wanting to talk". Set up the idea of the possible need for interruptions when introducing the session. Acknowledge, validate and normalise what is being said and then signpost to other sources of support.
The person with dementia perceives that their cognitive abilities are being tested.	Use Type B prompts (for those with moderate cognitive impairment) rather than Type A prompts (for those with mild cognitive impairment) so that the person with dementia does not perceive that their cognitive abilities are being tested.
The person with dementia says they do not feel like doing anything that will give them pleasure.	Explore whether they can do something even if they don’t feel like it . Explain that some people find they need to practice enjoying things again because it is as if they've "got out of the habit of enjoying things". Explain that some people find they can take a while to reconnect with pleasurable feelings again - at first they may even need to practice enjoying things or "going through the motions" until the feelings return.
The person with dementia and/or caregiver does not want to engage or is reluctant to engage in the intervention.	Explore the reasons for this. Discuss with the member of the local research team, if necessary.
The caregiver does most of the talking for the person with dementia.	Explore the reasons for this (e.g. the person with dementia feels anxious about answering questions and so the caregiver answers instead, or the caregiver has always done most of the talking for the person with dementia, even before the diagnosis). Use this understanding to decide how the person with dementia could be supported to engage in the conversations (e.g. use Type B prompts to help the person with dementia engage in the sessions). Have a separate discussion with the caregiver about the importance of the person with dementia being heard in the sessions (e.g. to feel empowered, engaged, etc), if necessary.
The caregiver wants to talk about his or her own problems.	Normalise and validate what the caregiver is reporting (e.g. *"It’s completely understandable for you to be struggling with this… a lot of people in your situation experience that difficulty too", "That sounds really challenging… I would find that situation difficult too"*). Emphasise that while the focus of the sessions are on the person with dementia, the skills introduced can be similarly applied to their own situation. Signpost the caregiver to other sources of support and/or make a referral for psychological support in their own right, if necessary.
The caregiver is reluctant to allow the person with dementia to test out a preferred solution (e.g. due to safety issues or time issues).	Normalise and validate the caregiver's concerns. Explore possible solutions to this problem. Evaluate the pros and cons of allowing the person with dementia to test out the preferred solution. If there are conflicting perceptions (e.g. the caregiver, but not the person with dementia, thinks the person is at risk of falling), then treat this as you would any other tension in the relationship (see Chapter 3). Consider whether the caregiver would benefit from psychoeducation about dementia or coping strategies (signpost to relevant resources or give the “List of coping strategies for caregivers” worksheet, if necessary).
The person with dementia feels upset about being asked about suicidal ideation.	Explain that this is a question that you have to ask each week as part of the study, and that we need to ask this question so that we can make sure people get the right support at the right time.
The caregiver keeps interrupting the session to explain that what the person with dementia is saying is not correct.	After the session, explain to the caregiver that you know that not everything the person with dementia says may be accurate, explain that it's not helpful in this context for the person with dementia to be reminded of what they're forgetting, and suggest that you discuss these kind of issues with the caregiver after the session.

Common pitfall	Examples of possible ways of overcoming the issue
The person with dementia or the caregiver says “they’ve tried it all before”.	Explain that what we hope to do here is to build on what they already know and are already doing by introducing some new ideas, as well as helping them to review what is and isn’t working for them.
The person with dementia and/or caregiver do not complete the home practice (e.g. do not complete a pleasurable activity or fail to test out a solution).	Explore what obstacles or barriers got in the way of completing it (particularly reluctance to change or try things out), how they are helping and how they might be overcome. Treat this as a new problem, if necessary. Before setting home practice, ensure that you: i) provide a rationale for the home practice; ii) discuss possible obstacles or barriers to completing the home practice with the person with dementia and the caregiver; and iii) discuss how helpful the home practice might be for them. When reviewing home practice, ensure that you positively reinforce any tasks that are completed by bringing attention to any positive or desirable consequences of completing the home practice (see session outlines).
The person with dementia and/or caregiver does not want to write things down (e.g. due to embarrassment about their reading or writing abilities).	Encourage the caregiver to write things down, if possible, or write things down yourself for them. Consider treating this as a new problem, if necessary and if highlighted by the person with dementia as a priority area of concern.
The person with dementia is reluctant to talk to the caregiver about an issue.	Explore what is underlying the reluctance to talk to the caregiver. Normalise and validate any concerns. Explore the pros and cons of talking about the issue. Be aware that this may be a source of tension between the person with dementia and the caregiver. Consider treating this as a new problem, if necessary and if highlighted by the person with dementia as a priority area of concern.
The caregiver tells you some sensitive information in the 5 mins discussion after the session.	Suggest bringing it into the next session so that it can be treated as a new problem, if necessary.
The problem list is focused around other people’s behaviour (e.g. children or partners).	Emphasise that it can be difficult to change other people’s behaviour. Consider treating the tension that arrives from the person with dementia’s dissatisfaction with other people’s behaviour as the ‘problem’. Help the person with dementia to manage any negative feelings that result from dissatisfaction with other people’s behaviour.
The person with dementia says they’ve completed an activity but the caregiver says they haven’t.	Be aware that this may be a source of tension between the person with dementia and the caregiver and that you may need to work with the tension. Have a discussion early on in the sessions with the caregiver to explain that you know that not everything the person with dementia says is going to be accurate and that this doesn’t need to be corrected in the session as it can make the person with dementia feel sad or frustrated. Acknowledge any negative emotions and explore ways of reducing them. Explore ways of helping the person with dementia to see what they have done (e.g. weekly planner of activities with a tick box indicating whether something was completed, which can be reviewed in the session).

[Return to Table of Contents]

Chapter 5: Session outlines, worksheets and handouts

5.1 Session outlines, worksheets and handouts

An outline of each session is presented in this chapter, along with the associated worksheets and handouts for each session. Suggestions for what to say to people with dementia and/or caregivers are presented in *italics* e.g. "*Problem Adaptation Therapy (or PATH) is a home-delivered treatment for depression...*". Suggestions for actions are given in triangular brackets e.g. <Give worksheet>. Key points and worksheets or handouts are highlighted in **bold** type.

A list of worksheets and handouts that are relevant for each session is presented in Table 9, and are provided after each session outline. Therapists may not need all of the worksheets and handouts that are listed for a specific session – which worksheets and handouts are used will depend on the cognitive abilities of the person with dementia.

(Press 'ctrl' at the same time as clicking on a heading to follow the link to that section.)

5.1.1 Session 1 - Assessment

In session 1, the modified PATH intervention is introduced to the person with dementia and the caregiver, and depression, cognitive strengths, and cognitive, physical and functional limitations in the person with dementia are explored. The caregiver's ability, willingness, and availability to help is also explored if there is time.

5.1.2 Session 2 - Things that trigger negative and positive emotions

Session 2 comprises an examination of the problems, concerns and situations that trigger negative emotions in the person with dementia, as well as identification of pleasurable activities that promote positive emotions (if there is time).

5.1.3 Sessions 3-7 - Techniques to reduce negative emotions and increase positive ones

In sessions 3-7, specific problems are identified to work on, associated negative emotions and ways to reduce negative emotions and increase positive ones are identified, and a plan is developed to test this out. It may only be possible for therapists to fully address 2-3 problems in detail in sessions 3-7, especially if the pace is slower or the problem is more complex.

5.1.4 Session 8 - Review

Session 8 comprises a review of which problems and negative emotions were addressed, and what emotion regulation strategies were used to reduce negative emotions and increase positive ones (including what caregiver assistance was needed and what tools to support memory, attention and planning were utilised).

5.1.5 Top-up sessions 1 & 2 - Recap and review

Finally, in top-up sessions 1 and 2, the personalised written summary developed in session 8 is reviewed and revised, as appropriate.

[Return to Table of Contents]

Table 9: Worksheets and handouts that accompany each session.

Session(s)	Title	Description
1	Information about Problem Adaptation Therapy (version A&B)	Provides an introduction to PATH.
	Patient Information Form (for therapists)	Lists areas to be explored in the assessment session.
	Caregiver Information Form (for caregivers to complete)	Assesses a caregiver's willingness and ability to help facilitate the intervention.
	Activities that I enjoy doing	Use to identify pleasurable activities as a technique for increasing positive emotions.
	Things to try out before the next session	Summarises what was discussed in the session and the plans for home practice.
2	List of problems that trigger negative emotions (for therapists)	Questions that therapists can ask to identify problems/situations that trigger negative emotions.
	List of problems that can trigger negative feelings	Use as a prompt to identify triggers (problems and situations that lead to negative emotions).
	List of negative feelings	Use as a prompt to identify negative emotions.
	Rating the impact of problems	Use to rate the impact of a problem on a person's day-to-day life.
	Activities that I enjoy doing	Use to identify pleasurable activities as a technique for increasing positive emotions.
	Calendar of activities	Use this calendar to schedule pleasurable activities for the following week.
	Things to try out before the next session	Summarises what was discussed in the session and the plans for home practice.
3-7	List of common obstacles or barriers	Use as a prompt to identify the obstacles or barriers that might be getting in the way of the person doing things.
	List of negative feelings	Use as a prompt to identify negative emotions.
	Activities that I enjoy doing	Use to identify pleasurable activities as a technique for increasing positive emotions.
	List of tools to support memory, attention and planning	Use as a prompt to identify tools or strategies that could be used to reduce negative emotions.
	Activity plan - smaller steps	Use as a prompt to identify tools or strategies that could be used to reduce negative emotions.
	List of strategies for distracting or soothing myself	Use as a prompt to identify tools or strategies that could be used to reduce negative emotions.
	Information about looking after yourself as a caregiver	Information about coping strategies for caregivers.
	Information about depression in people with dementia	Information about depression in people with dementia.
	Calendar of activities	Use this calendar to schedule pleasurable activities for the following week.
	Things to try out before the next session	Summarises what was discussed in the session and the plans for home practice.
8	Summary of the sessions (for therapists)	Summarises the problems or situations that were addressed in the sessions, and the techniques that were used to reduce negative emotions and increase positive ones.
	My summary of therapy - Problem _____ (version A)	Provides a template for the personalised summary of therapy for people with milder dementia.
	My summary of therapy - Problem _____ (version B)	Provides a template for the personalised summary of therapy for people with more moderate dementia.
	Calendar of activities	Use this calendar to schedule pleasurable activities for the following week.
	Things to try out before the next session	Summarises what was discussed in the session and the plans for home practice.
Top-up sessions 1-2	Information about Problem Adaptation Therapy (version A&B)	Provides an introduction to PATH.
	A summary of strategies used in PATH	Provides those with milder dementia and caregivers a reminder of the PATH steps.
	List of common obstacles or barriers	Use as a prompt to identify the obstacles or barriers that might be getting in the way of the person doing things.
	List of negative feelings	Use as a prompt to identify negative emotions.
	Activities that I enjoy doing	Use to identify pleasurable activities as a technique for increasing positive emotions.
	List of tools to support memory, attention and planning	Use as a prompt to identify tools or strategies that could be used to reduce negative emotions.
	Activity plan - smaller steps	Use as a prompt to identify tools or strategies that could be used to reduce negative emotions.
	List of strategies for distracting or soothing myself	Use as a prompt to identify tools or strategies that could be used to reduce negative emotions.
	Calendar of activities	Use this calendar to schedule pleasurable activities for the following week.
	Things to try out before the next session	Summarises what was discussed in the session and the plans for home practice.

5.1.1 Session 1 - Assessment

At the start of the session (5 mins)

1) Make sure you have all the **relevant equipment** with you (appropriate worksheets, pens, encrypted digital voice recorder, a watch so that you can keep track of time).

2) Introduce yourself and show your ID badge. Explain that you are their therapist in the PATHFINDER study. Establish the **preferred names** of the person with dementia and the caregiver.

3) Ensure that the person with dementia and and/or their caregiver have their **glasses or hearing aid**, if needed.

4) **Minimise distractions** (e.g. ask for the TV/radio to be turned off, see if noisy pets can be put in a separate room).

5) Try to ensure that the **seating arrangement** allows the therapist to sit closer to the person with dementia than the caregiver.

6) Ask permission to use the **digital voice recorder** (e.g. *"We'll be recording the session just so that my supervisor can check that I am facilitating these sessions as best as I can. They won't be focusing on what you're saying or doing - they will be focusing on what I am saying. Is that OK?"*). If the person with dementia and and/or their caregiver refuse to allow the session to be recorded then proceed with the session, make a note of why the session was not recorded in the study files, and discuss with your supervisor, if necessary.

7) Ask the person with dementia if they are **happy for their caregiver to be present** (e.g. *"Are you happy for [name of caregiver] to join us for this session, and to answer any questions that you or I might have?"*).

8) Remind the person with dementia that, with their consent, you will be having a **short discussion (5 mins) with the caregiver** after the session to discuss home practice (e.g. *"Would it be OK if I have short chat with [name of caregiver] after the session ends so that we can discuss any preparations that are needed for the next session?"*).

9) Discuss whether the person with dementia is having a **'good day' or a 'bad day'** with respect to feeling confused, muddled or low in mood (e.g. *"Before we begin, we know that people can have good days and bad days with respect to feeling confused, muddled or low in mood… it's helpful for me to know what kind of day you're having so that I don't go too quickly with things. Would you say this is a good day or a bad day for you?"*). Use this information to pace the session appropriately. Try not to get caught up in lengthy discussions about this.

10) Check that the person with dementia is **willing to participate** further in the session (e.g. *"Are you happy for us to continue with the session?"*).

11) Explain which **session you are on** (e.g. *"We're on session 1 out of 8 sessions. The aim of these sessions is to help you feel better by finding ways of reducing negative feelings and increasing positive feelings in your daily lives."*).

12) Briefly discuss the **agenda for today's session** (e.g. *"In today's session, I'm going to tell you about what this therapy involves, and we'll find out more about the difficulties you have been experiencing. At the end, I will summarise what we have discussed in the session, and what things you can try out in between now and the next session. Finally, I will have a brief chat with each of you to discuss how you found the session. How does that sound?"*).

13) Discuss the possible **need for interruptions** (e.g. *"At times I might need to interrupt you so that I can make sure we keep on track with what we've got planned for today's session and so that you get the most out of the session. Is that OK?"*). Politely interrupt when you notice yourselves going off topic (e.g. *"I'm really sorry to interrupt you… I'm just aware of the time and want you to get the most out of today's session. Could we come back to…?").*

Introduce PATH to the person with dementia and the caregiver (5-10 mins)

<Give "**Information about Problem Adaptation Therapy (version A)"** handout for people with milder dementia or "**Information about Problem Adaptation Therapy (version B)"** handout for people with more moderate dementia, and read out the accompanying script below (either version A or version B, as appropriate).>

Version A (for people with milder dementia):

"I'm going to start off by telling you a bit about this therapy. Problem Adaptation Therapy (or PATH as we like to call it) is a talking therapy that targets low mood and other negative feelings, as well as difficulties with functioning in day-to-day life. Research has shown that day-to-day problems and situations may contribute to low mood and other negative feelings such as anxiety or frustration. So it is important that we try to reduce these negative feelings in order to help you feel better."

"Therefore, in this therapy we're going to start by identifying problems and situations that trigger negative feelings for you." <Point to Step 1 in Figure 1.>

"And then we're going to explore different ways of reducing these negative feelings or reducing their impact on your life, as well as finding ways of increasing positive feelings." <Point to Step 2 in Figure 1.>

"And then we're going to help you to put a plan together for testing out which of these ways of helping you to feel better work best for you." <Point to Step 3 in Figure 1.>

"Do you have any questions about this?"

"In terms of the different ways of reducing negative feelings that we will explore, some of these ways might involve doing less of the things that trigger negative feelings, and doing more of the things that you enjoy." <Point to box 1 in Figure 1.>

"Some of these ways might involve doing things differently, for example, using memory aids or getting help from others." <Point to boxes 2 and 3 in Figure 1.>

"And some of these ways might involve finding different things that can help to distract you or soothe you when you're experiencing negative feelings." <Point to box 4 in Figure 1.>

"How does this sound?"

"So now let's talk about the structure of therapy. Therapy will consist of 8 sessions on a weekly basis, with each session lasting up to 1 hour. If you find one hour to be too much for you, then we can make the sessions shorter. I will make sure that we work at a pace that is acceptable for you."

"In the first two sessions, I will be asking you questions about your present and past difficulties so that we can better understand the problems and situations that might be making you feel worse. In Sessions 3-7, we will work on identifying the best ways to reduce negative feelings and increase positive ones in order to help you feel better. Finally, in session 8, we will review what we have discussed in the sessions, and will develop your own personalised summary of therapy, which you can use as a reminder of what helps you feel better."

"After we finish the 8 sessions, I will see you for two booster sessions, 3 and 6 months after we finish therapy. We'll recap on what we've discussed in these sessions and explore how you can continue to use what we've discussed to help you feel better."

"Do you have any questions?"

<To the person with dementia:> *"In terms of who's involved in these sessions, _________ [give name of caregiver] is here with your permission to help you get the most out of this therapy. The three of us will work together to try to help you feel better, using the techniques I have just described. We might ask _________ [give name of caregiver] to help you to try things out in between the sessions, but we'll always check if that's OK with you first."*

<To the caregiver:> *"At times, we may ask you to help _________ [give name of person with dementia] try out different things that might help them to feel better, based on what we've discussed in these sessions. We will be working on this together and so your feedback will be very important, particularly with respect to whether things were or were not helpful. Before we ask you to take part, I will discuss with you your availability and the extent of your participation."*

"How does this sound to you both?"

"Finally, let's talk about the location of therapy. The first two sessions will be in your own home so that I can get an idea about how problems and situations are triggering negative feelings in your day-to-day life. The remaining sessions will either be in your local clinic or at your home (if you are not able to travel to clinic). We will discuss this together to see what works best for us all."

"Do you have any questions?"

<u>Version B (for people with more moderate dementia):</u>

"I'm going to start off by telling you a bit about this therapy. Problem Adaptation Therapy (or PATH as we like to call it) is a talking therapy that is aimed at helping people to feel better. We know that day-to-day problems and situations can make people feel low in mood, anxious, frustrated, etc. So if we can reduce these negative feelings then we might be able to help people feel better."

"Do you have any questions about this so far?"

"We have three main aims in this therapy. First, we will try to find out what problems and situations make you feel low in mood, anxious, frustrated, etc." <Point to Step 1 in Figure 1.>

"Second, we will explore different ways of helping you to feel better. These might be ways that reduce negative feelings or ways that increase positive feelings." <Point to Step 2 in Figure 1.>

"Third, we will test out which of these ways of helping you to feel better work best for you." <Point to Step 3 in Figure 1.>

"How does this sound?"

"In terms of the structure of therapy, we will have 8 sessions together on a weekly basis, and each session will last up to 1 hour. If you find one hour to be too much for you, then we can make the sessions shorter. After we finish the 8 sessions, I will see you for two booster sessions, 3 and 6 months after we finish therapy."

"Do you have any questions?"

<To the person with dementia:> *"In terms of who's involved in these sessions, _________ [give name of caregiver] is here with your permission to help you get the most out of this therapy. The three of us will work together to try to help you feel better."*

<To the caregiver:> *"At times, we may ask you to help _________ [give name of person with dementia] try out different things that might help them to feel better, based on what we've discussed in these sessions. We will be working on this together and so your feedback will be very important, particularly with respect to whether things were or were not helpful. Before we ask you to take part, I will discuss with you your availability and the extent of your participation."*

"How does this sound to you both?"

"Finally, let's talk about the location of therapy. The first two sessions will be in your own home so that I can get an idea about problems and situations in your day-to-day life. The remaining sessions will either be in your local clinic or at your home (if you are not able to travel to clinic). We will discuss this together to see what works best for us all."

"Do you have any questions?"

<u>Evaluate depression, cognitive strengths and difficulties (i.e. what they can and cannot do), and physical and functional limitations in the person with dementia (20-30 mins)</u>

<Complete the **Patient Information Form**.>

Briefly summarise the session and ask for feedback (5 mins)

Briefly summarise what was covered in the session: *"Today we discussed _______. How did you find the session? What did you find helpful? What was less helpful?"*

Set the home practice (5 mins)

i) **Set the home practice** (phrased as “*things to try out before the next session*” or whatever term they prefer):

a) Ask the person with dementia and their caregiver to **write down any further comments or questions** they might have about therapy and bring them with them to the next session. **Give a rationale** for it (e.g. *"Sometimes questions won't come to you until you've had some time to think about things. It's important that we talk about any comments or questions you may have as soon as possible so that we can make sure you get the most out of the sessions"*).

b) Ask the person with dementia and their caregiver, if necessary, to complete the **Activities that I enjoy doing** worksheet. Explain how to complete it and ask them to bring it with them to the next session. **Give a rationale** for it (e.g. *"It is important for us to know what kind of things you enjoy doing so that we can see if a lack of these activities in your life is making you feel worse"*).

c) Ask the caregiver to complete the **Caregiver Information Form** and bring it with them to the next session. **Give a rationale** for this (e.g. *"It is helpful for us to know from the outset how much you might be able to help the person you care for take part in this therapy so that we do not over-burden you or ask you to do too much"*).

ii) Discuss **possible obstacles or barriers** to completing the home practice (e.g. *"What might stop you from trying out these things before the next session?", "What might get in the way?", "What could you do to ensure that you do them?”).*

iii) Discuss **how helpful the home practice might be** for them (e.g. *"How might it help if you try out these things before the next session?"*).

iv) <Give the **Things to try out before the next session** worksheet.> Help the person with dementia and/or caregiver to write a few notes about what was discussed in today’s session and to make a note of the home practice. Ask the person with dementia and/or caregiver to make some brief notes in relation to completing the home practice (see the **Things to try out before the next session** worksheet for prompts).

Individual time (5 mins each)

Allow **individual time with the person with dementia and their caregiver** (with the person with dementia’s consent) to discuss how they found the session, home practice and any issues raised in the session (5 mins each). If issues such as tension between the person with dementia and their caregiver are raised, then suggest that it would be helpful to bring this up in the next session as the problem to be addressed. Follow guidelines for working with tension in the subsequent session if the caregiver agrees to this.

After the session

Write some brief notes on **what worked well and any challenges** you faced so that you can bring this to supervision, if necessary.

[Return to Table of Contents]

<u>**INFORMATION ABOUT PROBLEM ADAPTATION THERAPY (version A)**</u>

<u>*What is Problem Adaptation Therapy?*</u>

Problem Adaptation Therapy (PATH) is a new form of talking therapy for people with mild to moderate dementia who are experiencing difficulties with low mood. It was originally developed in the USA and is now being tested in the UK with people with mild to moderate dementia who are experiencing low mood.

<u>*What are the goals of PATH?*</u>

PATH aims to help improve people's mood by finding ways of reducing negative feelings (such as sadness or frustration) and increasing positive feelings (such as happiness or contentment) in their daily lives. A plan is then put together to test out whether these ways of reducing negative feelings and increasing positive ones help you to feel better (see Figure 1).

Figure 1: Problem Adaptation Therapy

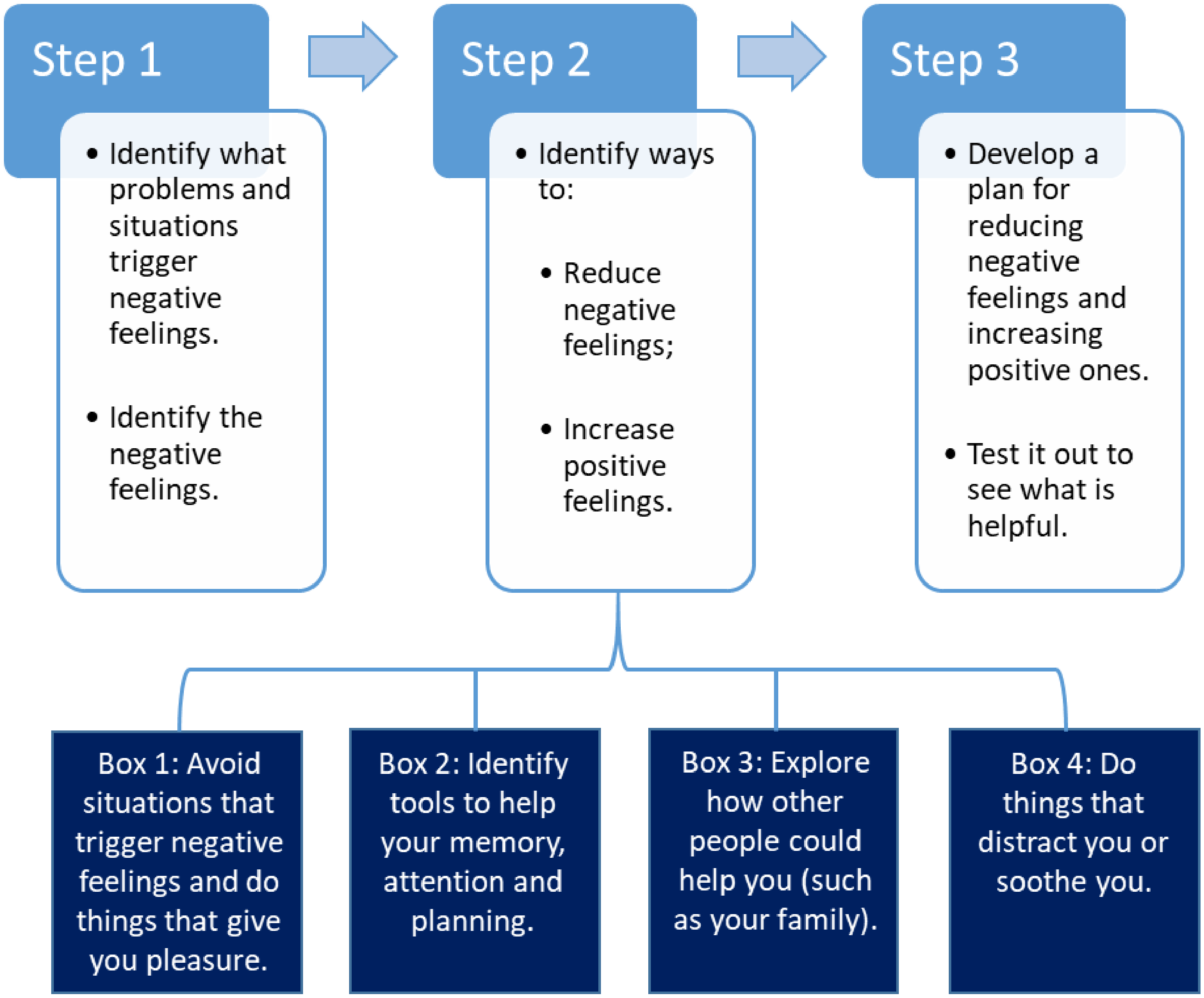

INFORMATION ABOUT PROBLEM ADAPTATION THERAPY (continued)

What will taking part in PATH involve?

- You and your caregiver (who may be a spouse, partner, family member or friend) will be seen for eight therapy sessions over the course of 12 weeks.
- There will be two further 'booster' sessions 3 and 6 months after the end of therapy.
- Each session will involve you and your caregiver meeting with the therapist for up to 1 hour. It is fine for sessions to be shorter than 1 hour if you need them to be.
- The therapist will work with you at a pace that is acceptable for you both.
- You and your caregiver (if necessary) will be asked to try out things in between sessions in order that we can find ways of helping you to feel better.

Where will PATH sessions take place?

- The first two PATH sessions will be in your own home so that the therapist can get an idea about how you are managing things in your daily life.
- The remaining sessions will either be in your local clinic or at your home (if you are not able to travel to clinic).

Who is my PATH therapist?

Your therapist's name is:

What should I do if we are unable to make an appointment or I need to contact my therapist between sessions?

If you find that you are unable to make an appointment (e.g. due to ill health) or you would like to speak to your therapist in between sessions, then please contact:

<u>INFORMATION ABOUT PROBLEM ADAPTATION THERAPY (version B)</u>

<u>What is Problem Adaptation Therapy?</u>

Problem Adaptation Therapy (PATH) is a new form of talking therapy for people with mild to moderate dementia who are experiencing difficulties with low mood that is being tested in the UK.

<u>What are the goals of PATH?</u>

PATH aims to help people feel better by finding ways of reducing negative feelings (such as sadness or frustration) and increasing positive feelings (such as happiness or contentment).

Figure 1: Problem Adaptation Therapy (PATH)

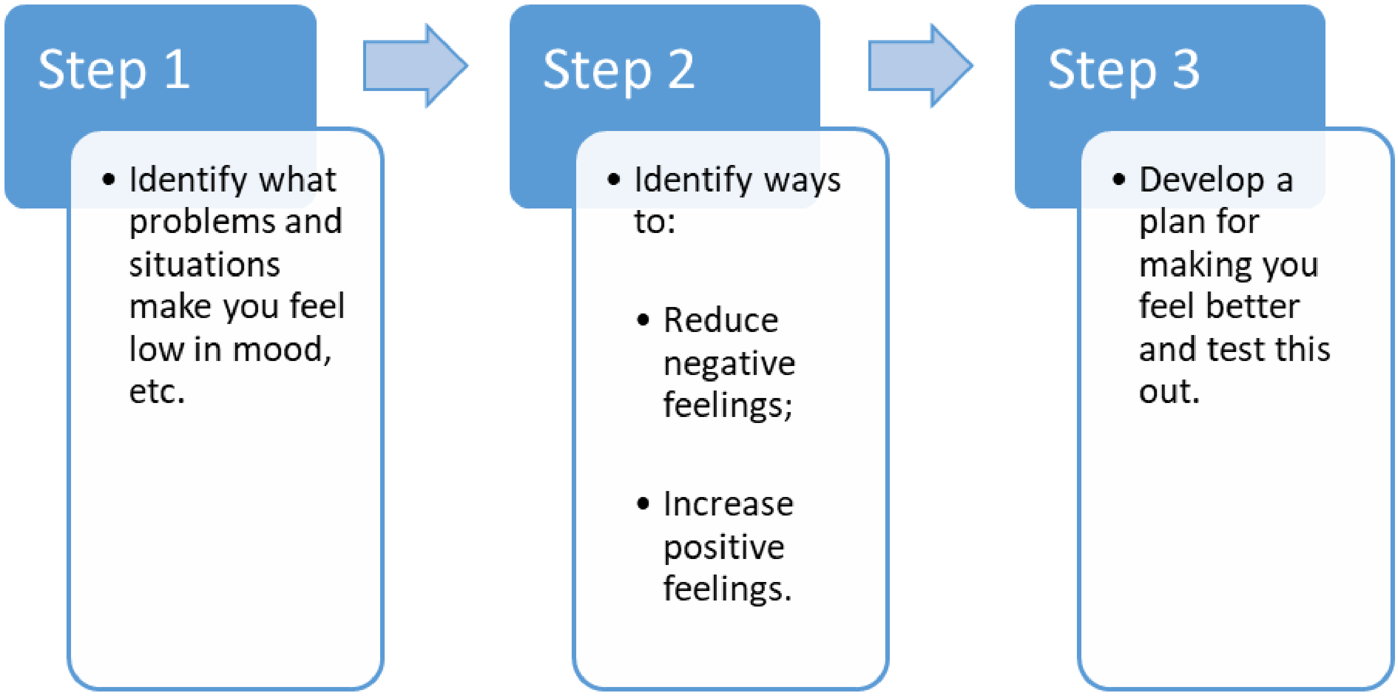

<u>What will taking part in PATH involve?</u>

- You and your caregiver will be seen for eight sessions over the course of 12 weeks. There will be two further sessions 3 and 6 months after the end of therapy.

- You and your caregiver will meet with the therapist for <u>up to</u> 1 hour in each session.

- You and your caregiver will be asked to try out things in between sessions in order that we can find ways of helping you to feel better.

INFORMATION ABOUT PROBLEM ADAPTATION THERAPY (continued)

Where will PATH sessions take place?

- The first two PATH sessions will be in your own home so that the therapist can get an idea about how you are managing things in your daily life.

- The remaining sessions will either be in your local clinic or at your home (if you are not able to travel to clinic).

Who is my PATH therapist?

Your therapist's name is:

What should I do if we are unable to make an appointment or I need to contact my therapist between sessions?

If you find that you are unable to make an appointment (e.g. due to ill health) or you would like to speak to your therapist in between sessions, then please contact:

PATIENT INFORMATION FORM (for therapists)

Session Number: ___________ *Patient ID:* ___________ *Date:* _________________

Please note: Responses to questions should be kept brief so that you can complete this assessment in 20-30 mins.

Current symptoms of depression

"Can you tell me about how you have been feeling in terms of your mood?"

If a prompt is needed then ask: *"Have you been experiencing any of the following symptoms of depression?"*

☐ Sad or depressed mood or feeling low most of the day, nearly every day
☐ Loss of interest or pleasure in activities that used to give pleasure
☐ Poor appetite or weight loss or increased appetite or weight gain
☐ Difficulties with sleeping or excessive sleepiness
☐ Difficulties with not being able to sit still (e.g. restlessness) or difficulties with feeling slowed down
☐ Fatigue or loss of energy
☐ Feelings of hopelessness, helplessness, worthlessness, excessive or inappropriate guilt, self-blame or failure
☐ Irritability

"How do these symptoms impact on your daily life? What do they stop you from doing?"

Suicidal ideation

"This is a standard question that we have to ask everybody in the study each week in case somebody needs more support than we are currently offering. Have you been feeling so bad that you have had thoughts about hurting yourself or others, that life is not worth living or that you'd be better off dead?" If yes, then assess further (see Appendix 8 for suicidal ideation guidelines and Appendix 3 for safeguarding guidelines).

Reported cognitive difficulties (i.e. what the person with dementia can no longer do or finds difficult)

"Can you tell me about the main difficulties you have been experiencing with your memory, attention, planning or thinking abilities?"

If a prompt is needed then ask: *"Have you been experiencing difficulties with: i) memory; ii) attention (e.g. staying focused on a task, being distractible, concentrating while reading or speaking to others); iii) planning (e.g. making plans and getting organised); iv) completing tasks (e.g. starting new tasks, sticking with a task until it is completed, performing tasks with several steps); or v) behaviour (e.g. repetitive behaviour, emotional outbursts)?"*

"How do difficulties with your memory, attention, planning or thinking abilities impact on your daily life? What do they stop you from doing? What concerns you the most?"

Overcoming difficulties or limitations

"What kind of things are you doing or using to help you overcome these difficulties and continue doing the things you want to do in your daily life?"

If a prompt is needed then ask: "*For example, do you use memory aids (e.g. calendars, diaries, alarms)? Do people close to you give you reminders about things? Do you get medicines delivered in a blister pack?"*

Other physical health, physical mobility or sensory problems

"Do you have any other problems with your physical health or physical mobility that impact on your daily life? Do you have any sensory problems (e.g. problems with vision or hearing) or problems with reading/writing?"

If yes: "How do these problems impact on your daily life? What do they stop you from doing?"

CAREGIVER INFORMATION FORM (for caregivers to complete)

It is helpful for us to know how much you might be able to help the person you care for to take part in this therapy so that we do not over-burden you or ask you to do too much.

What is your relationship to the person that you care for or support? *(please circle)*

Partner / spouse / family member / friend or neighbour / other

If other, then please specify:

Do you live with the person that you care for or support? *(please circle)* YES / NO

IF NO, how far away do you live from the person that you care for or support? If you don't know, then how long does it take you to travel to see them?

How often would you be willing to support the person you care for to take part in this therapy (bearing in mind your own duties and responsibilities)? *(please circle)*

____ Daily ____ times a week ____ times a month

Other (please specify):

Do you have any physical health conditions or mental health conditions (e.g. depression, anxiety) that may interfere with how much you are able to support the person you care for take part in therapy?

Are there any other things that we need to consider with respect to how much you are able to support the person you care for take part in therapy (e.g. full-time job, other caring responsibilities)?

ACTIVITIES THAT I ENJOY DOING

Some common activities that people enjoy doing are shown below. Which ones do you enjoy doing?

Put a tick in the box ☑ next to an activity if you think that you might enjoy it or have enjoyed it previously.

**Daily activities**	**Self-care & spiritual activities**	**Social activities**	**Leisure activities & hobbies**	**Outdoor & physical activities**
☐ Watching TV or listening to the radio	☐ Grooming (e.g. shaving, wearing makeup)	☐ Having people over for something to eat or drink	☐ Reading books, newspapers or magazines	☐ Going to a theatre, cinema, concert, gallery, museum
☐ Setting the table	☐ Wearing favourite clothes	☐ Spending time with family or friends	☐ Writing (e.g. letters, stories, poetry)	☐ Watching wildlife
☐ Cooking, preparing snacks or drinks	☐ Having a bath, shower or massage	☐ Chatting to family or friends (e.g. phone)	☐ Painting, drawing or doing crafts	☐ Going to watch sports (e.g. football game)
☐ Doing the cleaning or light housework	☐ Taking care of oneself (e.g. eating healthily, taking medication)	☐ Having meals or drinks with family or friends	☐ Knitting, crocheting or sewing	☐ Going on outings (e.g. park, picnic, shopping for leisure)
☐ Doing the laundry or ironing	☐ Going to the barber or hairdresser	☐ Discussing photos of family or friends	☐ Doing crosswords or puzzles	☐ Gardening
☐ Doing the dishes	☐ Going to a spiritual gathering (e.g. church) or retreat	☐ Recalling and discussing past events with others	☐ Listening to music, singing or playing a musical instrument	☐ Doing exercise (e.g. walking, swimming, cycling, gym)
☐ Doing the shopping	☐ Reading a spiritual text or praying	☐ Meeting new people	☐ Watching sports on TV (e.g. tennis)	☐ Going dancing
☐ Watching, listening to or reading about the daily news	☐ Practising meditation or relaxation	☐ Going to a social group or gathering	☐ Playing games (e.g. board games, computer games)	☐ Doing yoga, pilates or Tai Chi
Other:	Other:	Other:	Other:	Other:

THINGS TO TRY OUT BEFORE THE NEXT SESSION

Summary of today's session

What we discussed:

Home practice

What we agreed to try out before the next session:

1.

2.

3.

4.

THINGS TO TRY OUT BEFORE THE NEXT SESSION (continued)

Experience of home practice

If you have been able to complete what you agreed to try out before the next session, then please write down some brief notes about it:

How did it go?

How did you feel afterwards?

What was helpful?

What was not helpful?

If you come to the clinic for your sessions, please don't forget to bring your PATHFINDER materials with you to the next session.

5.1.2 Session 2 - Things that trigger negative and positive emotions

At the start of the session (5-10 mins)

1) Make sure you have all the **relevant equipment** with you (relevant worksheets, pens, encrypted digital voice recorder, a watch so that you can keep track of time).

2) Introduce yourself and show your ID badge. Explain that you are their therapist in the PATHFINDER study.

3) Ensure that the person with dementia and and/or their caregiver have their **glasses or hearing aid**, if needed.

4) **Minimise distractions** (e.g. ask for the TV/radio to be turned off, see if noisy pets can be put in a separate room).

5) Try to ensure that the **seating arrangement** allows the therapist to sit closer to the person with dementia than the caregiver.

6) Ask permission to use the **digital voice recorder** (e.g. *"We'll be recording the session just so that my supervisor can check that I am facilitating these sessions as best as I can. They won't be focusing on what you're saying or doing - they will be focusing on what I am saying. Is that OK?"*). If the person with dementia and and/or their caregiver refuse to allow the session to be recorded then proceed with the session, make a note of why the session was not recorded in the study files, and discuss with your supervisor, if necessary.

7) Ask the person with dementia if they are **happy for their caregiver to be present** (e.g. *"Are you happy for [name of caregiver] to join us for this session, and to answer any questions that you or I might have?"*).

8) Remind the person with dementia that, with their consent, you will be having a **short discussion (5 mins) with the caregiver** after the session to discuss home practice (e.g. *"Would it be OK if I have short chat with [name of caregiver] after the session ends so that we can discuss any preparations that are needed for the next session?"*).

9) Discuss whether the person with dementia is having a **'good day' or a 'bad day'** with respect to feeling confused, muddled or low in mood (e.g. *"Before we begin, we know that people can have good days and bad days with respect to feeling confused, muddled or low in mood… it's helpful for me to know what kind of day you're having so that I don't go too quickly with things. Would you say this is a good day or a bad day for you?"*). Use this information to pace the session appropriately. Try not to get caught up in lengthy discussions about this.

10) **Assess risk** (e.g. *"I now need to ask you a question about your mood that we ask everybody in the study in case they need more support than we are currently offering. Have you been feeling so bad that you have had thoughts about hurting yourself or others, that life is not worth living or that you'd be better off dead?")*. If this was reported in a previous session then ask: *"You previously mentioned X [describe what was previously reported with respect to suicidal ideation, plans, intent, protective factors] - has anything changed since then?"* If this has not been reported in a previous session, then assess further (see Appendix 8, if necessary).

11) Check that the person with dementia is **willing to participate** further in the session (e.g. *"Are you happy for us to continue with the session?")*.

12) Explain which **session you are on** (e.g. *"We're on session 2 out of 8 sessions. To recap, the aim of these sessions is to help you feel better by finding ways of reducing negative feelings and increasing positive feelings in your daily lives."*).

13) Briefly discuss the **agenda for today's session** (e.g. *"In today's session, I'm going to recap on what we discussed in the previous session, and we're going to review how you got on with the home practice. We're then going to identify the problems or situations that trigger negative emotions for you. At the end, I will summarise what we have discussed in the session, and what things you can try out in between now and the next session. Finally, I will have a brief chat with each of you to discuss how you found the session. How does that sound?")*.

14) Discuss the possible **need for interruptions** (e.g. *"At times I might need to interrupt you so that I can make sure we keep on track with what we've got planned for today's session and so that you get the most out of the session. Is that OK?"*). Politely interrupt when you notice yourselves going off topic (e.g. *"I'm really sorry to interrupt you… I'm just aware of the time and want you to get the most out of the session. Could we come back to…?")*.

Briefly review previous session and home practice (5 mins)

Review what was discussed in the **previous session**: *"In the previous session we discussed _______ [describe what was discussed in the previous session]."*

Review the **home practice**: *"The things to try out before today's session were to _______ [describe home practice]. Did you manage to do this? What was helpful? What was not helpful?"*

If no, then ask: *"What got in the way?"* or *"Was there a particular reason you didn't get round to doing it?* If the home practice has not been completed then consider re-setting it as home practice (and discussing ways of overcoming the barriers to completing it) or completing it in the session.

Positively reinforce any home practice that is completed by bringing attention to any positive benefits of completing it (e.g. feeling better). For example, *"What happened after you did ______?"* (Type A prompt) or *"As we talk about this, how does it feel, right now, that you were able to do ______?"* (Type B prompt). Highlight any **positive or desirable consequences** of completing the home practice.

Identify problems/concerns/situations that trigger negative emotions (20-30 mins)

<Complete the **List of problems that trigger negative emotions** worksheet.>

Check how the person with dementia is feeling (e.g. *"We've spent quite a while talking about the problems or difficult situations you are currently facing. How are you feeling after discussing all of this?").*

Briefly summarise the session and ask for feedback (5 mins)

Briefly summarise what was covered in the session: *"Today we discussed _______. How did you find the session? What did you find helpful? What was less helpful?"*

Set the home practice (5 mins)

i) **Set the home practice** (phrased as "*things to try out before the next session*" or whatever term they prefer):

a) If the **Activities that I enjoy doing** worksheet has been completed for home practice, then ask the person with dementia to **engage in <u>at least two</u> pleasurable activities** before the next session. **Give a rationale** for this (e.g. *"Doing things we enjoy can help improve how we're feeling"*). Give the **Calendar of activities** worksheet and explain how to complete it. Include the two planned pleasurable activities on this.

b) If the **Activities that I enjoy doing** worksheet has not been completed, then ask the person with dementia and their caregiver to complete it for home practice. Explain how to complete it and ask them to bring it with them to the next session. **Give a rationale** for it (e.g. *"It is important for us to know what kind of things you enjoy doing so that we can see if a lack of these activities in your life is making you feel worse"*).

ii) Discuss **possible obstacles or barriers** to completing the home practice (e.g. *"What might stop you from trying out these things before the next session?", "What might get in the way?", "What could you do to ensure that you do them?", "Is this something that [name of caregiver] could help you with?"*).

iii) Discuss **how helpful the home practice might be** for them (e.g. *"How might it help if you try out these things before the next session?"*).

iv) <Give the **Things to try out before the next session** worksheet.> Help the person with dementia and/or caregiver to write a few notes about what was discussed in today's session and to make a note of the home practice. Ask the person with dementia and/or caregiver to make some brief notes in relation to completing the home practice (see the **Things to try out before the next session** worksheet for prompts). In particular, ask the person with dementia and/or caregiver to notice whether they feel better after completing the home practice.

Individual time (5 mins each)

Allow **individual time with the person with dementia and their caregiver** (with the person with dementia's consent) to discuss how they found the session, home practice and any issues raised in the session (5 mins each). If issues such as tension between the person with dementia and their caregiver are raised, then suggest that it would be helpful to bring this up in the next session as the problem to be addressed. Follow guidelines for working with tension in the subsequent session if the caregiver agrees to this.

After the session

Write some brief notes on **what worked well and any challenges** you faced so that you can bring this to supervision, if necessary.

Consider how you can use PATH stages, tools to support memory, attention and planning, involvement of caregivers and strategies for regulating mood to help reduce negative emotions and increase positive emotions in the person with dementia. Some questions that may help conceptualise and guide therapy are presented in Appendix 9.

[Return to Table of Contents]

<u>LIST OF PROBLEMS THAT TRIGGER NEGATIVE EMOTIONS (for therapists)</u>

Session Number: ___________ *Patient ID:* ___________ *Date:* _________________

Ask the person with dementia and their caregiver the following questions. Make a note of any disagreements between the person with dementia and their caregiver (e.g. disagreements in reported problems, emotions, impact or priority).

1) *"What difficult problems or situations have you been struggling with in your daily life? What has been making you feel down or low in mood?'"*

If a prompt is needed then say: *"Here's a list of problems that can make people feel upset, sad, anxious or frustrated."* <Give **List of problems that can trigger negative feelings** worksheet.>

2) *"When __________ [name of the problem] occurs, how does this make you feel?"*

If a prompt is needed, then say: *"Here's a list of feelings that people can experience when problems or difficulties occur."* <Give "**List of negative feelings**" worksheet.> *"When __________ [name of the problem] occurs, do you experience any of these feelings?"*

3) *"When __________ [name of the problem] occurs, how much of an impact does it have on you or on your daily life?"* <Give **Rating the impact of problems** worksheet.>

4) *"Because we won't have time to address every problem you may have in these sessions, we need to decide which ones you would like to work on with me. How important is it for us to work on __________ [name of the problem] in these sessions? Is it a low, medium or high priority problem?"* <Use **Rating the impact of problems** worksheet.>

**Problem, difficulty, concern or situation in the daily life of the person with dementia**	**Negative emotions triggered by the problem, difficulty, concern or situation in the person with dementia** *(use their own words)*	**Impact of the problem, difficulty, concern or situation on the person with dementia (1 = minimal impact, 10 = worst impact)**	**Priority (low, medium or high)**
1.			
2.			
3.			
4.			
5.			

(Continued overleaf)

**Problem, difficulty, concern or situation in the daily life of the person with dementia**	**Negative emotions triggered by the problem, difficulty, concern or situation in the person with dementia** *(use their own words)*	**Impact of the problem, difficulty, concern or situation on the person with dementia (1 = minimal impact, 10 = worst impact)**	**Priority (low, medium or high)**
6.			
7.			
8.			
9.			
10.			

Summary of problems:

Tick which problems apply to the person with dementia and indicate the priority areas (e.g. imminent safety issues).

**Area**	**Problems**	**Area**	**Problems**
Health	☐ Side effects of medication	Daily activities	☐ Not being able to do things that they used to be able to do
	☐ Managing or taking medication		☐ Not being allowed to do things that they used to do
	☐ Physical health and/or pain		☐ Loss of structure to the day
	☐ Attending appointments		☐ Lack of pleasurable activities
	☐ Safety	Cognitive abilities	☐ Difficulties with memory
Physical or sensory	☐ Limited physical mobility		☐ Difficulties with attention or concentration
	☐ Reduced vision or hearing		☐ Difficulties with planning or organising things
Relationships	☐ Keeping friendships going		☐ Problems with reading or writing
	☐ Relationships with others	Emotional or psychological	☐ Waking up feeling depressed
	☐ How they are being cared for by others		☐ Lack of interest or motivation
Finances	☐ Making ends meet		☐ Lack of confidence
	☐ Pensions, benefits or housing		☐ Loss of identity
	☐ Managing money		☐ Fear of being a burden
Other		Other	

<u>LIST OF PROBLEMS THAT CAN TRIGGER NEGATIVE FEELINGS</u>

**What problems or situations are currently causing you distress (including feelings of sadness, anxiety, embarrassment, anger, guilt, shame, hopelessness, etc)?**	**Tick if this problem or situation is having a significant impact on your day-to-day life or on your mood (✓)**
1.	
2.	
3.	
4.	
5.	
6.	
7.	

If you are struggling to think about problems or situations that are currently causing you distress, please look at the examples on the next page.

LIST OF PROBLEMS THAT CAN TRIGGER NEGATIVE FEELINGS (continued)

**Area of life**	**Are any of the following problems or situations currently causing you distress?**	**Tick (✓) if this is affecting your mood**	**Tick (✓) if this is having a significant impact on your day-to-day life**
Health	Side effects of medication (e.g. drowsiness, feeling sick)		
	Managing or taking medication		
	Physical health and/or pain		
	Attending appointments		
	Safety (e.g. getting lost or feeling confused when out)		
Physical or sensory	Limited physical mobility		
	Reduced vision or hearing		
Relationships	Keeping friendships going		
	Relationships with others (e.g. partner, family or friends)		
	How you are being cared for by others		
Finances	Making ends meet		
	Pensions, benefits or housing		
	Managing money (e.g. paying bills)		
Daily activities	Not being able to do things that you used to be able to do		
	Not being allowed to do things that you used to do		
	Loss of structure to the day		
	Lack of pleasurable activities		
Thinking abilities	Difficulties with memory		
	Difficulties with attention or concentration		
	Difficulties with planning or organising things		
	Problems with reading or writing		
Emotional or psychological	Waking up feeling depressed		
	Lack of interest or motivation		
	Lack of confidence		
	Loss of identity		
	Fear of being a burden		

LIST OF NEGATIVE FEELINGS

Some common negative feelings that people experience when they are faced with a difficult problem or situation are shown below.

**How do you feel when you are faced with this difficult problem or situation?**				
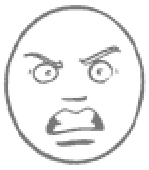	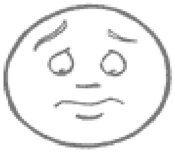	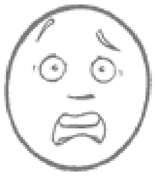	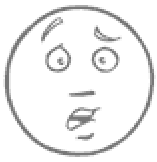	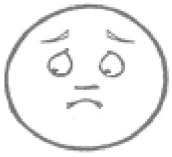
Angry	Miserable	Scared	Hurt	Ashamed
Annoyed	Sad	Anxious	Betrayed	Guilty
Frustrated	Lonely	Worried	Offended	Regretful
Irritated	Discouraged	Stressed	Rejected	Embarrassed
Impatient	Unmotivated	Confused	Disappointed	Disgusted
Other (please specify):				

RATING THE IMPACT OF PROBLEMS

After you've identified a problem or difficult situation, it is useful to think about what impact it is having on your daily life. This can help you to decide whether you need to try and do something about it.

For each problem or difficult situation that you identify, ask yourself the following question:

"When this problem or difficult situation occurs, how much of an impact does it have on me or my day-to-day life?"

Use the scale below to help you decide. The higher the score, the greater the impact the problem or difficult situation has on your day-to-day life.

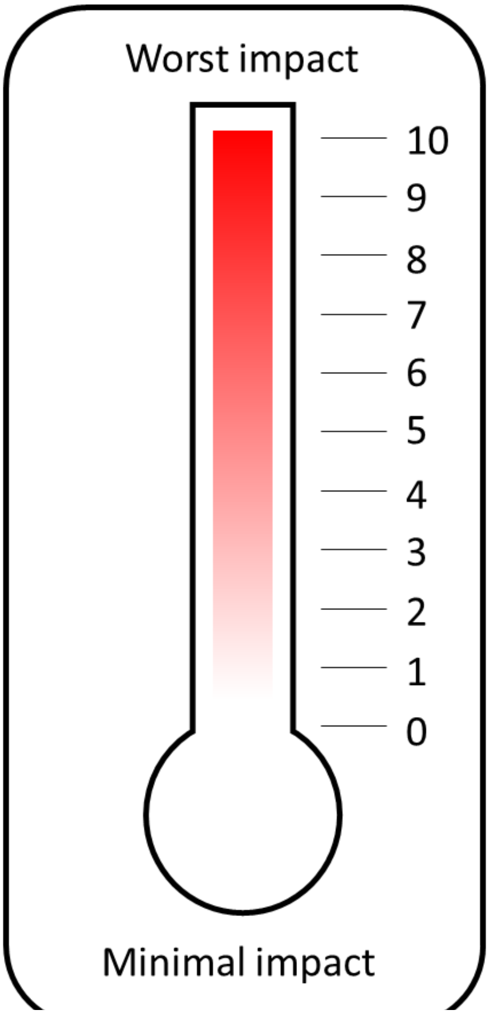

Finally, ask yourself: *"How important is it for us to work on this problem or difficult situation and the negative feelings associated with it in these sessions?"*

LOW **MEDIUM** **HIGH**

ACTIVITIES THAT I ENJOY DOING

Some common activities that people enjoy doing are shown below. Which ones do you enjoy doing?

Put a tick in the box ☑ next to an activity if you think that you might enjoy it or have enjoyed it previously.

**Daily activities**	**Self-care & spiritual activities**	**Social activities**	**Leisure activities & hobbies**	**Outdoor & physical activities**
☐ Watching TV or listening to the radio	☐ Grooming (e.g. shaving, wearing makeup)	☐ Having people over for something to eat or drink	☐ Reading books, newspapers or magazines	☐ Going to a theatre, cinema, concert, gallery, museum
☐ Setting the table	☐ Wearing favourite clothes	☐ Spending time with family or friends	☐ Writing (e.g. letters, stories, poetry)	☐ Watching wildlife
☐ Cooking, preparing snacks or drinks	☐ Having a bath, shower or massage	☐ Chatting to family or friends (e.g. phone)	☐ Painting, drawing or doing crafts	☐ Going to watch sports (e.g. football game)
☐ Doing the cleaning or light housework	☐ Taking care of oneself (e.g. eating healthily, taking medication)	☐ Having meals or drinks with family or friends	☐ Knitting, crocheting or sewing	☐ Going on outings (e.g. park, picnic, shopping for leisure)
☐ Doing the laundry or ironing	☐ Going to the barber or hairdresser	☐ Discussing photos of family or friends	☐ Doing crosswords or puzzles	☐ Gardening
☐ Doing the dishes	☐ Going to a spiritual gathering (e.g. church) or retreat	☐ Recalling and discussing past events with others	☐ Listening to music, singing or playing a musical instrument	☐ Doing exercise (e.g. walking, swimming, cycling, gym)
☐ Doing the shopping	☐ Reading a spiritual text or praying	☐ Meeting new people	☐ Watching sports on TV (e.g. tennis)	☐ Going dancing
☐ Watching, listening to or reading about the daily news	☐ Practising meditation or relaxation	☐ Going to a social group or gathering	☐ Playing games (e.g. board games, computer games)	☐ Doing yoga, pilates or Tai Chi
Other:	Other:	Other:	Other:	Other:

CALENDAR OF ACTIVITIES

1. Write down in a few words the pleasurable activity that you will do over the next week.<br><br>2. After you have completed the activity, rate how much you enjoyed this activity from 1 (not at all) to 6 (very much) using the rating scale on the right.	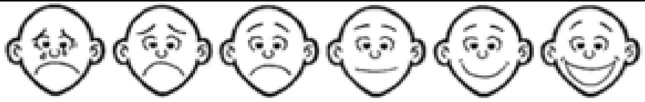<br>1 2 3 4 5 6<br>Not at all Very much

**Mon AM**	**Tues AM**	**Wed AM**	**Thu AM**	**Fri AM**	**Sat AM**	**Sun AM**
e.g. Go shopping 5	*e.g. Go to the Lunch Club 4*		*e.g. Go to the Lunch Club 5*		*e.g. Go to the cinema 6*	
**Mon PM**	**Tues PM**	**Wed PM**	**Thu PM**	**Fri PM**	**Sat PM**	**Sun PM**
		e.g. Coffee with friend 5		*e.g. Go for a walk 4*		*e.g. Dinner with family 6*

THINGS TO TRY OUT BEFORE THE NEXT SESSION

Summary of today's session

What we discussed:

Home practice

What we agreed to try out before the next session:

1.

2.

3.

4.

THINGS TO TRY OUT BEFORE THE NEXT SESSION (continued)

Experience of home practice

If you have been able to complete what you agreed to try out before the next session, then please write down some brief notes about it:

How did it go?

How did you feel afterwards?

What was helpful?

What was not helpful?

If you come to the clinic for your sessions, please don't forget to bring your PATHFINDER materials with you to the next session.

5.1.3 Sessions 3-7 - Problem solving

At the start of the session

1) Make sure you have all the **relevant equipment** with you (relevant worksheets, pens, encrypted digital voice recorder, a watch so that you can keep track of time).

2) Introduce yourself and show your ID badge. Explain that you are their therapist in the PATHFINDER study.

3) Ensure that the person with dementia and and/or their caregiver have their **glasses or hearing aid**, if needed.

4) **Minimise distractions** (e.g. ask for the TV/radio to be turned off, see if noisy pets can be put in a separate room).

5) Try to ensure that the **seating arrangement** allows the therapist to sit closer to the person with dementia than the caregiver.

6) Ask permission to use the **digital voice recorder** (e.g. *"We'll be recording the session just so that my supervisor can check that I am facilitating these sessions as best as I can. They won't be focusing on what you're saying or doing - they will be focusing on what I am saying. Is that OK?"*). If the person with dementia and and/or their caregiver refuse to allow the session to be recorded then proceed with the session, make a note of why the session was not recorded in the study files, and discuss with your supervisor, if necessary.

7) Ask the person with dementia if they are **happy for their caregiver to be present** (e.g. *"Are you happy for [name of caregiver] to join us for this session, and to answer any questions that you or I might have?"*).

8) Remind the person with dementia that, with their consent, you will be having a **short discussion (5 mins) with the caregiver** after the session to discuss home practice (e.g. *"Would it be OK if I have short chat with [name of caregiver] after the session ends so that we can discuss any preparations that are needed for the next session?"*).

9) Discuss whether the person with dementia is having a **'good day' or a 'bad day'** with respect to feeling confused, muddled or low in mood (e.g. *"Before we begin, we know that people can have good days and bad days with respect to feeling confused, muddled or low in mood… it's helpful for me to know what kind of day you're having so that I don't go too quickly with things. Would you say this is a good day or a bad day for you?"*). Use this information to pace the session appropriately. Try not to get caught up in lengthy discussions about this.

10) **Assess risk** (e.g. *"I now need to ask you a question about your mood that we ask everybody in the study in case they need more support than we are currently offering. Have you been feeling so bad that you have had thoughts about hurting yourself or others, that life is not worth living or that you'd be better off dead?")*. If this was reported in a previous session then ask: *"You previously mentioned X [describe what was previously reported with respect to suicidal ideation, plans, intent, protective factors] - has anything changed since then?"* If this has not been reported in a previous session, then assess further (see Appendix 8, if necessary).

11) Check that the person with dementia is **willing to participate** further in the session (e.g. *"Are you happy for us to continue with the session?")*.

12) Explain which **session you are on** (e.g. *"We're on session ________ out of 8 sessions. To recap, the aim of these sessions is to help you feel better by finding ways of reducing negative feelings and increasing positive feelings in your daily lives."*).

13) Briefly discuss the **agenda for today's session** (e.g. *"In today's session, I'm going to recap on what we discussed in the previous session, and we're going to review how you got on with the home practice. We're then going to start exploring ways of reducing negative emotions and increasing positive ones in relation to one or more of the problems we identified in the last session. At the end, I will summarise what we have discussed in the session, and what things you can try out in between now and the next session. Finally, I will have a brief chat with each of you to discuss how you found the session. How does that sound?")*.

14) Discuss the possible **need for interruptions** (e.g. *"At times I might need to interrupt you so that I can make sure we keep on track with what we've got planned for today's session and so that you get the most out of the session. Is that OK?"*). Politely interrupt when you notice yourselves going off topic (e.g. *"I'm really sorry to interrupt you…*

I'm just aware of the time and want you to get the most out of the session. Could we come back to…?").

Briefly review previous session and home practice (5 mins)

Review what was discussed in the **previous session**: *"In the previous session we discussed _______ [describe what was discussed in the previous session]."*

Review the **home practice**: *"The things to try out before today's session were to _______ [describe home practice]. Did you manage to do this? What was helpful? What was not helpful? "*

If no, then ask: *"What got in the way?"* or *"Was there a particular reason you didn't get round to doing it?* If the home practice has not been completed then consider re-setting it as home practice (and discussing ways of overcoming the barriers to completing it) or completing it in the session.

Positively reinforce any home practice that is completed by bringing attention to any positive benefits of completing it. For example, *"What happened after you did ______?"* (Type A prompt) or *"As we talk about this, how does it feel, right now, that you were able to do ______?"* (Type B prompt). Highlight any **positive or desirable consequence**s of completing the home practice.

Complete steps for reducing negative emotions and increasing positive emotions (30 mins)

There are 3 steps involved in reducing negative emotions, increasing positive emotions and potentially solving problems identified by the person with dementia and their caregiver. **How many of these steps you complete in the session will depend on the pace of the session, the ability of the person with dementia to engage in the session and the complexity of the problem.** If steps are not completed in this session then this can be continued in the next session. If you need to repeat steps in order to help a person learn the information then do this.

Step 1

1) Help the person with dementia to **select a problem to work on in today's session**. Use the list of problems identified in Session 2 as a prompt and ask: *"What problem would you like to work on in today's session?"*

2) **If the problem needs to be broken down** into smaller problems, then help the person to **select which smaller problem to focus on**: *"This is quite a large problem. We need to break it down into the following smaller problems ________ - which should we focus on today?"*

3) **Ask for more details about the problem or situation** that is triggering negative feelings for the person with dementia: *"Can you tell me more about that?"*. Give prompts if needed: *"When? Where? How often? With whom?"*

4) Explore their perceptions of **why this problem or situation is arising**:*"Why do you think ______ [problem] is happening?*

Use the **List of common obstacles or barriers** worksheet as a prompt, if necessary.

5) Find out **what negative feelings are triggered** by the problem or situation: *"How does it make you feel when ______ [problem] occurs?"*

Use the **List of negative feelings** worksheet as a prompt, if necessary.

6) Explore the **caregiver's willingness and ability to help** with the problem or situation: *"Is this problem something you would be willing and/or able to help with, if necessary?"*

Step 2

Consider which of the strategies listed below could be used to **help the person with dementia feel better when this problem or situation occurs**.

1) SELECT THE BEST SITUATIONS:

i) Explore which **situations** that trigger negative emotions could be **avoided**.

ii) Explore which **situations** that trigger positive emotions could be **encouraged**.

Use the **Activities that I enjoy doing** worksheet as a prompt, if necessary.

2) CHANGE THE SITUATION:

i) Explore how **situations** that trigger negative emotions could be **changed through tools** to support memory, attention and planning (with or without caregiver support).

Use the **List of tools to support memory, attention and planning** worksheets 1 and 2 as a prompt, if necessary.

Use the **Activity plan - smaller steps** worksheet as a prompt, if necessary.

ii) Explore how **situations** that trigger negative emotions could be **changed through caregiver support**.

3) SHIFT ATTENTION & MANAGE EMOTIONS:

i) Explore how the person with dementia could **redirect their attention** when faced with the problem or situation.

Use the **List of strategies for distracting or soothing myself** worksheet as a prompt, if necessary.

ii) Explore how the **person with dementia could use self-soothing strategies** to manage their emotions.

Use the **List of strategies for distracting or soothing myself** worksheet as a prompt, if necessary.

iii) Explore how the **caregiver could use self-soothing strategies** to manage their emotions.

Use the **Information about looking after yourself as a caregiver** handout, if necessary.

4) CHANGE PERSPECTIVES:

i) Explore how the person with dementia could be helped to **think differently** about the situation (e.g. by focusing on what the person with dementia can do rather than what they can't do) or **look at it from a different perspective**.

ii) Explore how the caregiver could be helped to **think differently** about the situation or **look at it from a different perspective**.

Use the **Information about depression in people with dementia** handout, if necessary.

Step 3

1) **Develop a plan** for trying out strategies for decreasing negative emotions and increasing positive ones.

Briefly summarise the session and ask for feedback (5 mins)

Briefly summarise what was covered in the session: *"Today we discussed _______. How did you find the session? What did you find helpful? What was less helpful?"*

Set the home practice (5 mins)

i) **Set the home practice** (phrased as "*things to try out before the next session*" or whatever term they prefer):

a) **Home practice will vary** depending on how far you got with completing the steps for reducing negative emotions and increasing positive emotions. **Give a rationale** for whatever home practice you set.

b) Ask the person with dementia to engage in **at least two** **pleasurable activities** before the next session. **Give a rationale** for it (e.g. *"Doing things we enjoy can help improve how we're feeling"*). Give the **Calendar of activities** worksheet and explain how to complete it. Include the two planned pleasurable activities on this.

ii) Discuss **possible obstacles or barriers** to completing the home practice (e.g. *"What might stop you from trying out these things before the next session?", "What might get in the way?", "What could you do to ensure that you do them?", "Is this something that [name of caregiver] could help you with?"*).

iii) Discuss how **helpful the home practice might be** for them (e.g. *"How might it help if you try out these things before the next session?"*).

iv) <Give the **Things to try out before the next session** worksheet.> Help the person with dementia and/or caregiver to write a few notes about what was discussed in today's session and to make a note of the home practice. Ask the person with dementia and/or caregiver to make some brief notes in relation to completing the home practice (see the **Things to try out before the next session** worksheet for prompts). In particular, ask the person with dementia and/or caregiver to notice whether they feel better after completing the home practice.

Individual time (5 mins each)

Allow **individual time with the person with dementia and their caregiver** (with the person with dementia's consent) to discuss how they found the session, home practice and any issues raised in the session (5 mins each). If issues such as tension between the person with dementia and their caregiver are raised, then suggest that it would be helpful to bring this up in the next session as the problem to be addressed. Follow guidelines for working with tension in the subsequent session if the caregiver agrees to this.

After the session

Write some brief notes on **what worked well and any challenges** you faced so that you can bring this to supervision, if necessary.

[Return to Table of Contents]

LIST OF COMMON OBSTACLES OR BARRIERS

Here are some common obstacles or barriers that can get in the way of people doing things.

**Are any of these obstacles or barriers getting in the way of you doing things?**<br><br>*Put a tick in the box ☑ if the obstacle or barrier applies to you.*	
**Physical obstacles or barriers**	**Emotional obstacles or barriers**
☐ Feeling unwell	☐ Finding it difficult to feel pleasure anymore
☐ Being in pain	☐ Feeling unmotivated
☐ Feeling too tired	☐ Feeling irritated or frustrated
☐ Finding it difficult to move around or get out	☐ Feeling anxious or worried or lack of confidence
☐ Finding it difficult to hold or manipulate things	☐ Feeling like you’re a burden
☐ Finding it difficult to see or hear	☐ Feeling embarrassed or loss of self-esteem
Other:	Other:
**Mental obstacles or barriers**	**Relationship obstacles or barriers**
☐ Difficulties with memory	☐ Lost contact with others
☐ Difficulties with attention or concentration	☐ Disagreements with others
☐ Difficulties with planning or organising things	☐ Difficulties that other people are experiencing
☐ Difficulties with starting things or getting going	☐ Don’t know what to talk about
☐ Finding it difficult to read or write	☐ Others stopping you from doing things
Other:	Other:

LIST OF NEGATIVE FEELINGS

Some common negative feelings that people experience when they are faced with a difficult problem or situation are shown below.

How do you feel when you are faced with this difficult problem or situation?

Put a tick in the box ☑ next to all of the responses that apply to you when you are faced with this difficult problem or situation.

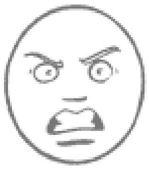	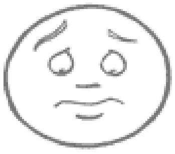	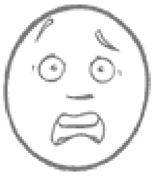	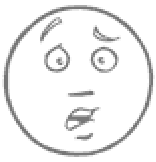	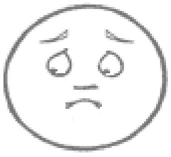
☐ Angry	☐ Miserable	☐ Scared	☐ Hurt	☐ Ashamed
☐ Annoyed	☐ Sad	☐ Anxious	☐ Betrayed	☐ Guilty
☐ Frustrated	☐ Lonely	☐ Worried	☐ Offended	☐ Regretful
☐ Irritated	☐ Discouraged	☐ Stressed	☐ Rejected	☐ Embarrassed
☐ Impatient	☐ Unmotivated	☐ Confused	☐ Disappointed	☐ Disgusted

Other (please specify):

ACTIVITIES THAT I ENJOY DOING

Some common activities that people enjoy doing are shown below. Which ones do you enjoy doing?

Put a tick in the box ☑ next to an activity if you think that you might enjoy it or have enjoyed it previously.

**Daily activities**	**Self-care & spiritual activities**	**Social activities**	**Leisure activities & hobbies**	**Outdoor & physical activities**
☐ Watching TV or listening to the radio	☐ Grooming (e.g. shaving, wearing makeup)	☐ Having people over for something to eat or drink	☐ Reading books, newspapers or magazines	☐ Going to a theatre, cinema, concert, gallery, museum
☐ Setting the table	☐ Wearing favourite clothes	☐ Spending time with family or friends	☐ Writing (e.g. letters, stories, poetry)	☐ Watching wildlife
☐ Cooking, preparing snacks or drinks	☐ Having a bath, shower or massage	☐ Chatting to family or friends (e.g. phone)	☐ Painting, drawing or doing crafts	☐ Going to watch sports (e.g. football game)
☐ Doing the cleaning or light housework	☐ Taking care of oneself (e.g. eating healthily, taking medication)	☐ Having meals or drinks with family or friends	☐ Knitting, crocheting or sewing	☐ Going on outings (e.g. park, picnic, shopping for leisure)
☐ Doing the laundry or ironing	☐ Going to the barber or hairdresser	☐ Discussing photos of family or friends	☐ Doing crosswords or puzzles	☐ Gardening
☐ Doing the dishes	☐ Going to a spiritual gathering (e.g. church) or retreat	☐ Recalling and discussing past events with others	☐ Listening to music, singing or playing a musical instrument	☐ Doing exercise (e.g. walking, swimming, cycling, gym)
☐ Doing the shopping	☐ Reading a spiritual text or praying	☐ Meeting new people	☐ Watching sports on TV (e.g. tennis)	☐ Going dancing
☐ Watching, listening to or reading about the daily news	☐ Practising meditation or relaxation	☐ Going to a social group or gathering	☐ Playing games (e.g. board games, computer games)	☐ Doing yoga, pilates or Tai Chi
Other:	Other:	Other:	Other:	Other:

LIST OF TOOLS TO SUPPORT MEMORY, ATTENTION AND PLANNING - WORKSHEET 1

Some tools and strategies that can be used to help support your memory, attention and planning abilities are listed below.

These tools do not take away the difficulties you may be experiencing, but they can help to reduce the negative impact they have on your day-to-day life.

In turn, this may reduce any distress caused by these difficulties.

An example of how the tools and strategies can be used to manage difficulties with remembering how and what to do when cooking a stew is also shown.

**Tools and strategies to help you remember things**	**Example: Tools that you could use to remember things when cooking a stew**
Memory aids that you can see:<br><br>• Calendars and diaries<br>• Clocks with day, date, month, and year<br>• Coloured tags<br>• Daily checklists<br>• Medication kit/blister packs/dosette box<br>• Notebooks<br>• Signs and pictures<br>• Sticky notepapers (e.g. post-it notes)<br>• Weekly planner<br>• Whiteboard<br><br>Memory aids that you can hear:<br><br>• Alarms and alarm clocks<br>• Computerised phone calls<br>• Customised audiotapes & pre-recorded messages<br>• Timers	1. Put signs/pictures on cupboards that show where ingredients are stored.<br><br>2. Put signs/pictures on cupboards that show where cooking equipment is stored.<br><br>3. Set a voice alarm or a standard alarm with a post-it note that says “Time to check what ingredients you need for cooking a stew”. The caregiver could also give this prompt.<br><br>4. If you need to go out shopping for the ingredients, write in the diary or on a calendar when you plan to do this.<br><br>5. If you need to go out shopping for the ingredients, set an alarm with a post-it note that says “Time to go out shopping for ingredients”.<br><br>6. Set an alarm with a post-it note that says “Time to start preparing for cooking a meal”.<br><br>7. Put a sign by the stove that says "Get your 'Cooking a meal' folder".<br><br>8. Set an alarm with a post-it note that says “Time to start cooking a stew”.

LIST OF TOOLS TO SUPPORT MEMORY, ATTENTION AND PLANNING - WORKSHEET 1 (continued)

Tools and strategies to help you pay attention	Example: Strategies that you could use to remember things when cooking a stew
Reduce distractions in the home environment	1. Reduce clutter in the kitchen.<br><br>2. Turn off the TV or radio when cooking.<br><br>3. Cook when you are least likely to be disturbed.

Tools and strategies to help you plan tasks	Example: Strategies that you could use to remember things when cooking a stew
Think about the things you might need to do before completing a task	1. Think about what you will need to do before cooking a stew:<br><br>a. Find a recipe for the stew.<br><br>b. Get all the ingredients and put them in a specific place.<br><br>c. Get all the equipment needed to make the stew.<br><br>d. Get the instructions for using the stove timer.
Break a task down into smaller steps	1. Break the task of 'cooking a stew' down into smaller steps:<br><br>a. Measure out all the ingredients.<br><br>b. Prepare all the ingredients (e.g. chop vegetables).<br><br>c. Put all the ingredients into the pan in the right order and right amount (e.g. following the recipe).<br><br>d. Set the stove timer.
Keep instructions and checklists in labelled folders	1. Label a folder "Cooking a meal".<br><br>2. Keep a checklist for cooking a meal, instructions for using the stove timer and favourite recipes in it.

LIST OF TOOLS TO SUPPORT MEMORY, ATTENTION AND PLANNING - WORKSHEET 2

Put a tick in the box ☑ if you think a tool or strategy would be helpful for you.

Memory aids - things that you can see:

- ☐ Calendars
- ☐ Clocks with day, date, month, and year
- ☐ Coloured tags
- ☐ Daily checklists
- ☐ Diaries or weekly planners
- ☐ Magnetic notepads
- ☐ Markers
- ☐ Medication kit/blister packs/dosette box
- ☐ Notebooks
- ☐ Pictures and/or signs (black and white signs, coloured signs)
- ☐ Sticky notepapers and reminders (e.g. post-it notes)
- ☐ Whiteboard

Memory aids - things that you can hear:

- ☐ Alarms and alarm clocks (with clear signs to show what the alarm is a reminder of)
- ☐ Computerised phone calls
- ☐ Customised audiotapes
- ☐ Key-chain recorder
- ☐ Timers (with clear signs to show what the timer is a reminder of)
- ☐ Timed pre-recorded messages
- ☐ Voice alarms

Strategies to help attention

- ☐ Reduce distractions

Strategies to help planning

- ☐ Think about the things you might need to do before completing a task
- ☐ Break a task down into smaller steps
- ☐ Keep instructions and checklists in labelled folders

ACTIVITY PLAN - SMALLER STEPS

Breaking an activity down into smaller steps can make it feel less overwhelming and can help you to make sure that you don't miss out important steps. An example of putting this strategy into practice is presented below:

What is the activity that you would like to do?

EXAMPLE: Go to the Day Centre

**What smaller steps do you need to take to complete this activity?** Write these in the boxes below.	**Tick (✓) when the step is completed**
Get up at 08:30	✓
Shower at 08:45	✓
Get ready and dress at 09:00	✓
Call for a taxi at 09:15 (0785 478 3269)	✓
Get the taxi to go to the Day Centre at 10:00	✓

What is the activity that you would like to do? Write it in the space below:

**What smaller steps do you need to take to complete this activity?** Write these in the boxes below.	**Tick (✓) when the step is completed**

LIST OF STRATEGIES FOR DISTRACTING OR SOOTHING MYSELF

Some common ways to distract yourself or make yourself feel better when you are feeling distressed (e.g. sad, anxious, embarrassed, worried, frustrated, hopeless) are listed below.

**Put a tick in the box ☑ if you think one of the following strategies might be helpful for distracting yourself or making yourself feel better when you are distressed.**	
Physical strategies	*Creative strategies*
☐ Go outside (e.g. garden or park)	☐ Do something artistic - drawing, painting or colouring
☐ Squeeze a stress ball	☐ Listen to music
☐ Do some cleaning	☐ Play a musical instrument
☐ Do some exercise (e.g. go for a walk)	☐ Sing a song
Other:	Other:
Productive strategies	*Soothing strategies*
☐ Read a book, newspaper or magazine	☐ Take a long bath/shower or have a massage
☐ Do a jigsaw or puzzle	☐ Watch your favourite film or TV show
☐ Do a crossword or word search	☐ Have some comforting food or drink
☐ Do the washing up	☐ Call a friend or family member
Other:	Other:

INFORMATION ABOUT LOOKING AFTER YOURSELF AS A CAREGIVER

Information about looking after yourself as a caregiver can be found on the Alzheimer's Society website (https://www.alzheimers.org.uk/get-support/help-dementia-care/looking-after-yourself#content-start). A summary of this is presented here:

As a caregiver for a person living with dementia, looking after yourself – and learning to deal with challenges – means you will be in a better position to continue to care for the affected person. Keeping yourself healthy – physically, mentally and emotionally – can also help you to have a better relationship with them.

As a caregiver, you may experience a range of difficult emotions. You may not have the time to do all the things you need to do, and this can be difficult too. The needs of the person you care for will often come before your own, and this can mean that you do not look after yourself properly. You might feel that it is not always possible to make time for yourself, but it is important for both you and the person with dementia.

The information below is for caregivers. It explains some of the challenges that you may face when caring for a person with dementia and how best to cope with them. It offers tips and advice on how to look after yourself, and tells you what help and support is available.

Your health and wellbeing

Caring can have a big impact on your mental and physical health and wellbeing. It is important to look after yourself well so that you can continue to care for the person with dementia.

- Try to eat a well-balanced diet, with at least five portions of fruit and vegetables every day. A healthy diet will be beneficial for the person you care for too.
- Taking regular exercise is good for your health – both physical and mental. You could try going for a walk or taking up an exercise class, or doing a crossword for example. Whatever you choose should be fun and something that you want to do.
- Spending time enjoying your hobbies and interests is good for your mental and physical health.
- Try to get enough sleep. Sleep is very important as it helps the brain and body recover from fatigue. It can be difficult if the person you care for has disturbed nights. You may find it easier to sleep when the person you care for is sleeping, and may be able to take advantage of daytime naps. If you are unable to get enough sleep, talk to your GP. They may be able to suggest services or techniques that can help.
- If you have a physical disability or a sensory impairment, these will affect your caring role. It is important to make sure you are getting all the support you are entitled to. Speak to your GP or social services department.
- If you have to help the person you care for to move, be careful to look after your back. Speak to your GP for advice. They may be able to refer you to a physiotherapist or an occupational therapist. Some local carers' organisations provide training sessions on moving and handling. Contact your local carers' organisation, GP or social services to find out what is available in your area.

- See your GP on a regular basis to check up on your own health. You may also want to be put on the Carers' Register. This is a list of patients who are caregivers – which GPs have been asked to keep – to help support them and the people they care for. Speak to your GP for more information or to register.
- If you are struggling to cope or feeling depressed, anxious or stressed, talk to your GP. There is help available, such as counselling or extra support services. These problems are easier to manage if you do something about them at an early stage.
- Consider using technology to help you in your caring role. This could include using the internet to help with online banking to pay bills, or shopping online to buy groceries. You may also want to think about electronic devices, known as 'assistive technology', such as gas monitors or locator devices.

Taking a break

It can be difficult to find time for yourself when you are caring for a person with dementia. You may feel guilty about wanting time alone, but it is important for your own wellbeing. When you do manage to get time to yourself, you may want to use it to catch up with other tasks such as housework or managing finances. However, taking breaks and continuing to do things that you enjoy can help you manage your caring role. This could include having some 'time out' during the day to do a crossword or go for a coffee.

Many people find that taking the time to pursue things they enjoy helps them with their caring role. By taking regular breaks you may find yourself better able to support yourself and the person you care for. Time apart can also be good for both of you and can make you both feel better.

You don't have to take long breaks from caring, but a short time to enjoy yourself could make a lot of difference. Try to make time for something you enjoy every day, whether it is on your own or with the person you care for. By having a break, the person with dementia may also get to experience new things and have a change from their routine. Types of break might include:

- taking the time to sit down and have a cup of tea, read the paper, listen to music, or go for a walk
- going out for a coffee or drink
- meeting a friend or going shopping
- pursuing interests, hobbies and activities that you find enjoyable
- having a short holiday, whether it is a few days or a week.

Some of these activities may help you feel less isolated as well. You may be able to take a break if someone you know, such as a friend or family member, can spend a few hours with the person you care for. If not, your local authority may be able to help. Alternatively, you may have to find a local organisation or charity who offer this service. Providing care so that you can take a break is called 'respite care', or sometimes 'replacement care'. It could be for an hour or two, or for days or weeks.

INFORMATION ABOUT DEPRESSION IN PEOPLE WITH DEMENTIA

Information about depression in people with dementia can be found on the Alzheimer's Society website (https://www.alzheimers.org.uk/about-dementia/symptoms-and-diagnosis/depression). A summary of this is presented here:

What is depression and how common is it in people with dementia?

A number of feelings, such as sadness and hopelessness, dominate a person's life and make it difficult for them to cope.

People with depression may also experience physical symptoms, such as loss of energy and appetite changes. Physical symptoms of depression are more common in older people with the condition.

At least one in five people in the UK will experience depression at some time in their lives. It is more common among people with dementia (20–40% of whom may have depression), particularly those who have vascular dementia or Parkinson's disease dementia.

Depression is often diagnosed in the early stages of dementia, but it may come and go, and may be present at any stage. Depression is also common among family caregivers supporting a person with dementia.

What are the symptoms of depression?

Depression affects people in different ways and to different degrees. Doctors may talk about mild, moderate and severe depression.

Some of the more common symptoms include:

- a sad, hopeless or irritable mood for much of the time
- a loss of interest or pleasure in activities that were once enjoyed
- feelings of low self-esteem, worthlessness or undue guilt
- feelings of isolation and of being cut off from other people
- sleep disturbance, such as early morning waking
- problems with remembering, concentrating or making simple decisions
- increased agitation and restlessness
- tiredness or loss of energy
- eating too little or too much, with weight loss or gain
- aches and pains that appear to have no physical cause
- thoughts of death and suicide.

Some of these symptoms (such as problems with memory or concentrating, and withdrawal) are similar to those experienced by people with dementia. This is why assessment of someone for possible dementia will usually include ruling out depression first, in case depression alone, rather than dementia, is causing their symptoms.

Difficulties of depression and dementia

A person with both dementia and depression will be struggling with two lots of difficulties. They may find it even harder to remember things and may be more confused or withdrawn. Depression may also make behavioural changes worse in people with dementia, causing aggression, problems sleeping or refusal to eat. In the later stages of dementia, depression tends to show itself in the form of depressive 'signs', such as tearfulness and weight loss.

Tips for caregivers

Someone who is feeling depressed or anxious will often find the following helpful:

- Talking about their feelings – if someone is feeling depressed or anxious, or something very upsetting or traumatic has happened to them, they may find it helpful to talk to someone close to them about it. (Patience and understanding will be more helpful than trying to get the person to 'cheer up'.)
- Support to help them maintain social contact with other people – this will help them to feel less isolated.
- Persevering with treatment – those close to the person should encourage them to keep taking their medication or seeing their therapist even if improvement feels slow at the start.
- Keeping active – physical exercise is good for relieving feelings of anxiety and depression, and can also help people with sleep problems and apathy. Supporting the person to do other activities that they enjoy will often also help.
- Eating a healthy diet – a poor diet can contribute to feelings of anxiety and depression, as can alcohol and caffeine. It is therefore a good idea to try to eat a healthy diet and not drink too much alcohol or caffeinated drinks.

Consulting the doctor

It is important to see the doctor if a person with dementia is behaving in an unusual or worrying way, or has deteriorated more rapidly than expected. These changes could be caused by apathy, depression or anxiety, or could be due to an illness or the effects of medication.

In order to diagnose apathy, anxiety or depression, the doctor will talk to the person with dementia and their caregiver. They will try to assess the person's behaviour, mood and any changes that have occurred (for example, have they become more agitated or do they have less energy?).

It can be difficult for a doctor to diagnose depression in a person with dementia because the symptoms of depression and dementia are so similar. Symptoms of apathy and depression can also overlap.

CALENDAR OF ACTIVITIES

1. Write down in a few words the pleasurable activity that you will do over the next week.<br><br>2. After you have completed the activity, rate how much you enjoyed this activity from 1 (not at all) to 6 (very much) using the rating scale on the right.	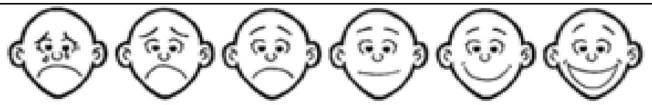<br>1 2 3 4 5 6<br>Not at all Very much

**Mon AM**	**Tues AM**	**Wed AM**	**Thu AM**	**Fri AM**	**Sat AM**	**Sun AM**
e.g. Go shopping 5	*e.g. Go to the Lunch Club 4*		*e.g. Go to the Lunch Club 5*		*e.g. Go to the cinema 6*	
**Mon PM**	**Tues PM**	**Wed PM**	**Thu PM**	**Fri PM**	**Sat PM**	**Sun PM**
		e.g. Coffee with friend 5		*e.g. Go for a walk 4*		*e.g. Dinner with family 6*

THINGS TO TRY OUT BEFORE THE NEXT SESSION

Summary of today's session

What we discussed:

Home practice

What we agreed to try out before the next session:

1.

2.

3.

4.

THINGS TO TRY OUT BEFORE THE NEXT SESSION (continued)

Experience of home practice

If you have been able to complete what you agreed to try out before the next session, then please write down some brief notes about it:

How did it go?

How did you feel afterwards?

What was helpful?

What was not helpful?

If you come to the clinic for your sessions, please don't forget to bring your PATHFINDER materials with you to the next session.

5.1.4 Session 8 - Review

At the start of the session

1) Make sure you have all the **relevant equipment** with you (relevant worksheets, pens, encrypted digital voice recorder, a watch so that you can keep track of time).

2) Introduce yourself and show your ID badge. Explain that you are their therapist in the PATHFINDER study.

3) Ensure that the person with dementia and and/or their caregiver have their **glasses or hearing aid**, if needed.

4) **Minimise distractions** (e.g. ask for the TV/radio to be turned off, see if noisy pets can be put in a separate room).

5) Try to ensure that the **seating arrangement** allows the therapist to sit closer to the person with dementia than the caregiver.

6) Ask permission to use the **digital voice recorder** (e.g. *"We'll be recording the session just so that my supervisor can check that I am facilitating these sessions as best as I can. They won't be focusing on what you're saying or doing - they will be focusing on what I am saying. Is that OK?"*). If the person with dementia and and/or their caregiver refuse to allow the session to be recorded then proceed with the session, make a note of why the session was not recorded in the study files, and discuss with your supervisor, if necessary.

7) Ask the person with dementia if they are **happy for their caregiver to be present** (e.g. *"Are you happy for [name of caregiver] to join us for this session, and to answer any questions that you or I might have?"*).

8) Remind the person with dementia that, with their consent, you will be having a **short discussion (5 mins) with the caregiver** after the session to discuss home practice (e.g. *"Would it be OK if I have short chat with [name of caregiver] after the session ends so that we can discuss any preparations that are needed for the next session?"*).

9) Discuss whether the person with dementia is having a **'good day' or a 'bad day'** with respect to feeling confused, muddled or low in mood (e.g. *"Before we begin, we know that people can have good days and bad days with respect to feeling confused, muddled or low in mood… it's helpful for me to know what kind of day you're having so that I don't go too quickly with things. Would you say this is a good day or a bad day for you?"*). Use this information to pace the session appropriately. Try not to get caught up in lengthy discussions about this.

10) Assess risk (e.g. *"I now need to ask you a question about your mood that we ask everybody in the study in case they need more support than we are currently offering. Have you been feeling so bad that you have had thoughts about hurting yourself or others, that life is not worth living or that you'd be better off dead?").* If this was reported in a previous session then ask: *"You previously mentioned X [describe what was previously reported with respect to suicidal ideation, plans, intent, protective factors] - has anything changed since then?"* If this has not been reported in a previous session, then assess further (see Appendix 8, if necessary).

11) Check that the person with dementia is **willing to participate** further in the session (e.g. *"Are you happy for us to continue with the session?"*).

12) Explain which **session you are on** (e.g. *"We're on session 8 out of 8 sessions. To recap, the aim of these sessions is to help you feel better by finding ways of reducing negative feelings and increasing positive feelings in your daily lives."*).

13) Briefly discuss the **agenda for today's session** (e.g. *"In today's session, I'm going to recap on what we discussed in the previous session, and we're going to review how you got on with the home practice. We're then going to review what we've discussed in these sessions in order to develop your own personalised summary of therapy. At the end, I will summarise what we have discussed in the session, and what things you can try out in between now and the next session. Finally, I will have a brief chat with each of you to discuss how you found the session. How does that sound?").*

14) Discuss the possible **need for interruptions** (e.g. *"At times I might need to interrupt you so that I can make sure we keep on track with what we've got planned for today's session and so that you get the most out of the session. Is that OK?"*). Politely interrupt when you notice yourselves going off topic (e.g. *"I'm really sorry to interrupt you…*

I'm just aware of the time and want you to get the most out of the session. Could we come back to…?").

Briefly review previous session and home practice (5 mins)

Review what was discussed in the **previous session**: *"In the previous session we discussed _______ [describe what was discussed in the previous session]."*

Review the **home practice**: *"The things to try out before today's session were to _______ [describe home practice]. Did you manage to do this? What was helpful? What was not helpful? "*

If no, then ask: *"What got in the way?"* or *"Was there a particular reason you didn't get round to doing it?* If the home practice has not been completed then consider re-setting it as home practice (and discussing ways of overcoming the barriers to completing it) or completing it in the session.

Positively reinforce any home practice that is completed by bringing attention to any positive benefits of completing it. For example, *"What happened after you did ______?"* (Type A prompt) or *"As we talk about this, how does it feel, right now, that you were able to do ______?"* (Type B prompt). Highlight any **positive or desirable consequences** of completing the home practice.

Review which problems and negative emotions were addressed, what tools to support memory, attention and planning were utilised, and what emotion regulation strategies were used (35 mins)

<Complete the **Summary of the sessions** worksheet to facilitate writing of the personalised treatment summary.>

Explain that a **personalised summary of therapy** will be sent to the person with dementia and their caregiver within the next 1-2 weeks (see Appendix 10 and 11 for examples of these).

<Complete version A (for people with milder dementia) or version B (for people with more moderate dementia) of the blank **personalised summary of therapy** after the session.>

Briefly summarise the session and ask for feedback (5 mins)

Briefly summarise what was covered in the session: *"Today we discussed _______. How did you find the session? What did you find helpful? What was less helpful?"*

Set the home practice and discuss the next booster session (5 mins)

i) **Set the home practice** (phrased as "*things to try out before the next session*" or whatever term they prefer):

a) **Home practice will vary** depending on what is discussed in the session. **Give a rationale** for whatever home practice you set.

b) Ask the person with dementia to engage in **at least two pleasurable activities** on a weekly basis before the booster session. **Give a rationale** for it (e.g. *"Doing things we enjoy can help improve how we're feeling"*). Give the **Calendar of activities** worksheet and explain how to complete it.

ii) Discuss **possible obstacles or barriers** to completing the home practice (e.g. *"What might stop you from trying out these things before the next session?", "What might get in the way?", "What could you do to ensure that you do them?", "Is this something that [name of caregiver] could help you with?"*).

iii) Discuss how **helpful the home practice might be** for them (e.g. *"How might it help if you try out these things before the next session?"*).

iv) <Give the **Things to try out before the next session** worksheet.> Help the person with dementia and/or caregiver to write a few notes about what was discussed in today's session and to make a note of the home practice. Ask the

person with dementia and/or caregiver to make some brief notes in relation to completing the home practice (see the **Things to try out before the next session** worksheet for prompts). In particular, ask the person with dementia and/or caregiver to notice whether they feel better after completing the home practice.

v) Discuss the **booster session**: *"We will arrange to see you for a booster session on [date corresponding to 6 months after the start of therapy]. In this session we'll recap on what we've discussed in these sessions and explore how you can continue to use the techniques to help you feel better. Is that OK?"*

<u>Individual time (5 mins each)</u>

Allow **individual time with the person with dementia and their caregiver** (with the person with dementia's consent) to discuss how they found the session, home practice and any issues raised in the session (5 mins each). If issues such as tension between the person with dementia and their caregiver are raised, then suggest that it would be helpful to bring this up in the next session as the problem to be addressed. Follow guidelines for working with tension in the subsequent session if the caregiver agrees to this.

<u>After the session</u>

Write some brief notes on **what worked well and any challenges** you faced so that you can bring this to supervision, if necessary.

[Return to Table of Contents]

<u>SUMMARY OF THE SESSIONS (for therapists)</u>

Session Number: ___________ *Patient ID:* ___________ *Date:* _________________

For each problem consider which of the following strategies/tools helped:
i) Tools to support memory, attention and planning;
ii) Involving the caregiver;
iii) Strategies for distracting or soothing oneself;
iv) Thinking differently about things.

Problem 1:

**"A problem, difficulty, situation or concern we addressed was________."**	**"The negative feelings we addressed were ______."**	**"The strategies and tools we used to...**
		**...reduce negative feelings were_________.":**<br><br>**...increase positive feelings were_________.":**<br><br>**...overcome any limitations were_________.":**

Problem 2:

**"A problem, difficulty, situation or concern we addressed was________."**	**"The negative feelings we addressed were ______."**	**"The strategies and tools we used to...**
		**...reduce negative feelings were_________.":**<br><br>**...increase positive feelings were_________.":**<br><br>**...overcome any limitations were_________.":**

<u>SUMMARY OF THE SESSIONS (for therapists - continued)</u>

For each problem consider which of the following strategies/tools helped:
i) Tools to support memory, attention and planning;
ii) Involving the caregiver;
iii) Strategies for distracting or soothing oneself;
iv) Thinking differently about things.

Problem 3:

**"A problem, difficulty, situation or concern we addressed was________."**	**"The negative feelings we addressed were ______."**	**"The strategies and tools we used to...**
		**...reduce negative feelings were_________.":**<br><br>**...increase positive feelings were_________.":**<br><br>**...overcome any limitations were_________.":**

Problem 4:

**"A problem, difficulty, situation or concern we addressed was________."**	**"The negative feelings we addressed were ______."**	**"The strategies and tools we used to...**
		**...reduce negative feelings were_________.":**<br><br>**...increase positive feelings were_________.":**<br><br>**...overcome any limitations were_________.":**

MY SUMMARY OF THERAPY – Problem _____ (version A)

My problem or situation that was making me feel distressed (the triggers):

Negative feelings that were associated with this problem or situation:

Strategies that I found helpful for reducing negative feelings & increasing positive ones:

What I need to remember:

MY SUMMARY OF THERAPY – PROBLEM _____ (version B)

My problems or situations that were making me feel distressed (the triggers)

→

Negative feelings that were associated with these problems or situations

→

Strategies that I found helpful for reducing negative feelings and increasing positive ones

CALENDAR OF ACTIVITIES

1. Write down in a few words the pleasurable activity that you will do over the next week.<br><br>2. After you have completed the activity, rate how much you enjoyed this activity from 1 (not at all) to 6 (very much) using the rating scale on the right.	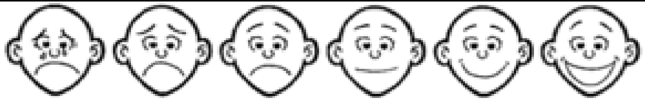<br>1 2 3 4 5 6<br>Not at all Very much

**Mon AM**	**Tues AM**	**Wed AM**	**Thu AM**	**Fri AM**	**Sat AM**	**Sun AM**
e.g. Go shopping 5	*e.g. Go to the Lunch Club 4*		*e.g. Go to the Lunch Club 5*		*e.g. Go to the cinema 6*	
**Mon PM**	**Tues PM**	**Wed PM**	**Thu PM**	**Fri PM**	**Sat PM**	**Sun PM**
		e.g. Coffee with friend 5		*e.g. Go for a walk 4*		*e.g. Dinner with family 6*

THINGS TO TRY OUT BEFORE THE NEXT SESSION

<u>Summary of today's session</u>

What we discussed:

<u>Home practice</u>

What we agreed to try out before the next session:

1.

2.

3.

4.

THINGS TO TRY OUT BEFORE THE NEXT SESSION (continued)

Experience of home practice

If you have been able to complete what you agreed to try out before the next session, then please write down some brief notes about it:

How did it go?

How did you feel afterwards?

What was helpful?

What was not helpful?

If you come to the clinic for your sessions, please don't forget to bring your PATHFINDER materials with you to the next session.

5.1.5 Top-up sessions 1 & 2 - Recap and review

At the start of the session

1) Make sure you have all the **relevant equipment** with you (relevant worksheets, pens, encrypted digital voice recorder, a watch so that you can keep track of time, personalised written summary from Session 8).

2) Introduce yourself and show your ID badge. Explain that you are their therapist in the PATHFINDER study.

3) Ensure that the person with dementia and and/or their caregiver have their **glasses or hearing aid**, if needed.

4) **Minimise distractions** (e.g. ask for the TV/radio to be turned off, see if noisy pets can be put in a separate room).

5) Try to ensure that the **seating arrangement** allows the therapist to sit closer to the person with dementia than the caregiver.

6) Ask permission to use the **digital voice recorder** (e.g. *"We'll be recording the session just so that my supervisor can check that I am facilitating these sessions as best as I can. They won't be focusing on what you're saying or doing - they will be focusing on what I am saying. Is that OK?"*). If the person with dementia and and/or their caregiver refuse to allow the session to be recorded then proceed with the session, make a note of why the session was not recorded in the study files, and discuss with your supervisor, if necessary.

7) Ask the person with dementia if they are **happy for their caregiver to be present** (e.g. *"Are you happy for [name of caregiver] to join us for this session, and to answer any questions that you or I might have?"*).

8) Remind the person with dementia that, with their consent, you will be having a **short discussion (5 mins) with the caregive**r after the session to discuss home practice (e.g. *"Would it be OK if I have short chat with [name of caregiver] after the session ends so that we can discuss any preparations that are needed for the next session?"*).

9) Discuss whether the person with dementia is having a **'good day' or a 'bad day'** with respect to feeling confused, muddled or low in mood (e.g. *"Before we begin, we know that people can have good days and bad days with respect to feeling confused, muddled or low in mood… it's helpful for me to know what kind of day you're having so that I don't go too quickly with things. Would you say this is a good day or a bad day for you?"*). Use this information to pace the session appropriately. Try not to get caught up in lengthy discussions about this.

10) **Assess risk** (e.g. *"I now need to ask you a question about your mood that we ask everybody in the study in case they need more support than we are currently offering. Have you been feeling so bad that you have had thoughts about hurting yourself or others, that life is not worth living or that you'd be better off dead?").* If this was reported in a previous session then ask: *"You previously mentioned X [describe what was previously reported with respect to suicidal ideation, plans, intent, protective factors] - has anything changed since then?"* If this has not been reported in a previous session, then assess further (see Appendix 8, if necessary).

11) Check that the person with dementia is **willing to participate** further in the session (e.g. *"Are you happy for us to continue with the session?").*

12) Explain which **session you are on** (e.g. *"We're on top-up session _____. To recap, the aim of these sessions is to help you feel better by finding ways of reducing negative feelings and increasing positive feelings in your daily lives."*).

13) Briefly discuss the **agenda for today's session** (e.g. *"In today's session, I'm going to recap on what we discussed in the previous session, and we're going to review how you got on with the home practice. We're then going to recap on what PATH is and review your personalised summary of therapy to see if there's anything we need to change. At the end, I will summarise what we have discussed in the session. Finally, I will have a brief chat with each of you to discuss how you found the session. How does that sound?").*

14) Discuss the possible **need for interruptions** (e.g. *"At times I might need to interrupt you so that I can make sure we keep on track with what we've got planned for today's session and so that you get the most out of the session. Is that OK?"*). Politely interrupt when you notice yourselves going off topic (e.g. *"I'm really sorry to interrupt you… I'm just aware of the time and want you to get the most out of the session. Could we come back to…?").*

Briefly review previous session (5 mins)

Review what was discussed in the **previous session**: *"In the previous session we discussed _______ [describe what was discussed in the previous session]."*

Review the **home practice**: *"The things to try out before today's session were to _______ [describe home practice]. Did you manage to do this? What was helpful? What was not helpful?"*

If no, then ask: *"What got in the way?"* or *"Was there a particular reason you didn't get round to doing it?* If the home practice has not been completed then consider re-setting it as home practice (and discussing ways of overcoming the barriers to completing it) or completing it in the session.

Positively reinforce any home practice that is completed by bringing attention to any positive benefits of completing it. For example, *"What happened after you did ______?"* (Type A prompt) or *"As we talk about this, how does it feel, right now, that you were able to do ______?"* (Type B prompt). Highlight any **positive or desirable consequences** of completing the home practice.

Recap on PATH (5 mins)

<Give **Information about Problem Adaptation Therapy** worksheet.>

"As discussed in the first session, Problem Adaptation Therapy (or PATH) is a talking therapy that targets low mood and other negative feelings, as well as difficulties with functioning in day-to-day life. Research has shown that day-to-day problems and situations may contribute to low mood and other negative feelings such as anxiety or frustration. So it is important that we try to reduce these negative feelings in order to help you feel better."

"Therefore, in this therapy we identified problems and situations that triggered negative feelings for you." <Point to Step 1 in Figure 1.>

"And then we explored different ways of reducing these negative feelings or reducing the impact they had on your life. We also explored ways of increasing positive feelings." <Point to Step 2 in Figure 1.>

"We then put a plan together for testing out which of these ways of helping you to feel better worked best for you." <Point to Step 3 in Figure 1.>

"Do you have any questions about this?"

Review and revise (if necessary) the personalised written summary developed in the previous session (25 mins)

1) **Review the personalised treatment summary** of the sessions: *"We were able to identify a number of strategies for feeling better that worked best for you. We summarised these in our last session together. Here is that summary."*

2) **Check whether any changes need to be made** to the personalised treatment summary: *"How are these strategies continuing to work for you?" "How helpful are they are?"*

If prompts are needed, ask:

"How helpful have the tools for supporting memory, attention and planning been? Do any changes need to be made to this?" If yes, review the **List of tools to support memory, attention and planning** worksheet and/or the **Activity plan - smaller steps** worksheet.

"How helpful has it been to have [name of caregiver] supporting you? Do any changes need to be made to this?"

"How helpful have the strategies for distracting or soothing yourself been? Do any changes need to be made to this?" If yes, review the **List of strategies for distracting or soothing myself** worksheet.

"How helpful have the strategies for thinking differently about the situation been? Do any changes need to be made to this?"

"Are you still doing things that you enjoy?" If not, review the **Activities that I enjoy doing** worksheet.

3) Check whether any **new problems or situations** need to be considered in the session: *"Have any new problems or situations arisen since our last session together?" "How might we use these strategies to help make you feel better?"*

<u>Briefly summarise the session and ask for feedback (5 mins)</u>

Briefly summarise what was covered in the session: *"Today we discussed* **_______**. *How did you find the session? What did you find helpful? What was less helpful?"*

<u>Set home practice (5 mins)</u>

i) **Set the home practice** (phrased as "*things to try out before the next session*" or whatever term they prefer):

a) **Home practice will vary** depending on what is discussed in the session. **Give a rationale** for whatever home practice you set.

b) Ask the person with dementia to engage in **<u>at least two</u> pleasurable activities** on a weekly basis before the booster session. **Give a rationale** for it (e.g. *"Doing things we enjoy can help improve how we're feeling"*). Give the **Calendar of activities** worksheet and explain how to complete it.

ii) Discuss **possible obstacles or barriers** to completing the home practice (e.g. *"What might stop you from trying out these things before the next session?", "What might get in the way?", "What could you do to ensure that you do them?", "Is this something that [name of caregiver] could help you with?"*).

iii) Discuss how **helpful the home practice might be** for them (e.g. *"How might it help if you try out these things before the next session?"*).

iv) <Give the **Things to try out before the next session** worksheet.> Help the person with dementia and/or caregiver to write a few notes about what was discussed in today's session and to make a note of the home practice. Ask the person with dementia and/or caregiver to make some brief notes in relation to completing the home practice (see the **Things to try out before the next session** worksheet for prompts). In particular, ask the person with dementia and/or caregiver to notice whether they feel better after completing the home practice.

<u>Discuss the next booster session or the ending of sessions</u>

If on Top-up session 1 - **Discuss next booster session**: *"We will arrange to see you for a booster session on [date corresponding to 9 months after the start of therapy]. In this session we'll recap on what we've discussed in these sessions and explore how you can continue to use the techniques to help you feel better. Is that OK?"*

OR if on Top-up session 2 - **Discuss ending of the sessions**: Share your experience of working with the person with dementia and their caregiver, and thank them for participating in the sessions. Also, discuss getting help in the future.

<u>Individual time (5 mins each)</u>

Allow **individual time with the person with dementia and their caregiver** (with the person with dementia's

consent) to discuss how they found the session, home practice and any issues raised in the session (5 mins each). If issues such as tension between the person with dementia and their caregiver are raised, then suggest that it would be helpful to bring this up in the next session as the problem to be addressed. Follow guidelines for working with tension in the subsequent session if the caregiver agrees to this.

After the session

Write some brief notes on **what worked well and any challenges** you faced so that you can bring this to supervision, if necessary.

[Return to Table of Contents]

INFORMATION ABOUT PROBLEM ADAPTATION THERAPY (version A)

What is Problem Adaptation Therapy?

Problem Adaptation Therapy (PATH) is a new form of talking therapy for people with mild to moderate dementia who are experiencing difficulties with low mood. It was originally developed in the USA and is now being tested in the UK with people with mild to moderate dementia who are experiencing low mood.

What are the goals of PATH?

PATH aims to help improve people's mood by finding ways of reducing negative feelings (such as sadness or frustration) and increasing positive feelings (such as happiness or contentment) in their daily lives. A plan is then put together to test out whether these ways of reducing negative feelings and increasing positive ones help you to feel better (see Figure 1).

Figure 1: Problem Adaptation Therapy (PATH)

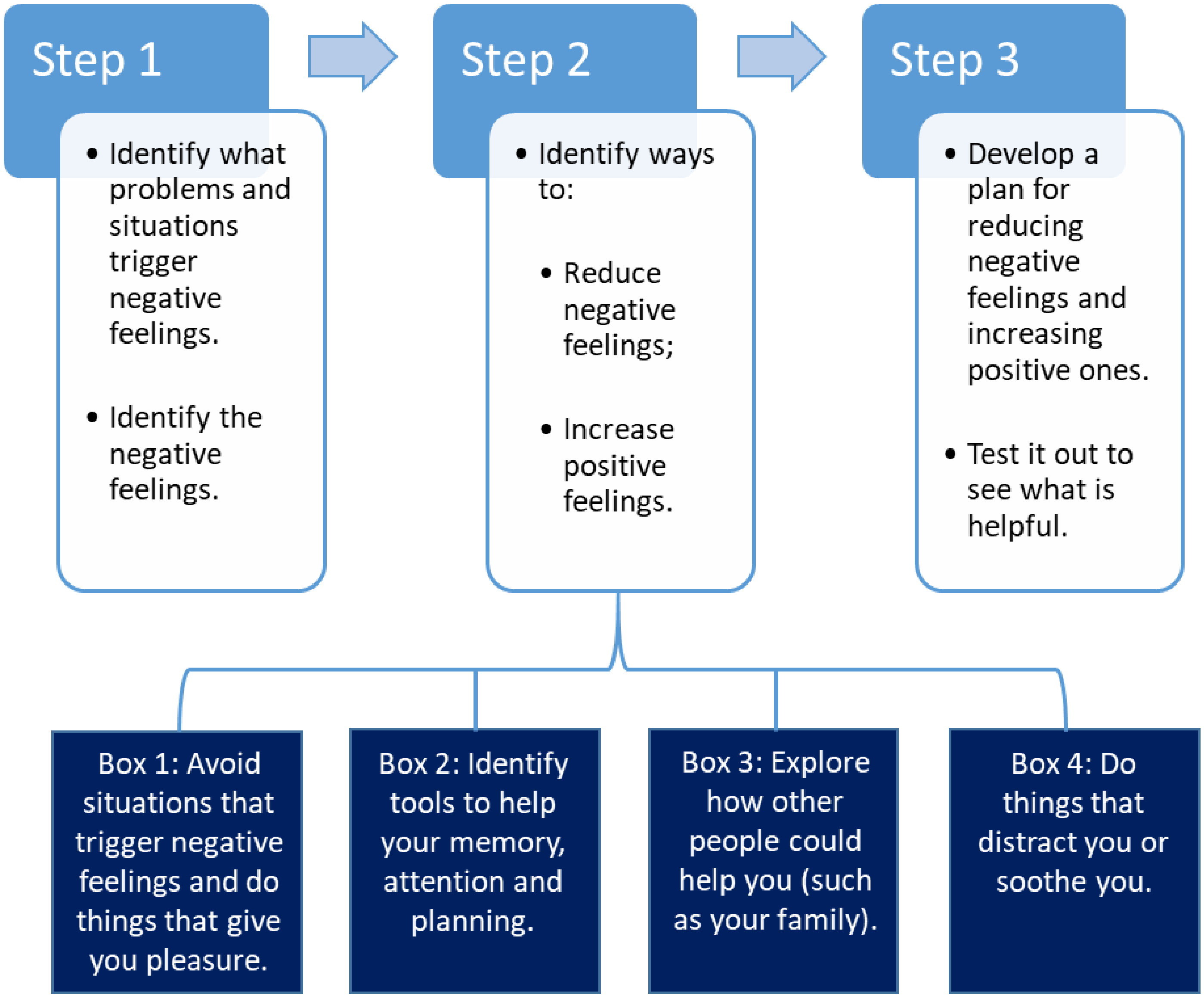

INFORMATION ABOUT PROBLEM ADAPTATION THERAPY (version B)

What is Problem Adaptation Therapy?

Problem Adaptation Therapy (PATH) is a new form of talking therapy for people with mild to moderate dementia who are experiencing difficulties with low mood that is being tested in the UK.

What are the goals of PATH?

PATH aims to help people feel better by finding ways of reducing negative feelings (such as sadness or frustration) and increasing positive feelings (such as happiness or contentment).

Figure 1: Problem Adaptation Therapy (PATH)

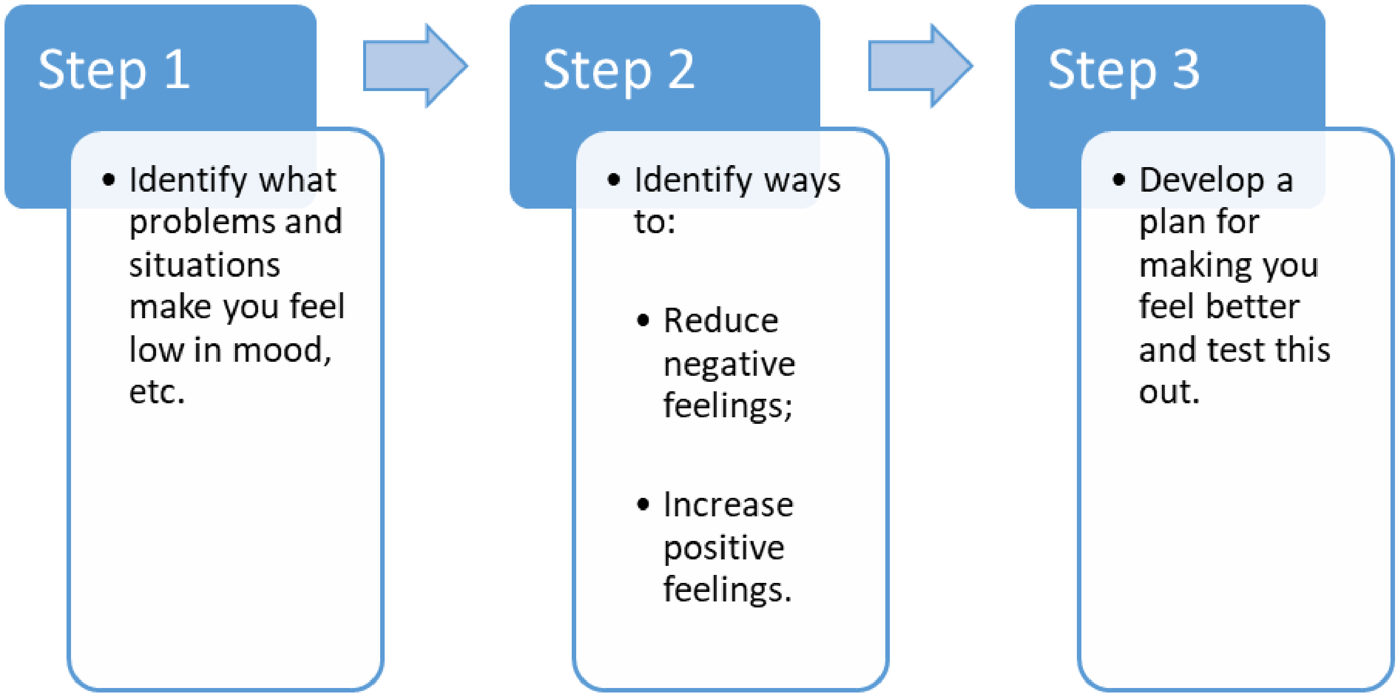

A SUMMARY OF STRATEGIES USED IN PATH

STEP 1: UNDERSTAND THE PROBLEM OR SITUATION

- Explore the details of the problem or situation (What? When? How often? Where? With whom?).
- Consider why the problem or situation might be occurring. Look at the **List of common obstacles or barriers** worksheet for examples, if necessary.
- Identify the negative feelings that are triggered by this problem or situation. Look at the **List of negative feelings** worksheet for examples, if necessary.

STEP 2: SEE HOW WE CAN REDUCE NEGATIVE FEELINGS AND INCREASE POSITIVE FEELINGS

- Consider whether any of the following strategies could help reduce negative feelings or increase positive feelings when this problem or situation occurs.

**Strategy**	**Where to look for guidance**
Avoid situations that trigger negative feelings.	See whether the problem or situation can be avoided.
Do something pleasurable.	Look at the **Activities that I enjoy doing** worksheet, if necessary.
Change the situation (e.g. use memory aids or ask for help with something).	Look at the **List of tools to support memory, attention and planning** and the **Activity plan - smaller steps** worksheets, if necessary.
Use distraction strategies (e.g. watch TV).	Look at the **List of strategies for distracting or soothing myself** worksheet, if necessary.
Use self-soothing strategies (e.g. practice relaxation).	Look at the **List of strategies for distracting or soothing myself** sheet, if necessary.
Change the way you look at a situation (e.g. focus on what you can do rather than on what you cannot do).	Look at your personalised summary of therapy for a reminder of what helped you feel better.

STEP 3: PUT A PLAN TOGETHER

Consider how you could test out whether any of the strategies in Step 2 are helpful for making you feel better.

LIST OF COMMON OBSTACLES OR BARRIERS

Here are some common obstacles or barriers that can get in the way of people doing things.

**Are any of these obstacles or barriers getting in the way of you doing things?**<br><br>*Put a tick in the box ☑ if the obstacle or barrier applies to you.*	
**Physical obstacles or barriers**	**Emotional obstacles or barriers**
☐ Feeling unwell	☐ Finding it difficult to feel pleasure anymore
☐ Being in pain	☐ Feeling unmotivated
☐ Feeling too tired	☐ Feeling irritated or frustrated
☐ Finding it difficult to move around or get out	☐ Feeling anxious or worried or lack of confidence
☐ Finding it difficult to hold or manipulate things	☐ Feeling like you're a burden
☐ Finding it difficult to see or hear	☐ Feeling embarrassed or loss of self-esteem
Other:	Other:
**Mental obstacles or barriers**	**Relationship obstacles or barriers**
☐ Difficulties with memory	☐ Lost contact with others
☐ Difficulties with attention or concentration	☐ Disagreements with others
☐ Difficulties with planning or organising things	☐ Difficulties that other people are experiencing
☐ Difficulties with starting things or getting going	☐ Don't know what to talk about
☐ Finding it difficult to read or write	☐ Others stopping you from doing things
Other:	Other:

LIST OF NEGATIVE FEELINGS

Some common negative feelings that people experience when they are faced with a difficult problem or situation are shown below.

How do you feel when you are faced with this difficult problem or situation?

Put a tick in the box ☑ next to all of the responses that apply to you when you are faced with this difficult problem or situation.

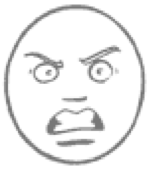	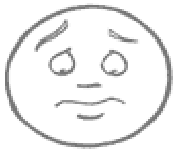	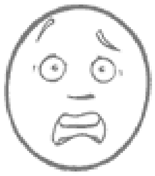	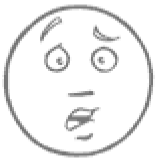	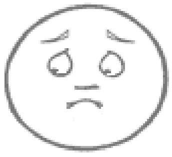
☐ Angry	☐ Miserable	☐ Scared	☐ Hurt	☐ Ashamed
☐ Annoyed	☐ Sad	☐ Anxious	☐ Betrayed	☐ Guilty
☐ Frustrated	☐ Lonely	☐ Worried	☐ Offended	☐ Regretful
☐ Irritated	☐ Discouraged	☐ Stressed	☐ Rejected	☐ Embarrassed
☐ Impatient	☐ Unmotivated	☐ Confused	☐ Disappointed	☐ Disgusted

Other (please specify):

ACTIVITIES THAT I ENJOY DOING

Some common activities that people enjoy doing are shown below. Which ones do you enjoy doing?

Put a tick in the box ☑ next to an activity if you think that you might enjoy it or have enjoyed it previously.

**Daily activities**	**Self-care & spiritual activities**	**Social activities**	**Leisure activities & hobbies**	**Outdoor & physical activities**
☐ Watching TV or listening to the radio	☐ Grooming (e.g. shaving, wearing makeup)	☐ Having people over for something to eat or drink	☐ Reading books, newspapers or magazines	☐ Going to a theatre, cinema, concert, gallery, museum
☐ Setting the table	☐ Wearing favourite clothes	☐ Spending time with family or friends	☐ Writing (e.g. letters, stories, poetry)	☐ Watching wildlife
☐ Cooking, preparing snacks or drinks	☐ Having a bath, shower or massage	☐ Chatting to family or friends (e.g. phone)	☐ Painting, drawing or doing crafts	☐ Going to watch sports (e.g. football game)
☐ Doing the cleaning or light housework	☐ Taking care of oneself (e.g. eating healthily, taking medication)	☐ Having meals or drinks with family or friends	☐ Knitting, crocheting or sewing	☐ Going on outings (e.g. park, picnic, shopping for leisure)
☐ Doing the laundry or ironing	☐ Going to the barber or hairdresser	☐ Discussing photos of family or friends	☐ Doing crosswords or puzzles	☐ Gardening
☐ Doing the dishes	☐ Going to a spiritual gathering (e.g. church) or retreat	☐ Recalling and discussing past events with others	☐ Listening to music, singing or playing a musical instrument	☐ Doing exercise (e.g. walking, swimming, cycling, gym)
☐ Doing the shopping	☐ Reading a spiritual text or praying	☐ Meeting new people	☐ Watching sports on TV (e.g. tennis)	☐ Going dancing
☐ Watching, listening to or reading about the daily news	☐ Practising meditation or relaxation	☐ Going to a social group or gathering	☐ Playing games (e.g. board games, computer games)	☐ Doing yoga, pilates or Tai Chi
Other:	Other:	Other:	Other:	Other:

LIST OF TOOLS TO SUPPORT MEMORY, ATTENTION AND PLANNING - WORKSHEET 2

Put a tick in the box ☑ if you think a tool or strategy would be helpful for you.

Memory aids - things that you can see:

- ☐ Calendars
- ☐ Clocks with day, date, month, and year
- ☐ Coloured tags
- ☐ Daily checklists
- ☐ Diaries or weekly planners
- ☐ Magnetic notepads
- ☐ Markers
- ☐ Medication kit/blister packs/dosette box
- ☐ Notebooks
- ☐ Pictures and/or signs (black and white signs, coloured signs)
- ☐ Sticky notepapers and reminders (e.g. post-it notes)
- ☐ Whiteboard

Memory aids - things that you can hear:

- ☐ Alarms and alarm clocks (with clear signs to show what the alarm is a reminder of)
- ☐ Computerised phone calls
- ☐ Customised audiotapes
- ☐ Key-chain recorder
- ☐ Timers (with clear signs to show what the timer is a reminder of)
- ☐ Timed pre-recorded messages
- ☐ Voice alarms

Strategies to help attention

- ☐ Reduce distractions

Strategies to help planning

- ☐ Think about the things you might need to do before completing a task
- ☐ Break a task down into smaller steps
- ☐ Keep instructions and checklists in labelled folders

ACTIVITY PLAN - SMALLER STEPS

Breaking an activity down into smaller steps can make it feel less overwhelming and can help you to make sure that you don't miss out important steps. An example of putting this strategy into practice is presented below:

What is the activity that you would like to do?

EXAMPLE: Go to the Day Centre

**What smaller steps do you need to take to complete this activity?** Write these in the boxes below.	**Tick (✓) when the step is completed**
Get up at 08:30	✓
Shower at 08:45	✓
Get ready and dress at 09:00	✓
Call for a taxi at 09:15 (0785 478 3269)	✓
Get the taxi to go to the Day Centre at 10:00	✓

What is the activity that you would like to do? Write it in the space below:

**What smaller steps do you need to take to complete this activity?** Write these in the boxes below.	**Tick (✓) when the step is completed**

LIST OF STRATEGIES FOR DISTRACTING OR SOOTHING MYSELF

Some common ways to distract yourself or make yourself feel better when you are feeling distressed (e.g. sad, anxious, embarrassed, worried, frustrated, hopeless) are listed below.

**Put a tick in the box ☑ if you think one of the following strategies might be helpful for distracting yourself or making yourself feel better when you are distressed.**	
Physical strategies	*Creative strategies*
☐ Go outside (e.g. garden or park)	☐ Do something artistic - drawing, painting or colouring
☐ Squeeze a stress ball	☐ Listen to music
☐ Do some cleaning	☐ Play a musical instrument
☐ Do some exercise (e.g. go for a walk)	☐ Sing a song
Other:	Other:
Productive strategies	*Soothing strategies*
☐ Read a book, newspaper or magazine	☐ Take a long bath/shower or have a massage
☐ Do a jigsaw or puzzle	☐ Watch your favourite film or TV show
☐ Do a crossword or word search	☐ Have some comforting food or drink
☐ Do the washing up	☐ Call a friend or family member
Other:	Other:

CALENDAR OF ACTIVITIES

1. Write down in a few words the pleasurable activity that you will do over the next week.<br><br>2. After you have completed the activity, rate how much you enjoyed this activity from 1 (not at all) to 6 (very much) using the rating scale on the right.	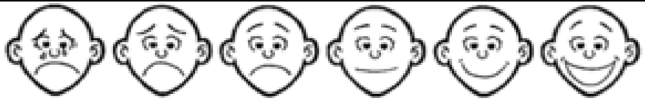<br>1 2 3 4 5 6<br>Not at all Very much

Mon AM	Tues AM	Wed AM	Thu AM	Fri AM	Sat AM	Sun AM
e.g. Go shopping 5	*e.g. Go to the Lunch Club 4*		*e.g. Go to the Lunch Club 5*		*e.g. Go to the cinema 6*	
**Mon PM**	**Tues PM**	**Wed PM**	**Thu PM**	**Fri PM**	**Sat PM**	**Sun PM**
		e.g. Coffee with friend 5		*e.g. Go for a walk 4*		*e.g. Dinner with family 6*

THINGS TO TRY OUT BEFORE THE NEXT SESSION

Summary of today's session

What we discussed:

Home practice

What we agreed to try out before the next session:

1.

2.

3.

4.

THINGS TO TRY OUT BEFORE THE NEXT SESSION (continued)

Experience of home practice

If you have been able to complete what you agreed to try out before the next session, then please write down some brief notes about it:

How did it go?

How did you feel afterwards?

What was helpful?

What was not helpful?

If you come to the clinic for your sessions, please don't forget to bring your PATHFINDER materials with you to the next session.

Chapter 6: References

Alexopoulos GS, Areán P, Raue P. Problem-solving therapy versus supportive therapy in geriatric major depression with executive dysfunction. Am J of Geriatr Psychiatry 2003;11:46-52.

Alexopoulos GS, Raue P, Kiosses DN, Mackin RS, Kanellopoulos D, McCulloch C, Arean PA (2011) Problem-solving therapy and supportive therapy in older adults with major depression and executive dysfunction: effect on disability. Archives of General Psychiatry 68: 33-41.

Alexopoulos GS, Abrams RC, Young RC, Shamoian CA. Cornell Scale for Depression in Dementia. Biol Psychiatry 1988;23:271-284.

Areán PA, Raue P. Social Problem Solving Therapy for Depression and Executive Dysfunction. 2002. Unpublished Manual.

Arean PA, Raue P, Mackin RS, Kanellopoulos D, McCulloch C, Alexopoulos GS (2010) Problem-solving therapy and supportive therapy in older adults with major depression and executive dysfunction. American Journal of Psychiatry 167: 1391-8.

Banerjee S, Hellier J, Dewey M, Romeo R, Ballard C, Baldwin R, Bentham P, Fox C, Holmes C, Katona C, Knapp M, Lawton C, Lindesay J, Livingston G, McCrae N, Moniz-Cook E, Murray J, Nurock S, Orrell M, O'Brien J, Poppe M, Thomas A, Walwyn R, Wilson K, Burns A (2011) Sertraline or mirtazapine for depression in dementia (HTA-SADD): a randomised, multicentre, double-blind, placebo-controlled trial. Lancet 378: 403-11.

Bergh S, Selbaek G, Engedal K (2012) Discontinuation of antidepressants in people with dementia and neuropsychiatric symptoms (DESP study): double blind, randomised, parallel group placebo controlled trial. BMJ 344: e1566.

Brown GK, Bruce ML, Pearson JL. High-risk management guidelines for elderly suicidal patients in primary care settings. Int J Geriatr Psychiatry. 2001;16:593-601.

Burns A, Jacoby R, Levy R (1990) Psychiatric phenomena in Alzheimer's disease III: disorders of mood. British Journal of Psychiatry 157:81-6.

Cicerone KD, Dahlberg C, Kalmar K, Langenbahn DM, Malec JF, Bergquist TF, Felicetti T, Giacino JT, Harley JP, Harrington DE, Herzog J, Kneipp S, Laatsch L, Morse PA. Evidence-based cognitive rehabilitation: recommendations for clinical practice. Arch Phys Med Rehabil 2000;81:1596-1615.

D'Zurilla TJ, Nezu AM. Problem-solving therapy: a social competence approach to clinical intervention. New York: Singer, 1999.

Enache D, Winblad B, Aarsland D (2011) Depression in dementia: epidemiology, mechanisms and treatment. Current Opinion in Psychiatry 24: 461-72.

Gonzalez-Salvador MT, Arango C, Lyketsos CG, Barba AC (1999) The stress and psychological morbidity of the Alzheimer patient caregiver. International Journal of Geriatric Psychiatry 14 701-10.

Greenwald BS, Kramer-Ginsberg E, Marin DB (1989) Dementia with coexistent major depression. American Journal of Psychiatry 146:1472-8.

Gross JJ. The emerging field of emotion regulation: An integrative review. Rev Gen Psychol. 1998;2(3):271-299.

Gross JJ. Emotion Regulation: Conceptual and Empirical Foundations. In Gross JJ, ed. Handbook of Emotion Regulation. 2nd ed. New York, NY: Guilford; 2014:3-20.

Hamilton M. A rating scale for depression. J Neurol Neurosurg Psychiatry 1960; 23:56-62.

Kessing LV, Harhoff M, Andersen PK (2007) Treatment with antidepressants in patients with dementia – a nationwide register-based study. International Psychogeriatrics 19: 902-13.

Kiosses DN, Arean PA, Teri L, Alexopoulos GS (2010) Home-delivered Problem Adaptation Therapy (PATH) for depressed, cognitively impaired, disabled elders: A preliminary study. American Journal of Geriatric Psychiatry 18: 988-98.

Kiosses DN, Ravdin LD, Gross JJ, Raue P, Kotbi N, Alexopoulos GS. Problem adaptation therapy for older adults with major depression and cognitive impairment: a randomized clinical trial. JAMA Psychiatry. 2015 Jan;72(1):22-30. PubMed PMID: 25372657.

Kiosses DM, Teri L, Velligan DI, Alexopoulos GS (2011) A home-delivered intervention for depressed, cognitively impaired, disabled elders. International Journal of Geriatric Psychiatry 26: 256-62.

Kirkham JG, Choi N, Seitz DP (2016) Meta-analysis of problem solving therapy for the treatment of major depressive disorder in older adults. International Journal of Geriatric Psychiatry 31:526-35.

Lyketsos CG, Steele C, Baker L, Galik E, Kopunek S, Steinberg M, Warren A (1997) Major and minor depression in Alzheimer’s disease: prevalence and impact. Journal of Neuropsychiatry and Clinical Neuroscience 9: 551-61.

Orgeta V, Qazi A, Spector A and Orrell M (2015) Psychological treatments for depression and anxiety in dementia and mild cognitive impairment: systematic review and meta-analysis. British Journal of Psychiatry 207: 293-8.

Orgeta V, Tabet N, Nilforooshan R, Howard R (2017) Efficacy of antidepressants for depression in Alzheimer’s disease: Systematic review and meta-analysis. Journal of Alzheimer’s Disease 58:725-733.

Rosenberg PB, Drye LT, Martin BK, Frangakis C, Mintzer JE, Weintraub D, Porsteinsson AP, Schneider LS, Rabins PV, Munro CA, Meinert CL, Lyketsos CG and the DIADS-2 Research Group (2010) Sertraline for the treatment of depression in Alzheimer disease. American Journal of geriatric Psychiatry 18:136-145.

Starkstein SE, Jorge R, Mizrahi R, Robinson RG (2005) The construct of minor and major depression in Alzheimer’s disease. American Journal of Psychiatry 162: 2086-93.

Stern Y, Tang MX, Albert MS, Brandt J, Jacobs DM, Bell K (1997) Predicting time to nursing home care and death in individuals with Alzheimer’s disease. JAMA 277: 806-12.

Teri L, Logsdon RG, Uomoto J, McCurry SM. Behavioural treatment of depression in dementia patients: A controlled clinical trial; Journal of Gerontology: Psychological Sciences 1997;52:159-166.

Thompson S, Herrmann N, Rapoport MJ, Lanctot KL (2007) Efficacy and safety of antidepressants for treatment of Alzheimer’s disease: a meta-analysis. Canadian Journal of Psychiatry 52: 248-55.

Velligan DI, Bow-Thomas CC. Two case studies of cognitive adaptation training for schizophrenic outpatients. Psychiatr Serv 2000a;51:25–29.

Velligan DI, Bow-Thomas CC, Huntzinger C, Ritch J, Ledbetter N, Prihoda TJ, Miller AL. Randomized controlled trial of the use of compensatory strategies to enhance adaptive functioning in outpatients with schizophrenia. Am J Psychiatry 2000b; 157:1317-1323.

[Return to Table of Contents]

Chapter 7: Appendices

Press 'ctrl' at the same time as clicking on a heading to follow the link to that section.

Appendix 1: Themes from qualitative interviews and focus groups in the PATHFINDER study.

Appendix 2: Tools to support memory, attention and planning.

Appendix 3: Safeguarding guidelines

Appendix 4: Case examples.

Appendix 5: Issues to discuss in therapist supervision.

Appendix 6: PATH cheat sheet - a reminder of the key principles of PATH

Appendix 7: Examples of problems faced by people with mild to moderate depression and dementia.

Appendix 8: Suicidal ideation guidelines

Appendix 9: PATH conceptualisation

Appendix 10: An example of a personalised treatment summary for a person with milder dementia and a person with more moderate dementia.

Appendix 11: An example of a personalised treatment summary for a person with more moderate dementia.

[Return to Table of Contents]

Appendix 1: Themes from qualitative interviews and focus groups in the PATHFINDER study.

Summary of interviews with people with dementia and depression, their caregivers, and healthcare professionals

We conducted interviews with people with mild-to-moderate dementia and depression, separate interviews with their main caregivers, and a series of focus groups with healthcare professionals who routinely work with people with dementia. We focused on gaining a clearer understanding of their experiences of living with and caring for someone with dementia and depression, and their preferences with respect to psychological interventions. This was in order that PATH can be grounded in these experiences and clinicians delivering PATH can begin with a clear understanding of the challenges that they will have to engage with. A summary of the main themes regarding the experience of dementia and depression, the **potential negative emotions associated with the themes and triggers** (for both the person with dementia and the caregiver), and the main points regarding intervention preferences is provided below.

Loss of social contact

Loss was a major theme in all of the interviews with people with dementia and their caregivers, manifesting in many different ways. A major contributing factor towards depression was loss of social contact **(Potential Negative Emotions: Feelings of Isolation, Loneliness)**. People with dementia talked about how they have lost many friends because they are not able to go out as much due to memory and physical health problems. They also said that when they do go out, they find it harder to interact in the ways they used to **(Potential Negative Emotions: Frustration, Helplessness, Worthlessness, Low self-esteem)**. For example, some felt unable to go to the pub as the level of background noise, combined with general confusion, prevented them from following conversations. Several people expressed embarrassment at their symptoms, such as forgetfulness during conversations, and irritability and frustration at not understanding others or being understood by others **(Irritability, Frustration)**. Some actively avoided spending time with friends in the way they used to as they felt they had less to talk about as they either do or remember less. Reduced family contact was also a factor in social isolation with adult children and grandchildren moving away and being busy with their lives. This was frequently mentioned as a factor in low mood. Caregivers and clinicians also identified loss of social contact as a key issue, and caregivers reported that one of the main things they think would help their relatives' mood is having more social contact **(Feelings of Isolation, Loneliness)**.

Edith, 73 - "It's nice having somebody to talk to, you know what I mean? I mean, all my friends are dead now, they all died, all my best friends."

Jenny, 69 - "I have definitely closed in on myself. I know I've become socially much more isolated. You just, you know, it's too much of a faff being able to get somewhere, to meet somebody and… it's getting there on public transport and things like that…"

Agata, 78 - "Sometimes I wake up in the morning and feel sad… I feel like I've got four kids, and I get up and I'm on my own. Before, when I used to have the grandchildren, they were here all the time, but now it's empty, no one here. It's horrible. Having house empty."

Cassie, daughter of Sam - "He's very focused on one option, he can't deal with any distraction near him, or beside him…"

Loss of functioning and independence

Another common theme related to loss was the loss of functioning and independence. People with dementia and their caregivers both said that they felt that being unable to do things that they used to do with ease leaves people with dementia feeling upset and frustrated and contributes to depression, which clinicians agreed with **(Helplessness, sadness)**. Many people with dementia associated low mood with times when their memory was particularly bad, and felt that the confusion and frustration caused by 'bad memory' days left them with a sense of **hopelessness** and **uselessness**. Additionally, several people with dementia identified the impact that these days have on overall confidence, with fear of making mistakes stopping people with dementia from trying to do things **(Low self-esteem)**. Being unable to go to the shops or visit friends without support from a caregiver was often raised as a particular concern, with feelings of being a burden exacerbating low confidence and low mood, which in turn prevented people from trying to do things themselves **(Burdensome, uselessness)**.

Clinicians talked about people with dementia being aware that there is an infrastructure developing around them that

both compensates for and reinforces their reduced independence. Some people with dementia identified that family members doing too much for them was making them feel worse, and that they felt they could be doing more, but caregivers were not letting them **(Resentment)**. Several expressed sadness at being aware that people were frustrated at them for doing things more slowly, for example, and so ended up not doing things even if they wanted to **(Disappointment)**.

Jenny, 69 - "Virtually everything that I enjoyed was mental. So… you know… it's the quality, my quality of life has absolutely plummeted. The real catastrophe has been reading. Because previously I read about three books a week, depending on the book obviously. And now I can't follow a whole book, anymore, I'm reading short stories. Which I find immensely unsatisfying."

Edna, 68 - "I loved jewellery making! I used to make them, take them and give them to people at church and all that and all that, I used to have on the TV a channel to help you make it, but now that I can't find the channel, I don't know how to find the channel…"

Agata, 78 - "I used to be… active woman, I go to aerobics! Now I'm stuck here, it's difficult. I used to go and do my shopping, my own shopping. Now I don't know how to do my shopping."

Cassie, daughter of Sam - "He used to always go out, go shopping by himself, he can't do that anymore. Or he will go to the shop and know what he wants, but he gets there, he has no idea what he was getting. I think he'd be happier if he could go to the shop on his own and come back with what he wanted."

<u>Loss of key life roles</u>

Many people have a role in life that is a core part of their self-image and self-esteem. Most participants spoke about how, as their memory problems progressed, they had lost one or more important roles in their lives, and as a result were feeling very depressed and as if they were losing themselves and their purpose in life **(Potential Negative Emotions: Feeling lack of purpose, Discouragement, Sadness)**. For example, losing one's role or identity as an intellectual person because of being unable to read and remember books anymore, losing roles as church or community leaders, or losing roles as the provider for the family or the person who looks after the whole family **(Worthlessness, Helplessness, Discouragement, Hopelessness)**. Caregivers also identified this as a key issue contributing to low mood. Clinicians talked about loss of role, but their focus was mainly around traditional gender roles, rather than considering the many other important roles in participants' lives.

Leticia, wife of John - "He's been very down. He's been very down. Because I mean he used to be very active, used to work in IT, did a Masters degree and everything. So he's always led a very active life, so now he feels like his life is turning round around him. So it's not been easy."

John, 66 - "I feel low because I'm always in a supporting role. I support… many, many other communities. But now, this is me. I've not been able to participate and do… things that I used to do in the past, you know… I feel…I'm not even capable."

Focus Group 2 – Healthcare practitioners working with dementia - "Where because the person has been either diagnosed with dementia or has been quite frail, that there's this idea that we look after mum and mum doesn't do anything. She doesn't need to do anything because we look after her, because we're good children and family. So what does mum do, she just sits."

<u>Caregiver Involvement</u>

All participants felt that having a caregiver involved in an intervention would be beneficial to both the person with dementia and their caregiver. Many caregivers thought that it would give them something constructive to do with the person they cared for, and that it would help them get a better understanding of how to help with low mood. People with dementia generally liked the idea of having a caregiver attend sessions with them. However, some expressed hesitation around talking about some issues with caregivers present, burdening their caregivers with another thing for them to do, and in some cases, whether their caregiver would actually want to attend **(Burdensomeness, Worthlessness)**. Some people with dementia also felt that their caregiver might be inclined to talk on their behalf, and talk about what they think the person with dementia wants, which may not align with what the person actually wants **(Loss of control, helplessness)**. Some caregivers reported that they would have more problems to share than the person with dementia, with some adding that people with dementia often forget what the problems are, or don't see

things as being problematic as the caregivers think they are **(Helplessness)**. Some caregivers expressed worry about coming to sessions, with their primary concerns being around upsetting their relative by disagreeing with them about what the problems were, and getting backlash (e.g. criticism) from them or having arguments with them later due to disagreeing about things **(Helplessness, Guilt)**.

June, wife of Charlie - "What I'll get out of it, is for me to understand, so then I know I can sit down with him and explain to him what they're talking about, the therapy."

Cassie, daughter of Sam- "It would give me a little bit of a break away to do something to help, with him, my dad. Like a little thing that we could do together."

Expert Patient Focus group - "If you are a bit low, it could well be that the person you've come with, will be doing all of the talking before you. Then they say what they think you want, rather than what you want. And nobody's going to argue, because it's in therapy."

Frank, husband of Margaret - "I'd rather let the wife do her thing… Though she might make mistakes, she can't remember something. Because, afterwards, I know I get the backlash. 'Why did you make me look a fool?' So I let her make her own statements."

Edith, 73 - "It would be helpful for him to come. Because I can't explain everything to him, because he just thinks I've got a screw loose, you know. Whether he would be interested, I don't think he would, really. He's a sort of a hard nut, in that way (laughs). He thinks he can solve all his own problems, but he can't. You know, he can't."

<u>Relationship tensions</u>

In focus groups, healthcare professionals frequently expressed concern that the problem of relationship tensions between the person with dementia and their caregiver would frequently dominate sessions and need to be the focus of problem solving. However, while some people with dementia acknowledged relationship tensions exist at times, both people with dementia and their caregivers reported their main concerns were general irritability and anxiety about telling each other how they are really feeling and coping **(Irritability, Anxiety, Frustration)**. This was typically reported as being an effect of dementia rather than relationship tensions *per se*. Some people with dementia felt that differing perspectives could cause tension as it is difficult to understand their point of view if you don't have dementia **(Anxiety, Helplessness, Irritability)**.

Frank, husband of Margaret - "She gets upset when her daughter doesn't come around. I talk to her about it but I don't say too much, because if I do, she'll start… start getting quick tempered."

Edith, 73 - "We both get so fed up with one another. That's what's really doing me. We've been together 46 years, and I never thought that would happen, you know."

Charlie, 81 - "She's a good wife to me but I don't want to bother her. Anytime I say don't bother her because I know she'll be concerned too much."

Expert Patient Focus group - "Some people feel more confident, and other people think 'I'm not going to let my wife know what I really think, because I don't want to worry her'."

<u>Caregivers' own needs</u>

Caregivers and clinicians identified a number of concerns that caregivers might have, such as their own mental health difficulties, physical health problems, high numbers of other demands on their time and energy, and the stress of the day-to-day difficulties of being caregivers **(Feeling overwhelmed)**. These often culminated in general **stress** and **frustration**, but caregivers typically talked about wanting something constructive to do with their relative to reduce stress for both parties **(Guilt)**. All participants talked about this as something to be aware of when delivering PATH, to acknowledge the difficulties caregivers might have in supporting their relative in PATH, and to not assume that caregivers are easily available and in a better state of health than the person with dementia themselves.

This theme links strongly with the theme of burden (described below), as people with dementia tended to be acutely aware of everything that their caregiver was doing for them and the fact that they were also experiencing their own difficulties and stresses.

Margaret, 81 - "He's getting a bit deaf so it's very hard for him."

Cassie, daughter of Sam - "I let him come shopping with me, but then he gets stressed out there as well. Because like I've got him and I've got David, my son, with me, it's like having two children, two toddlers. If I'm near a road, dad will come holding the back of David, and David will hold my hand. Then David will kick off, then Dad will kick off, it's really like two toddlers. It's horrible. Two toddlers struggling. So now just half the time I won't let Dad come out with me. It's just for my stress levels, I can't deal with it."

June, wife of Charlie - "Almost everything now I am the one doing it. If he needs another channel, he'll call somebody if they can put that other channel for him. But he won't do it. If we were to have the therapy, I want to know what to say, to be helpful for him. I don't want to lose my temper with him."

Burden

Most people with dementia talked about the feeling of being a burden, with some even suggesting that their families would be better off without them **(Burdensomeness)**. Many people spoke about this as something that they felt was their responsibility to manage, by keeping things to themselves rather than talking to others about how they were feeling **(Feeling isolated, not connected)**. Caregivers expressed awareness of their relatives' fears of being a burden, and felt that they had to manage this so that they did not inadvertently give this impression **(Worry)**. Clinicians thought that engaging in something like PATH together might help reduce the feelings and worries about burden in both people with dementia and their caregivers.

Frank, husband of Margaret - "Her mum was a nice lady, but with this dementia, like, she just… Just went. My wife went up every day, and she didn't even know us. So she does worry about becoming like that… she does worry a lot… she doesn't want to be a burden on people."

Nancy, daughter of Edna - "There would have to be an individual one-on-one session. Because… yeah… you don't ever want that person to feel like they're a burden, and there's some things that they don't recognise that they do, which could be frustrating to you being the caregiver, that you don't necessarily want to say in front of that person, because… it's not nice to hear…"

Edna, 68 - "I'm not going to visit my mother anymore, because I don't want to make her sad, so I don't go… I have decided, that this is something for me to deal with, and I'm not going to burden my family with it."

Anxiety

People with dementia frequently reported **worry** and **anxiety** since their diagnosis, when asked what kind of problems or difficulties they were experiencing now. They reported that their symptoms cause anxiety (e.g. worrying about forgetting things and making mistakes), as well as worrying about the future and the impact of their dementia on their loved ones **(Hopelessness)**. Caregivers reported that they felt that anxiety leads to **irritability** and **impatience**, and that this in turn leads to lower mood. The impact of anxiety can be seen in many areas, particularly in loss of social contact **(Isolation)**, **self-esteem**, and **burden**, so it is likely to be a challenge that PATH therapists will frequently encounter. Many people with dementia reported that anxiety stops them from doing things just as much as their dementia or depression symptoms.

Nancy, daughter of Edna - "She's… a lot less patient, and I think that's because she has to get what she has to get out, because her fear of not remembering."

Leticia, wife of John - "He's very sensitive to little things. So we've noticed and we don't really discuss anything to do with finance, to do with the house, we don't discuss it with him because he's not working at the moment so he feels he needs to contribute, you know? So we don't discuss anything, because it sets his anxiety level high."

Edna, 68 - "I worry about my bills, because I did get a bit confused on Monday, I was a little bit unsure when I went to go pay them. I just don't want, you know, to have a letter come from someone saying you didn't pay your bills for how many months, and that is what I get anxious about."

John, 66 - "I go to church, but I worry a bit now about it. You just have to be careful what you say and what you do and all that, because in the church, there are things that you have to say to members of the church. Just like, this is how to talk to… members, or these are new members that are coming in, and you don't want to just say anything to

them… that will upset them, or… yeah. So I worry a bit about that and about many things."

Self-esteem and self-stigma

A common theme running through several of the other themes was low self-esteem. Many people with dementia talked about themselves with self-critical or self-stigmatising language that is likely to reflect how they see themselves now **(Worthlessness, Low Self-Esteem)**. For example, people with dementia used terms such as "useless", "incapable", "backwards", "falling apart", "a nutter", "nobody", and "not important" **(Low self-esteem, worthlessness)**. Some people with dementia also reported a reluctance for other people to know they have dementia for fear of being treated differently because of this **(Feeling stigmatized, Worthlessness)**. Several caregivers mentioned their relatives' low self-esteem, with a mix of people who had always had low self-esteem, and those who had developed it since their memory problems began.

Jenny, 69 - "My mind has always been, you know, just super A one. So, so much of my sense of self worth has been because of that. And now I can't even read a book."

Charlie, 81 - "I cannot provide for my wife. And that makes me think a lot. In who am I? I always feel I'm nobody. I feel very hopeless… A man has to provide for his family."

Sam, 66 - "I'm just useless."

Charlotte, 85 - Interviewer: When you've had problems in the past, have you been somebody to talk it through with someone? You know, when you've had upset in your life?" Interviewee: "You mean before I was a nutter?"

John, 66 - "Because I'm always in a supporting role. I support… many, many other communities. But now, this is me. I'm not even capable. I have three children. I worry about them now. I do. Especially that I'm not capable. Can't run after them and look after them, and things like that."

Waiting to die

It was common for people with dementia to talk about feeling as if their life was over and they were just waiting to die **(Hopelessness)**. Several people talked about actively wishing that they were dead **(Hopelessness, Feeling suicidal)**. Caregivers often reported that they were aware their relative felt like this, and this exacerbated their own feelings of not knowing what to do to help, as well as their own low mood **(Helplessness of caregiver)**.

Jenny, 69 - "My idea of the best thing that could happen to me is not to wake up tomorrow morning. Which is unfortunately not going to be the case, but however that is the way I do feel. I have felt this way since the diagnosis."

Edna, 68- "Sometimes I did feel suicidal, I was thinking in my mind that if I did die, maybe it would be better."

Natasha, of Anne - "She talks about dying, but not like in a suicidal way. Just that she's sort of looking forward to her time. Or she hopes her time comes soon. If I talk about something coming up she says oh, I hope I go before then."

Religion

While this theme was not present in all people with dementia, in people where religion was part of their life, this was a strong theme. Religion played a part in their identity and key life roles, with several participants having significant roles as church leaders or community members. It was a clear supportive factor in their lives, either from lone practice of a religion or from knowing their religious community was supporting them.

Edna, 68 - "I mean, the people in the church, I've asked them, they must pray for me. And they made me know that they were praying for me and that things were going to change. And that's what made me just… I just snapped out of it."

Charlie, 81 – "I'm a very private person. Even my wife sometimes I need something, I don't want to talk about it. I keep it to myself. But I always talk to God."

Black and white thinking

There were clear differences in attitudes towards dementia between participants. Some people seemed to feel that their lives were over now and were resigned to merely existing while they waited to die **(Hopelessness, Lack of purpose)** while others viewed dementia as something they had to live with and tried to just carry on with their lives. The former attitude underlies many of the other themes, particularly suicidality, self-esteem and self-stigma, and relationship tensions. Relationship tensions were evident as people with this attitude appeared less inclined to want to do things to help themselves, leading to more **frustration** for caregivers, and **irritability** on both sides **(Helpessness)**. The attitude of life being over was more prominent in those with more severe depression and low motivation.

Jenny, 69 - "But… nobody can change anything. It doesn't matter how good you are at your job. You can't make it go away. And that's the only thing that would make a real difference to me. Which is not going to happen… so… I'd like to believe in fairies, but I don't."

Sam, 66 - "I just turn the TV on and that's it. Just forget everything, try to anyway."

Edna, 68 - "I just felt that I needed to look at myself, because I was never like this before. Because then I was thinking, it's this thing that I've got that's causing me to feel like this, and so, I don't want to feel like this, so I'm just gonna change my own thoughts."

Agata, 78 - "Most of the time I feel lonely, or down. But I just try to cope, what can you do?"

Participant preferences regarding psychological interventions

Language and tone of therapists and intervention materials

All participant groups talked about the importance of using the right language and tone when talking to people with dementia and making sure that clinicians did not appear patronising. People with dementia typically didn't identify with the words 'depression' or 'dementia', but with more general terms such as 'memory problems', 'being fed up', or 'feeling down'. Similarly, clinicians felt the word 'therapy' might be something that people would resist or become defensive about, and some people with dementia suggested there is suspicion about the term 'therapy'. Caregivers reported the need for clinicians to approach this sensitively with people with dementia, use language and examples that are relevant to them, and ask the right questions using small steps to avoid confusing the person with dementia.

Expert Patient Focus group - "I think the word therapy puts it on another level – 'you're not coping, you need help'."
Leticia, wife - "For someone going through a lot, there's a lot of anxiety, you don't need someone else to come and start asking this, this, this, this. No, you need someone calm to be empathetic to look after you. Not someone come in and 'do this, do this, you don't do that'. I didn't enjoy the first meeting I had with the doctor at all."

Frank, husband of Margaret - "She'll agree to be polite. She might think she's upsetting someone. The more questions, though, the more confused she gets."

Nancy, daughter of Edna - "She skims over a lot of what she is thinking but doesn't actually get it out, then she'll come back to it and she'll get it mixed up, and she can't remember… So you need to ask questions in steps."

Importance of flexibility

All participants referenced the value of giving people with dementia choice over where to attend sessions, as well as how long they last. People had different preferences for where to attend sessions with some preferring to leave the house for appointments to give them something to do, and some preferring a clinician visit them so they can be more comfortable and avoid worrying about travel. Clinicians acknowledged that the latter might be impractical, but felt that from their experience if people had the option to go to the clinic or be seen at home it would improve engagement. Some people talked about how they struggle to find words sometimes, suggesting that there would be times when they found it harder to talk to a therapist and that flexibility in the length of sessions would be helpful on these occasions. Some also suggested that a suitable length depended on the content of the session, with people not liking lots of questions or feeling like they were being talked at for the whole time.

Jenny, 69 - "I can see arguments for both sides, having sessions here or at the clinic… but I think, particularly with

me… it'd probably… be good in, you know, sort of to get out and do it… because… that's forcing you to, you know, to just not be in your little cocoon."

Margaret, 81 - *"As my husband always used to say, "you can talk the hind legs off a donkey". But sometimes, sometimes I can't now, you know what I mean?"*

Sam, 66 - *"It depends what you're talking about. If someone's going to keep talking all the time you're going to get pissed off with them, I suppose."*

Frank, husband of Margaret - *"She won't mind sitting here with someone talking, but she wouldn't be interested in going if she had to go to the clinic."*

Homework

Most people with dementia liked the idea of homework and said they would enjoy this. They also liked the idea of having a notebook or folder with worksheets to fill in to record their progress or check what to do. However, some said that homework would have to be simple to complete so as to avoid making them feel overwhelmed. Some caregivers felt that their relatives would struggle with homework, and some people with dementia reported that they were losing their abilities to read and write and so were worried about being able to complete homework.

Edith, 73 - *"I think it's good to have it on alternative days, you know? I don't think I'd be able to do it every day. If I go out, I'd be sitting here at night time doing it, writing it all down."*

Cassie, daughter of Sam - *"I think he might enjoy homework, because he likes to watch A do his homework as well, so it could be something they can do together, it becomes like a joint thing."*

Lucy, daughter of Anne - *"I don't think she'd be able to do it. She wouldn't look. You could say on the phone, and she's say oh what is that then? And she'd go looking for it then get in a panic that she couldn't find it…"*

Expert Patient Focus group - *"I would be a bit, 'oh dear, another thing I've got to do and remember' and I don't know… Just the idea of sitting down and doing this form, it's concentrating, it's organising… which is difficult."*

[Return to Table of Contents]

Appendix 2: Tools to support memory, attention and planning

Further details about tools that can be used to support the person with dementia's memory, attention and/or planning abilities are provided below.

A note on the use of tools in people with dementia: Tools vary in how 'high tech' they are. Voice alarms, customised audio tapes and timed pre-recorded messages are fairly 'high tech', whereas strategically placed notepads, signs/pictures, calendars/diaries/weekly planners, and whiteboards are less so. It can be tempting to try and introduce the use of 'high tech' tools. However, the cost to the person with dementia/caregiver, learning required and the investment of time means that this may not be practical within PATH. **'Low tech' tools should be favoured unless the person with dementia is familiar with 'high tech' ones.** Consideration should be given to **how caregivers can support the person with dementia to make the best use of these tools**, where necessary.

Memory aids - visual tools

Checklists: Checklists can be helpful to most people with dementia. The checklists may remind people of sequences of actions necessary before a task is completed. People with significant memory and executive functioning deficits may benefit by checking off the steps that they accomplish.

Pictures: Pictures may be helpful in initiating certain tasks. Pictures of cooking ingredients attached next to a recipe may help a person to cook. A picture of the person's friend may motivate them to call the friend.

Signs: Signs can be used to trigger behaviour or remind people with dementia to perform an action. For example, a sign at the dinner table or in the kitchen saying "Take your medication" may help remind the person to take their medication and may improve medication adherence. A sign inside the front door saying "Did you take money, keys, and bank cards with you?" may serve as a reminder. Sometimes signs may not offer adequate assistance to severely impaired people and the caregiver may need to help. Furthermore, a person may habituate or get used to the signs and start ignoring them; as a result, they may become less effective. Changing the colour of the signs or the font used can be helpful to trigger the person's attention.

Sticky notepapers and reminders: Sticky notepapers (e.g. post-it notes) and reminders can be used to elicit a response from mildly impaired people. Sticky notepapers are not as large as signs and they may not capture the attention of moderately and severely impaired people.

Magnetised notepads: Magnetised notepads, like sticky notepapers, can be useful for mildly impaired people with dementia.

Coloured tags: Coloured tags can be useful to capture a person's attention and can be used in conjunction with signs and reminders to initiate actions.

Memory aids - acoustic tools

Customised audiotapes: Customised audiotapes with specific instructions can help a person with dementia initiate a specific action, maintain attention, and perform a sequence of actions. For example, customised audiotapes saying "Prepare your clothes for tomorrow morning. First, select your clothes from the wardrobe….Second, put them on the chair near your bed…" may help a person have his or her clothes ready for the next morning. A customised audiotape may have instructions for how to prepare to do certain tasks (e.g. go shopping) or even how to use other reminder tools such as checklists, voice alarms, timers, or pre-recorded messages. For example, customised audiotapes saying "Please take the checklist of household chores from the drawer in the bedroom,…check off the first item on the check list "pick up and hang your clothes",…did you check off the first item?" may help a person keep an orderly home.

Voice alarms: Voice alarms can help a person with dementia initiate a specific action, even though they are not frequently used. For example, a voice alarm saying "call your daughter" may help the person make the phone call. Voice alarms can be used as reminders of an action or of a sequence of actions. For example, a voice alarm saying "Always set the timer when you use the stove" may help a person avoid overcooking his or her food and even prevent accidents. The therapist should prepare the voice messages together with the person with dementia and teach them how to use a voice alarm. The instructions for use of a voice alarm should be with the voice alarm. A caregiver may help the person with dementia, if necessary.

Computerised phone calls: Even though computerised phone calls are rarely used, they are commercially available and function like the wake-up call services offered in hotels. Specifically, a computer program may trigger a phone call to the person with dementia at a specific time and remind them to perform an action through a pre-recorded message. For example, a person may be called in the morning and reminded to take medication, or they may be reminded that they should expect a visit from the physiotherapist in one hour and that they need to start getting dressed. The computer may generate another phone call 15 minutes before the appointment to make sure that the person did not get distracted and got ready for the appointment. However computerised phone calls are used, the PATH therapist must prepare a step-by-step plan for the person with dementia to follow so that he or she will know what behaviour the computerised phone call is meant to be triggering.

Timers: Timers can be used to indicate both the beginning and the end of a task. They are helpful in initiating a sequence of actions. For example, timers can be used to draw attention to beginning the next household chore out of a list of chores. Timers can also help people with attention deficits to keep track of a sequence of actions. Timers may put a high demand on memory unless they are combined with other tools such as notebooks and signs that will indicate the kind of action that needs to be taken. For example, a checklist, a sign, and a timer may help a person to prepare lunch. The checklist can provide the list of the ingredients. For each step, a sign can ask the person to set the timer for the next step. The sound of the timer will indicate the end of one step and the beginning of the next step. The caregiver may be involved, if necessary.

Timed pre-recorded messages: Timed pre-recorded messages are a great tool to trigger specific behaviours. Messages such as "It is time to get dressed" or "It is time to clean the kitchen now - start with washing the dishes" can be used to initiate action. Timed pre-recorded messages may be used to remind people of specific actions. For example, a repeated message like "Call your daughter on 020 4555 1212" can be quite successful in helping the person make the phone call.

Beeping watches: Beeping watches may help people begin a scheduled action. For example, a beeping sound may remind a person to call their granddaughter. For severely impaired people, watches may be used in conjunction with other tools such as signs or reminders. For example, a beeping sound coupled with a sign "Get the post at the sound of the beep" may remind the person to go and get his or her post.

Alarms: Alarms could be used to trigger action. For example, in order to help a physically mobile person who finds it difficult to get out of bed in the morning, the alarm may be set to ring every ten minutes. The therapist and the person with dementia may agree to place the alarm away from the person's bed, the instructions close to it, so that they have to get up in order to disable it. For more impaired people, the caregiver may help the person set the alarm.

Attentional tools

Sustaining attention: This procedure can be used to initiate specific actions or to remind a person of specific steps in a task by helping them to avoid distractions. For example, a person has decided to call his or her friend, but because of distractions, he or she does not make the phone call. During the PATH session, the therapist may observe the person as he or she is going through the steps from deciding to call his or her friend to actually making the phone call. The therapist should identify the reasons for the person getting distracted and provide positive social reinforcement (e.g. encouragement, praise, rewards) to keep the person’s attention on the task. The therapist may keep records of the solution and give it to the person for future reference. Most of the time, this procedure is used in conjunction with minimising distractions (see below).

Minimise distractions: During this process, the therapist may remove environmental distractions or things in the environment that may make it more difficult for a person to focus on the task at hand. In the example of a person who wishes to make a phone call, the therapist may ask the person to make all of his or her calls from a specific room at a specific time when distractions are least likely to occur. In the example of a person who enjoys reading, the therapist may instruct the person to do his or her reading in a room with minimal distractions at a time when they are least likely to be disturbed.

Planning tools

Breaking a task down into smaller steps: This is a useful technique for starting a specific action or a sequence of actions. Step-by-step division of actions can be used in conjunction with other tools such as signs, reminders, etc. For example, going out for a walk in the morning can be divided into several steps: Get out of bed, take a shower, get dressed, eat breakfast, and go out for a walk. Step-by-step planned activities can be part of a written checklist when

needed, and provide a helpful structure for people with problems with getting started. For example, cooking a meal can be divided in small steps: getting all the ingredients, putting the ingredients in the right order and the right amount, and setting the timer on the stove.

Preparatory steps for a task: This strategy is used to increase the likelihood of putting a plan into practice and is used in conjunction with breaking a task down into smaller steps. For example, a person who remains in his or her nightclothes the whole day may make the following plan with the therapist: Wake up using an alarm at 9:00 am, take a shower at 9:15 am, and get dressed at 9:50 am. The preparatory steps for carrying out the plan include: Checking the alarm, preparing a checklist to make sure he or she has everything needed for a shower, helping the person to select the clothes he or she will wear the next morning, and placing them on a chair near the bed. People experiencing difficulty following the preparatory steps may need help from their caregivers.

Calendars: Calendars are useful to keep track of scheduled actions such as appointments, activities, etc, but also can be used as triggers to start a scheduled activity and follow a scheduled action. For example, a note on the calendar may remind a person to call the pharmacy for a repeat prescription. To minimise distractions, the pharmacy's telephone number and the details of the repeat prescription may be put on the calendar or on a sticky notepaper attached to the calendar. PATH therapists must review the existing calendar and check whether they have been used effectively. If the person is so impaired that he or she cannot learn how to use a calendar effectively, the caregiver may need to provide help. People with difficulty starting tasks and following through may welcome this structure.

Filing tools: Instructions, checklists, and other documents may be kept in folders for the person with dementia to use when needed. A table of contents for each folder should be placed in a visible place near the folders. Organising the folder and creating a table of contents may be a challenge for those with advanced cognitive impairment and may require the help of the family, significant other, or professional caregiver.

Motivational tools

Motivational phrases: Selected motivational prompts may help people start tasks that they neglect e.g. "taking a shower in the morning makes me feel better during the day." For those whose writing and reading abilities are impaired, the caregiver may assist in preparing motivational prompts.

Notebooks: Memory notebooks may be used by people with dementia, whose writing and reading abilities are relatively intact, to remind themselves of important details, as well as the sequence of actions in a specific activity. The person must have a notebook that he or she uses to keep notes, writes questions, and completes homework. For example, the person may write down important procedures like the steps necessary to refill his or her medications. Appropriately placed notes with directions may reduce the need for attention. For example, keeping a notebook of instructions on how to use the washer and dryer may be kept near the washing machines along with a sign "look at the notebook for instructions." Notebooks can be used in conjunctions with checklists, which help the person complete all the steps of the instructions. Notebooks are also important to have during doctors' appointments.

Diaries: People with dementia whose writing and reading abilities are relatively intact may utilise diaries to keep track of thoughts and emotions regarding a specific behaviour. For example, "Today, I spoke with my friend Susan. Whenever I call her, my mood is lifted." Another person may keep positive experiences and statements to review when he or she feels depressed in the diary. For people with impaired reading and writing abilities, the caregiver may help write the person's thoughts and emotions in the diary. Reading them may motivate a person to start desirable actions.

[Return to Table of Contents]

Appendix 3: Safeguarding guidelines

As with any health condition in which a person is reliant on another for the provision of care, there is the potential for safeguarding issues to arise during the course of therapy. This may occur in the form of disclosure or suspicions about any type of abuse experienced by the person with dementia and depression. If such issues arise then please ensure that you do the following:

1) **Assess** further.

2) **Continue** with the session, if possible. Alternatively, offer another session of modified PATH if the whole session has been put on hold due to assessing these issues (though this should be rarely necessary).

3) **Follow the local safeguarding policy**. Call the person with dementia's care coordinator in the Memory Service/CMHT or GP and inform them of the safeguarding issue. Ask them to speak to the person with dementia and/or caregiver. If you are not routinely part of the Memory Service/CMHT and are only seeing the person with dementia as part of the PATHFINDER trial, ensure that the Memory Service/CMHT or GP understand that you are only seeing the person with dementia in a research capacity and that the Memory Service/CMHT or GP have responsibility for the care of the person with dementia.

4) **Inform the research team** as this may need to be reported as an adverse event or serious adverse event depending on the nature of the disclosed event.

[Return to Table of Contents]

Appendix 4: Case examples

Data from the preliminary study of PATH found that people who are depressed, cognitively impaired, and disabled were concerned with the following problems/issues: 1) lack of pleasurable activities; 2) social isolation/loneliness; 3) safety and health related issues (including activities such as taking medication and keeping doctors' appointments); 4) tension with others (e.g. caregiver, other family members, or friends); and 5) problems with instrumental activities of daily living (including doing laundry, shopping for groceries, managing money, using public transport, and using the telephone). The following examples focus on these problems. They are not intended to describe the course of PATH treatment – they are a guide as to how PATH could be implemented rather than a prescription. They are selected to highlight how you could integrate tools to support memory, attention and planning and the involvement of family or other caregivers to improve emotion regulation in depressed elders with cognitive impairment and disability. The negative emotions and the emotion regulation strategies that the therapist used in these examples are highlighted.

1. LACK OF PLEASURABLE ACTIVITIES

- Identify the reasons for giving up pleasurable activities (e.g. physical disability, anhedonia, fatigue, etc), so that you can decide which tools to support memory, attention and planning might be helpful. For example, the person with dementia may report lack of interest and inability to experience pleasure, memory and organizational difficulties, and mobility problems (use of wheelchair) as the reason for not engaging in pleasurable activities. *Negative emotions: anhedonia, feeling discouraged.*
- Create a list of pleasurable activities that the person with dementia enjoyed in the past *("Select the best situations").*
- Enrich the list of previous activities with new activities that the person with dementia may be able to perform given her limitations. Explore the interest and ability of a family or other caregiver in helping *("Change the situation").*
- Provide the person with dementia with a list of techniques to distract himself when she feels discouraged *("Change the situation", "Shift attention").*
- Help the person with dementia and the caregiver evaluate each activity and select the best two based on the meaning to the person with dementia and feasibility. For example, the person with dementia may select to go on a matinee movie on Saturdays and to have brunch at a local restaurant on Sundays *("Change the situation").*
- Inform the person with dementia of potential resistance to following the plan. Explain repeatedly that it is essential to follow the plan despite her inclination to avoid it. Inform the person with dementia that having these activities regularly will eventually increase her interest and her ability to experience pleasure. Ask the caregiver to encourage the person with dementia at the right times. Establish that the nearby multiplex cinema has wheelchair access and identify a few restaurants with wheelchair access. Help the person with dementia mark her calendar and include telephone numbers. Encourage the person with dementia and the caregiver to use the calendar and phone numbers to identify a movie and make restaurant reservations *("Change the situation").*
- Schedule the activity to start right after the session, if necessary.

2. SOCIAL ISOLATION/LONELINESS

- Divide the problem into smaller, more manageable and focused problems. For example, if the person with dementia feels isolated from her friends, this problem may be divided into 3 smaller problems: calling the friends, arranging a meeting with them, and preparing to go to the meeting. In this example, we will target the first two problems (calling the friends and arranging the meeting). *Negative emotions: sadness, hopelessness, irritability, anxiety.*
- Find more information about the person's friends (e.g. names) and their relationship with the person with dementia; find out more about the activities that he used to do with them. Explore reasons for the person with dementia's lack of contact with friends (e.g. physical limitations, lack of initiative, memory and attention difficulties, disorganisation, inability to experience pleasure, lack of energy), so that you can decide which tools to support memory, attention and planning might be helpful. In this example, the principal obstacles were the person's distractibility and forgetfulness, as well as his poor eyesight that made reading of telephone numbers difficult. These limitations prevented him from making phone contact with her friends *("Select the best situations").*
- Explore the personal meaning to the person with dementia of re-establishing contact with each of his friends. In this case, the person with dementia felt that he was not worthy of his friends' attention because of his disabilities.

Help the person with dementia to develop a different perspective and highlight the positive aspects of himself. Help him to distract himself when feeling worthless, and encourage him to engage in pleasurable and rewarding activities. When the therapist provided examples of his friends' sustained interest in him, he agreed to work on this problem *("Change the situation", "Change perspectives", "Shift attention").*

- Explore the caregiver's ability and interest in helping with this problem. In this case, a professional caregiver was willing and available to help.
- Ask the person with dementia, with the help of the caregiver, to create: a) a written list of his friends and telephone numbers; and b) a list of possible meeting places and times. The list should be on large prints because of the person with dementia's poor eyesight *("Select the best situations", "Change the situation").*
- Help the person with dementia put priority numbers on the list of friends and meetings *("Change the situation").*
- Ask the caregiver to get the person with dementia a telephone with larger-printed buttons *("Change the situation").*
- Help the person with dementia decide whom to call and how to try to arrange a meeting. Role-play if necessary *("Change the situation").*
- Help the person with dementia select the days that he will make the phone calls and encourage the person with dementia to call back if he fails to make contact on the first attempt *("Change the situation").*
- Ask the caregiver to encourage the person with dementia to use a large print calendar on a daily basis. Place a sign on the calendar to remind the person with dementia when to make the phone calls. The caregiver will also remind the person with dementia, when necessary.
- Create a list with the telephone numbers of the friends to call. The caregiver and the person with dementia may create a sign with the meeting times prioritised.
- Encourage the person with dementia to make arrangements for subsequent meetings with the friend during the first meeting. The person with dementia and the caregiver will create a plan to follow before the meeting (e.g. arrange transportation, take medication) (*"Change the situation").*
- If during the first meeting the person with dementia fails to make plans for subsequent meetings, place a reminder in their calendar indicating the need for them to make a follow-up phone call.

3. *SAFETY AND HEALTH-RELATED ISSUES*

<u>Taking medication inconsistently</u>

- Explore the reasons for the problem, so that you can decide which tools to support memory, attention and planning might be helpful. For example, what happens when the person with dementia forgets to take medication? Are they distracted by another task? Is the failure to take medication a result of disorganisation evident in other activities? Are there specific medications that the person does not take? Are the medications that the person forgets to take giving him/her side effects? Does the person have idiosyncratic beliefs about any of his/her medication? Evaluate the family caregiver's ability and interest in helping to find a solution to this problem. The person with dementia or a caregiver should contact the person with dementia's GP and ask whether the medication instructions can be simplified. <u>Negative emotions:</u> *frustration, irritability, anxiety, helplessness.*
- Brainstorm solutions with the person with dementia and the caregiver, if necessary, and help the person with dementia to evaluate each solution and select the best solution. For example, the best solution might be to buy a medication kit. The caregiver may fill the kit with medications once a week *("Change the situation").*
- Discuss with the person with dementia the details of the solution and how it will be implemented. Prepare signs (or use a voice recorder in case of poor eyesight) to remind the person with dementia to take the medication at specific times *("Change the situation").*
- Ask the caregiver to buy the medication kit and place the signs at appropriate places.
- Encourage the person with dementia to use a calendar of activities on a daily basis. Create and place the signs in a visible place to remind the person with dementia when to take the medication (e.g., a sign on the mirror reminding the person with dementia to take the morning medication, a sign on the night table asking whether the evening medication was taken). Establish a routine for the person with dementia to bring the medication kit to the table during breakfast and dinner.
- Enlist the family caregiver to record whether the medication has been taken.

<u>Difficulty in managing repeat prescriptions</u>

- Find out what happens when the person with dementia fails to get his repeat prescriptions in time and why this might be occurring, so that you can decide which tools to support memory, attention and planning might be helpful. Does the person with dementia have repeat prescriptions? Is the person with dementia's GP responsive to the person with dementia or family's calls? Does the person with dementia inspect the medication bottles regularly? Does the person with dementia procrastinate? For example, the principal obstacles might be the person with dementia's distrust of medication and his forgetfulness in calling the GP on time. *<u>Negative emotions:</u> frustration, irritability, sadness, helplessness.*
- Write down the names of the medications, and when repeat prescriptions need to be requested; write down the phone number of the GP, and the phone number of the pharmacy used by the person with dementia. Evaluate the caregiver's ability and interest in helping *("Change the situation", "Select the best situations").*
- Create a written list of possible solutions that may help the person with dementia get the repeat prescriptions in time *("Change the situation", "Select the best situations").*
- Create a list of possible ways to get his repeat prescriptions from the pharmacy; the spouse or a caregiver may be involved *("Change the situation", "Select the best situations").*
- Clarify the role of each medication for the person with dementia. Advise the caregiver to work with the person with dementia to enter the repeat prescription dates on the person with dementia's calendar; the caregiver may remind the person with dementia five days before he needs to request his repeat prescriptions and, if necessary, call the GP himself/herself *("Change the situation", "Select the best situations").*
- Prepare a taped message that mentions all the steps for requesting repeat prescriptions, which is to be used on the dates that repeat prescriptions need to be requested. Prepare a sticky notepaper to be attached to the calendar to remind the person with dementia to use the pre-recorded message at a specific time in the morning. Role-play a phone call to the GP, if necessary *("Change the situation").*
- Ask the person with dementia to report to you whether he was able to request his repeat prescriptions.

<u>Missing doctors' appointments</u>

- Explore the specifics of the problem, so that you can decide which tools to support memory, attention and planning might be helpful. Is the person with dementia unable to get ready for the appointments due to disorganisation? Is their family involved in setting up appointments? For example, the principal obstacles might be the person with dementia's mobility problems (has to use a walker), hopelessness, inability to initiate new tasks, and memory difficulties. <u>Negative emotions:</u> *anxiety, sadness, anger*.
- Prepare a list of the medical conditions that need to be followed. Include how often they need to be followed, and the names, addresses, and phone numbers of the physicians responsible for the care of these conditions. Place the list in a folder and put the folder in a drawer; label the drawer "Medical Information." Evaluate the caregiver's ability and interest in helping *("Change the situation", "Select the best situations").*
- Explore how specific thoughts in relation to hopelessness interfere with keeping appointments. Help the person with dementia create a list of possible solutions that may help him keep his next appointments (i.e. calling the doctor, making the appointment, arranging the transportation, and making sure to keep the appointment) *("Change the situation").*
- Identify assumptions that lead to his disinterest in seeing his doctors and encourage exploration of the pros and cons of each assumption. Through the exploration, help the person with dementia see a realistic view of his medical prognosis. Emphasise that, even if he feels hopeless, his medical prognosis would improve if he receives appropriate medical care. Help the person with dementia use techniques to reduce feelings of hopelessness when they occur *("Change perspectives", "Manage emotions").*
- Remind the person with dementia to write the appointments on the calendar. Invite the caregiver to help the person with dementia arrange transportation. Prepare a timed list of activities on the day of each appointment to help the person with dementia keep his appointments *("Change the situation").*
- Discuss with the person with dementia and the caregiver the details of the solutions and how they will be implemented. If the person with dementia and family found it difficult to negotiate appointments with a physician's staff, arrange that the person with dementia may call the physician during the next session. Discuss ways to reduce frustration or anger if the plan doesn't work out *("Change the situation", "Select the best situations", "Manage emotions").*

- Prepare a written timed list of activities that need to precede the appointment. Caregivers may need to be involved. When necessary, the caregiver may need to contact the physician's office, inform the staff of the difficulties the person with dementia is experiencing, and advise the staff of ways to facilitate communication with the person with dementia *("Change the situation").*

4. *TENSION WITH OTHERS*

<u>Tension with spouse or significant other</u>

- In this example, the person with dementia has tension with his wife because according to the person with dementia "they are not doing things together anymore." Gather more information about the problem (e.g., identify what they have done together in the past but they do not do anymore). Try to identify the reasons for the problem (e.g. physical limitations, inability to get organised, memory and attention difficulties, lack of interest, fatigue, hopelessness), so that you can decide which tools to support memory, attention and planning might be helpful. In this example, poor organisational skills and poor night vision in both the person with dementia and the spouse limit their ability to drive. *<u>Negative emotions:</u> anger, sadness, worthlessness, hopelessness, guilt.*
- Explore the personal meaning and consequences "of not doing things together" to the person with dementia and spouse. For example, the person with dementia feels that resuming common activities with his spouse will revitalise their relationship even though he has doubts that the spouse will agree to resume these activities. Help the person with dementia with different techniques to reduce his discouragement about finding a solution to the problem. Communicate your understanding to the person with dementia and try to raise the person with dementia's interest to work with you on this problem (*"Select the best situations", "Change perspectives", "Manage emotions").*
- The person with dementia mentions that he feels that his wife doesn't care about him anymore. He has been very hurt by her behaviour and he wants to withdraw and not do anything. Discuss different perspectives with the person with dementia to help him evaluate whether the way he perceives the situation is accurate *("Change perspectives").*
- Explore the spouse's ability and interest in helping with this problem. Create a list of possible choices for shared activities. Discuss with the caregiver how to reduce her frustration with the problem *("Change the situation", "Change perspectives", "Manage emotions").*
- Evaluate each choice (writing pros and cons) with the person with dementia and the spouse and together identify the most meaningful and feasible activities.
- Help them decide on a single specific activity (e.g. having lunch out twice a week). Encourage them to reorient themselves and value daytime activities that do not require night driving. Set up a time and date that will be convenient for both of them and make a timed list of steps to be followed by the person with dementia prior to going out for lunch (e.g. time to take a shower, time to dress, time to comb hair, etc). With the person with dementia's agreement ask the spouse to encourage the person with dementia to adhere to the list *("Change the situation", "Select the best situations").*
- Encourage the person with dementia to use a calendar. Create a reminder for the person with dementia to call and make reservations. Ask the person with dementia and the spouse to write down the appropriate telephone numbers and place them near the phone. Create a sign and place it on the calendar to remind the person with dementia of the time to start following the calendar of activities necessary for getting ready for lunch out. Help the person with dementia utilise a voice alarm to remind him/her to get ready an hour before leaving the house (*"Change the situation").*
- Help the person with dementia to write in a notebook about pleasant thoughts and emotions experienced when he went out with his spouse in the past. Encourage the person with dementia to use the notebook as a motivational tool when he loses interest in activities *("Change perspectives").*

<u>Tension with adult children</u>

- In this example, a depressed mother, with significant cognitive deficits, feels that she "is not doing house chores" to help her adult daughter. As a result, the daughter, who is working full time, feels overwhelmed and states that her mother is not "willing to help her even though she can".
- Try to understand the reasons for the problem (e.g. attention and concentration difficulties, memory deficits, disorganisation, procrastination, physical limitations, lack of interest or energy), so that you can decide which tools to support memory, attention and planning might be helpful. For example, the principal obstacles could be

lack of energy, difficulty selecting a task, and difficulty remembering doing it. Negative emotions: *worthlessness, sadness, irritability, guilt.*

- Explore the personal meaning and consequences "of not doing house chores" with the person with dementia and her daughter. In this case the person with dementia felt that her lack of contribution to house chores burdened her daughter. Communicate your understanding to the person with dementia and try to raise the person with dementia's interest in working with you on this problem. Help the person with dementia see the daughter's point of view and provide some examples to help her do that. Further, educate the caregiver about the person with dementia's depression and cognitive difficulties and explain that even though the person with dementia may want to help, she may lack the motivation or interest due to depression *("Change the situation", "Change perspectives").*
- Explore the daughter's ability and interest in offering help with this problem. Discuss the person with dementia's difficulties with the daughter, and explore the pros and cons of the tension. Try to help the daughter see the mother's vulnerabilities. Write down the chores that the person with dementia would like to do *("Change perspectives").*
- Help the person with dementia and her daughter to evaluate (writing pros and cons) the appropriateness and feasibility of each chore.
- Help the person with dementia and her daughter to use techniques to avoid escalation of conflict and reduce irritability when the tension escalates (*"Change the situation", "Select the best situations", "Manage emotions").*
- Help the person with dementia select a single chore and enlist her daughter's help. In this case the selected chore was to vacuum two rooms of the house twice a week.
- Help the person with dementia decide on the specific days, times and specific rooms to vacuum and how long it would take. Create a fallback plan to be used if the person with dementia's first attempt fails (e.g. an alternative day and time) *("Change the situation").*
- Help the person with dementia write down the reasons that make vacuuming important and use this approach to increase the person with dementia's motivation. Ask the daughter to remind the person with dementia that vacuuming is planned ahead of time and help the person with dementia with preparations to vacuum. Ask the person with dementia and the daughter to think about distractions that may capture the person with dementia's attention during vacuuming. Remove potential sources of distractions (e.g. furniture that are in the person with dementia's way, clutter, etc) *("Change the situation", "Select the best situations").*
- Prepare a timed step-by-step plan for the person with dementia to follow prior to vacuuming. Encourage the use of a calendar on a daily basis and help the person with dementia enter in the calendar the dates and times of vacuuming, as well as the steps needed prior to actual vacuuming.

5. *PROBLEMS WITH INSTRUMENTAL ACTIVITIES OF DAILY LIVING*

Difficulty in managing money

- In this example, the person with dementia does not pay bills on time. Try to understand the consequences of not paying bills on time and the personal meaning of this problem to the person with dementia. In this case, the person with dementia indicates that failing to pay bills is anxiety producing and that asking others to undertake this function is humiliating for her. Negative emotions: *anxiety, worthlessness, irritability, hopelessness.*
- Try to understand barriers to paying bills e.g. inability to sort the mail, lack of structure, forgetfulness, etc), so that you can decide which tools to support memory, attention and planning might be helpful. In this case, the principal obstacles are the person with dementia's lack of organisation, memory problems, and inability to sustain attention when she starts paying the bills. Help the person with dementia with techniques to reduce irritability when she can't pay the bills. Help the person to distract herself from negative emotions and concentrate on positive aspects. Explore the interest and ability of a caregiver to help *("Change the situation", "Change perspectives", "Manage emotions", "Shift attention").*
- Brainstorm solutions with the person with dementia and the caregiver, if necessary, and help the person with dementia to evaluate each solution and select the best solution. In this case, the person with dementia selects to create a written list of activities necessary for paying bills (e.g. set up a time to sort the mail on a daily basis, identify a place where bills are placed, record in the calendar dates and times for paying bills, write a check for each bill and keep a record of the cheque number and the amount) *("Change the situation").*
- Discuss with the person with dementia and the caregiver about the implementation of the solution.

- Prepare the necessary material that may be used during the implementation of the plan (e.g., encourage the person with dementia to use her calendar, place on the refrigerator the list of activities required for paying bills). The caregiver may supervise the implementation of the solution, if necessary.

Difficulty initiating or returning phone calls

- Interview the person with dementia and the family to identify barriers to this behaviour (e.g., does the person with dementia write down that he got a message? Is the person with dementia distracted and does not return phone calls?) Use this knowledge to decide which tools to support memory, attention and planning might be helpful. Try to understand what the person with dementia experiences as consequences of his failure to use the telephone appropriately and make the person with dementia aware of your understanding. For example, the principal obstacles could be the person with dementia's lack of interest in socialisation, memory deficits, and difficulty in planning, initiating and carrying on a conversation. *Negative emotions: worthlessness, helplessness.*
- Explore the interest and ability of a caregiver in helping. Work with the person with dementia and the family to create a list of the phone calls that need to be initiated or returned during the next week and identify days and times for doing so *("Change the situation").*
- Encourage the person with dementia to write in his diary the reasons for calling friends and describe positive feelings from past interactions. Help the person with dementia to remind himself of these past positive interactions when he feels worthless. Ask the caregiver to be involved, if necessary. *("Change the situation", "Change perspectives").*
- Help the person with dementia use strategies to distract himself when negative feelings arise. Encourage the caregiver to gently remind the person with dementia about distraction strategies *("Shift attention", "Manage emotions").*
- Create a list of possible solutions that will help the person with dementia to remember to return the phone calls on time *("Change the situation").*
- After review of the proposed solutions, the best solution was recording all missed calls and keeping the telephone messages next to the telephone, setting up the same time every day to return telephone calls, making a list every morning of telephone calls that need to be initiated, and preparing a list of issues or conversation topics that need to be communicated during the calls *("Change the situation").*
- Make a dated plan assigning specific actions to the person with dementia and discuss the plan with the person with dementia and the caregiver in concrete detail. Place the friend's picture near the phone to serve as a motivator for the person with dementia to call his friend. Ask the person with dementia to review his diary to increase his motivation on the planned day of contact.
- Arrange so that the person with dementia will make the phone calls during the session, if necessary. Role play, if necessary.

Difficulty maintaining former key roles

- In this example, the person with dementia was a leader in his church and worked with many voluntary organisations before he developed dementia, which gave him a real sense of purpose in his life. Since he became more forgetful and confused, he struggled to keep doing these things and eventually had to stop. Now he feels that he is unable to support or be useful to anyone, and feels very sad and hopeless about this. As a result of his negative feelings about himself, he has withdrawn and doesn't participate in church activities. *Negative emotions: worthlessness, sadness, hopelessness.*
- Identify the triggers of the negative emotions. In this example, the person with dementia was feeling worthless and sad during late afternoon or early evening when he was not busy.
- Develop a plan to keep the person with dementia busy during the times when they are at risk of having these negative emotions. In this case, the person with dementia and his wife decided that a good way to reduce feelings of worthlessness and sadness during this time was for him to listen to his favourite music and to watch his favourite TV program. When he practiced this strategy, he realized that keeping himself busy was helping to reduce these negative feelings *("Select the best situations", "Shift attention").*
- Help the person with dementia to focus on what he can do rather than what he cannot do. In this example, the person with dementia was also feeling worthless because he didn't have any significant position in his church. As a result, he had withdrawn from all church activities and isolated himself from the church community. As part of a strategy to increase his pleasure and reduce his feelings of worthlessness and sadness, the person with dementia

and his wife decided to participate in church activities every Sunday. The person with dementia practiced self-talk when engaging in church activities ("Any help is appreciated, no matter how small", "I can do…"), rather than focusing on what he could not do. After implementing this plan for a couple of weeks, the person with dementia reported a significant reduction in feelings of worthlessness and sadness *("Change perspectives", "Select the best situations")*.

[Return to Table of Contents]

Appendix 5: Issues to discuss in therapist supervision

TRIGGERS - Problems or situations that lead to negative emotions:

NEGATIVE EMOTIONS - Negative emotions associated with the problems or situations:

TECHNIQUES - Strategies for reducing negative feelings and increasing positive ones:

Are there any difficulties or issues arising in the delivery of the intervention?

Is there any tension between the person with dementia and the caregiver?

Have there been any reports of suicidal ideation (with or without plans or intent)? If yes, what?

Are there any safeguarding concerns? If yes, what?

Has a personalised treatment summary been developed for this participant?

If yes, please bring it with you to supervision and discuss it with your supervisor.

Appendix 6: PATH cheat sheet - a reminder of the key principles of PATH

- Stay focused on emotions.
    - The focus of PATH's simplified problem solving approach is to reduce negative emotions that are associated with the specific problem, and promote positive emotions, as shown in the figure below.

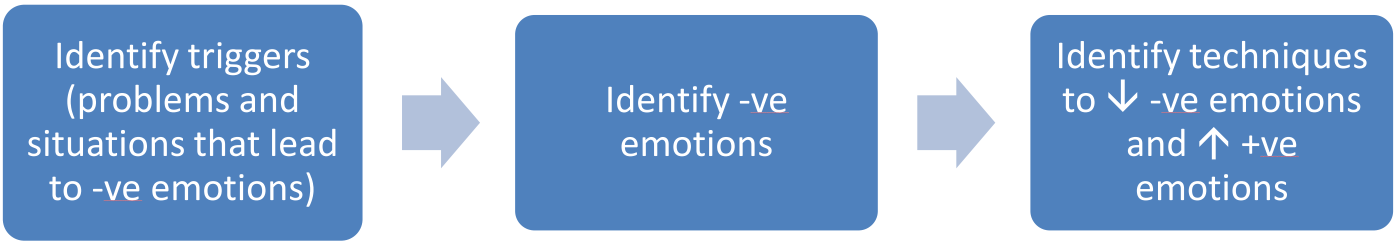

- Stay focused on the person with dementia.
    - Help the caregiver to feel listened to by having a short discussion (5 mins) with them after the session has ended, with the consent of the person with dementia.
    - Refer the caregiver for psychological support in their own right, if necessary.
- Adapt how you engage the person with dementia.
    - Use Type A prompts for those with milder dementia and Type B prompts for those with more moderate dementia. For example:
        - Type A: "What's getting in the way of you being able to do the things that you used to enjoy?"
        - Type B: "A number of things can get in the way of us doing the things that we used to enjoy. Are any of the following things getting in the way of you doing things you enjoy?" <Show list of common obstacles or barriers.>
- Set an appropriate pace for the sessions.
    - Remember that it may only be possible to address 2-3 problems if the pace is slower or the problem is more complex.
    - Be aware that the pace may need to be slower on 'bad days' with respect to memory, confusion or mood.
- Use behavioural activation techniques to help people with dementia engage in pleasurable activities. Use worksheets for participants as and when they are appropriate:
- Address tension between the person with dementia and their caregiver.
    - i) Identify and define the tension;
    - ii) Avoid escalation of the tension (e.g. helping each person to see the other's perspective);
    - iii) Evaluate the pros and cons of the tension;
    - iv) Brainstorm and test out solutions for reducing the tension.
- Decide which tools to support memory, attention and planning should be used and how the caregiver should be involved by asking yourself:
    - i) Does the person with dementia have difficulty in learning the new procedures or techniques?
    - ii) Does the person with dementia have the ability to form a plan and follow sequential steps until its completion?
    - iii) Does the person with dementia have the ability to complete the home practice assignments?
- Use the person's own words for terms such as depression, dementia, home practice, etc.
- Use worksheets and handouts for participants as and when they are appropriate.
- Give brief summaries of what you discussed previously and what you've been discussing in the current session.
- Help the person with dementia and their caregiver to write things down.
- Encourage participants to complete home practice by:
    - Giving the rationale for the home practice;
    - Discussing possible obstacles or barriers to completing the home practice with the person with dementia and the caregiver;
    - Discussing how helpful the home practice might be for them;
    - Positively reinforcing any home practice that is completed.

A reminder of how to improve emotional regulation in people with dementia, and the different types of tools or strategies for supporting memory, attention and planning in PATH are presented in Tables 1-3.

Table 1: Examples of ways to regulate emotions.

Strategies	Examples
Select the best situations to expose the person to	1. Identify situations and activities that trigger negative emotions associated with depression and avoid these situations.
	2. Identify situations and activities that trigger positive emotions and promote these situations.
Change the situation the person is in	1. Identify tools to support memory, attention and planning to overcome functional limitations that trigger a strong negative emotional response in the person with dementia.
	2. The caregiver modifies these emotionally charged situations accordingly.
Shift or redirect attention	1. Use tools to support memory, attention and planning to overcome functional limitations and redirect a person's attention to positive aspects of life (e.g. through distraction).
Change perspectives	1. Help the person with dementia and caregiver (if necessary) to develop a realistically hopeful approach to functional and cognitive limitations (e.g. cognitive impairment doesn't necessarily prevent them from enjoying life; focus on what they can do rather than what they can't do; tools to support memory and attention may reduce functional limitations).
Manage emotions	1. Help the person with dementia and caregiver (if necessary) to use skills for managing emotions during emotionally charged situations (e.g. using techniques to reduce escalation of tension between the person with dementia and caregiver).

Table 2: Specific techniques for regulating specific emotions in people with dementia and depression.

Emotion	Technique(s)	Rationale / Suggested approaches
All	• Explore the pros and cons of the emotion in order to see whether it is helping the person to feel better<br>• If it is not, then use self-talk to shift or redirect attention to something else	"It's always helpful to examine the usefulness of our feelings and the effect that they have on us. If we realise that our feelings are not helpful to us, we should try to find ways to modify them. For example, when we're feeling upset, it is important to think about whether what we're feeling is helping us to feel better in our mood. If it is not, then reminding ourselves of this can be useful. For example, it can be useful to tell ourselves "This feeling is not helping me feel better...let me do X instead".
Sad or depressed	• Select the best situations by establishing a structured daily routine and scheduling pleasant activities within it (see "Activities that I enjoy doing" and "Calendar of Activities" worksheets)	• "When we feel sad or down we often feel like not getting out of bed, staying at home or isolating ourselves from others. However, this is not helpful for two reasons. One, because it means that we have more time to think about the things that are upsetting us. And two, because we have fewer opportunities to experience pleasure. Therefore, what we need to do is help you to start doing more things that give you pleasure in order to help you feel better."<br>• "Sometimes we think that if we engage in a particular activity, we will not enjoy it. As a result, our motivation to engage in activities is reduced. To overcome this hurdle, we need to engage in activities even if we think we won't enjoy them. Don't wait to feel motivated to engage in activities; engage in activities to increase your motivation."
Bored or lonely	• Select the best situations by establishing a structured daily routine and scheduling pleasant activities within it (see "Activities that I enjoy doing" and "Calendar of Activities" worksheets)	• "Not doing much each day can make you feel bored or lonely and even lower in mood. What we need to do is create more structure in your daily life in order to keep you busy and distract you from feeling lonely or bored."
Tension with caregiver	• Shift or redirect attention to positive aspects of the patient/caregiver relationship<br>• Change perspectives by encouraging them to look at the situation from the other person's perspective (if possible)	• "When we're feeling upset with another person, it's easy to focus on the negatives. However, this can make us feel even lower in mood. Instead, it is more helpful to focus on the positives in that relationship. It is also helpful for us to try to look at the situation from the other person's viewpoint."<br>• "It's always helpful to set aside our emotions – whenever we can – and think about the usefulness of having tension with our spouse/children/friend. How is this helping us? And if it's not helping us, how can we reduce it?"

Emotion	Technique(s)	Rationale / Suggested approaches
Anxious, frustrated or angry	• Shift or redirect attention to something else or manage emotions by soothing oneself (see "List of strategies for distracting or soothing myself" worksheet)	• "When we're feeling anxious/frustrated/ angry, we tend to focus on the things that are making us feel this way, which can make us feel even worse. What we need to do in these situations is do something that either distracts us or that calms us down."
Feeling hopeless or useless (e.g. due to thinking "I can't do anything anymore")	• Change perspectives by exploring what the person with dementia can still do and shift or redirect attention to this (e.g. "I can still do...")<br>• Change the situation by exploring new ways of doing activities using tools to support cognition and help from others<br>• Shift or redirect attention to something else (see "List of strategies for distracting or soothing myself" worksheet)	• "It's easy to focus on the negatives such as all the things we can no longer do when we're feeling down. However, this can make us feel even lower in mood. Instead, it is more helpful to focus on the positives and what we still can do. It is also helpful to look at what tools we can use or what support we can get from others to help us do things."<br>• "When we feel useless or bad about ourselves, we may ask ourselves "How is this helping me?", "What do I gain by being hard on myself?" We might reflect that it will probably make us feel worse, it will not help us experience pleasure in our day-to-day life, and it will affect our relationships. Saying to ourselves "Let me try to distract myself from these negative feelings by focusing on something positive, like engaging in a pleasurable or rewarding activity" can be helpful."
Rumination	• Shift or redirect attention to positive aspects of the situation (e.g. shift attention to positive memories of one's husband rather than ruminating about negative memories) or to something else (see "List of strategies for distracting or soothing myself" worksheet)	• "When we keep ruminating or dwelling on things that have happened in the past, this can make us feel upset and even lower in mood. Instead, it is more helpful to shift our attention to more positive aspects of the situation."<br>• "It is important to try to reduce rumination or dwelling on things as soon as it starts; so, whenever we notice that we have started ruminating, it's a great idea to shift our attention away from it. It's helpful to have created a list of distraction strategies so we will be ready to distract ourselves when we need to."
Lacking motivation or not feeling like doing anything	• Select the best situations by establishing a structured daily routine and scheduling pleasant activities within it (see "Activities that I enjoy doing" and "Calendar of Activities" worksheets)	• "Feeling down affects the way we think. We tend to think in a negative way when we're feeling down. For example, we may think that we need to feel like doing something before we can do it. In fact, the opposite is true. We need to do start doing something before we can feel like doing it."

Table 3: Types of tools or strategies for supporting memory, attention and planning in PATH.

Tool/strategy	Examples
Memory aid	Visual aids: Calendars, clocks, coloured tags, daily checklists, diaries, magnetic notepads, markers, medication kit, notebook, pictures, signs, sticky notepapers and reminders<br>Auditory aids: Alarms, alarm clocks, beeping watches, computerised phone calls, customised audiotapes, key-chain recorder, timers, timed pre-recorded messages
Keep attention on the task	Providing positive social reinforcement (e.g. encouragement, praise, rewards) to keep a person's attention on the task - usually done in conjunction with minimising distraction.
Minimise distractions	Reducing clutter in the environment, turning off the TV or radio when completing a task, completing a task when one is least likely to be disturbed
Break a task down into smaller steps	Break 'cooking a meal' down into smaller steps → get all the ingredients, put all the ingredients into the pan in the right order and right amount, and set the timer on the stove
Specify the preparatory steps for a task	Preparatory steps for cooking a meal → get a cookbook specifying the right amount of ingredients and the right order, put all the necessary ingredients in a specific place, make the measurements easily accessible to the person with dementia, and get instructions for the oven
Tools for filing	Folders in which instructions and checklists for completing certain tasks are kept

[Return to Table of Contents]

Appendix 7: Examples of problems faced by people with mild to moderate depression and dementia

Some common problems identified in our interviews and focus groups with people with mild to moderate dementia, caregivers and healthcare professionals, which may also be raised in the modified PATH intervention are listed below:

Medication side effects:
Several people with dementia expressed that medication side effects were causing some of the biggest problems in their lives. Problems identified included medication making them feel drowsy, making confusion worse, and causing slurred words.

Being cared for too much:
Many people with dementia talked about not being allowed to do things by their families/caregivers even though they felt they were quite able to. Being made to "just sit down" while others made them tea, cleaned, or did things that the person with dementia used to do was identified as a key contributor to low mood by most people with dementia.

Loss of key life roles:
Losing or having to give up important life roles is a major contributing factor in depression in people with dementia. Losing roles as church leaders, volunteer workers, or paid work which was key to their identity, all came up and was recognised as a major problem by both people with dementia and their caregivers.

Where traditional gender roles had played a key part in people's lives, the reversal or loss of these, such as women being unable to take care of the house and their husbands, or men being unable to be the provider of the household, was considered to cause depression and exacerbate low self-esteem and feelings of hopelessness.

Loss of function and structure to the day:
As a result of being unable to do things they would normally do, or having family members trying to help too much, people with dementia often ended up sitting watching the television all day. Many acknowledged that this made them despondent and worsened their mood and outlook.

Physical health problems and lack of mobility:
Many people with dementia also have significant comorbid health concerns that leave them in chronic pain and with significantly reduced mobility. When people have these kind of problems, they typically talk about just wanting their physical health to be better - if it were then everything else would be manageable.

Physical health problems also impact on many other problem areas, such as social functioning, general activity levels, sleep, and irritability.

Difficulty maintaining friendships:
Struggling to concentrate on one conversation when there is lots of background noise, and general difficulties focusing, mean that people with dementia often find it harder to meet friends in the same way they used to, such as at cafes, pubs, or in groups.

Problems with confusion and getting lost prevent many people from going out to places alone, and restrict their social lives as they depend on others for transport, and often don't want to have to ask for this.

Some people reported being embarrassed about their forgetfulness or irritability, or about not having anything to talk about because they don't do anything, so avoided seeing friends because of this.

Being unable to do favourite activities:
Memory and concentration problems meant that some people have had to stop doing things they loved. One person who was an avid reader found she could no longer read a whole book anymore and was very upset about this, and another was unable to do jewellery making anymore.

Changeable moods:
Many people reported that they just wake up feeling depressed, and are unable to attribute it to any particular problem or trigger. It was also common for mood to change and get better or worse throughout the day, again with people having difficulty identifying triggers or warnings that they are starting to feel worse.

Relationship tensions:
Personality changes as a result of dementia were thought by many people with dementia and their caregivers as being a key contributor to relationship tensions. The person with dementia was generally more short-tempered and irritable since developing memory problems, and their caregivers were often on the receiving end of snappiness and irritability.

People were usually aware that spending too much time together (e.g. if they were both in the house all day every day) was unhealthy for their relationships and led to them becoming more fed up and irritable with each other.

Frustration with feeling misunderstood or not listened to often resulted in people with dementia becoming more irritable with caregivers.

Both caregivers and people with dementia often tend to withhold information from each other to prevent the other worrying. This lack of communication can lead to the person with dementia feeling more alone, and frustration on both sides because they are unsure what the other person is thinking or feeling. The result is often irritability from both the person with dementia and their caregiver as they try to guess what the other is thinking and how to manage/respond to this.

Several people with dementia expressed disappointment with how family members were caring for them, while their family members reported that they felt they were doing too much. Disparity between these expectations can lead to tensions in the relationship between the person with dementia and their caregiver and is a key way in which relationship tensions present.

Mood being dependent on things out of their control:
Many people with dementia talked about their mood being dependent on external factors, such as the weather or children/grandchildren not visiting enough.

Meta-problems (problems that contribute to other problems):
Lack of confidence – Awareness of memory problems, confusion, word-finding difficulties, and physical limitations contribute to an increased lack of confidence in performing activities, making decisions, and engaging in conversation.

General low motivation – Lack of motivation to do anything is a major symptom of depression in those with dementia. Even when they are able to do more activities of daily living, many people with dementia and depression struggle to find the motivation to do these and so family members step in to take over these and support them.

Suicidality – Many people with dementia, but particularly those who have had to give up significant roles in their lives, felt that they were just waiting to die and have daily thoughts of this. This can have a knock-on impact on motivation and willingness to engage in something to improve daily life.

Reduced reading and writing ability – With age and progressing dementia symptoms, many people found that their reading, writing and general fine-motor skills deteriorate. This impacts on many pleasurable activities and daily functioning, as well as affecting confidence and leaving some people with a sense of shame about their reduced abilities.

Fear of being a burden – This prevents many people from asking for support to do activities or outings, and contributes to relationship tensions as it leads to disagreements about what to do.

[Return to Table of Contents]

Appendix 8: Suicidal ideation guidelines

1) If there is a **positive response** to the question "Have you been feeling so bad that you have had thoughts about hurting yourself or others, that life is not worth living or that you'd be better off dead?", then **assess further** (e.g. "Can you tell me more about that please?"). Some example questions are below:

Questions	Details
What thoughts have you had?<br>How often have they been occurring?<br>How long do they last when they occur?<br>When have they been occurring?	
Have you made any plans?<br>If yes, what? How? When? Where?<br>Have you started preparing for this plan (e.g. stockpiling medication, finding out information)?	
Do you intend to carry out this plan? When? Where?	
What would stop you from carrying out this plan? What would stop you from doing anything to harm yourself?	
Do you have a history of harming yourself or attempting to kill yourself? Have you harmed yourself in the past couple of weeks (e.g. deliberately cut yourself)?	

2) If necessary, discuss **ways in which the person with dementia and/or their caregiver can get support** using the information sheet on the next page.

3) **Get consent** from the person with dementia and/or their caregiver to speak to their care coordinator in the Memory Service/CMHT or GP. The person with dementia and/or their caregiver will have already consented to this as part of the study. If they say no, then remind them of this and say you have a duty of care to inform their Memory Service/CMHT or GP.

4) **Continue** with the session, if possible. Alternatively, offer another session of modified PATH if the whole session has been put on hold due to assessing these issues (though this should be rarely necessary).

5) **Follow the local policy**. Call the person with dementia’s care coordinator in the Memory Service/CMHT or GP and inform them of the suicidal ideation. Ask them to speak to the person with dementia and/or caregiver. If you are not routinely part of the Memory Service/CMHT and are only seeing the person with dementia as part of the PATHFINDER trial, ensure that the Memory Service/CMHT or GP understand that you are only seeing the person with dementia in a research capacity and that the Memory Service/CMHT or GP have responsibility for the care of the person with dementia.

7) **Tell your PATH supervisor**.

8) **Inform the local research team** as any new reports of suicidal behaviour (including self-harm) during the intervention will need to be reported as a serious adverse event.

9) The central research team will discuss with the Trial Management Group as to whether the person with dementia should be withdrawn from the study and referred for more appropriate treatment, if necessary.

[Return to Table of Contents]

WHAT TO DO IN A CRISIS - INFORMATION FOR PARTICIPANTS

What to do in a crisis – getting help

At times, some people may feel so overwhelmed by the problems they are facing that they have thoughts about hurting themselves (or others) or that their life is not worth living. They may think that ending their own life (or the life of another person) is the only way out.

If you're having these thoughts or feelings, it is important that you tell someone so that you can get the appropriate support. The therapy sessions can be a time to share these thoughts or feelings and consider how you can be supported. Other ways of getting help are listed below:

Please fill in the table below so that you have a list of useful contacts in one place.

Things to do	Name and telephone number
Call your GP (during working hours)	Name:<br><br>Tel. no.:
Call your out of hours GP (if out of hours)	Name:<br><br>Tel. no.:
Call your keyworker or care coordinator in the Memory Service or Community Mental Health Team	Name:<br><br>Tel. no.:
Tell a friend or family member	Name:<br><br>Tel. no.:
Call the Silverline 24-hour helpline for older people (freephone)	0800 470 8090
Call the Samaritans 24-hour helpline (freephone)	116 123

IF YOU NEED IMMEDIATE HELP:
Do one of the following:
1) If you have a pendant alarm, press the buzzer.
2) Go to your GP.
3) Go to the Accident and Emergency Department of your local hospital.

IF YOU NEED URGENT HELP:
Call the Emergency Services on 999 if it is an emergency.

Appendix 9: PATH conceptualisation

The following questions may help conceptualise and guide treatment.

1. Current history of depression and cognitive impairment:
    a. What depression symptoms does the person with dementia have? How do they affect his or her functioning? Any suicidal ideation? When and under what circumstances? Is the person with dementia safe or in danger of killing himself or herself?
    b. How cognitively impaired is the person with dementia? How does cognitive impairment affect his or her functioning? When did it start? What are their cognitive strengths?

2. Past history of depression: Any coping skills that can be used in the current episode?

3. Medical illnesses: Any relationship with depression or impairment in functioning?

4. Medications: When did he or she start an antidepressant? What are the current medications? Has depression reduced due to antidepressant medications? Does the person with dementia remember to take his or her medications?

5. Current functioning: How are depression and cognitive impairment affecting his or her functioning? Please provide examples to show the relationship.

6. How available and willing to help is the caregiver? Any depression or cognitive impairment? What is the relationship of the person with dementia to the caregiver? What are the stressors in their relationships? What are the positive aspects?
    a. What does the caregiver say about the person with dementia, in the caregiver's words?
    b. What does the person with dementia say about the caregiver, in the person with dementia's words?

7. What are the problems/difficulties/concerns that the person with dementia reports?
    a. What is the person with dementia's perspective about the problems and his or her situation?
    b. How are negative emotions associated with the problems the person with dementia faces? What are the negative emotions e.g. sadness, hopelessness, helplessness, worthlessness, guilt?
    c. How can we use environmental adaptations, caregiver participation, or change in the person with dementia's perspective to solve problems and reduce the impact of negative emotions on the person with dementia?

8. What are the problems that the caregiver reports?
    a. What is the caregiver's perspective about the problems and his or her situation?
    b. How does the caregiver contribute to the person with dementia's increase in negative emotions or positive emotions?
    c. How can we involve the caregiver to help solve the person with dementia's problems and reduce the person with dementia's depression and negative emotions associated with depression?

9. Does the person with dementia enjoy his or her everyday life? What are the activities he or she used to enjoy but currently does not enjoy? How can we help the person with dementia engage in pleasurable and rewarding activities?

10. What are the obstacles or barriers to the person with dementia and/or the caregiver engaging in therapy? How can we overcome these obstacles or barriers?

[Return to Table of Contents]

MY SUMMARY OF THERAPY (version A)

Problem 1:

Feeling sad about the death of my husband, Jim. Feeling lonely and missing him, particularly in the evening when I am on my own.

Negative feelings:

Sadness, loneliness, emptiness

Strategies that I found helpful for reducing negative feelings & increasing positive ones:

1. *Distracting myself when I am feeling sad or lonely:*

When I think about Jim, I feel sad and lonely. When I feel this way, I can distract myself from these negative feelings by doing something that I enjoy. This includes:

- Watching TV
- Calling my friends
- Spending time with my friends in the evening
- Playing with the dog
- Organizing/ cleaning (a little bit at a time)
- Reading a book
- Doing word searches
- Going outside

What I need to remember:

- When doing activities, I am distracted and feel *less sad and lonely.*
- I need to make a weekly schedule to help make sure I do these activities.
- I need to ask somebody to remind me to do these activities. It’s OK to ask my daughter to remind me to do these activities. She wants to help me to feel better.

2. *Changing the way I look at the situation:*

I am now able to appreciate all the time I had with Jim and realise that not everyone has the chance to have someone like him in their lives. I value and appreciate the journey we had together.

Instead of focusing on sad memories of Jim, I can focus on positive memories of him. I can also use positive self-talk (e.g. asking myself “What would Jim say?”), which makes me feel better.

Problem 2:

Feeling that I cannot help my daughter anymore, which makes me feel helpless and worthless, and makes me worry about my daughter.

Negative feelings:

Helplessness, worthlessness, worry

Strategies that I found helpful for reducing negative feelings & increasing positive ones:

1. *Changing the way I look at the situation:*

Instead of focusing on what I cannot do, I can focus on what I still can do for my daughter.

What I need to remember:

- My daughter may reject my help due to her own personal struggle.
- I can be there emotionally for my daughter.
- In order to help my daughter, I need to take care of my own physical and mental health.
- Other people can support/help my daughter too.
- Avoid “I should" as this statement makes everyone feel worse. Asking myself "Is it helpful to think this way?" whenever I start thinking that I should be helping my daughter is helpful.

Appendix 11: An example of a personalised treatment summary for a person with more moderate dementia (version B)

MY SUMMARY OF THERAPY – PROBLEM 1 (version B)

My problems or situations that were making me feel distressed (the triggers)

Problem 1:

Missing my husband, Jim, particularly in the evening when I am on my own.

Negative feelings that were associated with these problems or situations

Sadness

Loneliness

Emptiness

Strategies that I found helpful for reducing negative feelings and increasing positive ones

1. Distracting myself from negative feelings by doing something that I enjoy such as:

   -Watching TV
   -Calling my friends
   -Playing with the dog
   -Cleaning (a little bit at a time)
   -Going outside

2. Changing the way I look at this by:

   -Focusing on positive memories of Jim.

   -Using positive self-talk (e.g. asking myself "What would Jim say?").

MY SUMMARY OF THERAPY – PROBLEM 2 (version B)

My problems or situations that were making me feel distressed (the triggers)

Problem 2:

I cannot help my daughter.

Negative feelings that were associated with these problems or situations

Helplessness

Worthlessness

Worry

Strategies that I found helpful for reducing negative feelings and increasing positive ones

1. Focusing on the things I can do for my daughter rather than the things I cannot do for her (e.g. I can be there for her emotionally).

2. Remembering that I need to take care of my own physical and mental health so that my daughter doesn’t have to.

3. Asking myself “Is it helpful to think this way?” whenever I start thinking that I should be helping my daughter.

Notes pages

[Return to Table of Contents]
