# Supplementary material for: Adapted problem adaptation therapy for depression in mild to moderate Alzheimer's disease dementia: A randomized controlled trial
Source: Alzheimers Dement. 2024 Mar 13;20(4):2990–9. doi: 10.1002/alz.13766 (PMC11032547; doi:10.1002/alz.13766)
Supplement: Supplementary file 2 — PATH Adherence Rating Scale [file ALZ-20-2990-s003.docx]

PATH Treatment Adherence Rating Scale

*Participant ID: ___________________ Session number: _______*

*Date rated: ___________________ Initials of rater: _______*

**Rater instructions:**

Please assess how well each component was addressed using the scale below:

| 1 | 2 | 3 | 4 | 5 |
| --- | --- | --- | --- | --- |
| Very poor | Poor | Satisfactory | Good | Very Good |

| **Key PATH component** | **Session(s) that applies to** | **Rating** |
| --- | --- | --- |
| Introducing PATH | Session 1 and top-up sessions 1-2 | N/A 1 2 3 4 5 |
| PATH assessment | Session 1 | N/A 1 2 3 4 5 |
| Identifying problem(s) that trigger negative emotions | Sessions 2-7 | N/A 1 2 3 4 5 |
| Identifying negative emotions associated with the problem(s) | Sessions 2-7 | N/A 1 2 3 4 5 |
| Identifying strategies for reducing negative emotions | Sessions 3-7 | N/A 1 2 3 4 5 |
| Identifying strategies for increasing positive emotions | Sessions 3-7 | N/A 1 2 3 4 5 |
| Developing a plan to put into practice | Sessions 3-7 | N/A 1 2 3 4 5 |
| Evaluating a plan that was put into practice | Sessions 3-7 | N/A 1 2 3 4 5 |
| Reviewing problems, associated negative emotions, PATH tools & PATH strategies | Session 8 and top-up sessions 1-2 | N/A 1 2 3 4 5 |
| **General therapeutic component** | **Session(s) that applies to** | **Rating** |
| Setting up the session^1^ | All | N/A 1 2 3 4 5 |
| Adopting a therapeutic stance consistent with PATH^2^ | All | N/A 1 2 3 4 5 |
| **Global rating** | **Session(s) that applies to** | **Rating** |
| Overall adherence to the PATH manual | All | N/A 1 2 3 4 5 |
| Overall PATH competence of the therapist | All | N/A 1 2 3 4 5 |
| **Comments:** | | |

^1^ Rate points 7-13 in session 1 in the PATH manual and points 7-14 in all other sessions.

^2^ Assess the degree to which the therapist adhered to the PATH therapist stance (see p15-16 in the manual) and adapted session delivery for the needs of the person living with dementia (e.g. set an appropriate pace, appropriate use of type A/B prompts and version A/B worksheets, provided summaries, sought feedback, etc).
