# Supplementary material for: Adapted problem adaptation therapy for depression in mild to moderate Alzheimer's disease dementia: A randomized controlled trial
Source: Alzheimers Dement. 2024 Mar 13;20(4):2990–9. doi: 10.1002/alz.13766 (PMC11032547; doi:10.1002/alz.13766)
Supplement: Supplementary file 3 — Trial protocol [file ALZ-20-2990-s001.pdf]

FULL PROTOCOL TITLE OF THE STUDY

Problem Adaptation Therapy for individuals with mild to moderate dementia and depression.
The PATHFINDER Trial.

SHORT STUDY TITLE / ACRONYM

Problem adaptation therapy for depression in dementia (PATHFINDER)

Chief Investigator:

Professor Robert Howard
Division of Psychiatry, University College London
6th Floor Maple House, 149 Tottenham Court Road, London W1T 7NF

Supported by:

Health Technology Assessment Programme - Grant Reference 16/155/01

Sponsored by:

Camden and Islington NHS Foundation Trust

Protocol version number and date:

Version 6.0 12MAY2020

R&D / Sponsor Reference Number(s):

Study Registration Number:

ISRCTN11185706

PROTOCOL VERSIONS

Version Stage	Versions No	Version Date	Protocol updated & finalised by;	Appendix No detail the reason(s) for the protocol update
Current	6.0	12May2020	Professor Rob Howard Chief Investigator	NSA07 change to protocol in additional response to COVID-19 ongoing situation; to allow us to conduct remote screening and consenting for the trial without the need for face to face contact with participants who are currently shielded and will likely continue to be protected from potential infection in this way for the foreseeable future.
Previous	5.0	16MARCH2020	Professor Rob Howard Chief Investigator	NSA06 change to protocol in response to COVID-19 and reducing risk from face to face contact with participants.<br><br>Where it is not PATH therapy or Research visits are not able to be delivered/ conducted face to face, sessions/visits will be carried out via telephone or videoconference systems such e.g Skype, Zoom- whichever is most practical.
Previous	4.0	27 FEB 2020	Professor Rob Howard Chief Investigator	AM05 to confirm approval for 6 month Qualitative questionnaire for caregivers and therapists formatted and submitted<br><br>To add that Research Centres can recruit through Join Dementia Research

				To set window for research outcome visits<br><br>NSA04 to add participating recruiting sites and confirm PI detail
Previous	3.0	29Oct2019	Professor Rob Howard Chief Investigator	Protocol footnote updated in appendix 3-15<br><br>Score required on Cornell for eligibility expressed more clearly.<br><br>6 month Qualitative questionnaire for caregivers and therapists formatted and submitted<br><br>Recruitment sites added
Previous	2.0	16 April 2019	Professor Rob Howard Chief Investigator	14 Appendices to protocol are on page 112<br><br>Study design refined<br><br>CRFs now complete
Previous	1.0	09 May 2018	Professor Rob Howard Chief Investigator	

National Institute for Health Research

Camden and Islington NHS Foundation Trust

DECLARATIONS

The undersigned confirm that the following protocol has been agreed and accepted and that the investigator agrees to conduct the study in compliance with the approved protocol, GCP, The UK Data Protection Act 2018 and will adhere to the Research Governance Framework 2005 (as amended thereafter), the Trust Data & Information policy, Sponsor and Priment Clinical Trials Unit SOPs and applicable Trust policies and legal frameworks.

I (investigator) agree to ensure that the confidential information contained in this document will not be used for any other purposes other than the evaluation or conduct of the clinical investigation without the prior written consent of the Sponsor.

I (investigator) also confirm that an honest accurate and transparent account of the study will be given; and that any deviations from the study as planned in this protocol will be explained and reported accordingly.

Chief Investigator:

Signature:.. Date.12..../.May...../2020......

Print Name (in full):........Professor Robert Howard...

Position:.......................Professor of Old Age Psychiatry...

On behalf of the Study Sponsor:

Signature... .... Date 24/July/2020

Print Name(in full): Emmanuel Rollings-Kamara

Position: Head of Research Operations

National Institute for Health Research

Camden and Islington NHS Foundation Trust

STUDY SUMMARY

IDENTIFIERS	
IRAS Number	238724
REC Reference No	18/WA/0209
Sponsor Reference No	Z-1080
Other research reference number(s) (if applicable)	N/A
Full (Scientific) title	Problem Adaptation Therapy for individuals with mild to moderate dementia and depression. The PATHFINDER Trial.
Health condition(s) or problem(s) studied	Alzheimer's disease and depression
Study Type	Pragmatic randomised controlled clinical trial
Target sample size	334
**STUDY TIMELINES**	
Study Duration/length	54 Months
Expected Start Date	1st July 2018
End of Study definition	31/12/2022 on completion of data collection, analysis and

and anticipated date	submission of report to Funder.
Key Study milestones	1.<u>Month 12, End of Intervention Development Phase</u><br>Outcome of Credibility/Expectancy Questionnaire<br>If credibility and expectancy summed scores are >70%,<br>if >70% participants complete 3 sessions, satisfactory ratings are achieved on the Client Satisfaction Questionnaire, and if 5 or more potentially modifiable problems per participant can be identified in these sessions, will proceed to the full RCT.
**FUNDING & Other**	
Funding	NIHR Health Technology Assessment Programme
Other support	N/A
**KEY STUDY CONTACTS**	
Chief Investigator	Professor Robert Howard
Trial Coordinator/Manager	Liz Cort

KEY ROLES AND RESPONSIBILITIES

Trial Management Group (TMG)

Dr Rebecca Gould, Division of Psychiatry, University College London. r.gould@ucl.ac.uk

Dr Philip Wilkinson, Oxfordshire and Buckinghamshire Mental Health NHS Foundation Trust. philip.wilkinson@psych.ox.ac.uk

Professor Sube Banerjee, Centre for Dementia Studies, Brighton and Sussex Medical School. s.banerjee@bsms.ac.uk

Professor Gill Livingston, Division of Psychiatry, University College London. g.livingston@ucl.ac.uk

Dr Vasiliki Orgeta, Division of Psychiatry, University College London. v.orgeta@ucl.ac.uk

Professor Alan Thomas, Institute for Ageing and Health, Newcastle University. a.j.thomas@ncl.ac.uk

Rachael Hunter, Priment Clinical Trials Unit, Primary Care and Population Health, University College London. r.hunter@ucl.ac.uk

Dr Vanessa Lawrence, Health Services Research, Institute of Psychiatry, Psychology and Neuroscience, King's College London. vanessa.c.lawrence@kcl.ac.uk

Dr Peter Bentham, Birmingham and Solihull Mental Health NHS Foundation Trust. pete.bentham@virginmedia.com

Professor Kenneth Laidlaw, Department of Clinical Psychology, University of East Anglia. k.laidlaw@uea.ac.uk

Professor Chris Fox, Department of Clinical Psychology, University of East Anglia. chris.fox@uea.ac.uk

Professor Nick Freemantle, Trialist and Senior Statistician for Priment Clinical Trial Unit, UCL Institute of Clinical Trials and Methodology, University College London. nicholas.freemantle@ucl.ac.uk

Gillian Harrison, Alzheimer's Society Research Network. ziller@btinternet.com

Tracey McDermott, Age UK Camden. tracey.mcdermott@ageukcamden.org.uk

Trial Steering Committee (TSC)

Chair: Professor Esme Moniz-Cook, Psychology Ageing & Dementia Care Research, Faculty of Health Sciences, School of Health & Social Work, University of Hull, HU6 7RX, E.D.Moniz-Cook@hull.ac.uk, Tel: 1482 464686.

Independent Clinician: Dr Seamus McNulty, Consultant Psychiatrist (Older People) and Clinical Director, NHS Ayrshire and Arran, Kilmarnock KA3 1NQ. seamus.mcnulty@aapct.scot.nhs.uk. 01563 578646.

Independent Health Economist: Dr David Meads, Academic Unit of Health Economics, Leeds Institute of Health Sciences, University of Leeds. d.meads@leeds.ac.uk. 0113 3430860.

Independent Statistician: Dr Victoria Cornelius, Senior Lecturer in Medical Statistics, School of Public Health, Imperial College London, London W12 7RH. v.cornelius@imperial.ac.uk. 020 7594 1218.

PPI Representatives: Gillian Harrison, Alzheimer's Society Research Network. ziller@btinternet.com and Tracey McDermott, Age UK Camden. tracey.mcdermott@ageukcamden.org.uk.

Independent Data Monitoring Committee (IDMC)

Chair: Professor Rowan Harwood, Professor of Geriatric Medicine, Nottingham University Hospitals NHS Trust and Nottingham University, Queen's Medical Centre, Derby Road, Nottingham NG7 2UH. rowan.harwood@nottingham.ac.uk.

Independent Statistician: Ellen Lee, Statistician and Research Associate, Clinical Trials Research Unit, ScHARR, University of Sheffield, 217 Portobello, Sheffield S1 4DP. e.lee@sheffield.ac.uk. 0114 222 0805.

Independent Clinician: Dr Bart Sheehan, Consultant Liaison Psychiatrist, Coventry and Warwickshire Partnership Trust, Caludon Centre, Clifford Bridge Road, Coventry CV2 2TE. Bartholomew.Sheehan@covwarkpt.nhs.uk Tel: 02476 967956

KEY WORDS

Depression. Alzheimer’s Disease. Problem Adaptation Therapy.

LIST OF ABBREVIATIONS

Commonly used abbreviations

AD	Alzheimer’s Disease
AE	Adverse Event
AR	Adverse Reaction
CEQ	Credibility and Expectancy Questionnaire
CI	Chief Investigator
CRF	Case Report Form
CRN	Clinical Research Network
CSDD	Cornell Scale for Depression in Dementia
CMHT	Community Mental Health Team
GCP	Good Clinical Practice
HTA	Human Technology Assessment
GAfREC	Governance Arrangement for NHS Research Ethics
IAPTs	Improving Access to Psychological Therapies
ICF	Informed Consent Form
IDMC	Independent Data Monitoring Committee
ISF	Investigator Site File
ISRCTN	International Standard Randomised Controlled Studies Number

NIA-AA	National Institute on Aging and Alzheimer's Association
PATH	Problem Adaptation Therapy
PI	Principle Investigator
PIS	Participant Information Sheet
QA	Quality Assurance
QC	Quality Control
RCT	Randomised Clinical Trial
REC	Research Ethics committee
SAR	Serious Adverse Reaction
SAE	Serious Adverse Event
SDV	Source Data Verification
SOP	Standard Operating Procedure
SSI	Site Specific Information
TAU	Treatment As Usual
TMF	Trial Master File
TMG	Trial Management Group
TSC	Trial Steering Committee
SMMSE	Standardised Mini-Mental State Examination

National Institute for Health Research

Camden and Islington NHS Foundation Trust

CONTENTS

STUDY PROTOCOL FRONT PAGE .... **Error! Bookmark not defined.**

DECLARATIONS 4

STUDY SUMMARY 5

KEY ROLES AND RESPONSIBILITIES 7

KEY WORDS 9

LIST OF ABBREVIATIONS 10

CONTENTS 12

1 INTRODUCTION 14

2 BACKGROUND 14

3 RATIONALE 17

4 OBJECTIVES 17

5 STUDY DESIGN 18

6 ELIGIBILITY CRITERIA 20

6.1 Inclusion Criteria 20

6.2 Exclusion Criteria 20

7 RECRUITMENT 20

7.1 Participant Identification 21

7.2 Consent 22

8 TRIAL PROCEDURES 23

8.1 Eligibility Screening 23

8.2 Randomisation 24

8.3 Phase 1 - Intervention Development (months 1-12): 24

8.4 Phase 2 - Full Randomised Controlled Trial (months 13 – 54) 27

8.5 End of Study 30

9 METHOD OF DATA COLLECTION and DATA ANALYSIS....30
9.1 Outcome measures....33
10 SAMPLE AND STATISTICAL METHODS....37
11 PATIENT AND PUBLIC INVOLVEMENT (PPI)....38
12 FUNDING....39
13 DATA HANDLING AND MANAGEMENT....39
14 ETHICAL AND REGULATORY CONSIDERATIONS....40
14.1 Ethics and Risk Considerations....41
14.2 Regulatory Review Requirements....42
15 ASSESSMENT AND MANAGEMENT OF RISK....42
16 RECORDING AND REPORTING OF EVENTS AND INCIDENTS....44
16.1 Definitions of Adverse Events....44
16.2 Assessments of Adverse Events....**Error! Bookmark not defined.**
16.3 Severity....**Error! Bookmark not defined.**
16.4 Causality....44
16.5 Expectedness....45
16.6 Procedures for recording and reporting Serious Adverse Events....46
16.7 Reporting Urgent Safety Measures....46
16.8 Protocol deviations and notification of protocol violations....46
17 MONITORING AND AUDITING....46
18 TRAINING....47
19 INDEMNITY ARRANGEMENTS....47
20 ARCHIVING....47
21 PUBLICATION AND DISSEMINATION POLICY....47
22 REFERENCES....49

23 APPENDICES 55

23.1 Provider codes .... Error! Bookmark not defined.

23.2 Medication unit codes .... Error! Bookmark not defined.

1 INTRODUCTION

This study has been developed in response to a Commissioned Call by the National Institute for Health Research Health Technology Assessment Programme to develop an intervention based on Problem Adaptation Therapy (PATH) (1) to treat depression in people with mild and moderate dementia within the NHS, and to test the accessibility, acceptability and credibility of this in a development phase (Phase 1) and its clinical and cost-effectiveness in a multicentre RCT (Phase 2).

2 BACKGROUND

Depression and depressive symptoms are common in people with dementia. Around 50% of patients with Alzheimer's disease have clinically significant depressive symptoms (2, 3) and 20% meet criteria for major depressive disorder (4). Depression in dementia is important because it reduces quality of life (2), exacerbates cognitive and functional impairments (36), and increases mortality (37), risk of transition to a residential care or nursing home (5) and carer burden (6).

Antidepressant medication is often prescribed to people with dementia (7) and an antidepressant withdrawal study in people with dementia reported subsequent worsening of depressive symptoms (8). However, the HTA-SADD (Sertraline or mirtazapine for depression in dementia) trial (10) and the DIADS-II trial (38) reported no superiority of antidepressants over placebo in this situation. Participants in HTA-SADD generally showed improvement in Cornell Scale for Depression in Dementia (CSDD) scores, regardless of treatment allocation. CSDD scores for those treated with mirtazapine fell by 4.9 points, with sertraline by 4.2 points and with placebo by 5.9 points, over 13 weeks. Two meta-analyses, including one recently conducted by ourselves (9, 39), have also found no significant benefits of antidepressants over placebo in people with dementia and depression. The poor response of depression in dementia to antidepressants seen in the trials does not seem to be attributable to severity of depression in participants, the type of patients recruited or to reduced adherence with trial medication and it has been suggested that the neurobiology of depression in dementia might be different from that of depression in people without dementia.

Conventional psychological therapies have also been generally disappointing in this situation. A Cochrane review of psychological therapies, including cognitive behavioural therapy, for depression

in dementia identified 6 RCTs involving a total of 439 participants and reported a small and non-significant standardised mean difference with treatment of 0.22 [95% CI 0.41 to -0.03] (11), suggesting only very modest benefit at best. Variation between the individual Cochrane reviewed studies, in terms of both the modality of psychological intervention and treatment duration, makes it very difficult to draw conclusions regarding which therapies should be recommended for depression in dementia.

Problem-solving therapy has been widely used with older people with depression and a systematic review and meta-analysis of 6 studies involving 569 participants aged over 60 reported a mean improvement in Hamilton Rating Scale for Depression scores of 6.94 points [95% CI 2.97 to 10.91, d = 1.15] (40). Problem adaption therapy (PATH) is based on problem-solving therapy and has been largely developed for use with depressed and mildly cognitively impaired older people by the Cornell group (1, 12-15). PATH is theoretically based in the process model of emotion regulation (18) and aims to reduce the negative impact of behavioural functional limitations through a problem-solving approach and use of strategies to compensate for identified deficits, pleasurable or distracting activities, breaking activities into manageable steps, caregiver participation and environmental adaptations. Specifically, PATH uses a range of emotional regulation techniques to increase positive emotions (e.g. pleasure) and decrease negative emotions (e.g. sadness, hopelessness, anxiety). These include: (1) situation selection (i.e. helping people with dementia to avoid situations that trigger negative emotions or inhibit positive emotions, and engage in situations that promote positive emotions), (2) situation modification (i.e. compensating for cognitive and physical health difficulties through the use of environmental adaptations and the support of others), (3) attentional deployment (i.e. helping people with dementia to re-direct their attention away from events that trigger negative emotions towards those that promote positive emotions), (4) cognitive change (e.g. helping people with dementia and carers to identify more hopeful thoughts), and (5) response modulation (i.e. emotional de-escalation).

In their descriptions of PATH, the Cornell group have emphasized that the intervention is focussed on the patient's "ecosystem", which they take to include the patient, their caregiver and the home environment. PATH involves the teaching and use of problem-solving skills combined with environmental adaptation tools in order to reduce functional and behavioural limitations. Recognising that patients' cognitive impairment could undermine their ability to use these problem-solving techniques and environmental manipulations unaided, caregiver participation in problem-solving, environmental manipulation and facilitation of the patient's engagement in pleasurable activities is a crucial component of PATH (41). PATH environmental adaptation tools are designed to reduce patients' functional and behavioural limitations and to take advantage of an individual's retained cognitive strengths. Tools include calendars, checklists, notebooks, alarms, signs, diaries, timed and pre-recorded messages and the step-by-step breakdown of complex tasks into more easily accomplished components (42).

The structure of PATH has been published (11) and consists of the following steps. (i) *An Initial Assessment* is conducted in the first two sessions during which the therapist gathers relevant clinical and behavioural information to create a personalised treatment plan. (ii) Using a structured interview, the therapist assesses the patient's home environment, the severity of depression and cognitive impairment, and physical and behavioural limitations, as well as areas of retained strength in cognitive function and activities of daily living. (iii) The therapist also assesses the caregiver's physical and cognitive ability, their potential availability and their willingness and motivation to become involved as a co-therapist. By the end of the second session, the patient, caregiver and therapist will have created two lists. First, a list of the patient's problems that contribute to their depression and impaired functioning, and second, a list of activities that are pleasurable for the patient and which he/she may or may not have been able to engage in during the current episode of depression. *Problem-Solving and Adaptation Treatment Implementation* occupies the next 8 sessions and involves solving the patient's problems, identifying and using appropriate PATH tools, incorporating the assistance of the caregiver, and encouraging the patient's participation in pleasurable activities. Identification and solving of each problem follows the established problem-solving therapy stages: identify the problem, define the problem, brainstorm possible solutions, evaluate potential solutions, choose the best solution, create a plan to implement the solution, implement it and evaluate how well it worked (43,44). During this phase, the therapist encourages the use of PATH tools to facilitate problem-solving as well as assessing whether the caregiver is able to help to solve a particular problem. Finally, the therapist encourages the patient's weekly engagement with pleasurable activities that are designed to improve mood. In the *Conclusion Phase*, the therapist uses the last 2 sessions to review the solutions identified and summarises the most important components of the treatment plan. Problems that have been resolved are discussed and any obstacles that remain to finding a solution are identified and discussed. The therapist gives the patient and the caregiver a personalised booklet that describes the patient's problems and the identified solutions and presents the list of PATH tools that were used during the sessions and that may be utilised in the future.

Finally, further "top up" or booster sessions may be provided to support the longer-term benefits of PATH. This is an emerging feature of the PATH intervention as implemented in the US. For example, in the current Clinicaltrials.gov description of their current trial: *Problem Adaptation Therapy for Mild Cognitive Impairment and Depression (PATH-MCI)* (NCT 03043573), the Cornell-Johns Hopkins group detail the use of 3-monthly booster sessions once the initial 12 weeks of PATH has been completed. The primary outcome measure in the PATH-MCI trial, which will complete in July 2021, is improvement in global cognitive function at 52 weeks in patients with mild cognitive impairment. A potential criticism of the published PATH studies could be that they have only focussed on outcomes immediately following the completion of treatment and have not yet demonstrated medium or longer-term benefits. The use of booster sessions represents a potential but as yet formally untested method for extending the benefits of PATH beyond the 12 weeks of treatment and so we will include booster sessions in our adapted PATH intervention.

In the study referenced in the HTA commissioned call, PATH was shown to be superior to supportive therapy in reducing depression (effect size = 0.60) and disability (effect size = 0.67) over 12 weeks in people with very mild to moderate cognitive impairment (1). This promising result now needs to be investigated to determine the potential effectiveness of PATH for people with dementia and depression within the NHS.

3 RATIONALE

There are more than 850,000 people with dementia in the UK and, in the absence of disease-modifying agents, treatment within the NHS is directed to the improvement of symptoms. Specialist memory services, generally located within secondary care mental health services, have been developed to facilitate provision of early and high quality dementia diagnosis, access to cholinesterase inhibitors and memantine, signposting to appropriate advisory and support services and to provide access to evidence-based non-drug interventions such as cognitive stimulation therapy. Memory services and older people's community mental health teams are also the specialist NHS services within which the non-cognitive symptoms of dementia are assessed and treated. These symptoms, which include agitation, aggression, psychosis and depression, cause distress for patients and their carers and are often the precipitant of institutionalisation. Although controversial and associated with increased risk of stroke and death, atypical antipsychotics are used in the treatment of psychosis and in cases of agitated and aggressive behaviour where the associated risks to the patient and others are considered to outweigh the potential risks of treatment and risperidone is licensed for the short-term (6 weeks) treatment of aggression in Alzheimer's disease. Historically, sedating drugs such as antipsychotics have been over-prescribed in people with dementia and demonstration that educational and behavioural interventions can be effective in the management of agitation and aggression has done much to reduce unnecessary drug use. But we currently have little in the way of evidence-based interventions to offer as treatment for depression in dementia and the outcome of HTA-SADD, systematic reviews and meta-analyses showing antidepressants are not superior to placebo and a Cochrane review concluding that it is hard to draw positive conclusions from trials of conventional psychological therapies, all highlight the importance of investigating the potential effectiveness of promising novel therapies such as PATH.

4 OBJECTIVES

Our principal aims are to develop an adapted PATH intervention, suitable for use with people with mild and moderate dementia for delivery within the NHS, and to design and conduct a trial to answer the question posed by the HTA's commissioning brief: *What is the clinical and cost-effectiveness of problem solving therapy for depression adapted for older adults with mild to moderate dementia?*

We aim to answer this question by developing a manualised problem-solving therapy intervention for depression specifically for people with mild and moderate dementia, examining accessibility,

acceptability, credibility and feasibility with patients, carers and health professionals, and assessing its effectiveness within an RCT.

The specific objectives are to:

(1) Adapt and manualise PATH so that it is accessible and acceptable to people with mild and moderate dementia and their carers and can be delivered by existing staff in NHS secondary care (memory services and older people's community mental health teams) and IAPTs.

(2) Obtain quantitative estimates of the accessibility, acceptability, credibility and feasibility of the PATH intervention.

(3) Use qualitative approaches to explore the intervention's acceptability to people with dementia and their caregivers, as well as therapists delivering the intervention.

(4) Establish the clinical and cost-effectiveness of adapted PATH plus usual multidisciplinary care compared to usual multidisciplinary care alone in a multi-centre, single-blind, parallel, 2-arm RCT, with an internal pilot in the first 12 months to assess feasibility of recruitment and acceptability of randomisation.

5 STUDY DESIGN

This is a multicentre, single-blind, parallel, 2-arm randomised controlled trial to assess the clinical and cost-effectiveness of adapted PATH for depression in mild and moderate dementia with a 12-month internal pilot to assess feasibility of recruitment and acceptability of randomisation.

This is a two phased trial. Phase one will conduct focus groups involving professionals with the experience of delivering interventions to people with dementia in order adapt and manualise the PATH intervention for use by the caregivers of people with mild to moderate dementia. Phase two is a full randomised control trial which includes an initial 12 month internal pilot to assess feasibility of recruitment and acceptability of randomisation.

The primary outcome will be improvement in mood at 6 months post-randomisation, with assessments at 3 months to look for immediate post-intervention mood improvement and at 12 months to examine whether potential benefits are maintained. The internal pilot will provide a "Go" signal to proceed to the full RCT if 125 participants have been randomised after 12 months' recruiting (75% of estimated number needed to hit full recruitment at 24 months, assuming recruitment numbers vs. time graph is linear), with >70% of intervention sessions attended.

If clinical and cost-effectiveness of the intervention is demonstrated we will apply for training and implementation funding so that it can be fully disseminated and rolled out within the NHS.

6 ELIGIBILITY CRITERIA

6.1 Inclusion Criteria

Patients

(1) Diagnosis of probable AD or mixed AD and vascular dementia using NIA-AA criteria (48),
(2) Mild to moderate dementia severity, defined by an SMMSE score >10,
(3) Clinically significant depression, defined by score of 8 or more on the Cornell Scale for Depression in Dementia,
(4) Aged >50 years,
(5) Sufficiently fluent in English to engage with the PATH intervention,
(6) Identified family caregiver who spends >1 hour per day on at least 3 days per week with participant and agrees to act as co-therapist for intervention. (If the participant is required to isolate or be shielded from visits; the identified caregiver should be in contact with the participant twice a week and be available to act as a co-therapist)
(7) Living in own home (i.e. not in residential care)
(8) Participant has been given feedback with regard to their diagnosis of AD or mixed AD and vascular dementia

6.2 Exclusion Criteria

Patients

(1) Diagnosis of other dementias, including dementia with Lewy bodies, Parkinson's disease dementia and frontal-temporal dementia. This is because the needs of this patient group may be different.
(2) Initiation of prescription or change in dose of antidepressant or other psychotropic medication in previous 4 weeks or plan to change treatment during the next 12 weeks,
(3) Those currently engaged in formal psychological therapy,
(4) Those requiring treatment for a severe psychiatric disorder such as schizophrenia or bipolar disorder, or
(5) Are severely depressed and expressing suicidal ideation with active plans or suicidal behaviours and intent, as other forms of treatment and support would be indicated.

7 RECRUITMENT

Recruitment of therapists from NHS staff: Healthcare professionals who will act as therapists will be identified prior to the study starting and will be consented for involvement in the pre-orientation phase. They will be psychiatrists, nurses, assistant psychologists, clinical psychologists or allied health professionals who work primarily within the Memory Service or older people's Community Mental Health Teams who agree to participate in the study within the centres that have agreed to support the trial.

Recruitment from NHS and other services: Patient participants for the development phase and the full trial will be recruited from memory services and community mental health teams for older people within secondary care mental health services, from primary care and IAPT services, and from third sector signposting services for people with dementia. Information about the study will be made available through Join Dementia Research to enable public recruitment.

Recruitment from patients' place of residence: Most people with mild and moderate dementia live in their own homes. Only participants who are living in their own homes will be recruited, rather than those living in residential care settings. This is because the involvement of an individual caregiver (rather than a member of a professional care home team) and flexibility to make environmental manipulations are important principles of PATH. If participants who are living in care homes were included in the study, then this would require training of numerous care home staff members (as the person with dementia would be cared for by more than one staff member) and environmental adaptations would need to be implemented throughout the care home environment, which may be suitable for one person but not another.

7.1 Participant Identification

Potentially eligible patient participants (see 6.1) and their caregivers will be identified and approached about involvement in the trial in one of three ways:

Firstly, clinicians will approach patients attending routine appointments at the memory service or with the Community Mental Health Team (CMHT) about the study using the patient information sheet. They will then seek verbal agreement for a member of the local research team or a research nurse from the CRN to contact the patient and caregiver to discuss the study further.

Second, clinicians from the memory service or CMHT will identify potentially eligible participants from their clinic databases and patient lists. Clinicians will then contact patients and will ask for permission to give their details to a member of the research team if they are interested in learning more about participation in the study. If they are, a study invitation letter and patient information sheet will be sent to them by a member of the research team. This will include details of how the patient and caregiver can discuss the study further with a member of the local research team or a research nurse from the local Clinical Research Network (CRN).

Third, potential participants who have previously provided consent for contact about ongoing research studies will be identified by members of the research team or the CRN (where this information is available).

7.2 Consent

It is the responsibility of the Investigator, or person delegated by the Investigator to obtain written informed consent from each participant (or assent from their personal consultee) prior to participation in the trial.

Once potential participants have been identified and agreement for contact has been obtained, a member of the research team will contact them to discuss the study further. The informed consent process will be conducted by a member of the research team, who will be GCP trained, suitably qualified and experienced and will have been delegated this duty by the CI or PI on the delegation log. Patients and their caregivers will be given adequate time to consider the study and given the opportunity to ask any questions. It will be explained to the participant that they are under no obligation to participate in the trial and that they can withdraw at any time during the trial without having to give a reason and that their treatment within the Service will not be affected if they decide not to participate. Those who are still interested in participation will be invited to attend an eligibility screening appointment (either in the patient's home or in a clinical location, depending on patient preference). Where consent is in the patients home GDPR considerations are covered in the risk assessment. Once eligibility for inclusion has been determined, informed consent will be sought from patient participants who are deemed to have capacity. For potential patient participants who are assessed as not having capacity to give informed consent for inclusion, assent will be sought from their personal (or nominated) consultee who will usually be the main caregiver who lives with them and who will be involved in the study as co-therapist. Potential patient participants with dementia will only be included in the study if they agree to this, even if they are assessed as not having the capacity to make this decision.

Informed consent will also be sought from the identified caregiver for their own inclusion and participation in the study, as co-therapist.

A copy of the signed informed consent form will be given to the participant. The original signed form will be retained in the trial file at the site and a copy in the medical/case notes/source documents.

If the PIS and consent form are amended during the course of the trial, participants will be informed of the changes and will be re-consented as appropriate.

<u>Amendment in response to on-going Covid-19 Pandemic</u>
12/May/ 2020

Identification, Consent and Recruitment

Many of those who may be suitable to take part in the Pathfinder study are currently shielded from face to face visits due to the threat of infection from the Covid-19 virus. Therefore these trial

procedures will be conducted remotely until it is possible to resume visits safely with participants and caregivers

Potential participants who have given permission for contact will be contacted by the research team by telephone. The Pathfinder study would be introduced and following discussion the patient and caregiver Information Sheet (PIS) would be sent out by post or email, with a copy of the consent forms and the consultee nomination and declaration form.

A further phone call or video-call, depending on the potential participant's preference, would then be conducted with the patient and the caregiver to discuss the information materials, consent forms and to answer any questions. During this call, the researcher would assess whether or not the potential participant has capacity to understand the information provided and to ask questions about anything they are unsure about. For those potential participants who are assessed as not having capacity to give informed consent for inclusion, assent will be sought from their personal (or nominated) consultee who will usually be the main caregiver who lives with them and who will be involved in the study as co-therapist. Potential participants will only be included in the study if they agree to inclusion.

Following the receipt of written information by the participant and their nominated consultee and following discussion with the researcher, the participant would return the completed and signed consent form to the research office. The researcher would then confirm receipt of the consent form, sign and date the form, after which the screening and eligibility process can continue.

Video-conference systems

The style of remote communication with participants will rely on the participants preference and ability to use such systems. Research teams should be guided by their NHS Trust as to the permitted systems for e.g Attend anywhere, Microsoft teams, many of which are increasingly being used for virtual clinics.

8 TRIAL PROCEDURES

8.1 Eligibility Screening

Screening of participants for both the development phase and the full trial will take place after the potential patient participant has confirmed to their clinician that they are interested in learning more about the research and has given their permission to be contacted by a member of the research team. Medical records will then be accessed by a member of the responsible clinical team or a member of the research team and reviewed to check eligibility criteria (listed as bullet points below), which should have been be collected as part of routine clinical care. In cases where further eligibility screening is required (for example, if any of the measures have not been conducted and recorded in the medical records) this will take place either in the patient's home or in a clinical location,

depending on patient preference and will be conducted by a member of the research team or CRN and participants will be asked to give consent before this is conducted. An Eligibility Checklist will be used to record this information and will be included within the case report form, anonymised with the participant's study number.

The eligibility screening will involve the accessing and collection of what would be routinely collected demographic and clinical data in a depressed patient with Alzheimer's disease managed within a Memory Service, in order to determine eligibility for the study. This will include:

- diagnosis using NIA-AA diagnostic criteria,
- Standardised Mini-Mental State Examination (SMMSE)(20),
- Assessment of depression using the Cornell Scale for Depression in Dementia (CSDD),
- limited socio-demographic data specifically age, caregiver availability,
- limited clinical data specifically medication use, engagement in current formal psychological therapy and comorbid psychiatric diagnoses.

At the first face to face meeting, for all those who meet eligibility criteria, after consent to participate has been obtained, we will collect further socio-demographic and clinical data at baseline including sex, ethnicity, marital status, years of education, highest level of educational qualification and occupational attainment, ongoing medication use (dose and frequency), time since diagnosis and time since symptom onset, and risk of self-harm (e.g. suicidal ideation).

8.2 Randomisation

Once eligibility has been checked and confirmed, patient participants in the main RCT (but not in the development stage) will be randomised on a 1:1 basis between the PATH intervention and TAU. A member of the research team who is delegated to enrolling participants, will randomise the participant using a web-based secure randomisation service provided by the company Sealed Envelope. It will be set up, tested and validated following Priment CTU SOPs. The process to randomise participants will be done in accordance with the randomisation manual for the study. Randomisation will be stratified by baseline use of antidepressant medication.

8.3 Phase 1 - Intervention Development (months 1-12):

During Phase 1 we will develop and manualise an 8-session intervention for people with dementia who are depressed (months 1-6), and will test the acceptability and credibility of the intervention (months 7-12)

Phase 1(a) - Qualitative Interviews (months 1 - 6)

In-depth individual interviews with 10-15 depressed patients with mild to moderate dementia and their carers will be conducted to examine the situations or problems that trigger negative emotions or

inhibit positive emotions; beginning with memory and functional losses, behavioural and functional limitations, interpersonal difficulties (particularly in the relationship with the caregiver), social isolation and anhedonia. Once consent has been gained, a time and location that is convenient for patients and caregivers to be interviewed together by a research worker will be determined within a fortnight. Interviews will last for a maximum of one hour but can be terminated at an earlier point if the patient or their caregiver request this. We anticipate that a single interview will be sufficient to gather the relevant information, but if it becomes clear that a further interview is necessary to do this and the patient and caregiver are prepared to meet again, a second interview will be arranged within a fortnight. Participants will be asked to reflect on issues around cognitive impairment and the effects of behaviour and functional limitations before exploring the perceived feasibility and acceptability of strategies that may reduce their negative impact. Interviews will also explore possible barriers and facilitators to engagement in PATH, how best to involve caregivers as co-therapists, how best to support them in the role, and how best to adapt the intervention to an individual's circumstances and needs. All interviews will be audio recorded and transcribed for qualitative analyses.

Sampling will be purposive and conducted on the basis of dementia diagnosis, severity of depression, physical and communication difficulties, caregiver's relationship to the person with dementia and living arrangements to explore a range of perspectives.

Phase 1(a) Focus Groups (months 1 – 6)

Four focus groups will be conducted, comprising of up to 9 individuals and including professionals (clinical psychologists, psychological well-being practitioners, assistant psychologists, and psychiatric nurses) who have experience of delivering psychological interventions to people with dementia, members of the Alzheimer's Society and other PPI partners. Discussion and activities, informed by the preceding interviews, will focus on developing accessible, acceptable and credible problem solving approaches and PATH tools. Participants will be asked to consider the feasibility of delivering the intervention both within the trial and as part of standard NHS care. Focus groups will be facilitated by either the Chief Investigator, Rebecca Gould or the Trial Manager. They will have a duration of up to 1 hour and will take place at the Division of Psychiatry, 149 Tottenham Court Road, London W1T 7NF. All sessions will be audio-recorded for qualitative and thematic analyses.

The manualised intervention will be developed from the individual interviews and workshops within the first 6 months of the project and testing of the acceptability and credibility of this intervention will take place in months 7-12.

Phase 1(b) Test of the acceptability and credibility of the Intervention (months 7- 12)

In order to assess the acceptability of the intervention to patients and their caregivers, we will take the manualised therapy intervention back to the patient-caregiver dyads who participated in Phase 1(a). We will test the acceptability of the first 2 to 3 sessions, which comprise the Initial Assessment

Phase of the PATH intervention. Within this phase, the therapist gathers relevant clinical and behavioural information in order to create a personalised treatment plan. This is based on a list of the depressed patient's problems that are considered to have contributed to their depression and impaired functioning, and a list of activities that are known to be pleasurable to the patient and which he or she may not have been able to engage in during the current episode of depressed mood.

In order to assess the acceptability and credibility of the Initial Assessment Phase of the PATH intervention, we will assess credibility of the rationale for therapy and treatment expectancy in patients and their caregivers and will use the Client Satisfaction Questionnaire as a measure of acceptability and as a tool to identify potentially modifiable problems with the intervention.

Evaluation of Phase 1(b) will be through qualitative interviews and through quantitative measures; specifically with the Credibility/Expectancy Questionnaire (22) and the Client Satisfaction Questionnaire (35). It is important to evaluate treatment credibility/expectancy when developing a new intervention as this can have a significant impact on uptake and dropout rates. In addition, we will use rates of completion of the 3 sessions as a proxy for initial engagement.

Credibility/Expectancy Questionnaire

The Credibility/Expectancy Questionnaire is a 6-item self-report measure that assesses the credibility of the rationale for therapy and expectations about treatment, which has been adapted for people with dementia, and measured immediately after delivery of the first 3 sessions. Four items are rated on a 9-point scale from 1 to 9 (lower scores are worse) and 2 items are scored on an 11-point scale from 0 to 100% (refer to appendix 13). As the measure includes items rated on two scales, the items will be standardised and summed to form separate composite scores for credibility and expectancy. We will use patient, caregiver and therapist scores for credibility of the rationale for therapy and treatment expectancy and satisfaction with the intervention after delivery of the first 2 sessions as proxies for accessibility, engagement and adherence.

If credibility and expectancy summed scores are >70%, if >70% participants complete 3 sessions, satisfactory ratings (a score of 7 or more) are achieved on the Client Satisfaction Questionnaire, and if 5 or more potentially modifiable problems per participant can be identified in these sessions, we will proceed to the full RCT.

Client Satisfaction Questionnaire

The Client Satisfaction Questionnaire will be used after delivery of the first 2 or 3 sessions as a measure of acceptability, and identification of potentially modifiable problems as a measure of feasibility of the intervention.

Intervention Development Phase Qualitative Interviews

Patients, caregivers and therapists will all be interviewed once, for no more than 1 hour, after completion of the initial 2 or 3 sessions to assess their views on acceptability, likely engagement, utility of component parts and delivery within the NHS. Therapists will be asked to identify any challenging aspects of delivering the intervention and strategies that could be used to assist them, as well as how prepared, confident and supported they feel.

We will only judge acceptability and engagement to be sufficient at the end of the Development Phase to proceed to the Full RCT Phase if credibility and expectancy summed scores are higher than 70%, satisfactory ratings are achieved on the Client Satisfaction Questionnaire, and >70% participants complete 3 sessions

8.4 Phase 2 - Full Randomised Controlled Trial (months 13 – 54)

In Phase 2 (months 13-54), we will conduct a multicentre, single-blind, parallel, 2-arm RCT to assess the clinical and cost-effectiveness of the adapted PATH intervention for depression in mild and moderate dementia with a 12-month internal pilot to assess feasibility of recruitment and acceptability of randomisation. Our outcome measures will include measures of depression, health related quality of life for people with dementia, caregiver burden, cognitive function, anxiety and cost-effectiveness as specified in the brief. Our primary outcome will be improvement in mood at 6 months post-randomisation, with assessments at 3 months to look for immediate post-intervention mood improvement and at 12 months to examine whether and how potential benefits are maintained. Our internal pilot will provide a "Go" signal to proceed to the full RCT if we have randomised 125 participants after 12 months' recruiting (75% of estimated number needed by 12 months if we are to hit full recruitment (334 participants) at 24 months, assuming recruitment numbers vs. time graph is linear), with >70% of intervention sessions attended.

We will exclude the 10-15 patient-caregiver dyads who participated in the development of the intervention from participating in the RCT in order to avoid contamination bias.

The Intervention

It is anticipated (subject to change based on the results of Phase 1) that the intervention will:

(1) Consist of up to 8 manualised, face-to-face sessions of PATH (as described above) and delivered over 12 weeks (delivered approximately weekly), each session lasting up to 1 hour, and comprising 2 assessment sessions, 5 sessions focused on problem solving using PATH tools, and 1 review session. The intervention will be delivered at home or in a clinical setting with at least 2 sessions being home based;

Where face to face visits are not practical or might expose participants to risk during the Coronavirus emergency, PATH sessions can be conducted over the telephone and/or by videoconference systems

such as Teams, Access Anywhere (whichever is most acceptable and as permitted by the individual NHS Trust) with the person with dementia and caregiver.

(2) Be supplemented by a personalised therapy booklet for patients and caregivers, developed so that it addresses each person's individual needs, and a detailed manual for therapists;

(3) Be supplemented by booster 1 hour sessions at 6 and 9 months (i.e. 3 and 6 months post-intervention), which will review key problem-solving and emotional regulation strategies used in PATH.

All therapy sessions will be recorded using encrypted digital voice recorders and 10% of these will be reviewed in order to monitor adherence to the intervention manual. The transfer of recordings of sessions is considered in the risk assessment. It may not be possible to record PATH sessions that are not conducted face to face but if possible we will record via speaker facilities which should at least pick up the therapist intervention.

An integral part of the PATH intervention developed by the Cornell group for use with depressed patients with mild to moderate cognitive impairment has been the involvement of the patient's caregiver as a co-therapist in both the situational and environmental assessments and subsequent day-to-day delivery of the active change components of PATH. The caregiver's role as a co-therapist may involve any of the following: (1) helping to identify problems that are hypothesised to maintain the person with dementia's depression, (2) helping to identify meaningful pleasurable activities that the person with dementia currently enjoys or has previously enjoyed, (3) helping to identify solutions to the problems, (4) helping the person with dementia to use PATH tools (e.g. environmental adaptations to help overcome physical and behavioural limitations) to overcome these problems and engage in pleasurable activities. The involvement of the caregiver will be adapted to each patient's level of cognitive ability such that some people with dementia will require more input from their caregiver than others. The caregivers will participate in each therapy session.

Measures of Quality of PATH Intervention delivery

Intervention Adherence Checklist: This is a checklist of PATH components, techniques, and themes discussed in each session, together with any deviations from the manual, in order to monitor adherence to the intervention manual. This will be completed by therapists delivering the intervention at the end of each session, and will also be completed for 20% of randomly-selected audiotaped sessions by an independent clinical psychologist so that treatment fidelity can be assessed, and ratings can be compared with therapists' self-reports. For actual assessments see page 29.

Phase 2 Follow Up Assessments: Follow up assessments will take place via face-to-face interview in their own home or a clinical setting at 0, 3, and 6 (primary endpoint) and 12 months post-

randomisation by a blind outcomes assessor. They will be administered to participants in each arm of the study. The transfer of data has been covered by the risk assessment. At research assessments where face to face visits are not are not practical or might expose participants to risk during the Coronavirus emergency, research visits will be conducted over the telephone and/or by videoconference systems such as Skype or Zoom (whichever is most practical).

Phase 2 Qualitative Satisfaction Questionnaire: At 6 months post-randomisation, caregivers and therapists will complete an anonymous qualitative satisfaction questionnaire to further examine the acceptability and feasibility of the intervention (or usual multidisciplinary care for those in the TAU arm). Caregivers and therapists will be asked to complete the written questionnaire (see Appendix 16) via post or online, which will comprise a combination of open and closed questions. The caregiver version will examine satisfaction with the PATH intervention and its suitability for people with dementia. Participants will be asked for their views on the strengths and limitations of PATH and methods of delivery, the perceived impact of the person with dementia's condition and circumstance, difficulties in implementing the intervention in their everyday lives and any recommendations for revising the intervention. The therapist questionnaire will explore how PATH was delivered in practice (treatment fidelity, ease of delivery in people with dementia and therapist support needs). This data will provide insight into the context in which the implementation was delivered, thus supporting the interpretation of trial findings and helping to optimise implementation to the wider NHS.

Training of Clinical Staff and Caregivers

Professional therapists will be identified prior to the study starting and will be consented in the pre-orientation phase. They will be nurses, assistant psychologists and clinical psychologists who work primarily within the Memory Service or older people's Community Mental Health Teams who agree to support and participate in the study within the centres that have agreed to support the trial. All professional therapists will attend a 1-day training workshop on the use of PATH with people with dementia and their caregivers, supplemented by copies of our newly-developed patient workbook and therapist manual. Training will be developed and delivered by members of the core research team, and will also include two members of our PPI Group. After completing training, professional therapists will deliver PATH under fortnightly supervision from existing Band 7 clinical psychologists within IAPTs, memory services and community mental health teams for older people.

Professional therapists and supervisors will also attend a 1-day top-up training course after 12 months to review and consolidate skills in delivering PATH to people with dementia, and to ensure that new staff can be trained to deliver the intervention (which will also accommodate any turnover in staff). It should be noted that 1-day training workshops will be developed in preference to longer training courses to ensure the feasibility of delivery of these training sessions. It is likely that anything

longer than one day of training would be difficult to implement, in terms of staff being released to attend it, within the NHS should the intervention be rolled out beyond the course of the study.

8.5 End of Study

End of study is defined as completion of the 10 weeks of treatment and the last follow up visit, 12 months post the end of treatment of the last participant enrolled.

Post study activity involves data analysis and submission of the final report to the Funder on 31/12/2022.

9 METHOD OF DATA COLLECTION and DATA ANALYSIS

Eligibility Screening Data

We will collect socio-demographic and clinical data at screening in order to determine eligibility for the study. This will include diagnosis using NIA-AA diagnostic criteria, SMMSE, depression using the CSDD, age, caregiver availability, medication use, engagement in current formal psychological therapy and comorbid psychiatric diagnoses. For all those who meet eligibility criteria, we will collect socio-demographic and clinical data at baseline including sex, ethnicity, marital status, years of education, highest level of educational qualification and occupational attainment, ongoing medication use (dose and frequency), time since diagnosis and time since symptom onset, and risk of self-harm (e.g. suicidal ideation). We will also collect data on our primary and secondary outcome measures as outlined in Table 1. Data collection will be conducted via face-to-face interview at 0, 3, 6 (primary endpoint) and 12 months post-randomisation by a blind outcomes assessor.

At baseline, 3, 6 and 12 months post randomisation where face to face visits are not are not practical or might expose participants to risk during the Coronavirus emergency, research visits will be conducted over the telephone and/or by videoconference systems.. It is not possible to fully administer measures such as the Standardised Mini Mental Examination (SMMSE) over the telephone and in such cases some items would need to be missed. Researchers will administer the outcome measures (with priority to the primary outcome CSSD) as completely as possible bearing in mind the wellbeing of each person and the limitations of not meeting face to face. If the participant with dementia becomes less able to use the phone or engage with the videoscreen then the therapist will complete the questionnaires with the caregiver (nominated consultee).

Table 1: Assessment intervals for measures used in the RCT.

Outcome measures, cost-effectiveness measures	0	3	6	12

and measures of bias	months	months	months	months
Primary outcome measure*				
Cornell Scale for Depression in Dementia	✓*	✓	✓*	✓
Secondary outcome measures				
DEMQOL and DEMQOL-proxy (for patients)	✓	✓	✓	✓
EQ-5D-5L (for patients)	✓	✓	✓	✓
Bristol Activities of Daily Living Scale (for patients)	✓	✓	✓	✓
Standardised Mini-Mental State Examination (for patients)	✓	✓	✓	✓
Rating Anxiety in Dementia scale (for patients)	✓	✓	✓	✓
Zarit Burden Inventory (for caregivers)	✓	✓	✓	✓
GHQ-12 (for caregivers)	✓	✓	✓	✓
Client Satisfaction Questionnaire (for patients and caregivers)[1]		✓		
Cost-effectiveness-related measures				
Client Service Receipt Inventory (for patients)	✓	✓	✓	✓
Measures of bias				

Credibility/Expectancy Questionnaire (for patients and caregivers)[2]	✓			
Treatment preference (for patients and caregivers)[2]	✓			
Checklist of PATH components, techniques, and themes	At the end of each PATH session			
Assessment of blindness				✓

[1] This will be administered after the delivery of PATH.

[2] This will be administered during the informed consent process (prior to randomisation) after participants are given a rationale for PATH.

Interview and Focus Group Data

Qualitative data from interviews and focus group discussions in Phase 1 will be transcribed verbatim and anonymised to maintain confidentiality. Data will then be analysed iteratively using a focussed thematic analysis (54). Three members of the research team will independently read through initial transcripts, separate the data into meaningful fragments and label emerging themes with codes. Coding strategies will be compared with instances of disagreement discussed until a provisional conceptual framework is developed around the situations or problems that trigger negative emotions, or inhibit positive emotions, and the acceptability, relevance, perceived value and feasibility of strategies that may reduce the negative impact of behavioural functional limitations among people with mild-moderate dementia. Data from older adults and caregivers will be triangulated to obtain the most complete understanding of the priorities, needs and concerns of these groups in relation to the proposed intervention. The analytical framework will be applied to the remaining transcripts, with themes and subthemes refined as necessary. Ideas about themes and their relationships will be recorded in theoretical memos and discussed among our Patient/Caregiver Advisory Group, Project Steering Group and PPI groups. The computer programme QSR N-VIVO will be used to process the transcripts, enabling us to code and retrieve a large volume of narrative data. Data from focus groups and follow up in-depth interviews will be analysed using a similar strategy.

Baseline and Follow-up Assessment Data

The primary analysis will be by intention to treat using a generalised mixed model, including observations for baseline and follow-up, with patient identifier as a random intercept term. Models will include an identity link function and Gaussian mixed error structures. The denominator degrees of freedom for the model will be derived from the number of patients (rather than the number of

observations which number two for each patient). Models will include baseline severity of cognitive impairment and depression, use of antidepressants, study phase (baseline or follow-up) and randomised treatment (during follow-up phase only) as patient level fixed effects. We will describe the effects in subscales of the CSSD as secondary outcomes. Secondary outcomes will be analysed using analogous models. We will include a series of a priori defined supportive analyses including addressing clustering using GEE models, more conventional models in which the baseline value is used as a patient level covariate term, and examine any relevant transformations. We will also examine patterns of missing data using multivariate joint models.

9.1 Outcome measures

The primary outcome measure will be the Cornell Scale for Depression in Dementia (CSDD) (26) score at 6 months post-randomisation. This is a 19-item scale on which each item is rated 0 (absent), 1 (mild or intermittent) or 2 (severe) and a total score of 8 or more suggests significant depressive symptoms. The CSDD differs from rating scales for assessing depressed mood in people without dementia through inclusion of a combination of observed and informant-based questions rather than simply analysing differences in the phenomenology of depression in dementia. Taking up to 30 minutes to complete (20 minutes with the caregiver and 10 minutes with the patient), the scale covers 5 main areas of symptoms and signs of depression: (1) Mood-Related Signs (anxiety, sadness, lack of reactivity to pleasant events and irritability), (2) Behavioural Disturbance (agitation, retardation, multiple physical complaints, loss of interest in usual activities in last month), (3) Physical Signs (appetite loss, weight loss, lack of energy in last month), (4) Cyclic Functions (diurnal variation in mood, difficulty falling asleep, multiple awakenings during sleep, early morning awakening), (5) Ideational Disturbance (suicidal thoughts or attempts, poor self-esteem, pessimism, mood-congruent delusions). The CSDD was the primary outcome measure in HTA-SADD and we will use the same regimen of initial and refresher training, together with regular group exercises to assess reliability of research workers that we developed for HTA-SADD.

Secondary outcome measures

Secondary outcome measures will be as follows:

(1) CSDD score at 3 and 12 months post-randomisation.

(2) Disease-specific health-related quality of life (HRQL) measured with the DEMQOL and DEMQOL-proxy (27) and generic quality of life with the EQ-5D (28). DEMQOL (28 items) and DEMQOL-Proxy (31 items) are interviewer-administered measures which obtain self and informant reports of the HRQL of people with dementia; items cover the feelings, memory, and everyday life of the person with dementia in the last week and Likert-scale responses (a lot/quite a bit/a little/not at all) are used with higher overall total scores reflecting better HRQL. The EQ-5D-5L is a 5-item self-report measure of health-related quality of life used to calculate utility

scores for use in economic evaluations. Each of the 5 items is rated on a 5-point scale from no problem to extreme problems.

(3) Functional abilities with the Bristol Activities of Daily Living Scale (29), a 20-item scale completed with the caregiver and covering everyday daily living activities (food preparation, eating, drink preparation, drinking, dressing, personal hygiene, cleaning teeth, bathing/showering, toileting, transfers, mobility, orientation to time, orientation to place, communication, use of telephone, housework/gardening, shopping, finances, games/hobbies and transport) that has excellent psychometric properties across the mild to moderate dementia severity range.

(4) General cognitive function with the SMMSE (20), a more objective and valid version of the traditional MMSE, that takes between 5 and 10 minutes to administer to a patient.

(5) Anxiety, measured with the Rating Anxiety in Dementia scale (30), a 20-item scale assessing worry about physical health, cognitive performance, family problems, false beliefs, items considered trivial by others, level of frightfulness, noise sensitivity, sleep disturbance, irritability, trembling, motor tension, restlessness, fatigueability, palpitations, autonomic symptoms, hyperventilation, dizziness, sweating, phobia and panic attacks. Individual items are scored from 0 (absent) to 3 (severe) and a total score of 11 or more indicates clinically significant anxiety. It takes 20 minutes to complete; 10 minutes with the caregiver and 10 minutes with the patient.

(6) Resource use collected using the Client Service Receipt Inventory (CSRI) for Health and Social Care Resource Use (31). This is a measure of service utilisation used to calculate patient and caregiver costs, which we will modify for the patient group with mild to moderate dementia, and will use to record other forms of psychological therapy and pharmacotherapy received outside of the study.

(7) Measures specifically collected from caregivers about their own burden and wellbeing will include the Zarit Burden Inventory (32), a well-validated 22-item self-report measure of caregiver burden, and caregiver mental health assessed with the GHQ-12 (33), a short version of the General Health Questionnaire that is sensitive in the detection of minor psychiatric disorder through assessment of a respondent's current state and how this differs from their usual state.

(8) Satisfaction with the PATH intervention in people with dementia and their caregivers, measured with the Client Satisfaction Questionnaire (35). This was used to assess satisfaction with PATH in the original study by the Cornell group, and so has been validated in this population.

Cost effectiveness Analysis

The primary analysis for the economic evaluation will be the incremental cost per QALY of PATH intervention compared to TAU over the duration of the trial conducted from the NHS and social services cost perspective, with a secondary analysis from the societal perspective that will include the cost impact on informal caregivers.

Outcome assessment intervals

Assessments will be carried out at baseline, 3 months (i.e. in most cases this will be following completion of intervention delivery), 6 months and 12 months post-randomisation. The outcome assessment visit should be made as close to the calculated date as possible – within 2 weeks before or after the due date.

Satisfaction with the PATH intervention using the Client Satisfaction Questionnaire will only be completed at 3 months post-randomisation (i.e. immediately following completion of intervention delivery). Our choice of *depression rating at 6 months as the primary outcome* is different from many earlier trials of psychological interventions that have often placed the primary outcome immediately following completion of intervention delivery. Our approach was initially recommended by our PPI member who was particularly concerned about the magnitude of benefit that caregivers would see in return for the significant investment that will be asked of them in their participation as co-therapists. She didn't consider that anything less than an improvement that was sustained for 6 months would justify this investment and believed that this should be reflected in the trial's design. If a PATH-based intervention is successful in teaching patients and caregivers to use problem-solving skills and make changes to their environment that reduce functional and behavioural limitations, this should translate into enduring improvements in mood. A positive trial result at 6 months would have clear implications for the management of depression in people with dementia in the NHS.

Measures of bias and methods of controlling for potential sources of bias

Expectations about treatment, patients' preferences for treatment, adherence to the intervention by therapists, use of other forms of treatment during the study and blindness of outcome assessors are all potential sources of bias that can affect treatment outcomes. Consequently, we will include the following measures, which will also allow us to assess further the acceptability and feasibility of the intervention:

(1) Expectations about treatment: As noted above, it is important to evaluate treatment credibility/expectancy when developing a new intervention as this can have a significant impact on uptake and dropout rates. This will be measured during the informed consent process (prior to randomisation), after participants are given a rationale for PATH, using the Credibility/Expectancy Questionnaire.

(2) Treatment preference: We will include a measure of patients' and caregivers' preferences for treatment (collected on a four point Likert scale from 0 to 3). This will also be administered during the informed consent process (prior to randomisation) after participants are given a rationale for PATH.

(3) **Intervention Adherence Checklist:** All selection of therapy sessions will be recorded using encrypted digital voice recorders in order to monitor adherence to the intervention manual. We will develop a checklist of PATH components, techniques, and themes discussed in each session during the development phase, together with any deviations from the manual, in order to monitor adherence to the intervention manual during the intervention phase. The developed adherence checklist will be used during the full RCT and completed by therapists delivering the intervention at the end of each session, and will also be completed for 20% of randomly-selected audiotaped sessions by an independent clinical psychologist so that treatment fidelity can be assessed, and ratings can be compared with therapists' self-reports. The random selection of sessions will be stratified according to therapist, phase of the intervention (early, middle or late), and phase of study recruitment (early, middle or late), as previously recommended (53).

(4) Assessment of blindness: Although outcome assessors will be blinded to treatment allocation at follow-up and at each of the visits participants will be asked beforehand not to reveal their allocation to outcome assessors. Some may accidentally reveal this and some outcome assessors may be able to guess this. Consequently, we will ask all outcome assessors to guess whether they think the participant was allocated to the intervention or control arm.

In addition, we will control for potential sources of bias in a number of ways:

(1) Contamination from booster sessions: We will conduct booster sessions at 6 months and *after* the outcome assessment has been completed in order to avoid biasing outcomes at this time point.

(2) Contamination from those involved in developing the intervention: We will exclude the 15-20 patient-caregiver dyads who participated in the development of the intervention from participating in the RCT in order to avoid contamination bias. They will, however, be given access to the intervention materials.

(3) Contamination in the control group: A final potential source of bias is contamination in the TAU/control group; that is, use of therapies (pharmacological or behavioural), including elements of the study intervention, which may in turn attenuate the apparent effect of PATH in the trial. We will use the Client Service Receipt Inventory, a measure of service utilisation, to record other forms of psychological therapy and pharmacotherapy for depression received outside of the study. We will undertake additional exploratory data analysis to assess the impact of these

therapies on the effectiveness of PATH. Because of specific concerns about contamination of the TAU arm by elements of the PATH intervention we asked our statistician whether a cluster RCT design would be appropriate. His view was that potential contamination would have to be substantial to overwhelm the relative efficiencies of individual randomisation and that we should minimise contamination by other means. We will, for example, ensure that the trained PATH therapists cannot be the team keyworker for the participants within their clinical services who have been allocated to receive TAU.

10 SAMPLE AND STATISTICAL METHODS

The sample sizes for each of the activities in the development phase are given in the text describing each activity in 8.3.

Our sample size calculation for the full RCT indicates that 334 participants would allow detection of a 0.4 SD effect size (corresponding to a 2.0 point difference on the CSDD which is considered to be a minimum clinically important difference) with a 2-sided alpha of 5% and 90% power and assuming a 20% loss to follow-up at 6 months. A 2-points difference on the CSDD was used as the minimum clinically important difference for the HTA-SADD trial (10). This is more conservative than the 3-points difference included in the published sample size calculation for the RAPID trial of cognitive bias modification in the treatment of depression in people with dementia (51), but we have adopted it to be consistent with a previous HTA study and to avoid being underpowered to detect a potentially significant difference. In HTA-SADD, standard deviations of the change in CSDD score at 13 weeks in the drug (sertraline and mirtazapine) and placebo groups ranged from 4.7 to 5.1 points and at 39 weeks from 5.2 to 6.0 points and we have estimated a SD of 5.0 points at 24 weeks for PATHFINDER. Our loss to follow-up rates are based on those reported in the psychological intervention arm of the DAISY trial, which was a large RCT of psychosocial counselling vs. psychosocial support for depression in people with mild AD (34). They reported rates of 12% at 6 months follow-up and 20% at 12 months. As the mean baseline MMSE score was 24.1 in this study, we anticipate that our loss to follow-up rates may be higher due to the inclusion of people with more severe dementia. Therefore, we have used 20% loss to follow-up at 6 months to be conservative in our sample size calculation.

We will conduct a range of sensitivity analyses based on any assumptions made in the analysis in particular the grade of staff delivering PATH and duration of the intervention.

Baseline and Follow-up Assessment Data

The primary analysis will be by intention to treat using a generalised mixed model, including observations for baseline and follow-up, with patient identifier as a random intercept term. Models will include an identity link function and Gaussian mixed error structures. The denominator degrees of freedom for the model will be derived from the number of patients (rather than the number of observations which number two for each patient). Models will include baseline severity of cognitive

impairment and depression, use of antidepressants, study phase (baseline or follow-up) and randomised treatment (during follow-up phase only) as patient level fixed effects. We will describe the effects in subscales of the CSSD as secondary outcomes. Secondary outcomes will be analysed using analogous models. We will include a series of a priori defined supportive analyses including addressing clustering using GEE models, more conventional models in which the baseline value is used as a patient level covariate term, and examine any relevant transformations. We will also examine patterns of missing data using multivariate joint models.

Cost effectiveness Analysis

We will calculate the incremental cost per quality adjusted life year (QALY) gained with the PATH intervention compared to TAU over the 12-month duration of the trial. In the primary analysis costs will be from an NHS, social services cost perspective, and QALYs will be calculated based on patient and caregiver responses to the EQ-5D 5 level (EQ-5D-5L) and relevant UK tariff (54). Supporting analyses will include (i) costs from a societal perspective including cost impact on informal caregivers; (ii) QALYs calculated from responses to the DEMQOL. The cost of the intervention will include the cost of training, supervision and staff time to deliver PATH costed at the relevant staff grade. In both arms, we will collect health and social care resource use using an adapted version of the CSRI. The questionnaire will focus on other treatments for depression that patients' access as well as other depression related health and social care service use. We will also collect information about caring activities of paid and unpaid caregivers for the patient. Resource use will be costed using the most recent nationally published sources (59 & 60). Informal caregivers will be costed at the rate of paid caregivers based on the assumption that in the absence of an informal caregiver a paid carer would be required to undertake the same role. QALYs will be calculated as the area under the curve adjusting for baseline differences.

We will report means and 95% confidence intervals for all key results. These will be calculated using bootstrapping adjusting for baseline and including the same covariates as specified in the statistical analysis plan. For variables where there is greater than 85% missing data, missing values will be imputed using multiple imputation. Cost-effectiveness planes and cost-effectiveness acceptability curves for a range of values of willingness to pay for a QALY gained will be reported using the bootstrapped results.

11 PATIENT AND PUBLIC INVOLVEMENT (PPI)

Tracey McDermott, Dementia Wellbeing Lead for Age UK Camden, and Gillian Harrison, Alzheimer's Society Research Network Volunteer, informed the design of the study at the funder's Expression of Interest stage through their contribution to a PPI workshop. For the study conduct and dissemination phases, Tracey and Gillian agreed to join the research team as co-applicants and the Alzheimer's Society have agreed to add two further Research Network Volunteers with specific experience of

caring for a family member with dementia who had additional difficulties with low mood, who will collectively constitute our Patient/Carer Advisory Group. PPI co-applicants Tracey and Gillian will be members of the Trial Management Group, will contribute expertise and input to the initial intervention development and will continue to advise on recruitment, liaison with local Alzheimer's Society branches and membership at recruitment centres and will advise on the content of trial materials as well as ongoing trial conduct through to the analysis and interpretation of the findings. The two further Research Network Volunteers will provide a more independent PPI voice during the conduct of the trial through membership of the Trial Steering Committee. Our experience from previous trials is that it is important to have at least two PPI members of the Trial Steering Committee so that they can support each other, between and during meetings. Once data collection and analysis is completed, our PPI co-applicants and collaborators will work together with us to support dissemination of the results and potential implementation of the intervention within the NHS should it prove to be effective in the trial. The Alzheimer's Society is an ideal partner to co-produce and drive a roll-out and implementation plan.

12 FUNDING

The research costs for the study have been supported by NIHR's Health Technology Assessment Programme (Grant reference HTA 16/155/01).

13 DATA HANDLING AND MANAGEMENT

All data will be collected and handled in accordance with Priment SOP Data Handling and the trial specific arrangements will be detailed in the data management plan. This will ensure that all data will be handled in accordance with the UK Data Protection Act (2018), the advised applicable requirements of GDPR, and any subsequent updated Data Protection Act/Data Protection Bill(s). All research data will be fully anonymised using unique identification numbers and stored without contact details (names or addresses). Associations between participants' contact details and identification numbers will be stored in a separate encrypted electronic password-protected database. Access to this document will be restricted to the Chief Investigator and the trial manager. All data will be held on a secure database on an encrypted, password-protected computer, and access to it will be restricted to the research team. All research data will be retained for up to 25 years; data generated from the phase 1 Intervention Development will be retained a minimum of 10 years. Data generated from the RCT will be retained for up to 25 years.

A data management plan will be created which will include details of the data collection tools, methods of completing case report forms, sign off of completed CRFs, source document identification and methods to maximise completeness of data collection.

It will be the responsibility of the investigator to ensure the accuracy of all data entered in the CRFs. The delegation log will identify all those personnel with responsibilities for data collection and handling, including those who have access to the trial database.

Data collected for the study will be entered in the medical/case notes/source documents in the first instance and then transcribed into the CRFs. The CRFs will be entered into a web-based clinical data management system, Red Pill, provided by Sealed Envelope through Priment. Sealed Envelope has been assessed by Priment to ensure that adequate processes are in place and are being followed for quality management, software development and security. There will be an agreement in place with Sealed Envelope to ensure compliance and agreement with clinical trial regulations and data protection laws.

Audio files of qualitative interviews and therapy sessions recorded on encrypted digital voice recorders will be uploaded to a secure server using a system called Data Safe Haven, which satisfies the highest level security requirements of NHS trusts. Audio files of the interviews and therapy sessions will be retained until they have been transcribed to written form. Transcriptions of workshops and qualitative interviews will be completed as soon as possible after collection and anonymised. Audio files of qualitative interviews and therapy sessions will be deleted from encrypted digital voice recorders as soon as confirmation is received from the research team that the files have been correctly uploaded to Data Safe Haven. Digital voice recorders will be securely stored in a locked desk or cabinet when not in use.

Hardcopies of study consent forms held by the central research team will be kept in a locked cabinet in the Department of Psychiatry at University College London (UCL) and retained for 2 years post research data analysis, versions retained by host research sites will be retained in a secure facility with access limited to the site principal investigator and designated members of the research team. Please refer to risk assessment for full details. Consent forms may be converted to electronic versions and if so these will be stored on an encrypted server, on password protected computers, by the hosting research sites and at the C.I. and central research team's administrative base at UCL. Access to data will be restricted to the research team.

Personal data will be kept for no longer than contact with research participants is required, no longer than 12 months from when participation in the study ends, or until the research results have been written up so that it can be sent to the participants that have requested this.

14 ETHICAL AND REGULATORY CONSIDERATIONS

The study was externally expert peer reviewed as part of the HTA's competitive funding application process.

14.1 Ethics and Risk Considerations

Physical and/or communication difficulty. As with cognitive impairment, we will investigate the effects of adapting the approach to PATH and the involvement of the caregiver during the development phase so that PATH can be accessible to people with dementia who have physical and/or communication difficulties, in addition to cognitive difficulties. The theoretical basis for such adaptations will be taken from the principles of person-centred care (23), cognitive rehabilitation (24) and Selective Optimisation and Compensation (25), which will supplement PATH tools (e.g. environmental adaptation tools). Principles of person-centred care and 'positive person work' include recognising the person with dementia as a person (rather than just seeing the dementia), enabling the person with dementia to collaboratively and actively engage in tasks (rather than being a passive recipient or disabling them), and validating the person with dementia's experiences (rather than ignoring or invalidating them). Cognitive rehabilitation employs internal strategies aimed at enhancing learning and memory and external strategies aimed at compensating for cognitive deficits to help people achieve an optimal level of physical, psychological and social functioning given their cognitive impairment. Internal strategies include errorless learning and the use of cues to learn and remember information, and have been successfully demonstrated in people with mild to moderate AD (47). External strategies include the use of memory and communication aids, environmental adaptations (such as signs, reducing clutter, educating others, etc.), and assistive technology. Selective Optimisation with Compensation was originally developed to aid adaptation to the challenges of ageing. It involves helping people to choose the best functional domains to focus their resources on, engage in tasks that they perform best, and find ways of compensating for losses. All adaptations will be carefully implemented according to each person's individual needs so as to promote optimal problem resolution and engagement in pleasurable activities, without causing excess disability (e.g. by over-compensating for cognitive, physical and/or communication difficulties). In addition to adapting PATH using these principles, standard therapeutic strategies will be used to compensate for cognitive difficulties such as providing a personalised booklet in which visual clutter is minimised, using language tailored to the level of cognitive difficulties, providing session summaries as a reminder of the content of the sessions, clarifying and repeating key concepts and skills within and between sessions (e.g. recapping on the previous session at the start of the next session), and working at a slower pace.

Ongoing prescription medication. Although antidepressants have not shown significant superiority to placebo in the treatment of depression in people with dementia, apparent high rates of placebo response seen when they are used, as in the HTA-SADD trial, could potentially dilute the treatment effect of the PATH intervention in the trial. We will consequently not include participants who have started an antidepressant within the previous 4 weeks as a precaution against these potential placebo effects. Long term antidepressant prescription is common in people with dementia and excluding all patients on these drugs would mean that our participant group was not representative of people with depression in dementia within NHS practice as well as representing a potential barrier to recruitment.

We will therefore include participants who have been on a stable dose of antidepressant for longer than 4 weeks but remain sufficiently severely depressed to meet study inclusion criteria. We will monitor use of all psychotropic treatment during the course of therapy using the Client Service Receipt Inventory, and undertake exploratory analyses to assess the impact of this on the difference between the randomised groups.

Mobility of patients. Although the aim is to develop and evaluate a PATH-based intervention that can be used as widely as possible in people with mild to moderate dementia with depression in the NHS, there are some practical considerations involving participant mobility that could limit potential effectiveness of the PATH intervention. For example, people with dementia who are mostly confined to bed or have only very limited mobility within the home might find it more difficult to significantly improve their level of engagement with pleasurable activities. We will work to overcome these difficulties and find solutions in the therapy. We will offer the option of having PATH sessions delivered at home after completion of the first two sessions if the participant and caregiver request this, but we believe that with transport and support the large majority of participants will be able to come to the memory service or CMHT team base. If the PATH intervention is to be implemented into NHS practice it will have to be feasibly delivered within such NHS environments and we consider that the proven model from CST shows that this can work well for patients and their caregivers. We will collect information as to what proportion of this population need this accommodation.

14.2 Regulatory Review Requirements

This research is subject to review by the Health Research Authority for HRA Approval. HRA review is required for all research involving NHS organisations in England where the NHS organisation has a duty of care to participants, either as patients/service users or NHS staff/volunteers. REC review and approval is applicable to all research taking place within the UK that involves an individual that an NHS organisation has a duty of care towards, in the capacity of service provider.

This research will not commence until both REC Favourable Opinion and HRA Approval is received, and then not until each organisation where the research is to take place has confirmed that they can host the research.

15 ASSESSMENT AND MANAGEMENT OF RISK

Participants will remain under the care of their GP and care team for the duration of their involvement in the study. Risk of harm to self or others will be monitored throughout the study. If suicidal ideation without intent is expressed at any point then the participant's GP and care team will be contacted and the participant will be monitored according to local clinical protocol. Decisions as to whether the participant will be withdrawn from the study will depend on the outcome of this assessment and in full discussion with the participant, their caregiver and their care team. Evidence of any adverse

effects from the PATH intervention will be monitored throughout the study. Reasons for withdrawing participants from the intervention will be clarified with the Trial Management Group prior to the commencement of the feasibility study. Anyone experiencing an increase in distress will be assessed for risk, and standard operating procedures will ensure safety is respected. New reports of suicidal behaviour during the intervention will be reported as Serious Adverse Events.

16 RECORDING AND REPORTING OF EVENTS AND INCIDENTS

16.1 Definitions of Adverse Events

Term	Definition
Adverse Event (AE)	Any untoward medical occurrence in a patient or study participant, which does not necessarily have a causal relationship with the procedure involved.
Serious Adverse Event (SAE).	Any adverse event that:<br>• results in death,<br>• is life-threatening*,<br>• requires hospitalisation or prolongation of existing hospitalisation**,<br>• results in persistent or significant disability or incapacity, or<br>• consists of a congenital anomaly or birth defect
*A life- threatening event, this refers to an event in which the participant was at risk of death at the time of the event; it does not refer to an event which hypothetically might have caused death if it were more severe.<br>** Hospitalisation is defined as an in-patient admission, regardless of length of stay. Hospitalisation for pre-existing conditions, including elective procedures do not constitute an SAE.	

16.2 Causality

The assessment of relationship of serious adverse events to the procedure is a clinical decision based on all available information at the time of the completion of the case report form.

Causality assessments will be captured in the study specific SAE form.

The following categories will be used to define the causality of the serious adverse event:

Category	Definition
Definitely:	There is clear evidence to suggest a causal relationship, and other possible contributing factors can be ruled out.
Probably:	There is evidence to suggest a causal relationship, and the influence of other factors is unlikely
Possibly	There is some evidence to suggest a causal relationship (e.g. the event occurred within a reasonable time after administration of the study procedure). However, the influence of other factors may have contributed

	to the event (e.g. the participant's clinical condition, other concomitant events).
Unlikely	There is little evidence to suggest there is a causal relationship (e.g. the event did not occur within a reasonable time after administration of the study procedure). There is another reasonable explanation for the event (e.g. the participant's clinical condition).
Not related	There is no evidence of any causal relationship.
Not Assessable	Unable to assess on information available.

16.3 Expectedness

Category	Definition
Expected	An adverse event which is consistent with the information available about the modified PATH intervention.
Unexpected	An adverse event which is not consistent with the information available about the study intervention.

16.4 Procedures for recording and reporting Serious Adverse Events

All SAEs will be recorded on a serious adverse event (SAE) form. The Principal Investigator or designated individual will complete an SAE form documenting events that occur at their site and the form will be preferably emailed to the Sponsor within 5 working days of becoming aware of the event. The Chief or Principal Investigator will respond to any SAE queries raised by the sponsor as soon as possible.

Where the event is unexpected and thought to be related to the procedure this must be reported by the Investigator to the Health Research Authority within 15 days.

16.5 Reporting Urgent Safety Measures

If any urgent safety measures are taken the Chief Investigator will immediately and in any event no later than 3 days from the date the measures are taken, give written notice to the relevant REC and Sponsor of the measures taken and the circumstances giving rise to those measures.

16.6 Protocol deviations and notification of protocol violations

A deviation is usually an unintended departure from the expected conduct of the study protocol/SOPs, which does not need to be reported to the sponsor. The CI will monitor protocol deviations.

A protocol violation is a breach which is likely to effect to a significant degree –

(a) the safety or physical or mental integrity of the participants of the study; or

(b) the scientific value of the study.

The CI and sponsor will be notified immediately of any case where the above definition applies during the study conduct phase.

17 MONITORING AND AUDITING

The Chief Investigator will oversee the research to ensure activities conducted by the study team adhere to the protocol, and good clinical practice. The Chief Investigator will inform the sponsor should he/she have concerns which have arisen from monitoring activities, and/or if there are problems with oversight/monitoring procedures.

The trial will be subject to monitoring, auditing and inspection by the sponsor or the sponsor's delegated representatives, and the relevant authorities responsible for each of the sites where the research will take place. The purpose of the monitoring is to ensure that the study is conducted in accordance with the authorised study protocol, the principles of GCP and all applicable regulations.

The sponsor/sponsor's delegated representatives and regulators will require access to the study site for these inspections which will be supervised by the site investigator(s). The inspection activities apply to the following, and associated, areas in order to:

- review the trial master/site file (hard and soft copies);
- review the study participant consent forms;
- review research documents as approved by applicable regulatory body and those referenced in protocol;
- review templates for participant facing material, current and superseded versions;
- view the storage facilities/spaces for research documentation (paper and electronic);
- review site delegation of duties log;
- review trial amendment log.

18 TRAINING

The Chief Investigator will review and provide assurances of the training and experience of all staff working on this study. Appropriate training records will be maintained in the study files.

19 INDEMNITY ARRANGEMENTS

Camden and Islington NHS Foundation Trust will provide NHS indemnity cover for negligent harm, as appropriate and is not in the position to indemnify for non-negligent harm. NHS indemnity arrangements do not extend to non-negligent harm and NHS bodies cannot purchase commercial insurance for this purpose; it cannot give advance undertaking to pay compensation when there is no negligence attributable to their vicarious liability. The Trust will only extend NHS indemnity cover for negligent harm to its employees, both substantive and honorary, conducting research studies that have been approved by the R&D Department. The Trust cannot accept liability for any activity that has not been properly registered and Trust approved.

20 ARCHIVING

Each participating site that will collect research data recognise that there is an obligation to archive study-related documents at the end of the study (as such end is defined within this protocol), and the principal investigator at each site will take responsibility for ensuring the site specific research data is accordingly archived. The study master file will be archived at UCL in accordance with the UCLH Standard Operating Procedure 10 Archiving of Investigator Site File (ISF). It will be archived for a minimum of 10 years from the study end, and no longer than 30 years from study end.

21 PUBLICATION AND DISSEMINATION POLICY

We will disseminate our findings to the academic and clinical community, service users and their families and the wider public through:

(1) Peer-reviewed, high impact international open-access journals (e.g. New England Journal of Medicine and The Lancet). We will publish our protocol and report our findings in accordance with reporting guidelines for quantitative cohort studies (56) and qualitative research (57), as well as guidelines relevant to non-pharmacological treatment intervention such as the CONSORT guidelines (58).

(2) National and international academic conferences, including specialist AD and dementia meetings as well as geriatric medicine, nursing and social care conferences.

(3) Talks to local and national Alzheimer's Society meetings and other organisations following guidance from our Patient/Carer Advisory Group and including an interested member of this group.

(4) University and NIHR media releases, Twitter feeds and University websites.

(5) Training and seminars delivered via the NHS Memory Service Networks and professional bodies such as the Old Age Faculty of the Royal College of Psychiatrists and British Psychological Society and associated conferences and meetings. In particular, we will work with the Memory Services National Accreditation Programme (MSNAP), who work to assure and improve the quality of memory services and who have been effective in promoting adoption of evidence-based interventions within dementia services.

22 REFERENCES

1. Kiosses DM, Ravdin LD, Gross JJ, Raue P, Kotbi N, Alexopoulos GS (2015) Problem Adaptation Therapy (PATH) for older adults with major depression and cognitive impairment: A randomised clinical trial. JAMA Psychiatry 72: 22-30.

2. Lyketsos CG, Steele C, Baker L, Galik E, Kopunek S, Steinberg M, Warren A (1997) Major and minor depression in Alzheimer's disease: prevalence and impact. Journal of Neuropsychiatry and Clinical Neuroscience 9: 551-61.

3. Starkstein SE, Jorge R, Mizrahi R, Robinson RG (2005) The construct of minor and major depression in Alzheimer's disease. American Journal of Psychiatry 162: 2086-93.

4. Enache D, Winblad B, Aarsland D (2011) Depression in dementia: epidemiology, mechanisms and treatment. Current Opinion in Psychiatry 24: 461-72.

5. Stern Y, Tang MX, Albert MS, Brandt J, Jacobs DM, Bell K (1997) Predicting time to nursing home care and death in individuals with Alzheimer's disease. JAMA 277: 806-12.

6. Gonzalez-Salvador MT, Arango C, Lyketsos CG, Barba AC (1999) The stress and psychological morbidity of the Alzheimer patient caregiver. International Journal of Geriatric Psychiatry 14 701-10.

7. Kessing LV, Harhoff M, Andersen PK (2007) Treatment with antidepressants in patients with dementia – a nationwide register-based study. International Psychogeriatrics 19: 902-13.

8. Bergh S, Selbaek G, Engedal K (2012) Discontinuation of antidepressants in people with dementia and neuropsychiatric symptoms (DESP study): double blind, randomised, parallel group placebo controlled trial. BMJ 344: e1566.

9. Thompson S, Herrmann N, Rapoport MJ, Lanctot KL (2007) Efficacy and safety of antidepressants for treatment of Alzheimer's disease: a meta-analysis. Canadian Journal of Psychiatry 52: 248-55.

10. Banerjee S, Hellier J, Dewey M, Romeo R, Ballard C, Baldwin R, Bentham P, Fox C, Holmes C, Katona C, Knapp M, Lawton C, Lindesay J, Livingston G, McCrae N, Moniz-Cook E, Murray J, Nurock S, Orrell M, O'Brien J, Poppe M, Thomas A, Walwyn R, Wilson K, Burns A (2011) Sertraline or mirtazapine for depression in dementia (HTA-SADD): a randomised, multicentre, double-blind, placebo-controlled trial. Lancet 378: 403-11.

11. Orgeta V, Qazi A, Spector A and Orrell M (2015) Psychological treatments for depression and anxiety in dementia and mild cognitive impairment: systematic review and meta-analysis. British Journal of Psychiatry 207: 293-8.

12. Kiosses DM, Teri L, Velligan DI, Alexopoulos GS (2011) A home-delivered intervention for depressed, cognitively impaired, disabled elders. International Journal of Geriatric Psychiatry 26: 256-62.

13. Kiosses DN, Arean PA, Teri L, Alexopoulos GS (2010) Home-delivered Problem Adaptation Therapy (PATH) for depressed, cognitively impaired, disabled elders: A preliminary study. American Journal of Geriatric Psychiatry 18: 988-98.

14. Arean PA, Raue P, Mackin RS, Kanellopoulos D, McCulloch C, Alexopoulos GS (2010) Problem-solving therapy and supportive therapy in older adults with major depression and executive dysfunction. American Journal of Psychiatry 167: 1391-8.

15. Alexopoulos GS, Raue P, Kiosses DN, Mackin RS, Kanellopoulos D, McCulloch C, Arean PA (2011) Problem-solving therapy and supportive therapy in older adults with major depression and executive dysfunction: effect on disability. Archives of General Psychiatry 68: 33-41.

16. Alexopoulos GS, Kiosses DN, Heo M, Murphy CF, Shanmugham B, Gunning-Dixon F (2005) Executive dysfunction and the course of geriatric depression. Biological Psychiatry 58: 204-10.

17. Potter GG, Kittinger JD, Wagner HR, Steffens DC, Krishnan KR (2004) Prefrontal neuropsychological predictors of treatment remission in late-life depression. Neuropsychopharmacology 29: 2266-71.

18. Gross JJ (2014) Emotion Regulation: Conceptual and Empirical Foundations. In: Gross JJ, Editor. Handbook of Emotion Regulation. New York, NY: Guilford pp 3-20.

19. Jurica PJ, Leitten CL, Mattis S (2001) DRS-2. Dementia rating Scale – 2. Professional Manual. Lutz, Fl: Psychological Assessment Resources Inc.

20. Molloy, D.W., Alemayehu, E., & Roberts, R. 1991. Reliability of a Standardized Mini-Mental State Examination compared with the traditional Mini-Mental State Examination. American Journal of Psychiatry, 148(1), pp.102–105.

21. Kiosses DN, Rosenberg PB, McGovern A, Fonzetti P, Zaydens H, Alexopoulos GS (2015) Depression and suicidal ideation during two psychosocial treatments in older adults with major depression and dementia. Journal of Alzheimer's Disease 48: 453-62.

22. Devilly GJ, Borkovec TD (2000) Psychometric properties of the credibility/expectancy questionnaire. Journal of Behavioural Therapy and Experimental Psychiatry 31: 73-86.

23. Kitwood T (1997). Dementia reconsidered: the person comes first. Open University Press, Buckingham.

24. Clare L, Linden DE, Woods RT, Whitaker R, Evans SJ, Parkinson CH, van Paasschen J, Nelis SM, Hoare Z, Yuen KS, Rugg MD. Goal-oriented cognitive rehabilitation for people with early-stage Alzheimer disease: a single-blind randomized controlled trial of clinical efficacy. Am J Geriatr Psychiatry. 2010;5: 928–939.

25. Baltes PB, Baltes MM. Psychological perspectives on successful aging: the model of selective optimization with compensation. In: PB Baltes & MM Baltes, eds. Successful Aging: Perspectives from the Behavioral Sciences. Cambridge, United Kingdom: Cambridge University Press. 1990. pp.1–34.

26. Alexopoulos GS, Abrams RC, Young RC, Shamoian CA (1988) Cornell scale for depression in dementia. Biological Psychiatry 23: 271-84.

27. Smith SC, Lamping DL, Banerjee S, Harwood RH, Foley B, Smith P, Cook JC, Murray J, Prince M, Levin E, Mann A, Knapp M (2007) Development of a new measure of health-related quality of life for people with dementia: DEMQOL. Psychological Medicine 37: 737-47.

28. EuroQoL Group (1990) EuroQol – a new facility for the measurement of health-related quality of life. Health Policy 16: 199-208.

29. Bucks RS, Ashworth DI, Wilcock GK, et al. **(**1996**)** Assessment of activities of daily living in dementia: development of the Bristol Activities of Daily Living scales. Age and Ageing 25: 113-120.

30. Shankar K, Walker M, Frost D, Orrell M (1999) The development of a valid and reliable scale for rating anxiety in dementia (RAID) Aging and Mental Health 16: 592-602.

31. Beecham JK, Knapp MRJ (1992) Costing psychiatric interventions. In: Thornicroft G, Brewin C, Wing JK (eds) Measuring Mental Health Needs. Gaskell. London. 200-224.

32. Zarit SH, Reever KE, Bach-Peterson J (1980) Relatives of the impaired elderly: Correlates of feelings of burden. Gerontologist 20: 649-55.

33. Goldberg D, Williams P (1988) A user's guide to the General Health Questionnaire. Windsor, UK: NFER-Nelson.

34. Waldorff FB, Buss DV, Eckermann A, et al. (2012). Efficacy of psychosocial intervention in patients with mild Alzheimer's disease: the multicentre, rater blinded, randomised Danish Alzheimer Intervention Study (DAISY). BMJ 345: e4693.

35. Atkinson CC (1982) The client satisfaction questionnaire: Psychometric properties and correlations with service utilization and psychotherapy outcome. Evaluation and Program Planning 5:233-237.

36. Greenwald BS, Kramer-Ginsberg E, Marin DB (1989) Dementia with coexistent major depression. American Journal of Psychiatry 146:1472-8.

37. Burns A, Jacoby R, Levy R (1990) Psychiatric phenomena in Alzheimer's disease III: disorders of mood. British Journal of Psychiatry 157:81-6.

38. Rosenberg PB, Drye LT, Martin BK, Frangakis C, Mintzer JE, Weintraub D, Porsteinsson AP, Schneider LS, Rabins PV, Munro CA, Meinert CL, Lyketsos CG and the DIADS-2 Research Group (2010) Sertraline for the treatment of depression in Alzheimer disease. American Journal of geriatric Psychiatry 18:136-145.

39. Orgeta V, Tabet N, Nilforooshan R, Howard R (2017) Efficacy of antidepressants for depression in Alzheimer's disease: Systematic review and meta-analysis. Journal of Alzheimer's Disease 58:725-733.

40. Kirkham JG, Choi N, Seitz DP (2016) Meta-analysis of problem solving therapy for the treatment of major depressive disorder in older adults. International Journal of Geriatric Psychiatry 31:526-35.

41. Terri L, Logsdon RC, Uomoto J (1997) Behavioural treatment of depression in dementia patients: a controlled clinical trial. Journal of Gerontology B Psychological Science and Sociological Science 52:159-66.

42. Velligan DL, Bow-Thomas CC (2000) Two case studies of cognitive adaptation training for outpatients with schizophrenia. Psychiatric Services 51:25-9.

43. Nezu AM, Perri MG (1989) Social problem-solving therapy for unipolar depression: an initial dismantling investigation. Journal of Consulting Clinical Psychology 57:408-13.

44. D'Zurilla TJ, Nezu AM (1999) Problem-solving therapy: a social competence approach to clinical intervention. Singer. New York.

45. Medical Research Council (2008) Developing and evaluating complex interventions: New guidance.

46. Livingston G, Barber J, Rapaport P, Knapp M, King D, Griffin M, Livingston D, Walker Z, Mummery C, Sampson E, Cooper C (2013) Clinical effectiveness of a manual-based coping strategy programme (START) in promoting the mental health of carers of family members with dementia: pragmatic randomised controlled trial. BMJ 347:f6276.

47. Dechamps, A., Fasotti, L., Jungheim, J., Leone, E., Dood, E., Allioux, A. et al. (2011). Effects of different learning methods for instrumental activities of daily living in patients with Alzheimer’s dementia: A pilot study. American Journal of Alzheimer’s Disease & Other Dementias 26:273-281.

48. McKhann GM, Knopman DS, Chertkow H, Hyman BT, Jack CR, Kawas CH, et al. (2011) The diagnosis of dementia due to Alzheimer’s disease: Recommendations from the National Institute on Aging-Alzheimer’s Association workgroups on diagnostic guidelines for Alzheimer’s disease. Alzheimer’s and Dementia 7:263-269.

49. Cooper C, Bebbington P, McManus S, et al. (2010) The treatment of common mental disorders across age groups: results from the 2007 Adult Psychiatric Morbidity Survey. Journal of Affective Disorders 127:96-101.

50. Pettit S, Qureshi A, Lee W, Stirzaker A, Gibson A, Henley W, Byng R (2017) Variation in referral and access to new psychological therapy services by age: an empirical quantitative study. British Journal of General Practice, Online First.

51. Almeida OP, MacLeod C, Flicker L, Ford A, Grafton B, Etherton-Beer C (2014) Randomised controlled trial to improve depression and the quality of life of people with dementia using cognitive bias modification: RAPID study protocol. BMJ Open 4:e005623.

52. Treweek S, Pitkethly M, Cook J, et al. Strategies to improve recruitment to randomised controlled trials. Cochrane Database of Systematic Reviews 2010.

53. Nezu A, Nezu C. Evidence-based outcome research: A practical guide to conducting randomized controlled trials for psychosocial interventions. Oxford University Press: New York; 2008.

54. Braun V, Clarke V (2006) Using thematic analysis in psychology. Qualitative Research in Psychology 3:77-101.

55. Devlin N, Shah K, Feng Y, Mulhern B, Van Hout B (2016) Valuing Health-Related Quality of Life: An EQ-5D-5L Value Set for England. OHE Research Paper 16/01. London. Office of Health Economics.

56. von Elm E, Altman DG, Egger M, et al (2007) The Strengthening the Reporting of Observational Studies in Epidemiology (STROBE) Statement: Guidelines for reporting observational studies. Lancet 370:1453-1457.

57. Tong A, Sainsbury P, Craig J (2007) Consolidated criteria for reporting qualitative research (COREQ): a 32-item checklist for interviews and focus groups. International Journal for Quality in Health Care 19:349-357.

58. Boutron I, Moher D, Altman DG, et al for the CONSORT Group (2008) Methods and processes of the CPNSORT Group: example of an extension for trials assessing nonpharmacologic treatments. Annals of Internal Medicine W60-W67.

59. Curtis L. Unit costs of health and social care 2017. Personal Social Services Research Unit, 2017.

60. Department of Health. 2015–2016 NHS reference costs. Department of Health, 2016.

23 APPENDICES

Appendix 1: Flowchart

Phase 1 (months 1-12): Adaptation & acceptability

Months 1-6: Adapt PATH intervention for delivery in the NHS through interviews with 10-15 patient-carer dyads and 4 workshops.

↓

Months 7-12: Assess acceptability of adapted PATH intervention in 10-15 patient-carer dyads.

↓

Phase 2 (months 13-54): Full RCT including an internal pilot phase in months 13-24

Months 13-48: Identified and approached about RCT (n = 668). Patients ineligible or potentially eligible but declined to participate (n = 334)

↓

Consented and randomised[e] (n = 334)

↓

Allocated to PATH plus multidisciplinary TAU (n = 167)	Allocated to multidisciplinary TAU alone (n = 167)
n = 150 (10% loss to follow-up at 12 weeks post-randomisation)	n = 149 (10% loss to follow-up at 12 weeks post-randomisation)
n = 134 (20% loss to follow-up at 6 months post-randomisation [primary endpoint])	n = 133 (20% loss to follow-up at 6 months post-randomisation [primary endpoint])
n = 117 (30% loss to follow-up at 12 months post-randomisation)	n = 116 (30% loss to follow-up at 12 months post-randomisation
Months 49-54: Final analysis, write up and dissemination	*Months 49-54:* Final analysis, write up and dissemination

Appendix 2: Timetable for the PATHFINDER study (01/05/2018-31/10/2022)

	Dates	Nov 2017 -Apr 2018	May 2018 -Jul 2018	Aug 2018 -Oct 2018	Nov 2018 -Jan 2019	Feb 2019 -Apr 2019	May 2019 -Jul 2019	Aug 2019 -Oct 2019	Nov 2019 -Jan 2020	Feb 2020 -Apr 2020	May 2020 -Jul 2020	Aug 2020 -Oct 2020	Nov 2020 -Jan 2021	Feb 2021 -Apr 2021	May 2021 -Jul 2021	Aug 2021 -Oct 2021	Nov 2021 -Jan 2022	Feb 2022 -Apr 2022	May 2022 -Jul 2022	Aug 2022 -Oct 2022
	**Months**	**-6-0**	**1-3**	**4-6**	**7-9**	**10-12**	**13-15**	**16-18**	**19-21**	**22-24**	**25-27**	**28-30**	**31-33**	**34-36**	**37-39**	**40-42**	**43-45**	**46-48**	**49-51**	**52-54**
**Phase**	**Activity**																			
0	Apply for HRA approvals																			
0	Set up recruiting sites																			
1	Orientation																			
1	Adapt intervention																			

1	Assess intervention acceptability																			
1	Modify intervention																			
1	Data analysis																			
1	Train therapists																			
2	Recruitment for RCT (incl. internal pilot in months 13-24)																			
2	Deliver PATH (incl. booster sessions)																			

2	Collect data																			
2	Database lock, data analysis																			
2	Write up & dissemination																			
2	Revise intervention																			

Note: Phase 0 = pre-orientation (prior to study start date); Phase 1 = adapt intervention and assess its acceptability; Phase 2 = RCT (assessments at 0, 3, 6 and 12 months).

Appendix 3: Bristol Activities of Daily Living Scale:

Date of assessment: ..../ ..../........................ (DD/MMM/YYYY)

This questionnaire is designed to reveal the everyday ability of people who have memory difficulties of one form or another.

For each activity (No. 1 - 20), statements a - e refer to a different level of ability.

Thinking of the last 2 weeks, tick the box that represents your relative's/friend's AVERAGE ability. (If in doubt about which box to tick, choose the level of ability which represents their average performance over the last 2 Weeks. Tick 'Not applicable' if your relative never did that activity when they were well).

1. PREPARING FOOD		a) Selects and prepares food as required
		b) Able to prepare food if ingredients set out
		c) Can prepare food if prompted step by step
		d) Unable to prepare food even with prompting and supervision
		e) Not applicable
2. EATING		a) Eats appropriately using correct cutlery
		b) Eats appropriately if food made manageable and/or uses spoon
		c) Uses fingers to eat food
		d) Needs to be fed
		e) Not applicable
3. PREPARING DRINK		a) Selects and prepares drinks as required
		b) Can prepare drinks if ingredients left available
		c) Can prepare drinks if prompted step by step
		d) Unable to make a drink even with prompting and supervision
		e) Not applicable
4. DRINKING		a) Drinks appropriately
		b) Drinks appropriately with aids, beaker/straw etc.
		c) Does not drink appropriately even with aids but attempts to
		d) Has to have drinks administered (fed)
		e) Not applicable

5. DRESSING		a) Selects appropriate clothing and dresses self
		b) Puts clothes on in wrong order and/or back to front and/or dirty clothing
		c) Unable to dress self but moves limbs to assist
		d) Unable to assist and requires total dressing
		e) Not applicable
6. HYGIENE		a) Washes regularly and independently
		b) Can wash self if given soap, flannel, towel, etc.
		c) Can wash self if prompted and supervised
		d) Unable to wash self and needs full assistance
		e) Not applicable
7. TEETH		a) Cleans own teeth/dentures regularly and independently
		b) Cleans teeth/dentures if given appropriate items
		c) Requires some assistance, toothpaste on brush, brush to mouth etc.
		d) Full assistance given
		e) Not applicable
8. BATH/SHOWER		a) Bathes regularly and independently
		b) Needs bath to be drawn/shower turned on but washes independently
		c) Needs supervision and prompting to wash
		d) Totally dependent, needs full assistance
		e) Not applicable
9. TOILET/COMMODE		a) Uses toilet appropriately when required
		b) Needs to be taken to the toilet and given assistance
		c) Incontinent of urine or faeces
		d) Incontinent of urine and faeces
		e) Not applicable
10. TRANSFERS		a) Can get in/out of chair unaided
		b) Can get into a chair but needs help to get out
		c) Needs help getting in and out of a chair
		d) Totally dependent on being put into and lifted from chair
		e) Not applicable
11. MOBILITY		a) Walks independently
		b) Walks with assistance i.e. furniture, arm for support
		c) Uses aids to mobilise i.e. frame, sticks etc.
		d) Unable to walk
		e) Not applicable

12. ORIENTATION – TIME		a) Fully orientated to time/day/date etc.
		b) Unaware of time/day etc. but seems unconcerned
		c) Repeatedly asks the time/day/date
		d) Mixes up night and day
		e) Not applicable
13. ORIENTATION – SPACE		a) Fully orientated to surroundings
		b) Orientated to familiar surroundings only
		c) Gets lost in home, needs reminding where bathroom is, etc.
		d) Does not recognise home as own and attempts to leave
		e) Not applicable
14. COMMUNICATION		a) Able to hold appropriate conversation
		b) Shows understanding and attempts to respond verbally with gestures
		c) Can make self understood but difficulty understanding others
		d) Does not respond to, or communicate with others
		e) Not applicable
15. TELEPHONE		a) Uses telephone appropriately, including obtaining correct number
		b) Uses telephone if number given verbally/visually or predialled
		c) Answers telephone but does not make calls
		d) Unable/unwilling to use telephone at all
		e) Not applicable
16. HOUSEWORK/ GARDENING		a) Able to do housework/gardening to previous standard
		b) Able to do housework/gardening but not to previous standard
		c) Limited participation with a lot of supervision
		d) Unwilling/unable to participate in previous activities
		e) Not applicable
17. SHOPPING		a) Shops to previous standard
		b) Only able to shop for 1 or 2 items with or without a list
		c) Unable to shop alone, but participates when accompanied
		d) Unable to participate in shopping even when accompanied
		e) Not applicable
18. FINANCES		a) Responsible for own finances at previous level
		b) Unable to write cheque. Can sign name & recognises money values
		c) Can sign name but unable to recognise money values
		d) Unable to sign name or recognise money values
		e) Not applicable
19. GAMES/HOBBIES		a) Participates in pastimes/activities to previous standard
		b) Participates but needs instruction/supervision
		c) Reluctant to join in, very slow needs coaxing
		d) No longer able or willing to join in
		e) Not applicable

20. TRANSPORT		a) Able to drive, cycle or use public transport independently
		b) Unable to drive but uses public transport or bike etc.
		c) Unable to use public transport alone
		d) Unable/unwilling to use transport even when accompanied
		e) Not applicable

Appendix 4: The Zarit Burden Interview

The Zarit Burden Interview for Caregivers

Scoring system

0 : NEVER
1 : RARELY
2 : SOMETIMES
3 : QUITE FREQUENTLY
4 : NEARLY ALWAYS

Please circle the response that best describes how you feel. **Only one response should be circled per statement**.

Question		Score				
1	Do you feel that your relative asks for more help than he/she needs?	0	1	2	3	4
2	Do you feel that because of the time you spend with your relative that you don't have enough time for yourself?	0	1	2	3	4
3	Do you feel stressed between caring for your relative and trying to meet other responsibilities for your family or work?	0	1	2	3	4
4	Do you feel embarrassed over your relative's behaviour?	0	1	2	3	4
5	Do you feel angry when you are around your relative?	0	1	2	3	4
6	Do you feel that your relative currently affects our relationships with other family members or friends in a negative way?	0	1	2	3	4
7	Are you afraid what the future holds for your relative?	0	1	2	3	4
8	Do you feel your relative is dependent on you?	0	1	2	3	4
9	Do you feel strained when you are around your relative?	0	1	2	3	4
10	Do you feel your health has suffered because of your involvement with your relative?	0	1	2	3	4
11	Do you feel that you don't have as much privacy as you would like because of your relative?	0	1	2	3	4
12	Do you feel that your social life has suffered because you are caring for your relative?	0	1	2	3	4
13	Do you feel uncomfortable about having friends over because of your relative?	0	1	2	3	4
14	Do you feel that your relative seems to expect you to take care of him/her as if you were the only one he/she could depend on?	0	1	2	3	4

15	Do you feel that you don't have enough money to take care of your relative in addition to the rest of your expenses?	0	1	2	3	4
16	Do you feel that you will be unable to take care of your relative much longer?	0	1	2	3	4
17	Do you feel you have lost control of your life since your relative's illness?	0	1	2	3	4
18	Do you wish you could leave the care of your relative to someone else?	0	1	2	3	4
19	Do you feel uncertain about what to do about your relative?	0	1	2	3	4
20	Do you feel you should be doing more for your relative?	0	1	2	3	4
21	Do you feel you could do a better job in caring for your relative?	0	1	2	3	4
22	Overall, how burdened do you feel in caring for your relative?	0	1	2	3	4

Interpretation of Score:

0 - 21 little or no burden

21 - 40 mild to moderate burden

41 - 60 moderate to severe burden

61 - 88 severe burden

Appendix 5: Cornell Scale for Depression in Dementia

Cornell Scale for Depression in Dementia

The Cornell Scale for Depression in Dementia (CSDD) was specifically developed to assess signs and symptoms of major depression in patients with dementia. Because some of these patients may give unreliable reports, the CSDD uses a comprehensive interviewing approach that derives information from the patient and the informant. Information is elicited through two semi-structured interviews; an interview with an informant and an interview with the patient. Based on these interviews, the interviewer can score the CSDD by assigning a preliminary score to each item of the scale on the basis of the informant's report in the "Informant" column. The next step is for the rater to interview the patient using the Cornell scale items as a guide. The interviews focus on depressive symptoms and signs occurring during the week preceding the interview. Many of the items during the patient interview can be filled after direct observation of the patient. If there are discrepancies in ratings generated from the informant and the patient interviews, the rater should re-interview both the informant and the patient to resolve the discrepancies. The final ratings of the CSDD items represent the rater's clinical impression rather than the responses of the informant or the patient. The CSDD takes approximately 20 minutes to administer.

Scoring System: **A = unable to evaluate 0 = absent 1 = mild or intermittent 2 = severe**

No score (0) should be given if symptoms result from physical disability or illness.

		Caregiver	Patient	**Final score**
	**A. Mood-Related Signs**			
1	Anxiety: anxious expression, ruminations, worrying			
2	Sadness: sad expression, sad voice, tearfulness			
3	Lack of reactivity to pleasant events			
4	Irritability: easily annoyed, short-tempered			
	**B. Behavioural disturbance**			
5	Agitation: restlessness, handwringing, hairpulling			
6	Retardation: slow movement, slow speech, slow reactions			
7	Multiple physical complaints (score 0 if GI symptoms only)			
8	Loss of interest: less involved in usual activities (score only if change occurred acutely, i.e. in less than 1 month			
	**C. Physical Signs**			
9	Appetite loss: eating less than usual			
10	Weight loss (score 2 if greater than 5lb in 1 month)			
11	Lack of energy: fatigues easily, unable to sustain activities (score only if occurred acutely i.e. in less than 1 month)			
	**D. Cyclical Functions**			
12	Diurnal variation of mood: symptoms worse in the morning			
13	Difficulty falling asleep: later than usual for this individual			
14	Multiple awakenings during sleep			
15	Early morning awakening: earlier than usual for this individual			
	**E. Ideational Disturbance**			
16	Suicide: feels that life is not worth living, has suicidal wishes, or makes suicide attempt			
17	Poor self-esteem: self-blame, self- depreciation, feelings of failure			
18	Pessimism: anticipation of the worst			
19	Mood congruent delusions: delusions of poverty, illness or loss			
			Total	

Appendix 6: DEMQOL

DEMQOL

Instructions: *Read each of the following questions (in bold) verbatim and show the respondent the response card.*

I would like to ask you about your life. There are no right or wrong answers. Just give the answer that best describes how you have felt in the last week. Don't worry if some questions appear not to apply to you. We have to ask the same questions of everybody.

Before we start we'll do a practice question; that's one that doesn't count. *(Show the response card and ask respondent to say or point to the answer)***. In the last week how much have you enjoyed watching television?**

a lot **quite a bit** **a little** **not at all**

Follow up with a prompt question: **Why is that?** *or* **Tell me a bit more about that.**

For all of the questions I'm going to ask you, I want you to think about the last week.

First I'm going to ask you about <u>your feelings</u>. In the last week, have you felt.....

**1.**	**cheerful? ****	☐ a lot	☐ quite a bit	☐a little	☐ not at all
**2.**	**worried or anxious?**	☐ a lot	☐ quite a bit	☐ a little	☐ not at all
**3.**	**that you are enjoying life? ****	☐ a lot	☐ quite a bit	☐ a little	☐ not at all
**4.**	**frustrated?**	☐ a lot	☐ quite a bit	☐ a little	☐ not at all
**5.**	**confident? ****	☐ a lot	☐ quite a bit	☐ a little	☐ not at all
**6.**	**full of energy? ****	☐ a lot	☐ quite a bit	☐ a little	☐ not at all
**7.**	**sad?**	☐ a lot	☐ quite a bit	☐ a little	☐ not at all
**8.**	**lonely?**	☐ a lot	☐ quite a bit	☐ a little	☐ not at all
**9.**	**distressed?**	☐ a lot	☐ quite a bit	☐ a little	☐ not at all
**10.**	**lively? ****	☐a lot	☐ quite a bit	☐ a little	☐ not at all
**11.**	**irritable?**	☐ a lot	☐ quite a bit	☐ a little	☐ not at all
**12.**	**fed up?**	☐ a lot	☐ quite a bit	☐ a little	☐ not at all
**13.**	**that there are things that you wanted to do but couldn't?**	☐ a lot	☐ quite a bit	☐ a little	☐ not at all

Next, I'm going to ask you about <u>your memory.</u> In the last week, how worried have you been about....

14. forgetting things that happened recently?	□ a lot	□ quite a bit	□ a little	□ not at all
15. forgetting who people are?	□ a lot	□ quite a bit	□ a little	□ not at all
16. forgetting what day it is?	□ a lot	□ quite a bit	□ a little	□ not at all
17. your thoughts being muddled?	□ a lot	□ quite a bit	□ a little	□ not at all
18. difficulty making decisions?	□ a lot	□ quite a bit	□ a little	□ not at all
19. poor concentration?	□ a lot	□ quite a bit	□ a little	□ not at all

Now, I'm going to ask about <u>your everyday life.</u> In the last week, how worried have you been about......

20. not having enough company?	□ a lot	□ quite a bit	□ a little	□ not at all
21. how you get on with people close to you?	□a lot	□ quite a bit	□ a little	□ not at all
22. getting the affection that you want?	□ a lot	□ quite a bit	□ a little	□ not at all
23. people not listening to you?	□ a lot	□ quite a bit	□ a little	□ not at all
24. making yourself understood?	□ a lot	□ quite a bit	□ a little	□ not at all
25. getting help when you need it?	□ a lot	□ quite a bit	□ a little	□ not at all
26. getting to the toilet in time?	□ a lot	□quite a bit	□ a little	□ not at all
27. how you feel in yourself?	□ a lot	□quite a bit	□ a little	□ not at all
28. your health overall?	□ a lot	□ quite a bit	□ a little	□ not at all

We've already talked about lots of things: your feelings, memory and everyday life. Thinking about all of these things in the last week, how would you rate .....

**29.** your quality of life overall? **	□ very good	□ good	□ fair	□ poor

** items that need to be reversed before scoring

Appendix 7: DEMQOL (caregiver)

DEMQOL - Caregiver

Instructions: *Read each of the following questions (in bold) verbatim and show the respondent the response card.*

I would like to ask you about__________________ *(your relative's)* **life, as you are the person who knows him/her best. There are no right or wrong answers. Just give the answer that best describes how__________________** *(your relative)* **has felt in the last week. If possible try and give the answer that you think__________________** *(your relative)* **would give. Don't worry if some questions appear not to apply to __** *(your relative).* **We have to ask the same questions of everybody.**

Before we start we'll do a practice question; that's one that doesn't count. *(Show the response card and ask respondent to say or point to the answer)***. In the last week how much has __________________***(your relative)* **enjoyed watching television?**

a lot **quite a bit** **a little** **not at all**

Follow up with a prompt question: **Why is that?** *or* **Tell me a bit more about that.**

For all of the questions I'm going to ask you, I want you to think about the last week.

First I'm going to ask you about __________________*(your relative's)* **<u>feelings.</u> In the last week, would you say that**

(your relative) **has felt: .**

**1.**	**cheerful? ****	□ a lot	□ quite a bit	□a little	□ not at all
**2.**	**worried or anxious?**	□ a lot	□ quite a bit	□ a little	□ not at all
**3.**	**frustrated?**	□ a lot	□ quite a bit	□ a little	□ not at all
**4.**	**full of energy? ****	□ a lot	□ quite a bit	□ a little	□ not at all
**5.**	**sad?**	□ a lot	□ quite a bit	□ a little	□ not at all
**6.**	**content? ****	□ a lot	□ quite a bit	□ a little	□ not at all
**7.**	**distressed?**	□ a lot	□ quite a bit	□ a little	□ not at all
**8.**	**lively? ****	□ a lot	□ quite a bit	□ a little	□ not at all
**9.**	**irritable?**	□ a lot	□ quite a bit	□ a little	□ not at all
**10.**	**fed-up**	□a lot	□ quite a bit	□ a little	□ not at all
**11.**	**that he/she has things to look forward to? ****	□ a lot	□ quite a bit	□ a little	□ not at all

Next, I'm going to ask you about _________________ *(your relative's)* **<u>memory.</u> In the last week, how worried would you say**

___________*(your relative)* **has been about:**

12. his/her memory in general?	□ a lot	□ quite a bit	□ a little	□ not at all
13. forgetting things that happened a long time ago?	□ a lot	□ quite a bit	□ a little	□ not at all
14. forgetting things that happened recently?	□ a lot	□ quite a bit	□ a little	□ not at all
15. forgetting people's names?	□ a lot	□ quite a bit	□ a little	□ not at all
16. forgetting where he/she is?	□ a lot	□ quite a bit	□ a little	□ not at all
17. forgetting what day it is?	□ a lot	□ quite a bit	□ a little	□ not at all
18. his/her thoughts being muddled?	□ a lot	□ quite a bit	□ a little	□ not at all
19. difficulty making decisions?	□ a lot	□quite a bit	□ a little	□ not at all
20. making him/herself understood?	□ a lot	□ quite a bit	□a little	□ not at all

Now, I'm going to ask about__________________ *(your relative's)* <u>everyday life.</u> In the last week, how worried would you say

__________ *(your relative)* has been about:

21. keeping him/herself clean (eg washing and bathing)?	□ a lot	□ quite a bit	□ a little	□ not at all
22. keeping him/herself looking nice?	□a lot	□ quite a bit	□ a little	□ not at all
23. getting what he/she wants from the shops	□ a lot	□ quite a bit	□ a little	□ not at all
24. using money to pay for things?	□ a lot	□ quite a bit	□ a little	□ not at all

**25. looking after his/her finances?**	□ a lot	□ quite a bit	□ a little	□ not at all
**26. things taking longer than they used to?**	□ a lot	□ quite a bit	□ a little	□ not at all
**27. getting in touch with people?**	□ a lot	□quite a bit	□ a little	□ not at all
**28. not having enough company?**	□ a lot	□quite a bit	□ a little	□ not at all
**29. not being able to help other people?**	□ a lot	□ quite a bit	□ a little	□ not at all
**30. not playing a useful part in things?**	□ a lot	□ quite a bit	□ a little	□ not at all
**31. his/her physical health?**	□ a lot	□ quite a bit	□ a little	□ not at all

We've already talked about lots of things: ___________________ *(your relative's)* **feelings, memory and everyday life. Thinking about all of these things in the last week, how would you say ________________***(your relative)* **would rate ..**

**32.** his/her quality of life overall? **	□ very good	□ good	□ fair	□ poor

** items that need to be reversed before scoring

Appendix 8: Rating Anxiety In Dementia

Rating Anxiety In Dementia - RAID

Scoring system:

U. unable to evaluate. 0. absent. 1. mild or intermittent. 2. moderate. 3. severe

Rating should be based on symptoms and signs occurring during two weeks prior to the interview.

No score should be given if symptoms result from physical disability or illness.

Total score is the sum of items 1-18. A score of 11 or more suggests significant clinical anxiety

			Score
Worry	1	Worry about physical health.	
	2	Worry about cognitive performance (failing memory, getting lost when goes out, not able to following conversation).	
	3	Worry over finances, family problems, physical health of relatives.	
	4	Worry associated with false belief and/or perception.	
	5	Worry over trifles (repeatedly calling for attention over trivial matters).	
Apprehension and vigilance	6	Frightened and anxious (keyed up and on the edge).	
	7	Sensitivity to noise (exaggerated startle response).	
	8	Sleep disturbance (trouble falling or staying asleep).	
	9	Irritability (more easily annoyed than usual, short tempered and angry outbursts).	
Motor tension	10	Trembling.	
	11	Motor tension (complain of headache, other body aches and pains).	

	12	Restlessness (fidgeting, cannot sit still, pacing, wringing hands, picking clothes).	
	13	Fatigueability, tiredness.	
Autonomic hypersensitivity	14	Palpitations (complains of heart racing or thumping).	
	15	Dry mouth (not due to medication), sinking feeling in the stomach.	
	16	Hyperventilating, shortness of breath (even when not exerting).	
	17	Dizziness or light-headedness (complains as if going to faint).	
	18	Sweating, flushes or chills, tingling or numbness of fingers and toes.	
Phobias: (fears which are excessive, that do not make sense and tend to avoid- like afraid of crowds, going out alone, being in a small room, or being frightened by some kind of animals, heights, etc.) Describe.			
Panic attacks: (Feelings of anxiety or dread that are so strong that think they are going to die or have a heart attack and they simply have to do something to stop them, like immediately leaving the place, phoning relatives, etc.) Describe.			

Appendix 9: General Health Questionnaire (GHQ-12)

General Health Questionnaire (GHQ-12) to be given to caregivers

We want to know if you have any medical complaints and how your health has been in general, *over the past few weeks*, please answer all the questions simply by circling the response that best applies to you. Remember that we want to know about present and recent complaints not those you had in the more remote past.

Have you recently:

1. **Been able to concentrate on what you are doing**
   Better than usual (0) same as usual (1) less than usual (2) much less than usual (3)

2. **Lost much sleep over worry**
   Not at all (0) no more than usual (1) rather more than usual (2) much more than usual (3)

3. **Felt you were playing a useful part in things**
   More so than usual (0) same as usual (1) less so than usual (2) much less than usual (3)

4. **Felt capable of making decisions about things**
   More so than usual (0) same as usual (1) less so than usual (2) much less than usual (3)

5. **Felt constantly under strain**
   Not at all (0) no more than usual (1) rather more than usual (2) much more than usual (3)

6. **Felt you couldn't overcome your difficulties**
   Not at all (0) no more than usual (1) rather more than usual (2) much more than usual (3)

7. **Been able to enjoy your normal day-to-day activities**
More so than usual same as usual less so than usual much less than usual
(0) (1) (2) (3)

8. **Been able to face up to your problems**
More so than usual same as usual less so than usual much less than usual
(0) (1) (2) (3)

9. **Been feeling unhappy and depressed**
Not at all no more than usual rather more than usual much more than usual
(0) (1) (2) (3)

10. **Been losing confidence in yourself**
Not at all no more than usual rather more than usual much more than usual
(0) (1) (2) (3)

11. **Been thinking of yourself as a worthless person**
Not at all no more than usual rather more than usual much more than usual
(0) (1) (2) (3)

12. **Been feeling reasonably happy, all things considered**
More so than usual same as usual less so than usual much less than usual
(0) (1) (2) (3)

Appendix 10: Standardised Mini Mental State Examination

STANDARDIZED MINI-MENTAL STATE EXAMINATION (SMMSE)

Directions for the administration of the SMMSE

1. Before the questionnaire is administered, try to get the person to sit down facing you. Assess the person's ability to hear and understand very simple conversation, e.g. "what is your name?" If the person uses hearing or visual aids, provide these before starting.
2. Introduce yourself and try to get the person's confidence. Before you begin, get the person's permission to ask questions, e.g. "would it be all right to ask you some questions about your memory?" This helps to avoid catastrophic reactions.
3. Ask each question a maximum of three times. If the subject does not respond, score 0.
4. If the person answers incorrectly, score 0. Accept that answer and do not ask the question again, hint, or provide any physical clues as head shaking, etc.
5. The following equipment is required to administer the instrument:
    - A wristwatch
    - A pencil
    - Page 5 of this document (with CLOSE YOUR EYES written in large letters and two five sided figures intersecting to make a four sided figure)
    - Page 8 (a blank piece of paper)
6. If the person answers "What did you say?", do not explain or engage in conversation, merely repeat the same directions a maximum of three times.
7. If the person interrupts (e.g. "what is this for?"), just reply "I will explain in a few minutes, when we are finished. Now if we could proceed please… we are almost finished."

Say: I am going to ask you some questions and give you some problems to solve. Please try to answer as best you can.

NHS National Institute for Health Research

Camden and Islington NHS Foundation Trust

**1. (Allow 10 seconds for each reply)**	
a) What year is this? (accept exact answer only)	/1
b) What season is this? (during the last week of the old season or first week of a new season, accept either)	/1
c) What month is this? (on the first day of a new month or the last day of the previous month, accept either)	/1
d) What is today's date? (accept previous or next date)..........	/1
e) What day of the week is this? (accept exact answer only)	/1
**2. (Allow 10 seconds for each reply)**	
a) What country are we in? (accept exact answer only)	/1
b) What province or state are we in? (accept exact answer only)..........	/1
c) What city/town are we in? (accept exact answer only)	/1
d) (In home) What is the street address of this house?(accept street name and house number or equivalent in rural areas) (In facility) What is the name of this buliding? (accept exact name of institution only)..........	/1
e) (In home) What room are we in?(accept exact answer only) (In facility) What floor of the buliding are we on?(accept exact answer only)..........	/1
**3. Say:** I am going to name three objects. When I am finished, I want you to repeat them. Remember what they are because I am going to ask you to name them again in a few minutes. (say slowly at approximatly one-second intervals)<br>Ball Car Man<br>For repeated use: Bell, jar, fan; Bill, tar, can; Bull, bar, pan<br>Please repeat the three items for me. (score one point for each correct reply on the first attempt)	/3
Allow 20 seconds for reply; if the person did not repeat all three, repeat until they are learned or up to a maximum of five times. (but only score first attempt)	
**4. Spell the word WORLD.** (you may help the person to spell the word correctly) Say: Now spell it backwards please.. (allow 30 seconds; if the subject cannot spell world even with assistance, score 0) Refer to Page 2 for scoring instructions	/5
**5. Say:** *Now what were the three objects I asked you to remember?* .... (score one point for each correct answer regardless of order; allow 10 seconds)	/3
**6. Show wristwatch.** Ask: *What is this called?* .... (score one point for correct response; accept "wristwatch" or "watch"; do not accept "clock" or "time", etc.; allow 10 seconds)	/1
**7. Show pencil.** Ask: *What is this called?* .... (score one point for correct response; accept "pencil" only; score 0 for pen; allow 10 seconds for reply)	/1
**8. Say:** *I would like you to repeat a phrase after me: No ifs, ands, or buts*.......... (allow 10 seconds for response. Score one point for a correct repetition. Must be exact, e.g. no ifs or buts, score 0)	/1
**9. Say:** Read the words on this page and then do what it says.......... Then, hand the person the sheet with CLOSE YOUR EYES on it. If the subject just reads and does not close eyes, you may repeat: Read the words on this page and then do what it says, (a maximum of three times. This is covered in #3 directions section above). Allow 10 seconds, score one point only if the subject closes eyes. The subject does not have to read aloud.	/1
**10. Hand** the person a pencil and paper (Page 3). Say: Write any complete sentence on that piece of paper. Allow 30 seconds. Score one point. The sentence must make sense. Ignore spelling errors.	/1
**11. Place** design, pencil, eraser and paper in front of the person. Say: Copy this design please. Allow multiple tries. Wait until the person is finished and hands it back. Score one point for a correctly copied diagram. The person must have drawn a four-sided figure between two five-sided figures. Maximum time: One minute.	/1
**12. Ask** the person if he is right or left handed. Take a piece of paper, hold it up in front of the person and say the following: Take this paper in your right/left hand (whichever is non-dominant), fold the paper in half once with both hands and put the paper down on the floor. Allow 30 seconds. Score one point for each instruction executed correctly.	
Takes paper in correct hand..........	/1
Folds it in half..........	/1
Puts it on the floor..........	/1
TOTAL TEST SCORE:	/30
ADJUSTED SCORE:	/

This questionnaire should not be modified or reproduced without the written consent of Dr. D. William Molloy.
Molloy DW, Alemayehu E, Roberts R. Reliability of a standardized Mini-Mental State Examination compared with the traditional Mini-Mental state Examination. American Journal of Psychiatry, Vol. 14, 1991a, pp.102-105.

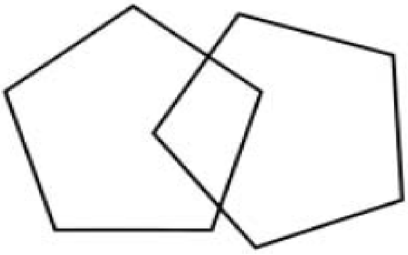

Scoring WORLD backwards (scoring instructions for item #4)
Write the person's response below the correct response.
Draw lines matching the same letters in the correct response and the response given.
These lines MUST NOT cross each other.
The person's score is the maximum number of lines that can be drawn without crossing any.

Examples:

D	L	R	O	W		
D	L	R	O	W	=	Score 5

D	L	R	O	W		
D	R	W	O	D	=	Score 3

D	L	R	O	W		
L	O	W	R	O	=	Score 3

D	L	R	O	W		
L					=	Score 1

D	L	R	O	W		
L	R	R	W	O	=	Score 3

D	L	R	O	W	
					=

Fold here and show instructions to person

Close Your Eyes

Item 10: Sentence Writing

Appendix 11: EQ-5D

EQ-5D

<u>Instructions for person with memory problems:</u>
Under each heading, please tick the ONE box that best describes your health TODAY.

1. Mobility
I have no problems in walking about ❑
I have slight problems in walking about ❑
I have moderate problems in walking about ❑
I have severe problems in walking about ❑
I am unable to walk about ❑

2. Self-Care
I have no problems washing or dressing myself ❑
I have slight problems washing or dressing myself ❑
I have moderate problems washing or dressing myself ❑
I have severe problems washing or dressing myself ❑
I am unable to wash or dress myself ❑

3. Usual Activities *(e.g. work, study, housework, family or leisure activities)*
I have no problems doing my usual activities ❑
I have slight problems doing my usual activities ❑
I have moderate problems doing my usual activities ❑
I have severe problems doing my usual activities ❑
I am unable to do my usual activities ❑

4. Pain / Discomfort
I have no pain or discomfort ❑
I have slight pain or discomfort ❑
I have moderate pain or discomfort ❑
I have severe pain or discomfort ❑
I have extreme pain or discomfort ❑

5. Anxiety / Depression
I am not anxious or depressed ❑
I am slightly anxious or depressed ❑
I am moderately anxious or depressed ❑
I am severely anxious or depressed ❑
I am extremely anxious or depressed ❑

We would like to know how good or bad your health is TODAY.

This scale is numbered from 0 to 100.

100 means the best health you can imagine.
0 means the worst health you can imagine.

Mark an X on the scale to indicate how your health is TODAY.

Now, please write the number you marked on the scale in the box below.

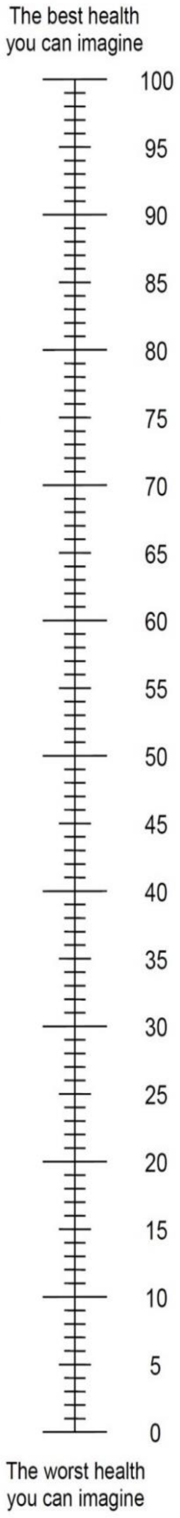

YOUR HEALTH TODAY = ☐

Appendix 12: CSRI

Appendix 12a 3month version

(3 month version to be used at Baseline, 3month and 6 month follow up)

Thinking about the past **3-months**, please complete below for any community based services you have used because of your **dementia** or **depression** symptoms

Primary care and community health		Tick if YES	If yes, total number of contacts in the past 3-months
General practitioner	At clinic	☐	
	By phone	☐	
	At home	☐	
Practice Nurse	At clinic	☐	
	By phone	☐	
	At home	☐	
Community/district nurse	At clinic	☐	
	By phone	☐	
	At home	☐	
	By phone	☐	
	At home	☐	

Memory Service/ Community Mental Health Team(CMHT)<br><br>Doctor	At clinic	☐I	
	By phone	☐	
	At home	☐	
Memory Service /CMHT<br><br>Nurse. Occupational Therapist or Social worker (please specify)	At clinic	☐	
	By phone	☐	
	At home	☐	
Memory Service/CMHT Psychologist	At clinic	☐	
	By phone	☐	
	At home	☐	
Memory Service/CMHT<br><br>Assistant Practitioner	At clinic	☐	
	By phone	☐	
	At home	☐	
Other community Practitioner (*please specify*)	At clinic	☐	
	By phone	☐	
	At home	☐	

Thinking about the past **3-months**, have you had any psychological therapy (appointments with a psychologist, counsellor or therapist) because of your **dementia** or **depression** symptoms? (this includes any contacts with IAPT services)

No ☐ Please skip ahead to complete the **secondary care services** section below.

Yes ☐ Please give more details below.

Was this group or individual therapy (please circle as appropriate)	**Individual / Group**
How many sessions of **individual** therapy **(1 to 1 with a therapist)** have you received in the last 3 months?	Please state number of sessions …………………..
How many sessions of **group** therapy have you received in the last 3 months?	Please state number of sessions …………………..
Did you pay out of your own pocket for this therapy<br><br>(or with assistance from family, etc.)?	Yes ☐ No ☐<br><br>If yes, please state the cost per session<br><br>(£) ………………………

Thinking about the past **3-months,** please complete below for any secondary care and emergency services you have used

**Emergency services**	Tick if yes	**If yes, total number of contacts in the past 3-months**
NHS direct or "Call 111"	☐	
Emergency call (999)	☐	
Paramedic only	☐	
Paramedic and ambulance to hospital	☐	
A&E attendance WITHOUT ambulance	☐	

**Overnight Inpatient stay**	Number of nights (please enter details separately for each inpatient admission)	Reason for admission
Unplanned (emergency) inpatient hospital stay for physical health reasons	Stay 1 .... nights	....
	Stay 2 .... nights	....
	Stay 3 .... nights	....
Planned (elective) inpatient hospital stay for physical health reasons	Stay 1 .... nights	....
	Stay 2 .... nights	....
	Stay 3 .... nights	....
Inpatient hospital stay for mental health reasons	Stay 1 .... nights	....
	Stay 2...................... nights	....

Stay 3………………..…… nights ……………..…………………..……

**Outpatient contacts**	Tick if YES	Number of attendances in last 3 months
General medical outpatient appointment	☐	
Day patient procedure/test	☐	
Hospital Memory clinic	☐	
Psychiatric outpatient appointment	☐	

**Community Group Support**	Tick if YES	Number of attendances in last 3 months
Memory Group	☐	
Support Group provided for people with Dementia for example by AGEUK	☐	

In the past 3 months, have you gone to a **day centre?**

☐ No – please go to **Adaptations** section below

☐ Yes – please complete details below

If yes, how many times do you go to the day centre per week? ___________visits per week.

Do you pay out of pocket to attend the day centre? If yes, how much do you pay per visit? £____ per visit

Adaptations

Have you had any adaptations or changes to your home including memory aids and alarms in the past 3 months? If so, please complete below.

	**Did you pay out of pocket for this?**	**Approximately how much did this cost in the last 3 months, if known?**

		Did you pay out of pocket for this?	Approximately how much did this cost in the last 3 months, if known?
A. Adaptive equipment	☐ Shower chair<br>☐ Bath board<br>☐ Bath lift<br>☐ Over toilet frame / raised seat<br>☐ Kitchen/bathroom stool<br>☐ Bed rail/lever<br>☐ Hospital/electric bed<br>☐ Riser recliner chair<br>☐ Other, please specify:<br>……………………..………………<br>…………..	☐ No<br>☐ Yes (if yes please specify if known)	………………………………..

		Did you pay out of pocket for this?	Approximately how much did this cost in the last 3 months, if known?
B. Mobility and memory aids	☐ Memory Aids<br>☐ Care alarm<br>☐ Walking stick<br>☐ Crutches<br>☐ Walking frame<br>☐ Walker with wheels<br>☐ Banana board/slide board<br>☐ Mobility Scooter<br>☐ Car adaptations<br>☐ Other, please specify:<br>………………..……………… …………..	☐ No<br>☐ Yes (if yes please specify if known)	………………………..

		Did you pay out of pocket for this?	Approximately how much did this cost in the last 3 months, if known?
C. Home adaptations	☐ Ramps installed<br>☐ Rails, grab-rails<br>☐ Stair-lift<br>☐ Furniture raisers<br>☐ Other renovations, please describe<br>………………………………………………	☐ No<br>☐ Yes (if yes please specify if known)	…………………………………

Medication

Please complete for medications related to your depression or dementia taken in the last 3 months. We are particularly interested in any medication for your mental health including antidepressants (for your mood), anti-anxiety medication to help you relax or keep calm, antipsychotics to reduce troubling thoughts or experiences or night time sleeping tablets you are prescribed.

Drug name	Dose	Units of dose	Frequency	Duration of prescription
1				

2				
3				
4				
5				
6				
7				
8				

We would like to know whether you have received help with any of the following activities. If you do receive help we would like to know *how often you receive help* and *who pays for it*.

Please think about the help you have received **over the past 3 months**.

Have you received any help for:

1. **Maintenance work and odd jobs?**

☐ No - go to question 2.

☐ Yes - please complete details below

Who pays for the care?

**State funded help?**	**If yes, approximately how often?**	**If yes, how long is a typical visit?**
Yes ☐ No ☐	☐ Once a month	☐ Less than 30 minutes
	☐ Once a fortnight	☐ 30 to 60 minutes
	☐ Once a week	☐ One to two hours
	☐ A few times a week	☐ More than two hours
	☐ Daily	If more than two hours please specify ……. hours

**Privately funded (paid out of pocket) help?**	**If yes, approximately how often?**	**If yes, how long is a typical visit?**
Yes ☐ No ☐	☐ Once a month	☐ Less than 30 minutes
	☐ Once a fortnight	☐ 30 to 60 minutes
	☐ Once a week	☐ One to two hours
	☐ A few times a week	☐ More than two hours
	☐ Daily	If more than two hours please specify ….. hours
**Unpaid help (friends/family)?**	**If yes, approximately how often?**	**If yes, how long is a typical visit?**
Yes ☐	☐ Once a month	☐ Less than 30 minutes

No ☐	☐ Once a fortnight	☐ 30 to 60 minutes
	☐ Once a week	☐ One to two hours
	☐ A few times a week	☐ More than two hours
	☐ Daily	If more than two hours please specify ….. hours

2. **Cleaning the house, ironing and cooking?**

☐ No - go to question 3.

☐ Yes - please complete details below

Who pays for the care?

**State funded help?** Yes ☐	**If yes, approximately how often?**	**If yes, how long is a typical visit?**
No ☐	☐ Once a month	☐ Less than 30 minutes
	☐ Once a fortnight	☐ 30 to 60 minutes
	☐ Once a week	☐ One to two hours
	☐ A few times a week	☐ More than two hours
	☐ Daily	If more than two hours please specify ……. hours

**Privately funded (paid out of pocket) help?** Yes ☐	**If yes, approximately how often?**	**If yes, how long is a typical visit?**
No ☐	☐ Once a month	☐ Less than 30 minutes
	☐ Once a fortnight	☐ 30 to 60 minutes
	☐ Once a week	☐ One to two hours
	☐ A few times a week	☐ More than two hours
	☐ Daily	If more than two hours please specify ….. hours

**Unpaid help (friends/family)?**	**If yes, approximately how often?**	**If yes, how long is a typical visit?**
Yes ☐	☐ Once a month	☐ Less than 30 minutes

No ☐	☐ Once a fortnight	☐ 30 to 60 minutes
	☐ Once a week	☐ One to two hours
	☐ A few times a week	☐ More than two hours
	☐ Daily	If more than two hours please specify ….. hours

3. Personal Care (washing, dressing/undressing, shaving)

☐ No – go to question 4
☐ Yes - please complete details below

Who pays for the care?

**State funded help?** Yes ☐	**If yes, approximately how often?**	**If yes, how long is a typical visit?**
No ☐	☐ Once a month	☐ Less than 30 minutes
	☐ Once a fortnight	☐ 30 to 60 minutes
	☐ Once a week	☐ One to two hours
	☐ A few times a week	☐ More than two hours
	☐ Daily	If more than two hours please specify ……. hours

**Privately funded (paid out of pocket) help?** Yes ☐	**If yes, approximately how often?**	**If yes, how long is a typical visit?**
No ☐	☐ Once a month	☐ Less than 30 minutes
	☐ Once a fortnight	☐ 30 to 60 minutes
	☐ Once a week	☐ One to two hours
	☐ A few times a week	☐ More than two hours
	☐ Daily	If more than two hours please specify ….. hours

**Unpaid help (friends/family)?**	**If yes, approximately how often?**	**If yes, how long is a typical visit?**

Yes ☐	☐ Once a month	☐ Less than 30 minutes
No ☐	☐ Once a fortnight	☐ 30 to 60 minutes
	☐ Once a week	☐ One to two hours
	☐ A few times a week	☐ More than two hours
	☐ Daily	If more than two hours please specify ….. hours

4. **During the last 3 months have friends, family or close others stayed off work to help you?**

   ☐ **No**

   ☐ **Yes**

If **yes**, approximately how many days did they take off work in the last 3 months? ☐☐

Appendix 12b 12month version

To be used at 12 month follow up capturing data over previous 6 months

Thinking about the past **6-months**, please complete below for any community based services you have used because of your **dementia** or **depression** symptoms

Primary care and community health		Tick if YES	If yes, total number of contacts in the past 6-months
General practitioner	At clinic	☐	
	By phone	☐	
	At home	☐	
Practice Nurse	At clinic	☐	
	By phone	☐	
	At home	☐	
Community/district nurse	At clinic	☐	
	By phone	☐	
	At home	☐	
	By phone	☐	

	At home	☐	
Memory Service/ Community Mental Health Team(CMHT)<br><br>Doctor	At clinic	☐I	
	By phone	☐	
	At home	☐	
Memory Service /CMHT<br><br>Nurse. Occupational Therapist or Social worker (please specify)	At clinic	☐	
	By phone	☐	
	At home	☐	
Memory Service/CMHT Psychologist	At clinic	☐	
	By phone	☐	
	At home	☐	
Memory Service/CMHT<br><br>Assistant Practitioner	At clinic	☐	
	By phone	☐	
	At home	☐	
Other community Practitioner (*please specify*)	At clinic	☐	
	By phone	☐	
	At home	☐	

Thinking about the past **6-months**, have you had any psychological therapy (appointments with a psychologist, counsellor or therapist) because of your **dementia** or **depression** symptoms? (this includes any contacts with IAPT services)

No ☐ Please skip ahead to complete the **secondary care services** section below.

Yes ☐ Please give more details below.

Was this group or individual therapy (please circle as appropriate)	**Individual / Group**
How many sessions of **individual** therapy **(1 to 1 with a therapist)** have you received in the last 6 months?	Please state number of sessions …………..……..
How many sessions of **group** therapy have you received in the last 3 months?	Please state number of sessions …………..……..
Did you pay out of your own pocket for this therapy<br><br>(or with assistance from family, etc.)?	Yes ☐ No ☐<br><br>If yes, please state the cost per session<br><br>(£) ………………………

NHS National Institute for Health Research

Camden and Islington NHS Foundation Trust

Thinking about the past 6-**months**, please complete below for any secondary care and emergency services you have used

**Emergency services**	Tick if yes	**If yes, total number of contacts in the past 6-months**
NHS direct or "Call 111"	☐	
Emergency call (999)	☐	
Paramedic only	☐	
Paramedic and ambulance to hospital	☐	
A&E attendance WITHOUT ambulance	☐	

**Overnight Inpatient stay**	Number of nights (please enter details separately for each inpatient admission)	Reason for admission
Unplanned (emergency) inpatient hospital stay for physical health reasons	Stay 1 .... nights	....
	Stay 2 .... nights	....
	Stay 3 .... nights	....
Planned (elective) inpatient hospital stay for physical health reasons	Stay 1 .... nights	....
	Stay 2 .... nights	....
	Stay 3 .... nights	....

Inpatient hospital stay for mental health reasons	Stay 1 ………………….. nights ……………………………………<br>Stay 2………………….. nights ……………………………………<br>Stay 3………………….. nights ……………………………………

**Outpatient contacts**	Tick if YES	Number of attendances in last 6 months
General medical outpatient appointment	☐	
Day patient procedure/test	☐	
Hospital Memory clinic	☐	
Psychiatric outpatient appointment	☐	

**Community Group Support**	Tick if YES	Number of attendances in last 6 months
Memory Group	☐	
Support Group provided for people with Dementia for example by AGEUK	☐	

In the past 6 months, have you gone to a **day centre?**

☐ No – please go to **Adaptations** section below

☐ Yes – please complete details below

If yes, how many times do you go to the day centre per week? ____________visits per week.

Do you pay out of pocket to attend the day centre? If yes, how much do you pay per visit? £____ per visit

Adaptations

Have you had any adaptations or changes to your home including memory aids and alarms in the past 6 months? If so, please complete below.

	**Did you pay out of pocket for this?**	**Approximately how much did this cost in the last 6 months, if known?**

		Did you pay out of pocket for this?	Approximately how much did this cost in the last 6 months, if known?
D. Adaptive equipment	☐ Shower chair<br>☐ Bath board<br>☐ Bath lift<br>☐ Over toilet frame / raised seat<br>☐ Kitchen/bathroom stool<br>☐ Bed rail/lever<br>☐ Hospital/electric bed<br>☐ Riser recliner chair<br>☐ Other, please specify:<br>……………………………………<br>……………	☐ No<br>☐ Yes (if yes please specify if known)	……………………………<br>..

		Did you pay out of pocket for this?	Approximately how much did this cost in the last 6 months, if known?
E. Mobility and memory aids	☐ Memory Aids<br>☐ Care alarm<br>☐ Walking stick<br>☐ Crutches<br>☐ Walking frame<br>☐ Walker with wheels<br>☐ Banana board/slide board<br>☐ Mobility Scooter<br>☐ Car adaptations<br>☐ Other, please specify:<br>…………………..……………… …………..	☐ No<br>☐ Yes (if yes please specify if known)	…………………………

		Did you pay out of pocket for this?	Approximately how much did this cost in the last 6 months, if known?
F. Home adaptations	☐ Ramps installed<br>☐ Rails, grab-rails<br>☐ Stair-lift<br>☐ Furniture raisers<br>☐ Other renovations, please describe<br>…………………..………………<br>…………...	☐ No<br>☐ Yes (if yes please specify if known)	

Medication

Please complete for medications related to your depression or dementia taken in the last 6 months. We are particularly interested in any medication for your mental health including antidepressants (for your mood), anti-anxiety medication to help you relax or keep calm, antipsychotics to reduce troubling thoughts or experiences or night time sleeping tablets you are prescribed.

Drug name	Dose	Units of dose	Frequency	Duration of prescription
1				

2				
3				
4				
5				
6				
7				
8				

We would like to know whether you have received help with any of the following activities. If you do receive help we would like to know *how often you receive help* and *who pays for it*.

Please think about the help you have received **over the past 6 months**.

Have you received any help for:

1. **Maintenance work and odd jobs?**

☐ No - go to question 2.

☐ Yes - please complete details below

Who pays for the care?

**State funded help?** Yes ☐ No ☐	**If yes, approximately how often?**	**If yes, how long is a typical visit?**
	☐ Once a month	☐ Less than 30 minutes
	☐ Once a fortnight	☐ 30 to 60 minutes
	☐ Once a week	☐ One to two hours
	☐ A few times a week	☐ More than two hours
	☐ Daily	If more than two hours please specify ……. hours

**Privately funded (paid out of pocket) help?** Yes ☐ No ☐	**If yes, approximately how often?**	**If yes, how long is a typical visit?**
	☐ Once a month	☐ Less than 30 minutes
	☐ Once a fortnight	☐ 30 to 60 minutes
	☐ Once a week	☐ One to two hours
	☐ A few times a week	☐ More than two hours
	☐ Daily	If more than two hours please specify ….. hours
**Unpaid help (friends/family)?**	**If yes, approximately how often?**	**If yes, how long is a typical visit?**
Yes ☐	☐ Once a month	☐ Less than 30 minutes

No ☐	☐ Once a fortnight	☐ 30 to 60 minutes
	☐ Once a week	☐ One to two hours
	☐ A few times a week	☐ More than two hours
	☐ Daily	If more than two hours please specify ….. hours

2. **Cleaning the house, ironing and cooking?**

☐ No - go to question 3.

☐ Yes - please complete details below

Who pays for the care?

**State funded help?** Yes ☐	**If yes, approximately how often?**	**If yes, how long is a typical visit?**
No ☐	☐ Once a month	☐ Less than 30 minutes
	☐ Once a fortnight	☐ 30 to 60 minutes
	☐ Once a week	☐ One to two hours
	☐ A few times a week	☐ More than two hours
	☐ Daily	If more than two hours please specify ……. hours

**Privately funded (paid out of pocket) help?** Yes ☐	**If yes, approximately how often?**	**If yes, how long is a typical visit?**
No ☐	☐ Once a month	☐ Less than 30 minutes
	☐ Once a fortnight	☐ 30 to 60 minutes
	☐ Once a week	☐ One to two hours
	☐ A few times a week	☐ More than two hours
	☐ Daily	If more than two hours please specify ….. hours

**Unpaid help (friends/family)?**	**If yes, approximately how often?**	**If yes, how long is a typical visit?**
Yes ☐	☐ Once a month	☐ Less than 30 minutes

No ☐	☐ Once a fortnight	☐ 30 to 60 minutes
	☐ Once a week	☐ One to two hours
	☐ A few times a week	☐ More than two hours
	☐ Daily	If more than two hours please specify ….. hours

3. Personal Care (washing, dressing/undressing, shaving)

☐ No – go to question 4
☐ Yes - please complete details below

Who pays for the care?

**State funded help?** Yes ☐	**If yes, approximately how often?**	**If yes, how long is a typical visit?**
No ☐	☐ Once a month	☐ Less than 30 minutes
	☐ Once a fortnight	☐ 30 to 60 minutes
	☐ Once a week	☐ One to two hours
	☐ A few times a week	☐ More than two hours
	☐ Daily	If more than two hours please specify ……. hours

**Privately funded (paid out of pocket) help?** Yes ☐	**If yes, approximately how often?**	**If yes, how long is a typical visit?**
	☐ Once a month	☐ Less than 30 minutes
No ☐	☐ Once a fortnight	☐ 30 to 60 minutes
	☐ Once a week	☐ One to two hours
	☐ A few times a week	☐ More than two hours
	☐ Daily	If more than two hours please specify ….. hours

**Unpaid help (friends/family)?**	**If yes, approximately how often?**	**If yes, how long is a typical visit?**

Yes ☐	☐ Once a month	☐ Less than 30 minutes
No ☐	☐ Once a fortnight	☐ 30 to 60 minutes
	☐ Once a week	☐ One to two hours
	☐ A few times a week	☐ More than two hours
	☐ Daily	If more than two hours please specify ….. hours

4. During the last 6 months have friends, family or close others stayed off work to help you?

☐ **No**

☐ **Yes**

If **yes**, approximately how many days did they take off work in the last 6 months? ☐☐

Appendix 13: Client Satisfaction Questionnaire - (CSQ-3)

CSQ-3

Please help us improve our programme by answering some questions about the services you have received. We are interested in your honest opinion, whether they are positive or negative. Please answer all of the questions. We also value your comments and suggestions. Thank you very much. We appreciate your help.

To what extent has our service/programme met your needs?

- ☐ 4= Almost all of my needs have been met
- ☐ 3= Most of my needs have been met
- ☐ 2= Only a few of my needs have been met
- ☐ 1= No needs have been met

In an overall, general sense, how satisfied are you with the service you received?

- ☐ 4= Very satisfied
- ☐ 3= Mostly satisfied
- ☐ 2= Indifferent of mildly dissatisfied
- ☐ 1= Quite dissatisfied

If you were to seek help again, would you come back to our programme?

- ☐ 4= Yes, definitely
- ☐ 3= Yes, I think so
- ☐ 2= No, I don't think so
- ☐ 1= No, definitely not

Appendix 14: Credibility and Expectancy question -(CEQ)

Therapy evaluation form

We would like you to indicate below how much you believe, *right now*, that the therapy you may receive will help to reduce your symptoms of depression. Belief usually has two aspects to it: (1) what one *thinks* will happen and (2) what one *feels* will happen. Sometimes these are similar; sometimes they are different. Please answer the questions below. In the first set, answer in terms of what you *think*. In the second set answer in terms of what you really and truly *feel*.

Set I

1. At this point, how logical does the therapy offered to you seem?

1 2 3 4 5 6 7 8 9

not at all logical somewhat logical very logical

2. At this point, how successfully do you think this treatment will be in reducing your symptoms of depression?

1 2 3 4 5 6 7 8 9

not at all useful somewhat useful very useful

3. How confident would you be in recommending this treatment to a friend who experiences similar problems?

1 2 3 4 5 6 7 8 9

not at all confident somewhat confident very confident

4. By the end of the therapy period, how much improvement in your symptoms of depression do you think will occur?

0% 10% 20% 30% 40% 50% 60% 70% 80% 90% 100%

Set II

For this set, close your eyes for a few moments, and try to identify what you really *feel* about the therapy and its likely success. Then answer the following questions.

1. At this point, how much do you really *feel* that therapy will help you to reduce your symptoms of depression?

1 2 3 4 5 6 7 8 9

not at all somewhat very much

2. By the end of the therapy period, how much improvement in your symptoms of depression do you really *feel* will occur?

0% 10% 20% 30% 40% 50% 60% 70% 80% 90% 100%

Therapy evaluation form- Caregiver version

We would like you to indicate below how much you believe, *right now*, that the therapy the person you care for may receive will help to reduce their symptoms of depression. Belief usually has two aspects to it: (1) what one *thinks* will happen and (2) what one *feels* will happen. Sometimes these are similar; sometimes they are different. Please answer the questions below. In the first set, answer in terms of what you *think*. In the second set answer in terms of what you really and truly *feel*.

Set I

1. At this point, how logical does the therapy offered to you seem?

1 2 3 4 5 6 7 8 9

not at all logical somewhat logical very logical

2. At this point, how successful do you think this treatment will be in reducing the symptoms of depression in the person you care for?

1 2 3 4 5 6 7 8 9

not at all useful somewhat useful very useful

3. How confident would you be in recommending this treatment to a friend who experiences similar problems?

1 2 3 4 5 6 7 8 9

not at all confident somewhat confident very confident

4. By the end of the therapy period, how much improvement in the symptoms of depression do you think will occur?

0% 10% 20% 30% 40% 50% 60% 70% 80% 90% 100%

Set II

For this set, close your eyes for a few moments, and try to identify what you really *feel* about the therapy and its likely success. Then answer the following questions.

1. At this point, how much do you really *feel* that therapy will help you to reduce the symptoms of depression for the person you care for?

1 2 3 4 5 6 7 8 9

not at all somewhat very much

2. By the end of the therapy period, how much improvement in the symptoms of depression (for the person you care for) do you really *feel* will occur?

0% 10% 20% 30% 40% 50% 60% 70% 80% 90% 100%

NHS
National Institute for Health Research

Camden and Islington NHS
NHS Foundation Trust

Appendix 15: Treatment Preference Questions

AFTER THE PATH RATIONALE HAS BEEN READ BY THE PATIENT and CAREGIVER, ASK TREATMENT PREFERENCE PRIOR TO RANDOMISATION

Treatment Preference (patient)

1. Although you will be chosen at random to have either Problem Adaptation Therapy or usual multidisciplinary care alone, if you could choose what treatment you received, how much would you hope to receive Problem Adaptation Therapy? Please rate this on a 4-point scale from 0 (not at all) to 3 (completely); circle one number.

   Rating: 0 (Not at all) 1 2 3 (Completely)

2. How much would you hope to receive usual multidisciplinary care alone (i.e. without Problem Adaptation Therapy)? Please rate this on a 4-point scale from 0 (not at all) to 3 (completely) ; circle one number.

   Rating: 0 (Not at all) 1 2 3 (Completely)

Treatment Preference (caregiver)

3. Although you will be chosen at random to have either Problem Adaptation Therapy or usual multidisciplinary care alone, if you could choose what treatment you received, how much would you hope to receive Problem Adaptation Therapy? Please rate this on a 4-point scale from 0 (not at all) to 3 (completely); circle one number.

   Rating: 0 (Not at all) 1 2 3 (Completely)

4. How much would you hope to receive usual multidisciplinary care alone (i.e. without Problem Adaptation Therapy)? Please rate this on a 4-point scale from 0 (not at all) to 3 (completely); circle one number.

   Rating: 0 (Not at all) 1 2 3 (Completely)

Appendix 16:

Qualitative Satisfaction Questionnaire:

Full RCT at 6 months post-randomisation, caregivers and therapists will complete an anonymous qualitative satisfaction questionnaire to further examine the acceptability and feasibility of the intervention (or usual multidisciplinary care for those in the TAU arm)

(The questionnaire format will allow space for answers)

a. Qualitative satisfaction questionnaire - therapists

We would be very grateful if you could answer the following questions in relation to your experiences of delivering the intervention to people with depression and dementia. Please answer as many questions as you can – any information we receive from you, no matter how small, will be helpful.

1. How many patient-carer dyads did you deliver the intervention to?

____

2. Overall, how satisfied were you with the intervention?

Not at all **Extremely**

1 2 3 4 5 6 7 8 9 10

3. Overall, how useful did you find the intervention?

Not at all **Extremely**

1 2 3 4 5 6 7 8 9 10

4. Overall, how acceptable was the intervention to this client group?

Not at all									Extremely
**1**	**2**	**3**	**4**	**5**	**6**	**7**	**8**	**9**	**10**

5. Overall, how feasible was it to deliver the intervention to this client group?

Not at all									Extremely
**1**	**2**	**3**	**4**	**5**	**6**	**7**	**8**	**9**	**10**

We would now like to ask you some specific questions about your experiences of delivering the intervention to people with depression and dementia.

6. Overall, how would you describe your experience of delivering the intervention?

7. What did you like or dislike about delivering the intervention? Why?

8. How easy was it to deliver the intervention? What was easier? What was more difficult? Why?

9. How understandable was the intervention to the patient-carer dyads you were working with? What was understandable? What was confusing?

10. How acceptable do you think your patient-carer dyads found the intervention? What seemed to be acceptable? What was less so?

11. What emotional, behavioural or cognitive changes did you see in the patient-carer dyads that you worked with over the course of the intervention?

12. What helped you deliver the intervention to this client group? What helped patient-carer dyads engage with the intervention?

13. What got in the way of delivering the intervention to this client group? What barriers were there to patient-carer dyads engaging with the intervention?

14. What did you think about the practical aspects of delivering the intervention (such as the number and frequency of sessions, or the setting in which the intervention was delivered)?

15. Have you experienced any changes in your professional practice as a result of delivering the intervention? If yes, what?

16. Would you recommend the intervention to other therapists for this client group? Why or why not?

17. How did the intervention meet your expectations as a therapist? Did you achieve all that you had hoped to achieve as a therapist with the intervention? Why or why not?

18. If you could, what would you change about the intervention?

19. How could we optimise engagement with the intervention? How could we optimise delivery of the intervention?

20. Would another form of psychological therapy have been more helpful for this client group? If yes, what?

If you have any other comments about the intervention, then please write them in the box below:

b. Qualitative satisfaction questionnaire - carer (usual care group)

We would be very grateful if you could answer the following questions about your experiences and the experiences of the person you care for in relation to how their mental health and emotional wellbeing is being managed within their usual care. If you are uncertain about the experiences of the person you care for then please just answer the questions from your perspective. Please answer as many questions as you can – any information we receive from you, no matter how small, will be helpful.

1. Is the person you care for currently receiving any support with respect to their mental health and emotional wellbeing from the Memory Service, GP, or other services? If yes, what and who is it being provided by?

2. Overall, how satisfied are you and the person you care for with how their mental health and emotional wellbeing is being supported within the Memory Service, GP, or other services?

Not at all **Extremely**

1 2 3 4 5 6 7 8 9 10

3. If the person you care for is receiving support with respect to their mental health and emotional wellbeing, how useful is this?

Not at all **Extremely**

1 2 3 4 5 6 7 8 9 10

4. If the person you care for is receiving support with respect to their mental health and emotional wellbeing, how suitable or acceptable is this to you both? How much is it meeting both of your needs?

Not at all **Extremely**

1 2 3 4 5 6 7 8 9 10

5. If the person you care for is receiving support with respect to their mental health and emotional wellbeing, how easy is it to receive this support from a practical point of view?

Not at all **Extremely**

1 2 3 4 5 6 7 8 9 10

6. If the person you care for is not receiving any support with respect to their mental health and emotional wellbeing, what kind of support would they or you like them to receive? What would that look like?

If you have any other comments about how the mental health and emotional wellbeing of the person you care for is currently being supported or should be supported, then please write them in the box below:

Thank you for sparing the time to answer these questions. It is greatly appreciated. Your responses will be extremely useful in helping us to modify and improve our care of people with dementia and depression.

c. Qualitative satisfaction questionnaire - carer (intervention group)

We would be very grateful if you could answer the following questions about your experiences and the experiences of the person you care for in relation to receiving Problem Adaptation Therapy. If you are uncertain about the experiences of the person you care for then please just answer the questions from your perspective. Please answer as many questions as you can – any information we receive from you, no matter how small, will be helpful.

1. How many sessions of therapy did you and the person you care for receive?

____

2. Overall, how satisfied were you and the person you care for with the therapy?

Not at all **Extremely**

1 2 3 4 5 6 7 8 9 10

3. Overall, how useful did you and the person you care for find the therapy?

Not at all **Extremely**

1 2 3 4 5 6 7 8 9 10

4. Overall, how suitable or acceptable was the therapy to you and the person you care for? How much did it meet both of your needs?

Not at all **Extremely**

1 2 3 4 5 6 7 8 9 10

5. Overall, how easy was it for you and the person you care for to take part in the therapy from a practical point of view?

Not at all **Extremely**

1 2 3 4 5 6 7 8 9 10

We would now like to ask you some specific questions about your experiences and the experiences of the person you care for of receiving the therapy.

6. Overall, how would you and the person you care for describe your experience of receiving the therapy?

7. How helpful did you and the person you care for find the therapy? What was helpful? What was not helpful?

8. What did you and the person you care for like about the therapy? What did you and the person you care for dislike about the therapy?

9. Have you or the person you care for experienced any changes as a result of receiving the therapy (such as changes in how you think about things or feel about life, changes in your wellbeing or health, changes in your relationships with others, or changes in your day-to-day life, leisure activities or hobbies)? If yes, what?

10. How easy to understand was the therapy? What made sense? What was confusing?

11. To what extent do you think the therapy met your needs and the needs of the person you care for? How did it meet both of your needs? How did it not meet both of your needs?

12. What did you and the person you care for think about the practical aspects of how the therapy was delivered (such as the number and frequency of sessions, or the setting in which the therapy was delivered)?

13. What helped you and the person you care for to take part in the therapy or 'give it a go'? What got in the way of you and the person you care for taking part in the therapy or 'giving it a go'?

14. Would you and the person you care for recommend the therapy to a friend who was experiencing similar difficulties to you? If yes, why? If no, why not?

15. Is there anything you or the person you care for would like to change about the therapy? If yes, what?

If you have any other comments about the therapy that you and the person you care for received, then please write them in the box below:

Thank you for sparing the time to answer these questions. It is greatly appreciated. Your responses will be extremely useful in helping us to modify and improve our care of people with dementia and depression.
