# Supplementary material for: Adapted problem adaptation therapy for depression in mild to moderate Alzheimer's disease dementia: A randomized controlled trial
Source: Alzheimers Dement. 2024 Mar 13;20(4):2990–9. doi: 10.1002/alz.13766 (PMC11032547; doi:10.1002/alz.13766)
Supplement: Supplementary file 4 — Statistical analysis plan [file ALZ-20-2990-s006.pdf]

PATHFINDER Trial
Statistical Analysis Plan

Version 0.6
20/01/20223

1 Introduction

1.0 Trial full title

Problem Adaptation Therapy for individuals with mild to moderate dementia and depression. The PATHFINDER Trial.

1.1 Purpose

This statistical analysis plan (SAP) contains details of the main statistical analyses for the PATHFINDER trial. These analyses are pre-specified in order that they are not influenced by the collected trial data after unblinding. This SAP describes the statistical analysis of the clinical outcomes. It does not contain details of any qualitative analyses.

The SAP does not preclude the undertaking of further ad hoc or exploratory analyses, although the results of any such analyses should be interpreted with caution. Furthermore, the SAP does not preclude the adaptation of any part of the trial analysis should situations arise in which such adaptation is deemed necessary. Any such adaptation will be transparent and fully justified.

This SAP contains only a brief overview of the trial design, population, intervention, comparison and outcome variables. More detail can be found in the protocol version v6.0 dated 12 May 2020 which is stored on S:\FPHS_Priment_CTU\Projects\Current\Non CTIMPS\Pathfinder PR0223 - Rob Howard\Feasibility Phase\6. Protocol\Current version.

1.2 Protocol version

This SAP has been written based on the information protocol version 6.0 dated 12th of May 2020.

1.3 Trial registration

The trial was prospectively registered with ISRTCN (ISRCTN11185706) and ClinicalTrials. gov (NCT04241796).

1.4 Authorship

The SAP has been written by Rebecca Jones (RJ), Nick Freemantle (NF), Mariam Adeleke (MA) and Martin Wiegand (MW).

1.5 SAP revisions

Version 0.1 dated 03/11/2020
Version 0.2 dated 13/01/2022
Version 0.3 dated 17/01/2022
Version 0.4 dated 07/06/2022
Version 0.5 dated 19/12/2022
Version 0.6 dated 20/01/2023

1.6 Signatures

The undersigned confirm that the following Statistical Analysis Plan has been agreed and accepted and that the Trial Statistician agrees to conduct the analysis in compliance with the approved Statistical Analysis Plan. Major deviations from the Plan will be agreed in advance before implementation. All deviations from the Plan will be explained, documented and reported accordingly.

Authorised by	Signature	Date
**Prof. Robert Howard,** Chief investigator	[signature]	2nd February 2023
**Prof. Nicholas Freemantle,** Lead statistician	Nick Freemantle	Digitally signed by Nick Freemantle<br>DN: cn=Nick Freemantle, o=UCL, ou=CCTU, email=nicholas.freemantle@ucl.ac.uk, c=GB<br>Date: 2023.07.24 16:33:16 +01'00'

2 Contents

1 Introduction 1
1.0 Trial full title 1
1.1 Purpose 1
1.2 Protocol version 1
1.3 Trial registration 1
1.4 Authorship 1
1.5 Signatures 2
3 List of Abbreviations 4
4 Trial Summary 5
4.0 Aims 5
4.1 Population 5
4.1.1 Inclusion criteria 5
4.1.2 Exclusion criteria 5
4.2 Intervention 5
4.3 Comparison .... **Error! Bookmark not defined.**
4.4 Primary outcome 6
4.5 Design 6
4.6 Sample size 6
4.7 Randomisation 6
4.8 Blinding 6
5 Summary of Quantitative Trial Data 6
5.0 Observation times 6
5.1 Outcome measures 7
5.1.1 Primary outcome 7
5.1.2 Secondary outcomes 7
5.1.3 Outcome Missingness .... **Error! Bookmark not defined.**
5.2 Other available data 8
6 Statistical Analyses of Clinical Outcomes 9
6.0 Organisation of data and analyses 9
6.1 Interim analyses 9
6.2 Recruitment and retention 9
6.3 Description of demographic variables at baseline 10
6.4 Primary outcome analysis 10
6.4.1 Confidence intervals 11
6.4.2 Missing data 11
6.5 Secondary analyses 11
6.6 Subgroup analyses 12
6.7 Adverse events 12
6.8 Reporting 12
7 References 12

3 List of Abbreviations

AD	Alzheimer's disease
BADLS	Bristol Activities of Daily Living Scale
CI	Confidence interval
CONSORT	Consolidated standards of reporting trials
CSDD	Cornell Scale for Depression in Dementia
CSQ	Client Satisfaction Questionnaire
DEMQOL	Quality of Life in Dementia scale
GHQ	General Health Questionnaire
IDMC	Independent Data Monitoring and Ethics Committee
NHS	National Health Service
PATH	Problem adaptation therapy
RAID	Rating Anxiety in Dementia
SAP	Statistical analysis plan
SD	Standard deviation
sMMSE	Standardised Mini Mental State Examination

4 Trial Summary

4.0 Aims

The specific objectives of the PATHFINDER study are as follows:

- Adapt and manualise problem adaptation therapy (PATH) so that it is accessible and acceptable to people with mild to moderate dementia and their caregivers and can be delivered by existing staff in NHS secondary care.
- Obtain quantitative estimates of the accessibility, acceptability, credibility and feasibility of the PATH intervention.
- Establish the clinical and cost effectiveness of adapted PATH plus usual care compared to usual care alone (carried out by the health economists of the trial management group).
- Use qualitative approaches to explore the intervention's acceptability to people with dementia and their caregivers, as well as to therapists during the intervention.

4.1 Population

Individuals with mild to moderate dementia and depression.

4.1.1 Inclusion criteria

People aged over 50 years with mild to moderate dementia, a diagnosis of probable Alzheimer disease (AD), or mixed AD and vascular dementia, and clinically significant depression, who are sufficiently fluent in English to engage with the PATH intervention, live in their own home and have a caregiver who agrees to act as co therapist.

4.1.2 Exclusion criteria

Individuals are not eligible for entry to the trial if they have been diagnosed with other dementias (including dementia with Lewy bodies, Parkinson's disease dementia and frontal temporal dementia), if they started or changed dose of antidepressant or other psychotropic medication in the four weeks prior to recruitment or plan to change treatment during the next 12 weeks, if they are currently engaged in formal psychological therapy, if they require treatment for a severe psychiatric disorder such as schizophrenia or bipolar disorder, or if they are severely depressed with suicidal ideation, behaviour or intent.

More detailed definitions of probable Alzheimer's disease, dementia severity and participants' therapeutic needs for the purpose of eligibility criteria for this study can be found in the protocol version 0.6.

4.2 Intervention

Up to eight manualised, face to face sessions of the PATH intervention delivered over 12 weeks (approximately weekly) plus usual multidisciplinary care. Sessions will comprise two assessment sessions, five sessions focused on problem solving using PATH tools and one review session, with each session lasting up to one hour.

4.3 *Control*

Usual multidisciplinary care alone.

4.4 *Primary outcome*

Depression symptoms measured using the Cornell Scale for Depression in Dementia [3] (CSDD) at six months post randomisation.

4.5 *Design*

A multicentre, single blind, parallel, two arm, randomised controlled trial to assess the clinical and cost-effectiveness of prescription of adapted PATH for depression in mild and moderate dementia with a 12 month internal pilot to assess feasibility of recruitment and acceptability of randomisation.

4.6 *Sample size*

The sample size calculation indicates that 334 participants will allow detection of a 0.4 SD effect size (corresponding to a 2.0 point difference on the CSDD which is considered to be a minimum clinically important difference) with a 2-sided alpha of 5% and 90% power and assuming 20% loss to follow-up at 6 months.

4.7 *Randomisation*

Participants will be randomly assigned in a 1:1 ratio to the PATH intervention plus usual multidisciplinary care or usual multidisciplinary care alone. Randomisation will be stratified by baseline use of antidepressant medication using block randomisation with varying block sizes. Full details can be found in the randomisation protocol (S:\FPHS_Priment_CTU\Projects\Current\Non CTIMPS\Pathfinder PR0223 - Rob Howard\Feasibility Phase\8. DM\Sealed Envelope Systems\).

4.8 *Blinding*

This is a single blind trial. Assessors are blind to treatment allocation; participants and therapists are not. Statisticians will also be blinded to allocation as far as possible until after the primary analysis has been agreed. MW will attend the Independent Data Monitoring and Ethics Committee (IDMC) and may become unblinded as a result of this role if the committee requires any statistics to be reported separately by study arm.

5 Summary of Quantitative Trial Data

5.0 *Observation times*

Data will be collected at the following time points during the trial:

- Baseline (before treatment)

- 3 months post randomisation (post treatment)
- 6 months post randomisation (primary endpoint)
- 12 months post randomisation

Not all measures will be recorded at every time point. The data recorded at baseline and at 6 months post treatment will constitute the full dataset for the purpose of analysis of the primary outcome. At each time point beyond baseline there is a data collection window of -2 weeks / +4 weeks. This has been extended from a planned window of -2 weeks/+2 weeks due to instances where the data collection was delayed by mandatory isolation of participants or study partners due to Covid-19. Data will be considered recorded at a given time point provided that these data are collected from each participant within this window. Any participants for whom data are not collected within this window will be considered missing at that time point for the purpose of the statistical analysis. The number and percentage of observations excluded for being outside the relevant time window will be summarised separately by study arm for the primary outcome only.

5.1 Outcome measures

5.1.1 Primary outcome

The primary outcome is depression symptoms as measured by the Cornell Scale for Depression in Dementia (CSDD) at 6 months post randomisation.

5.1.2 Secondary outcomes

- Depression symptoms at other follow up time points (3 and 12 months post randomisation), also measured by the CSDD
- Dementia related quality of life measured by the DEMQOL administered to patients and the DEMQOL proxy administered to caregivers
- Health related quality of life measured by the EQ-5D-5L
- Functional abilities measured by the Bristol Activities of Daily Living Scale (BADLS)
- Cognitive function measured by the Standardised Mini Mental State Examination (sMMSE)
- Anxiety measured by the Rating Anxiety in Dementia scale (RAID)
- Caregiver burden measured by the Zarit Burden Inventory
- Caregiver common mental disorders measured using the General Health Questionnaire (GHQ-12)
- Satisfaction with treatment measured by the Client Satisfaction Questionnaire (CSQ)

Further details on the scoring and ranges of all outcomes can be found in the trial protocol version v6.0 stored on S:\FPHS_Priment_CTU\Projects\Current\Non CTIMPS\Pathfinder PR0223 - Rob Howard\Feasibility Phase\6. Protocol\Current version. Table 1 provides an overview of primary and secondary outcomes and the time points at which they will be collected.

Table 1: Data collection measures and time points

Measure	Baseline	3 months follow up	6 months follow up (primary endpoint)	12 months follow up
**Primary outcome**				
Depression symptoms (CSDD)	✓	✓	✓	✓
**Secondary outcomes**				
Dementia related quality of life (DEMQOL and DEMQOL proxy)	✓	✓	✓	✓
Health related quality of life (EQ-5D-5L)	✓	✓	✓	✓
Functional abilities (BADLS)	✓	✓	✓	✓
Cognitive function (sSSME)	✓	✓	✓	✓
Anxiety (RAID)	✓	✓	✓	✓
Caregiver burden (Zarit Burden Inventory)	✓	✓	✓	✓
Caregiver common mental disorders (GHQ-12)	✓	✓	✓	✓
Satisfaction with treatment (CSQ)	✓	✓	✓	✓
Serious adverse events	✓	✓	✓	✓
**Other measures**				
Credibility/Expectancy	✓			
Treatment preference	✓			
Checklist of PATH components, techniques and themes		✓		
Assessment of blindness				✓

Notes:

5.2 Other available data

Demographic characteristics of the participants and their caregivers will be collected at baseline in order to assess the balance between the study arms. This information will include:

- Date of birth (age)
- Gender
- Ethnicity
- Marital status
- Highest educational qualification
- Work and socioeconomic status

Other available data will comprise:

- Centre identifier
- Baseline use of antidepressant medication (stratification factor)
- Dates of assessments
- Treatment preference
- Treatment credibility
- Treatment satisfaction
- Concomitant treatments and medications
- Reasons for withdrawal or loss to follow-up (if supplied)

6 Statistical Analyses of Clinical Outcomes

6.0 Organisation of data and analyses

The SAP will be finalised and approved prior to unblinding. The programs and code to be used for statistical analyses will be prepared prior to unblinding as far as possible. Two statisticians will perform the analysis relating to the primary outcome independently, to ensure its accuracy.

Prior to performing analyses, basic checks will be performed by the statisticians on the blinded data to ensure accuracy. Each outcome (primary and secondary) variable and baseline demographic variable will be checked for:

- Missing values
- Values outside an acceptable range
- Other inconsistencies

If missing values or other inconsistencies are found, the corresponding data will be sent to the Trial Manager for checking and will either be corrected, deemed to be missing or confirmed correct, as appropriate.

6.1 Interim analyses

Baseline demographic characteristics will be summarised by study arm for the IDMC. Summary statistics and data completeness for primary and secondary outcomes at baseline and follow up time points will likewise be summarised by study arm. There will be no interim analysis involving testing of the treatment efficacy, that would require adjustment in the primary analysis.

6.2 Recruitment and retention

A CONSORT diagram will be presented to provide a detailed description of participant numbers at each time point during the trial. In addition, a table summarising the number of participants who have been lost to follow up at each stage of the trial and reasons for loss to follow up (if supplied) will be presented.

6.3 *Description of demographic variables at baseline*

The demographic information collected at baseline will be presented in a table summarised separately by study arm. Categorical variables will be reported as counts and percentages. Continuous variables will be summarised as means and standard deviations (SD) or medians and interquartile ranges as appropriate depending on the distribution of the data. No statistical tests will be performed to assess baseline differences between study arms.

We will include the following covariates:

**Covariate**	**Measure**	**Units**
Demographics		
Age at randomisation	Median/ IQR/ range	Year
Sex	Number/ percentage	Binary
Ethnicity	Number/ percentage	Stratified by group
Education	Mean/ SD	Year
Highest completed degree	Number/ percentage	Stratified by group
Occupational attainment	Number/ percentage	Stratified by group
Marital status	Number/ percentage	Stratified by group
Medical characteristics		
Ongoing medication use	Number/ percentage/ mean dose/ SD	Stratified by type of medication
Time since diagnosis/ symptom onset	Mean/ SD	Months
CSDD baseline	Mean/ SD	Points
CSDD at 6 months	Mean/ SD	Points
DEMQOL	Mean/ SD	Points
EQ-5D-5L	Mean/ SD	Points
Participant preferences		
Expectancy questionnaire	Percentage of categorical response	Ordinal
Treatment preference	Count/ percentage	Binary

6.4 *Primary outcome analysis*

The primary outcome is depression symptoms on the CSDD at 6 months post randomisation. The primary analysis will be based on available data and conducted according to the intention to treat principle. The mean difference in the primary outcome in the intervention arm compared with the control arm will be estimated from a linear mixed model with measures of CSDD at 6 months as the outcome. The main explanatory variables will be the assigned study arm, and baseline use of antidepressant medication (stratification factor) and baseline CSDD. Potential clustering within study site will be accounted for by a random intercept of site.

$$CSDD6_{ij} = \beta_0 + \beta_1 TRT_{ij} + \beta_2 CSDD0_{ij} + \beta_3 AD_{ij} + u_j + \epsilon_{ij} \,;$$

CSDD6 and CSDD0 are the scores collected at 6 months and baseline, respectively. TRT is the intention to treat indicator (1 = PATH & TAU, 0 = TAU) and AD is the stratification factor of antidepressant prescription. The random error term $\epsilon_{ij} \sim N(0, \sigma_{ij})$ is assumed normally distributed and u_j denotes a random intercept for each site. The indices $j = 1, \ldots, k$ denote the cluster, while $i = 1, \ldots, n_j$ the participant within the cluster.

6.4.1 Confidence intervals

The estimated treatment effect will be reported with accompanying 95% confidence interval (CI) and p value. All reported confidence intervals and tests will be conducted two-sided.

6.4.2 Model checking

The statistical model for the primary outcome analysis includes an assumption that the residuals are normally distributed. This assumption will be checked through the construction of appropriate histograms and normal quantile plots. If these plots suggest that residuals are not normally distributed, then appropriate transformations of the primary outcome or application of the bootstrap method will be considered.

6.4.3 Missing data

Bias due to missing data will be investigated by comparing the baseline characteristics of participants with and without missing values, according to the same summary statistics chosen for the description of demographic baseline characteristics. Depending on the quantity of missing values, the predictors of missingness will be identified. We will then perform a sensitivity analysis by including any predictors of missingness as explanatory variables in the primary outcome model, in order to support the missing at random assumption. Multiple imputation will not be performed, since it offers few advantages for missing outcome data[1].

The primary analysis will be a complete case analysis. Analyses investigating the impact of missing data will be considered supportive.

6.5 Secondary analyses

Secondary outcomes are listed above and comprise depression symptoms (the primary outcome) at 3 and 12 months post randomisation and all secondary outcome measures at 3, 6 and 12 months post randomisation.

The effect of the intervention on secondary outcomes will be estimated using separate multilevel linear regression models for each outcome analogous to those described above for the primary outcome.

Non normally distributed continuous outcomes will be analysed using the bootstrap method and bias corrected 95% CI will be reported.

These analyses will be considered supportive and secondary outcomes will be analysed using available data only.

6.6 Subgroup analyses

To investigate whether participant attitude towards PATH therapy affects efficacy we will conduct the primary analysis stratified by positive and negative expectation towards the treatment. If a signal can be observed, we may conduct further effect modification analysis.

6.7 Adverse events

The number, nature and severity of serious adverse events (if any) will be reported separately by study arm at each follow up time point. The number of participants who experience adverse events will likewise be reported separately by study arm.

6.8 Reporting

Analyses will be reported with regard to the CONSORT checklist[2] and with any particular requirements of academic journals and the funders to which the results of analyses are submitted.

7 References

1. Sullivan TR, White IR, Salter AB, et al. Should multiple imputation be the method of choice for handling missing data in randomized trials? Stat Methods Med Res 2018;**27**(9):2610-26.

2. Schulz KF, Altman DG, Moher D. CONSORT 2010 Statement: updated guidelines for reporting parallel group randomised trials. BMJ 2010;**340**:c332.

3 Alexopoulos GS, Abrams RC, Young RC, Shamoian CA. Cornell Scale for Depression in Dementia. Biol Psychiatry. 1988;23(3):271–284.
